# Cooperative metathesis of H–H/Sn–C^Ar^ bonds in stannylene-Ni^0^ systems

**DOI:** 10.1039/d5sc09496h

**Published:** 2026-02-25

**Authors:** Jonas M. Gilch, Philip M. Keil, Mustapha Iddrisu, Tibor Szilvási, Terrance J. Hadlington

**Affiliations:** a Fakultät für Chemie, School of Natural Sciences, TU München Lichtenberg Strasse 4 85749 Garching Germany terrance.hadlington@tum.de; b Department of Chemical and Biological Engineering, University of Alabama Tuscaloosa AL 35487 USA tibor.szilvasi@ua.edu

## Abstract

The reaction of phosphine-appended (amido)(aryl)stannylenes (1) with Ni^0^ synthons leads to the facile formation of chelating-stannylene Ni^0^ complexes (2–4). Utilising the carbene-stabilised synthon IPr·Ni·(*η*^6^-toluene) (IPr = [(H)CN(Dipp)]_2_C:; Dipp = 2,6-^i^Pr_2_C_6_H_3_) leads to the high-yielding formation of targeted 16-electron Ni^0^ complexes. These systems activate H_2_ under non-forcing conditions (1 bar, RT), all forming the same single product, 5, which is found to be a mono-hydrido stannylene complex. Alternative synthetic routes in combination with computational calculations demonstrate that this species features a bridging (*i.e.* [Sn-(µ-H)-Ni]) hydride ligand, and may be described either as an agostic [Sn–H–Ni] bonded hydrido-stannylene complex, or a formal nickelo-stannylene. The formation of compound 5 arises from the metathesis of the Sn–C^Ar^ bonds with H_2_, leading to the elimination of Ar–H, which is detected by NMR and mass spectroscopic methods. The mechanism for this process, explored using DFT methods, proceeds through Ni-centre H_2_ binding, Sn–Ni cooperative hydrogen activation, and subsequent Ar–H elimination *via* a cooperative C–H bond formation at the Ni and Sn centres. Finally, complex 5 is shown to undergo further Ph-H elimination of a single aryl group of the chelating phosphine arm with the bridging hydride ligand, forming a unique nickelo-stannylene complex 7, which features two formally Sn^0^ metallostannylene centres binding Ni^II^.

## Abstract 2

Sn(ii) and Ni(0) in a stannylene → Ni complex cooperatively activate H_2_, leading to Sn–C/H–H bond metathesis. The formed H-bridged nickela-stannylene is indicative of net reduction at Sn, *via* a formal oxidative addition/reductive elimination process.

## Graphical abstract

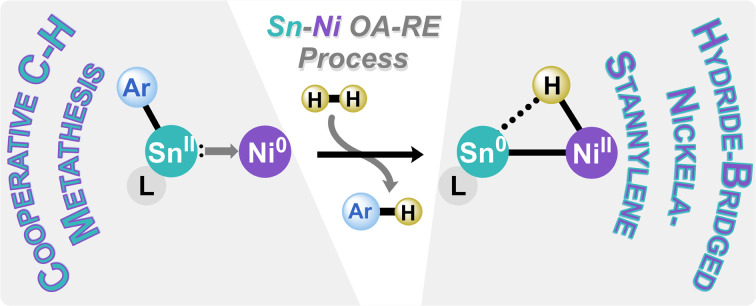

## Introduction

Dihydrogen is a key small-molecule across numerous facets of modern chemistry,^1–3^ centred perhaps most prominently in hydrogenation and dehydrogenation catalysis.^4–8^ Developing an understanding of these processes becomes increasingly more important in searching for sustainable, efficient catalysts which focus on abundant 3d metals and main group elements.^9–12^ In addition, reaction discovery is also of great importance in the development of new reactive mechanisms which involve dihydrogen activation. A prolific ligand class which has been truly pivotal in both 3d-metal and main group chemical discovery are *N*-heterocyclic carbenes (NHCs), the steric and electronic tunability of which is unparalleled amongst commonly applied ligand classes.^13–17^ Notably, NHCs bear typically stronger σ-donation when compared with phosphines and related popular donor ligands.^18^ Taking this further, one can look towards heavier tetrylenes – these have been computationally shown to be both stronger σ-donors and π-acceptors than NHC systems,^19,20^ though their utility as ligands in catalysis is significantly less explored.^21,22^ In this regard, they represent potential non-innocent (metallo-)ligand systems – broadly speaking, non-innocent ligands have led to the development of numerous novel bond activation pathways,^23–25^ including dihydrogen activation at ligand-3d metal interfaces.^26–32^ Here, the ligand plays an active role in H–H bond scission, typically resulting in ligand protonation in commonly employed systems.^33^ Due to the more electropositive nature of the heavier group 14 elements (*e.g.* relative to C/N/O), low-valent heavier group 14 element centres have been shown to direct ligand-centred nucleophile binding in their transition metal complexes.^34–37^ This combination of (a) their unique σ-donor and π-acceptor properties, and (b) their electropositive and chemically non-innocent behavior, makes them a promising class of ligand for the development of novel reactive pathways.

**Fig. 1:**
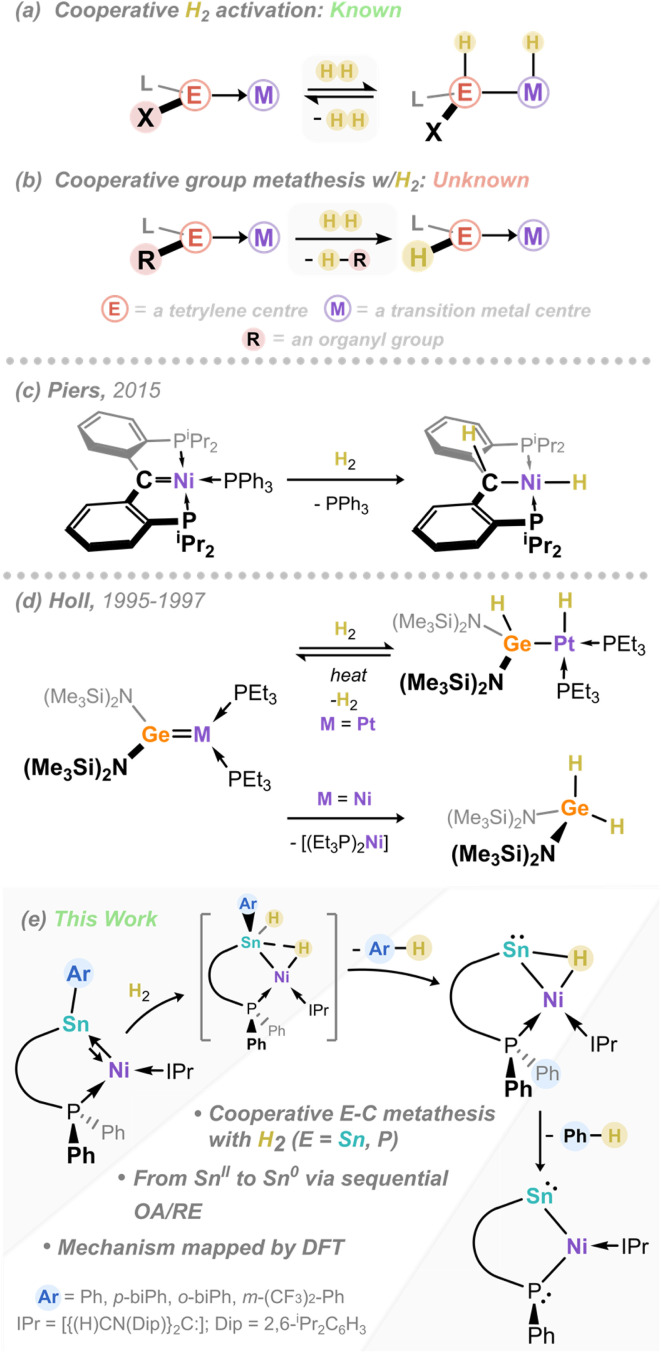
(a) Known (reversible) cooperative H_2_ activation processes in tetrylene-TM systems and (b) unknown H_2_ activation and group metathesis; (c) and (d) Examples of cooperative H_2_ activation in tetrylene-transition metal complexes, and (e) this work.

A number of low-valent group 14-element transition metal complexes are known to activate dihydrogen, typically proceeding across the E–TM bond (*viz.*Fig. 1(a); E = C–Si; TM = transition metal), though this remains uncommon. For carbon derivatives, examples from the groups of Piers,^26^ Roesler,^27^ Milstein,^28^ and Young^30^ all demonstrate the activation of H_2_ at the carbene-Ni/Fe interface (*e.g.*Fig. 1(c)). In heavier systems, an early example from Holl and co-workers demonstrated that dihydrogen activation is reversible at the Pt–Ge interface,^38^ whilst moving to the analogous Ni–Ge system led to elimination of the dihydrogermane, [(Me_2_Si)_2_N]_2_GeH_2_ (Fig. 1(d)).^39^ A number of examples of metallotetrylene/tetrylidyne complexes are also known to cleave H_2_.^36,40,41^ We've demonstrated in our own recent work that, through the electronic tuning of germylene ligands, one can tune the thermodynamics of reversible dihydrogen activation at the Ni–Ge interface.^42^ However, related systems which demonstrate divergent reactivity following H_2_ activation, *e.g.* H-atom transfer processes, are essentially unknown (*e.g.*Fig. 1(b)). Such reactivity would have direct consequences in bond functionalisation catalysis, and as such is key step in the exploration of novel cooperative catalysis pathways.

Herein we describe our efforts in the development of a family of chelating (amido)(aryl)stannylene ligands, and their utility in accessing 16-electron Ni^0^ complexes (Fig. 1(e)). We find that, although these complexes do rapidly and cooperatively activate dihydrogen, the reaction outcome diverges from described C^II^ and Ge^II^ – in all cases, protonated arene elimination if observed, representing a previously unobserved mechanism in cooperative H_2_ activation. This leads to a rare example of a H-bridged (hydrido-tetrylene)-transition metal complex,^41,43–45^ which can also be described as a metallotetrylene, through formal oxidative addition of the Sn–H bond at Ni. Efforts towards fully defining this unique process, which involves cooperative dihydrogen activation, are described, including labelling studies and computational reaction profiling. Finally, further P–Ar elimination is observed, forming a unique nickelo-bis(stannylene) complex. These findings define an unprecedented Sn–Ni cooperative metathesis process, through mild C–H bond forming reductive elimination reactions.

### Synthesis of (amido)(aryl)stannylenes and their 16-e^−^ Ni^0^ complexes

**Scheme 1:**
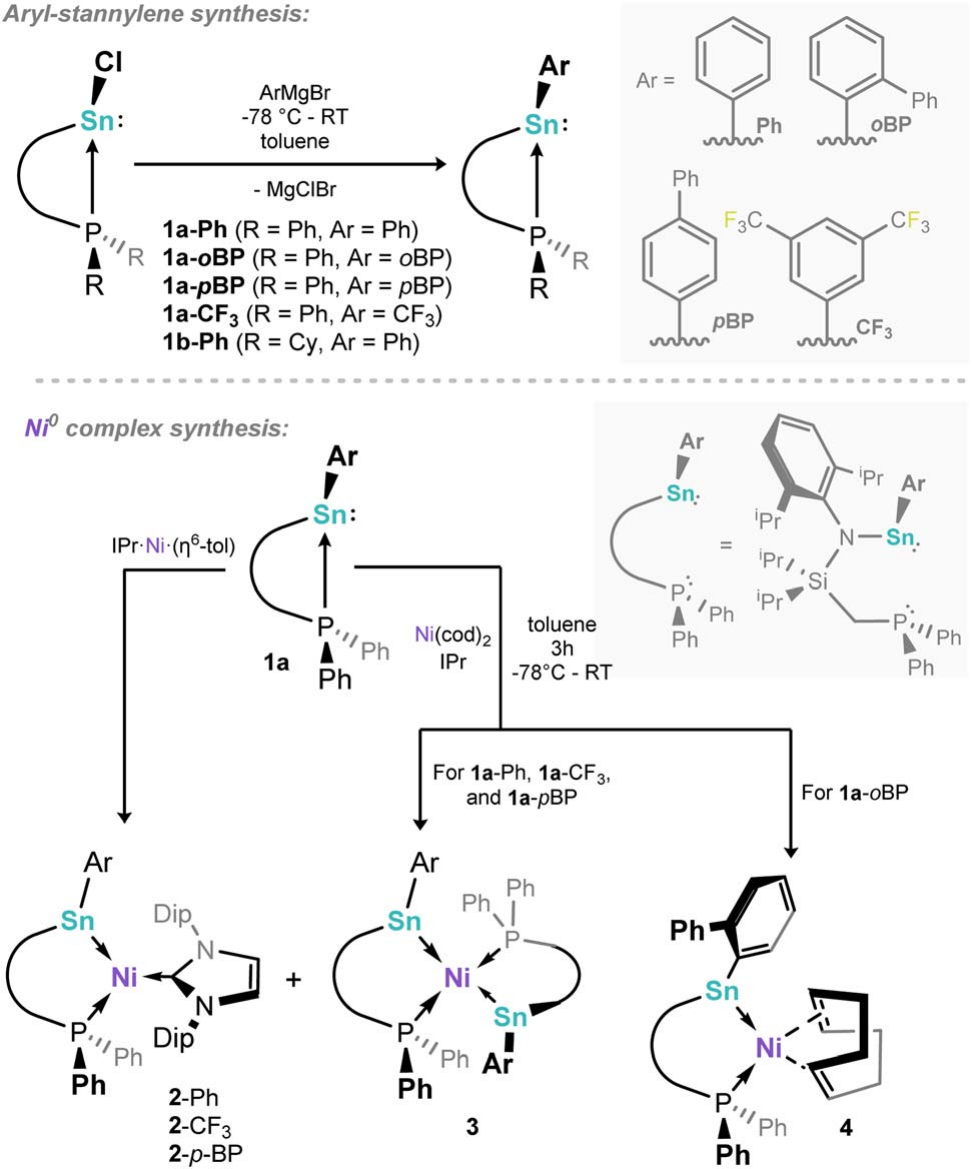
Synthesis of phosphine-appended aryl-stannylene ligands, and the complexation behaviour of ligands 1a.

Our study started with the synthesis of a family of (amido)(aryl)stannylenes, ^Ph^L(Ar)Sn: (^Ph^L = {[Ph_2_PCH_2_Si(^i^Pr)_2_](Dipp)N}; Dipp = 2,6-^i^Pr_2_C_6_H_3_), which can be accessed in good yield *via* the addition of aryl Grignard reagents to the chloro stannylenes ^Ph^L(Cl)Sn:.^46^ Here we describe the phenyl (1a-Ph), 3,5-bis(trifluoromethyl)phenyl (1a-CF_3_), *para*-biphenyl (1a-*p*BP), and *ortho*-biphenyl (1-*o*BP) substituted stannylenes (Scheme 1, top), demonstrating varying electronic and steric properties. Additionally, ^Cy^L(Ph)Sn: was synthesised (1b-Ph; ^Cy^L = {[Cy_2_PCH_2_Si(^i^Pr)_2_](Dipp)N}; Dipp = 2,6-^i^Pr_2_C_6_H_3_), to introduce a more strongly donating Cy_2_P-arm. One expects the increasing electron withdrawing nature of the aryl substituent to lead to an increased deshielding of the phosphorous centre, as per our earlier reported Ge^II^ systems.^42^ With the ^Ph^L-supported phenyl-stannylene 1a-Ph as a point of reference (^31^P NMR: *δ* = −4.4 ppm), the most significant high-field shift is found for 1a-CF_3_ (*δ* = −1.0), whilst *p*-biphenyl system 1a-*p*BP demonstrates a similar shift to 1a-Ph (*δ* = −4.2 ppm), and 1a-*o*BP demonstrates a significantly more low-field shift than 1a-Ph (*δ* = −6.2), which would surprisingly suggest that the *ortho-*biphenyl substituent is less electron withdrawing than phenyl. We hypothesise that this is a steric factor, whereby a co-planar *o*-Ph group is disfavoured, reducing electronic communication. Moving to the ^Cy^L-supported stannylene 1b-Ph, a lower field ^31^P NMR shift is observed already for the free ligand and the chloro-stannylene, relative to those and ^119^Sn satellites, however, indicate the strength of the Sn–P bond in these two ligand systems: 1a-Ph shows ^1^*J*_PSn_ values of 1291 (^117^Sn) and 1350 (^119^Sn) Hz, comparing to 1495 (^117^Sn) and 1565 (^119^Sn) Hz for 1b-Ph. This is indicative of a stronger P → Sn donor interaction in the latter, due to the stronger σ-donor character of alkyl phosphines, which may affect its utility in metal ligation. X-ray structures of 1a-Ph, 1a-CF_3_, and 1b-Phshow strongly pyramidalised Sn-centres (*e.g.* sum of angles @Sn1 in 1a-Ph: 274.0°), indicative of lone electron pairs at these element centres ††We note that the *para*-biphenyl stannylene, ^Cy^L(*p*BP)Sn:, was also synthesised, but only its crystal structure is reported here (Fig. S97 in SI)..

Our earlier reported syntheses of 16-electron germylene–Ni^0^ complexes were relatively straight forward, involving the addition of the germylene ligands to mixtures of Ni(cod)_2_ and the bulky NHC, IPr (IPr = [(H)CN(Dipp)]_2_C:).^42^ Presumably due to the increased Sn–Ni bond distance, relative to Ge–Ni, the selective formation of target mono-stannylene complexes was made more complex by the formation of bis(stannylene)-Ni^0^ complexes when using the described one-pot method from Ni(cod)_2_. For example, employing 1a-CF_3_ in this synthetic method led to the isolation of only small amounts of the target 16-electron complex 2-CF_3_ as a micro-crystalline powder, in addition to bis-stannylene complex 3 as the major product (Scheme 1, below), which we presume arises from the reaction of 2 equiv. 1a-CF_3_ with Ni(cod)_2_. Observation of ^31^P NMR spectra of crude reaction mixtures aligns with this product distribution (*i.e.*2-CF_3_: *δ* = 18.8 ppm; 3: *δ* = 25.4 ppm; Fig. S48), whilst the reaction of 1a-CF_3_ with 0.5 equiv. Ni(cod)_2_ leads exclusively to the formation of 3.

**Fig. 2:**
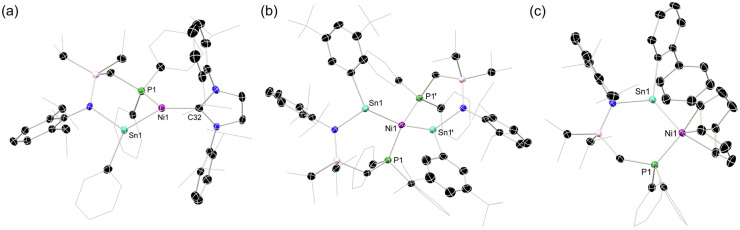
The molecular structures of (a) 2-Ph, (b) 3, and (c) 4, with thermal ellipsoids at 30% probability, and hydrogen atoms omitted for clarity. Selected bond lengths (Å) and angles (°) for 2-Ph: Ni1–Sn1 2.421(1); N1–Sn1 2.125(4); Ni1–P1 2.138(1); C32–Ni1 1.922(4); C32–Ni1–P1 126.8(1); C32–Ni1–Sn1 135.5(1); P1–Ni1–Sn1 97.09(4); C26–Sn1–N1 101.3(1); N1–Sn1–Ni1 111.78(9); C26–Sn1–Ni1 131.2(1). For 3: Sn1–Ni1 2.376(1); P1–Ni1 2.173(1); N1–Sn1 2.084(4); N1–Sn1–C32 101.4(1); Ni1–Sn1–C32 137.0(1); Ni1–Sn1–Ni1 120.84(1); P1–Ni1–Sn1 97.30(1); P1–Ni1–Sn1′ 108.87(1); Sn1–Ni1–Sn1′ 115.39(1); P1–Ni1–P1′ 130.15(1). For 4: Sn1–Ni1 2.3830(8); P1–Ni1 2.1665(8); C44–C45 1.391(5); C48–C49 1.366(5); Ni1–Sn1–N1 120.15(7); Ni1–Sn1–C32 130.28(7); N1–Sn1–C32 102.3(1).

**Fig. 3:**
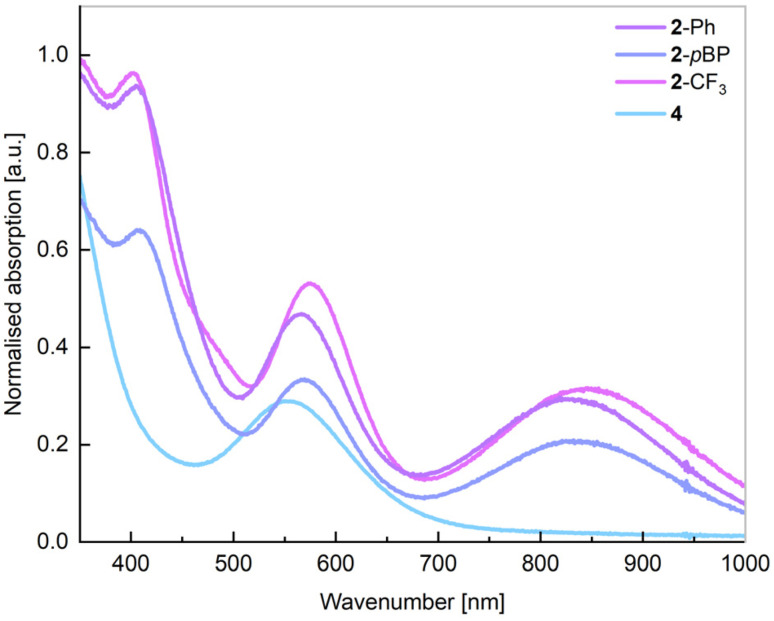
Normalised UV/vis spectra for complexes 2 and 4.

Characterisation of this species by single-crystal X-ray diffraction (Fig. 2(b)) reveals a distorted tetrahedral Ni centre, with symmetrical ligand environments; the two Sn–Ni–P angles in this species differ significantly (*i.e.* ∠Sn1–Ni1–P1 = 97.30(1)°; ∠Sn1– Ni1–P1′ = 108.87(1)°), with a more acute angle for the chelating P–Ni–Sn interaction. The Sn-centres are essentially trigonal planar (sum of angles @Sn = 359.24°). Moving to the more bulky ligand 1-*o*BP, in contrast to the formation of complexes 2 and 3, only the mono-stannylene Ni(cod) complex 4 is observed (Scheme 1, below; Fig. 2(c)). We presume that this is due to the sterically protrusive nature of the *o*-biphenyl substituent, precluding displacement of the second cod ligand at Ni, either by a further equivalent of 1a-*o*BP or the carbene, IPr. Whilst this is not conducive to the present study, this does demonstrate that straight-forward steric tuning in heavier tetrylene complexes allows for directed access to differing coordination complexes. This species bears resemblance to chelating bis(aryl)stannylene complexes reported by Wesemann;^47^ still, the 6-membered nature of the chelate in our systems leads to a near trigonal planar Sn centre showing a degree of pyramidalisation (sum of angles @Sn: 352.73°). Aside from arene-appended Ni^0^ derivatives, also reported by Wesemann, the Sn–Ni distance in 4 (2.3830(8) Å) is in line with reported examples. To avoid the above described synthetic shortfalls, *i.e.* the formation of bis(stannylene) systems 2, we looked towards the utility of IPr·Ni·(*η*^6^-toluene) as a stoichiometric source of [IPr·Ni].^48^ This gave essentially quantitative access to complexes 2 through direct combination with ^Ph^L-substituted stannylene ligands 1a, allowing for the isolation of analytically pure 2-Ph and 2-*p*BP, in addition to above described 2-CF_3_, in reasonable yields of 47–68% (Scheme 1, below). We note that attempts to make analogous complexes using the ^Cy^L system 1b-Ph failed, which we attribute the increase in donor strength for the Cy_2_P chelating arm. Two systems, *viz*. 2-Ph and 2-*p*BP, could be crystallographically characterized (Fig. 2(a)): both bear a trigonal planar Ni centre, but a pyramidalised Sn centre (sum of angles at Sn: 2-Ph = 344.34°; 2-*p*BP = 341.27°), suggesting a degree of acceptor character, and relatively weak Sn → Ni donor interactions, relative to our reported Ge^II^ systems. Wiberg Bond Index (WBI) and Mayer Bond Order (MBO) values of 0.737 and 1.023, respectively, indicate a single Sn–Ni σ-bond in 2-Ph (Table S1 in SI). Additionally, a Natural Bond Orbital (NBO) analysis of 2-Ph reveals the presence of a polarized bond (19.42% Ni *vs.* 80.58% Sn) overall suggesting Sn → Ni donor–acceptor interaction (Table S6 in SI). Observed Sn–Ni distances are surprisingly longer than in cod-complex 4 (4: 2.3830(8) Å; 2-Ph: 2.421(1) Å; 2-*p*BP: 2.425(1) Å), despite the lower coordinate Ni centre, which one may assume allows for increased bonding interactions between these two metal centres. It is feasible that the increased bond length is due to the steric bulk of the IPr ligand now at Ni. All systems contain a singlet resonance in their ^31^P{^1^H} NMR spectra between *δ* = 16 and 25 ppm, whilst the ^119^Sn{^1^H} NMR spectrum for 2-Ph displays a doublet at 688 ppm (^2^*J*_SnP_ = 698 Hz). The UV/vis spectra for all 16-electron Ni^0^ systems demonstrate two key absorptions in the visible region, one significantly red-shifted (2-Ph: 818 nm; 2-*p*BP: 832 nm; 2-CF_3_: 846 nm), in addition to one centred around ∼575 nm (Fig. 3). The former relates to the HOMO–LUMO (Highest Occupied Molecular Orbital; Lowest Occupied Molecular Orbital) separation in these systems. For example, the Ge derivative of 2-Ph demonstrates a red-shifted *λ*_max_ value of 752 nm, and a DFT derived Δ*E*_HOMO/LUMO_ of 1.62 eV.^42^ In the current case, calculations at the BP86-D3(SMD = Benzene)/def2-TZVP level of theory find a Δ*E*_HOMO/LUMO_ for 2-Ph of 1.43 eV (see Fig S102 and S103 in SI). Shedding further light on this, time-dependent density functional theory (TD-DFT) calculations show a HOMO–LUMO excitation for 2-Ph at 762 nm, close to the experimental value at 818 nm, solidifying UV/vis analyses as a good determinant of Δ*E*_HOMO/LUMO_ in these systems. Such a significant red-shift would typically be indicative of a highly reactive system. Notably, the cod-bound 18-electron complex 4 does not demonstrate such a red-shifted absorption (Fig. 3; calculated value: 629 nm), indicating a significant electronic difference between these complex classes.

### Dihydrogen activation in stannylene-Ni^0^ systems

**Fig. 4:**
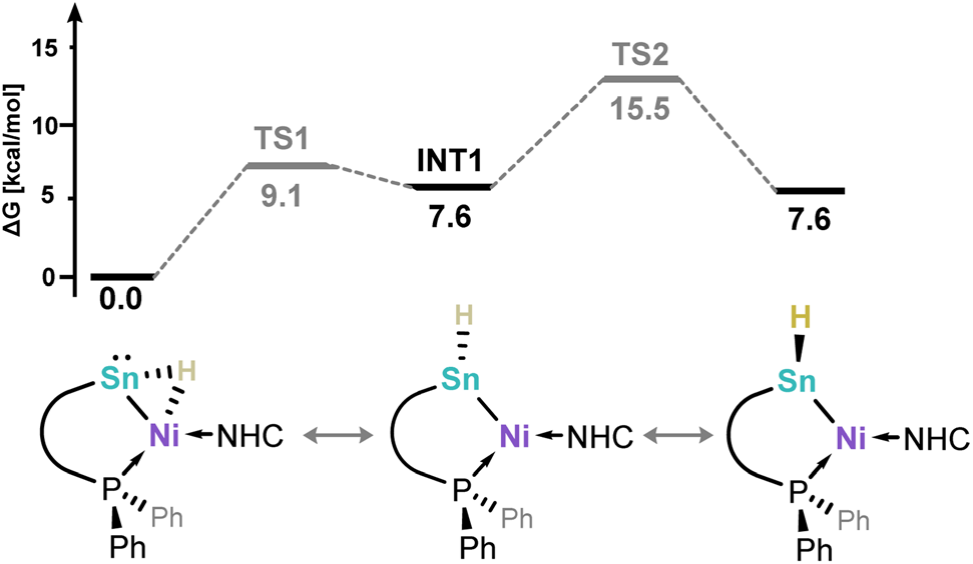
DFT-derived Gibbs free energy profile for the tautomerisation of bridge Sn-(µ-H)-Ni hydride complex 5 with its terminal Sn–H derivative.

A small number of known tetrylene-transition metal complexes have demonstrated the capacity to activate dihydrogen, in some cases reversibly,^38,39,42^ but is currently unknown for stannylene complexes. We thus aimed to probe the effect of the Sn^II^ centre on the dihydrogen activation process. In an initial experiment, a C_6_D_6_ solution of complex 2-Ph was treated with a 1 bar H_2_ atmosphere in a Teflon-sealed NMR tube. After 16 h, complete conversion to a single product, *viz.*5, is observed (^31^P NMR: *δ* = −14.8 ppm). This ^31^P NMR shift is significantly up-field when compared with the starting material (*δ* = 19.5 ppm), and indeed quite different to our earlier reported Ge systems (*δ* = ∼9 ppm). In addition, discernible high- or low-field resonances pertaining to terminal Sn–*H* or Ni–H moieties could not be observed in ^1^H NMR spectra of these mixtures, even at lower temperatures of −60 °C (*i.e.* in *d*_8_-toluene Fig. S86–S88). We thus moved to 2-CF_3_ and 2-*p*BP to gain further insights. We were surprised to find that identical ^31^P NMR resonances are observed upon treatment of these systems with 1.5 bar H_2_, which leads us to logically conclude that, remarkably, the activation of H_2_ leads to Sn–Ar scission for all species. This was confirmed for 2-CF_3_: following the reaction of this species with either H_2_ or D_2_ for 16 h, ^19^F NMR spectra show the clean formation of *e.g. m*-(CF_3_)_2_–C_6_H_3_D through comparison with a commercial sample (Fig. S88 in SI). In addition, these mixtures were quenched with D_2_O (for H_2_) or H_2_O (for D_2_) - GC-MS analyses of these mixtures indicated the formation of *m*-(CF_3_)_2_–C_6_H_4_ (for H_2_) or *m*-(CF_3_)_2_–C_6_H_3_D (for D_2_) (Fig. S89 and S90). As for the initial study involving 2-Ph, ^1^H NMR spectra for these reaction mixtures do not reveal a clear high- or low-shifted hydride resonance, and we have not yet been able to crystallise the formed complex due to further reactivity (*vide supra*). Still, the presence of a hydride ligand is clearly demonstrated by the analogous reactions with D_2_, in forming 5-D, which bears a 1 : 1 : 1 triplet signal in ^31^P NMR spectra centred at *δ* = −14.8 ppm, again for all systems (Fig. S80 in SI). We assign the coupling value of 5.9 Hz as a ^2^*J*_PD_ value, given the reduced gyromagnetic ratio of ^2^D relative to ^1^H. Comparing the ^1^H NMR spectra for reaction mixtures generated from H_2_ and D_2_ now allows for the determination of a resonance attributable to a Sn/Ni–H: a doublet centred at *δ* = 2.13 ppm for the H_2_ reaction, but absent in the D_2_ reaction (Fig. S91). A ^2^*J*_PH_ coupling of 39.9 Hz aligns with the corresponding ^2^*J*_PD_ observed for 5-D, when correcting with the relative magnitude of gyro magnetic ratios (*i.e.* H : D, 6.5 : 1). Evidence for the formation of a mono-hydride complex was gained through reaction of our reported hydrido-stannylene, ^Ph^LSnH,^49^ with IPr·Ni·(*η*^6^-toluene), leading to ^1^H and ^31^P NMR spectral data identical to those observed in the above described reactions between 2 and H_2_, *i.e.* forming 5 (Fig. S92). This species does not react with dihydrogen. This collection of data, then, would suggest that the reactions between aryl-stannylene-Ni^0^ complexes 3 and dihydrogen lead to a mono-hydrido stannylene-Ni^0^ complex, also in keeping with LIFDI-MS data for these reaction mixtures (Fig. S87). To shed light on the relative energy of an expected terminal Sn–H complex and the apparent bridging Sn-(µ-H)-Ni hydride complex 5, DFT calculations were carried out (Fig. 4). We find that the bridged hydride tautomer is 7.6 kcal mol^−1^ lower in energy than the terminal hydride derivative, albeit with energetic barriers expected to be accessible at ambient temperature. This aligns with the discussed NMR spectra, whereby a terminal Sn–H or Ni–H would be typically appear at highly low- or high-field shifts, respectively.^50–53^

**Scheme 2:**
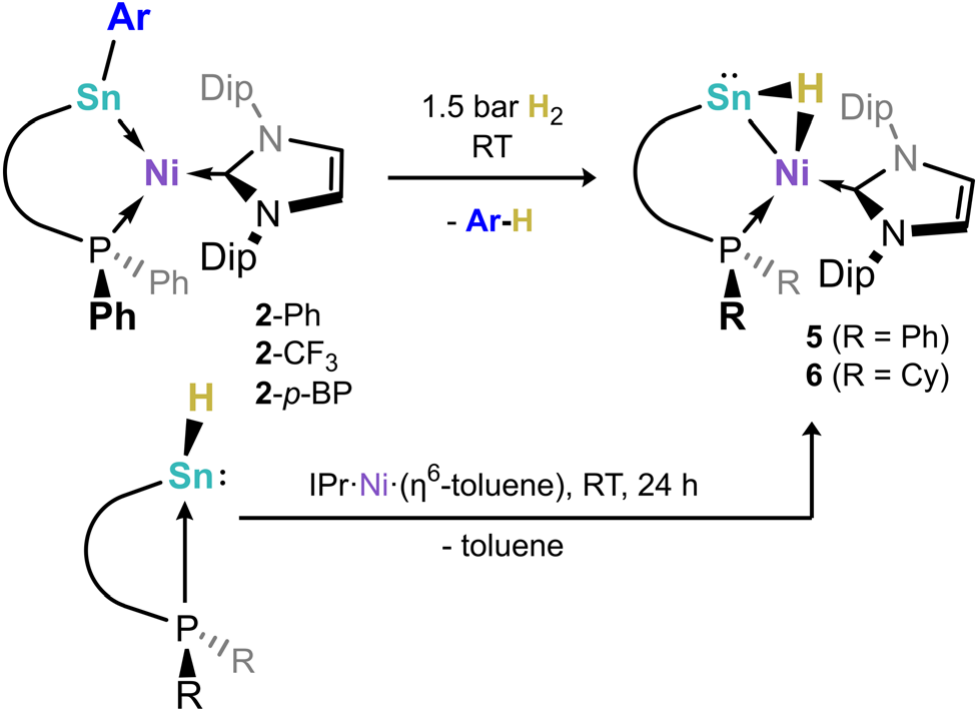
The scission of Sn–C^Ar^ bonds upon H_2_ activation by compounds 2, and an alternative synthetic pathway to the so formed hydride complexes 5 and 6.

**Fig. 5:**
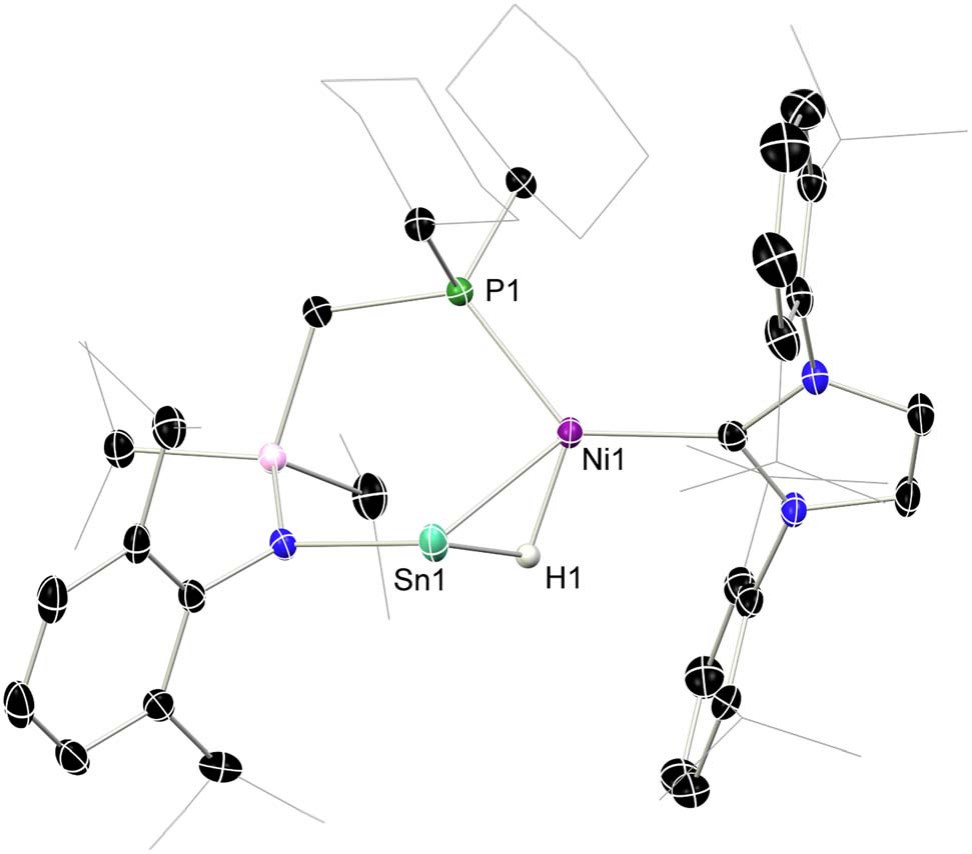
The molecular structure of 6, with thermal ellipsoids at 40% probability, and hydrogen atoms omitted for clarity. Selected bond lengths (Å) and angles (°): Ni–Sn 2.4303(7); P1–Ni1 2.1589(8); Sn1–H1 1.83(3); Ni1–H1 1.57(3); N1–Sn1 2.138(2); P1–Ni1–C32 128.81(6); Sn1–Ni1–P1 92.79(2); Sn1–Ni1–C32 133.63(6); Ni1–Sn1–N1 107.98(5).

In order to gain structural evidence for this unique species, and particularly to elucidate the nature of the hydride ligand, we synthesized the novel hydrido stannylene ^Cy^L(H)Sn: (1b-H), which we hoped would aid crystallization. As per the analogous ^Ph^L(H)Sn:, the novel hydrido-stannylene readily reacts with IPr·Ni·(*η*^6^-toluene) in the formation of a single new species, *viz*. 6 (Scheme 2). Notably, ^1^H NMR spectra demonstrate the absence of a low-field shifted Sn–H resonance, present at *δ* = 10.78 ppm for the free stannylene ligand. Large crystals grown from concentrated pentane solutions allowed for the acquisition of structural data for 6, revealing the target hydrido-stannylene Ni^0^ complex 6 (Fig. 5). The hydride ligand could be located and freely refined, found to bridge the Sn and Ni centres, confirming our hypothesis based on NMR spectroscopy and DFT studies. The Sn–Ni bond length is similar to those in (amido)(aryl)stannylene complexes 2 (2.4303(7) Å) despite the significant difference in geometry at Sn, which negates any significant Sn → Ni σ-donation (*vide infra*). This is perhaps better borne out by a more acute N–Sn–Ni angle of 107.98(5)°, some 5° contracted relative to those in *e.g.*2-Ph, and likely indicative a greater electron density residing on the Sn centre. The ^1^H NMR spectrum of dissolved crystals of 6 reveals a doublet at *δ* = 3.33 ppm (^2^*J*_HP_ = 26.8 Hz) attributable to a Sn-(µ-*H*)-Ni ligand, and so indirectly confirming the bridging nature of the hydride ligand in the analogous complex 5. A significantly broadened signal is observed in the ^119^Sn{^1^H} NMR spectrum of this sample, centred at *δ* = 1220 ppm. Notably, no such signal can be located in corresponding spectra for 5, likely indicative of rapid hydride exchange in solution. DFT calculations on the nature of the Sn–Ni interaction in complex 5 shows an elongated Sn–Ni bond length of 2.509 Å relative to complex 2-Ph (2.408 Å), again indicating a weaker Sn–Ni bonding interaction due to the presence of the bridging hydrogen (see Tables S3 and S5 in SI). Calculated WBI and MBO values for this bond of 0.501 and 0.576, respectively, are lower than those in 2-Ph (WBI: 0.737; MBO: 1.023), and indicate partial bonding between the Sn and Ni centres. This is further confirmed by the absence well-defined Sn–Ni bonding orbitals in the NBO analysis. In this regard, the calculated HOMO of 5 represents a Sn-centred lone electron pair, further confirming the absence of a Sn → Ni donor interaction. Additionally, NBO analyses indicate that the Ni centre in 5 bears a Ni–H bond having a significant electron occupation of 1.62 e^−^ and a significant polarization towards hydrogen (28.84% Ni/71.16% H; see Table S8 in SI), suggestive of a covalent Ni–H bond. As such, complexes 5 and 6 may be described either as a formal (hydrido)nickelo-stannylene species, representing the first examples of group 10-derived metallotetrylenes, or agostic [RSn–H–Ni] bonded (hydrido-stannylene)-Ni^0^ complexes.

**Fig. 6:**
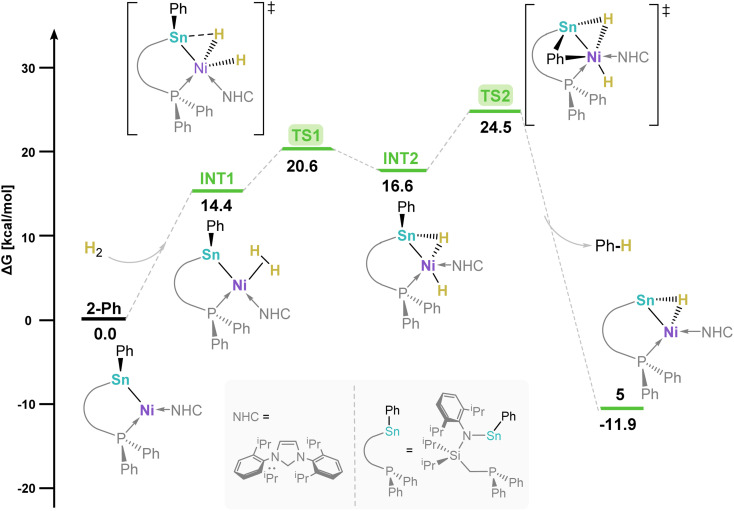
DFT-derived Gibbs free energy reaction profile for H_2_ activation of complex 2-Ph. TS and INT denote transition state and intermediates, respectively.

The novel process leading to 5 warrants greater exploration. The DFT derived reaction mechanism for H_2_ activation in 2-Ph leading to complex 5 (Fig. 6) suggests that the initial coordination of H_2_ proceeds *via* a barrierless step at the Ni centre (INT1, 14.4 kcal mol^−1^). The following H–H scission process involves Sn-coordination, with a barrier of 20.6 kcal mol^−1^ (TS1), in forming 1,2-dihydride species INT2 (16.6 kcal mol^−1^); this suggests a cooperative pathway to H_2_ cleavage. From INT2, formal C–H reductive elimination of benzene *via* TS2 (24.5 kcal mol^−1^) is strongly favoured, in forming complex 5 with a global exergonic value of −11.9 kcal mol^−1^. Although the rate limiting step for this overall mechanism is benzene elimination (*i.e.* 24.5 kcal mol^−1^ relative to 2-Ph), the above-described exergonic value clearly drives the reaction. Nevertheless, the long reaction times (*i.e.* 16 h) align with this high energetic barrier.

### Formation of a metallo-bis(stannylene) complex

**Fig. 7:**
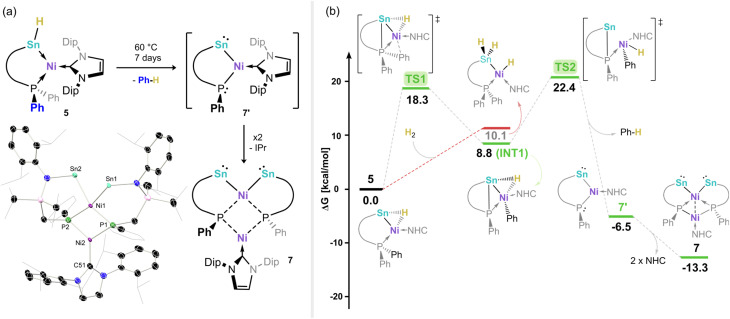
(a) Synthesis of nickel-bis(stannylene) complex 7. Inset: the molecular structure of 7, with thermal ellipsoids at 30% probability, and hydrogen atoms removed for clarity, and (b) the DFT-derived Gibbs free energy reaction profile for the disfavoured H_2_ activation by 5 (red), and the formal reductive elimination of benzene from 5 in forming 7′ and 7. Selected bond lengths (Å) and angles (°) for 7: Sn1⋯Sn2 3.464(2); Sn1–Ni1 2.538(3); Sn2–Ni1 2.532(4); Ni1–Ni2 2.433(5); N1–Sn1 2.096(5); N2–Sn2 2.151(5); N1–Sn1–Ni1 107.0(2); N2–Sn2–Ni1 101.2(1); Sn1–Ni1–Sn2 86.2(1); Sn1–Ni1–P1 80.0(1); Sn2–Ni1–P2 85.1(1).

Prolonged storage of NMR samples of 5 led to the gradual appearance of a single new species with a low-field ^31^P NMR shift of *δ* = 87.4 ppm. Such a drastic shift from that for 5 (*i.e.* −14.8 ppm) suggests a significant change in the phosphorous environment. Heating pure samples of 5, generated *in situ* from the reaction between 2-Ph and H_2_, for 7 days at 60 °C leads to the clean formation of the same species, as ascertained by ^31^P NMR spectra of reaction mixtures. No signals are observed in ^119^Sn NMR spectra of this species, perhaps indicative of complex heteronuclear coupling. Crystallisation of these mixtures allowed for the isolation of large purple crystals, revealing the formation of the unique nickelo-bis(stannylene) complex 7 (Fig. 7(a)). We hypothesise that this species forms through the elimination of Ph-H (*i.e.* benzene), arising from one Ph-group of the phosphine arm and the Ni–H ligand in 5. This would initially form intermediate 7′, a phosphido-nickelostannylene, which can dimerise through loss of IPr in forming 7 (*vide infra*). Notably, the molecular ion peak for 7′ is observed in LIFDI-MS spectra of solutions of 5 (Fig. S87), giving circumstantial evidence for the formation of this monomer.

The molecular structure of 7 reveals a tetrametallic core, with two formal metallo-stannylene centres and two phosphide centres binding the two nickel centres. Given the greater electronegativity of P and Sn *vs.* Ni,^54,55^ this complex contains two Ni^2+^ centres, and two formally dianionic chelating [Sn–PPh]^2−^ ligands, with one [Sn_2_NiP_2_] site, and a second [P_2_Ni·IPr] site. The former sits in a slightly distorted square planar geometry (sum of angles @Ni1: 362.96°), aligning with a *d*_8_ Ni^II^ centre. The second lies in a trigonal planar geometry (sum of angles @Ni2: 359.6°). A long Sn⋯Sn distance of 3.491(2) Å negates any bonding interaction between these centres, whereby such large Sn–Sn bond distances are only found in Zintyl clusters of tin.^56,57^ As such, compound 7 represents an additional novel example of a metallo-tetrylene incorporating a group 10 metal as the metallo-ligand, and the first with distinct divalent Sn centres. The DFT-derived frontier orbitals of 7 are representative of the stannylene-character this species, borne out by the presence of a high s-character lone electron pair at each Sn centre as the HOMO (Table S12 and Fig. S101). Notably, the LUMO represents the in-phase combination of the corresponding two vacant p-orbitals, which, through an end-on interaction, form a vacant bonding orbital of σ-symmetry (Fig. S100).

Given the novel route by which 7 is formed, and our hypothesis that an intermediary monomeric species (*viz.*7′) is formed initially on heating 5, additional DFT calculations were carried out to define the mechanism of this process (Fig. 7(b)). We find that this reaction proceeds *via* the transfer of P-coordinated Ph to Ni over a barrier of 18.3 kcal mol^−1^, leading to the formation of INT1 (8.8 kcal mol^−1^) with a newly formed Sn–P bond. Subsequently, the bridging Sn–Ni hydride present in INT1 is cleaved and transferred to the Ph-ligand, representing a formal C–H bond reductive elimination (TS2, 22.4 kcal mol^−1^), yielding hypothesized complex 7′, with an overall exergonic reaction coordinate of −6.5 kcal mol^−1^. This species can then dimerize in the elimination of IPr, forming the experimentally observed complex 7, with an additional energy gain of 6.8 kcal mol^−1^. This calculated mechanism gives further key insights into an additional C–H bond formation process which involves multiple heavier main group elements, which we aim to utilise in later synthetic protocols.

## Conclusions

We have described the development of a novel family of chelating (amido)(aryl)stannylene ligands (*viz*. 1), and a selection of their Ni^0^ complexes. Depending on ligand bulk, 16-electron Ni(NHC)- or 18-electron Ni(cod)-complexes are accessed, giving insights into steric control over complex formation for this unique ligand class. We demonstrate the facile activation of dihydrogen in isolated 16-electron systems: this leads to an initial, intermediary 1,2-dihydride complex, shown by computational modelling. However, in contrast to known systems which achieve similar cooperative H_2_ activation, these aryl-stannylene systems undergo the facile elimination of the Ar–H fragments through 1,2-ligand exchange, in generating a H-bridged hydrido-stannylene complex, being the first example of such a species. We demonstrate that further heating of this complex leads to an additional Ar–H elimination, cleaving one P–C bond of the ligand's chelating arm. This ultimately leads to a unique example of a nickelo-bis(stannylene). These results give unique insights regarding the diverse chemistry possible at the stannylene–nickel interface, particularly driven by reductive elimination processes – typically challenging for Ni. We continue to explore this reactivity in the context of cooperative catalysis.

## Author contributions

JG and PMK carried out all experimental and analytical work. MI carried out all computational work. TS supervised the computational aspect of the work. TJH supervised the experimental aspects of this work, and devised the study.

## Conflicts of interest

There are no conflicts of interest to declare.

## Supplementary Material

SC-017-D5SC09496H-s001

SC-017-D5SC09496H-s002

## Data Availability

CCDC 2492619–2492628 (1a-Ph, 1a-CF_3_, 1b-Ph, 1b-pBP, 2-Ph, 2-pBP, 3-CF_3_, 4, 6, and 7) contain the supplementary crystallographic data for this paper.^58*a*–*j*^ The data supporting this article have been included as part of the supplementary information (SI). Supplementary information is available. See DOI: https://doi.org/10.1039/d5sc09496h.
