# Supplementary material for: Cooperative metathesis of H–H/Sn–CAr bonds in stannylene-Ni0 systems
Source: Chem Sci. 2026 Feb 25;17(16):7938–46. doi: 10.1039/d5sc09496h (PMC12962257; doi:10.1039/d5sc09496h)
Supplement: SC-017-D5SC09496H-s001 [file SC-017-D5SC09496H-s001.pdf]

Supplementary Information (SI) for Chemical Science.
This journal is © The Royal Society of Chemistry 2026

Supporting Information

From Sn^{II} to Sn^0 *via* Cooperative Metathesis of H-H/Sn-C^{Ar} bonds in stannylene-Ni^0 systems

Jonas M. Gilch,[a] M. Iddrisu,[b] Philip M. Keil,[a] Tibor Szilvási,[b,*] and Terrance J. Hadlington[a,*]

[a] Lehrstuhl für anorganische Chemie mit Schwerpunkt neue Materialien, Technische Universität München, Lichtenbergstraße 4, 85747 Garching, Germany

[b] Department of Chemical and Biological Engineering, University of Alabama, Tuscaloosa, AL 35487, United States.

Table of Contents

1. General Considerations 2
2. Synthetic details and data 3
   NMR, MS, UV/vis spectra of the synthesized compounds 20
3. Analysis of reactions leading to ^{Ph}L(H)Sn·Ni·IPr] 4 62
4. X-ray crystallographic details 72
5. Computational methods and details 76
6. References 132

1. General Considerations

All experiments and manipulations were carried out under dry oxygen free argon atmosphere using standard Schlenk techniques or in a MBraun UNIlab inert atmosphere glovebox containing an atmosphere of high purity argon 5.0. THF and Et_2O were dried over Na/Benzophenone, distilled and stored over 4Å molecular sieves. All other solvents were dried over activated 4Å molecular sieves and thoroughly degassed before use. C_6D_6 and toluene-d_8 were dried, degassed and stored over a potassium mirror. Ni(cod)$_2$,[1] IPr,[2] IPr·Ni·(η^6-toluene),[3] ^{Ph}LK,[4] ^{Ph}L(Cl)Sn:,[5] ^{Ph}L(H)Sn:,[6] and ^{Cy}LK [7] were synthesized using reported procedures.

NMR-Spectroscopy. NMR spectra were recorded on a Bruker AV 400 Spectrometer. The spectra were processed using the MestReNova 14.3.3 software suite. All chemical shifts δ are given in ppm. The ^{1}H, and ^{13}C{^{1}H} NMR spectra were referenced to the residual solvent signals as internal standards. ^{29}Si{^{1}H} NMR spectra were externally calibrated with $SiMe_4$. ^{31}P{^{1}H} and ^{31}P NMR spectra were externally calibrated with H_3PO_4. ^{119}Sn NMR spectra were externally referenced with $SnMe_4$. The coupling constants J are given in Hz. For variable temperature NMR experiments with a targeted temperature of over 298 K, the temperature inside the probe head was externally referenced via monitoring the OH/CH_2 peak separation in an 80 wt% glycol/DMSO-d_6 solution. For variable temperature NMR experiments with a targeted temperature of under 298 K, the temperature inside the probe head was externally referenced via monitoring the OH/CH_3 peak separation in a 4 wt% methanol/methanol-d_4 solution. For signal multiplicities, the following abbreviations were used: s = singlet, d = doublet, t = triplet, q = quartet, p = pentet, h = heptet, sept= septet, m = multiplet, br = broad and their combinations.

LIFDI-MS. Liquid Injection Field Desorption Ionization Mass Spectrometry (LIFDI-MS) was measured with a Thermo Fisher Scientific Exactive Plus Orbitrap equipped with an ion source from Linden CMS. Sample preparation and injection was performed in a MBraun glovebox under argon atmosphere.[8]

GC-MS. GC-MS was measured with an Agilent Technologies 7890B GC featuring an MS 5977A single quadrupole mass detector. Sample separation is obtained *via* a capillary column HP-5MS UI (length 30 m, inside diameter 0.25 mm, film 0.25 µm, stationary phase

(5%-phenyl)-methylpolysiloxane. Masses and isotope patterns were calculated with enviPat Web 2.4.

IR-Spectroscopy. Infrared spectra were measured with the Alpha FT-IR from Bruker containing a platinum diamond ATR device. The compounds were measured as solids in an MBraun Labmaster dp inert atmosphere glovebox containing a dry oxygen free atmosphere of high purity argon 5.0.

UV-Vis-Spectroscopy. Absorption spectra (UV-Vis) were measured in an inert MBraun UNIlab glovebox using a mirror probe connected to an Agilent Cary 60 UV/Vis spectrophotometer *via* fiberglass cable.

Elemental Analysis. Elemental analyses (C, H, N) were performed with a combustion analyzer (elementar vario EL, Bruker).

2. Synthetic details and data

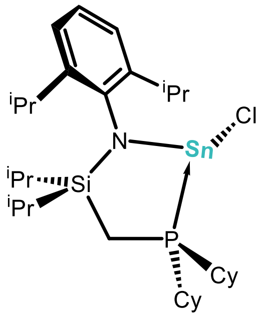

^{Cy}L(Cl)Sn:. The stannylene was synthesized by dropwise adding ^{Cy}LK (4.00 g, 7.41 mmol, 1.00 eq.) in THF (10.0 mL) to a cooled (- 80 °C) suspension of $SnCl_2$ (1.40 g, 7.41 mmol, 1.00 eq.) in THF (5.00 mL). After stirring in the cold for 1 h, the mixture was stirred at ambient temperature for an additional 2 h. The solvent was removed *in vacuo* and the remains extracted with toluene (15.0 mL). The dried extract was washed with pentane (2 x 10.0 mL) to obtain an off-white solid (3.30 g, 5.04 mmol, 68 %).

^{1}H NMR (C_6D_6, 400 MHz, 298 K): δ = 7.21-7.23 (m, 1H, Ar-*H*), 7.09-7.13 (m, 2H, Ar-*H*), 4.31 (sept, $^{3}J_{HH}$ = 6.9 Hz, 1H, L-iPr-*CH*), 3.48 (sept, $^{3}J_{HH}$ = 6.9 Hz, 1H, L-iPr-*CH*), 2.31 (m, 1H, P-Cy-*H*), 1.67-1.93 (m, 5H Cy-*H*), 1.58-1.67 (m, 6H, Cy-*H* + Si-C*H*$_2$-P), 1.47-1.58 (m, 7H, Si-iPr-*CH$_3$*+ Si-iPr-*CH*), 1.44 (virt dd, $^{3}J_{HH}$ = 6.8 Hz, J = 2.4 Hz, 6H, L-iPr-*CH$_3$*), 1.36 (d, $^{3}J_{HH}$ = 6.9 Hz, 3H, L-iPr-*CH$_3$*), 1.19-1.24 (m, 6H, L-iPr-*CH$_3$* + Si-iPr-*CH$_3$*), 0.88-1.19 (m, 14H, Cy-*H* + Si-iPr-*CH*), 0.72 (d, $^{3}J_{HH}$ = 6.8 Hz, 3H, Si-iPr-*CH$_3$*).

^{13}C{^{1}H} NMR (C_6D_6, 101 MHz, 298 K): δ = 148.7 (s, L-^{ipso}*C*), 147.6 (s, L-^{ortho}*C*H), 144.9 (d, $^{3}J_{PC}$ = 7.2 Hz, L-^{ortho}*C*H), 144.9 (s, L-^{ortho}*C*H), 124.2 (s, L-^{meta}*C*H), 124.0 (s, L-^{meta}*C*H), 123.7 (s, L-^{para}*C*H), 35.2 (d, J_{PC} = 12.6 Hz, Cy-*C*), 34.2 (d, J_{PC} = 6.3 Hz, Cy-*C*), 31.2 (d, J_{PC} = 3.4

Hz, Cy-*C*), 30.6 (s, Cy-*C*), 30.1 (s, Si-iPr-*C*), 29.3 (d, J_{PC} = 4.0 Hz, Cy-*C*), 28.8 (s, L-iPr-*C*H_3), 28.7 (s, L-iPr-*C*H_3), 28.3 (s, L-iPr-*C*H_3), 27.9 (s), 27.8 (s), 27.6 (s), 27.6 (s), 27.5 (s), 27.5 (s), 27.5 (s), 27.4 (s), 27.3 (s, L-iPr-*C*H), 26.2 (d, J_{PC} = 6.6 Hz, Cy-*H*), 23.7 (s, L-iPr-*C*H_3), 23.1 (s, L-iPr-*C*H_3), 20.6 (s, Si-iPr-*C*H_3), 20.1 (s, Si-iPr-*C*H_3), 20.0 (s, Si-iPr-*C*H_3), 18.1 (s, Si-iPr-*C*H_3), 17.9 (d, J_{PC} = 3.7 Hz, Si-iPr-*C*H), 15.8 (s, J_{PC} = 2.2 Hz, Si-iPr-*C*H), 0.1 (d, $^1J_{PC}$ = 6.3 Hz, Si-*C*H_2-P).

^{29}Si{^{1}H} NMR (C_6D_6, 99 MHz, 298 K): δ = 13.8 (d, $^2J_{Si,P}$ = 6.3 Hz, CH_2-*Si*-(^{i}Pr)$_2$).

^{31}P{^{1}H} NMR (C_6D_6, 162 MHz, 298 K): δ = 18.8 (s, CH_2-*P*-(Cy)$_2$, $^1J_{117SnP}$ = 1480 Hz, $^1J_{119SnP}$ = 1550 Hz).

MS/LIFDI-HRMS found (calcd.) m/z: 655.2596 (655.2550) for [^{Cy}L(Cl)Sn:]$^+$.

Anal. calcd. for $C_{31}H_{55}ClNPSiSn$: C, 56.85%; H, 8.46%; N, 2.14%. **found**: C, 56.52 %; H, 7.93 %; N, 2.12 %.

^{Cy}L(H)Sn:, 1b-H. To a solution of ^{Cy}L(Cl)Sn: (2.75 g, 4.20 mmol, 1.00 eq.) dissolved in THF (35 mL), cooled to -80 °C, a solution of K-Selectride (1.00 M in THF, 4.20 mL, 4.20 mmol, 1.00 eq.) was slowly added (*i.e.* over the course of 5 min). After stirring for 3h all volatile components were removed *in* vacuo. The remains were extracted in toluene. After removing the solvent an off-white powder identified as ^{Cy}L(H)Sn (1.47 g, 56 %).

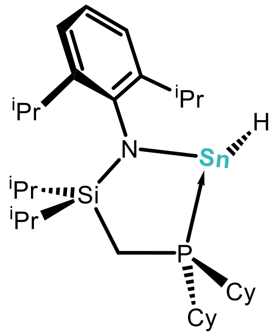

^{1}H NMR (C_6D_6, 400 MHz, 298 K): δ = 10.79 (d, 1H, Sn-*H*, $^2J_{PH}$ = 4 Hz, $^1J_{SnH}$ = 24 Hz), 7.19 (m, 2H, L-*mCH*), 7.13 (m, 1H, L-*pCH*), 4.18 (sept, $^3J_{HH}$ = 6.9 Hz, 1H, L-iPr-*CH*), 3.58 (sept, $^3J_{HH}$ = 6.9 Hz, 1H, L-iPr-*CH*), 2.00 (m, 1H, Cy-*H*), 1.93 (m, 1H, Cy-*H*), 1.81 (m, 2H, Cy-*H*), 1.73 (dd, $^2J_{HH}$ = 6.9 Hz, $^2J_{HP}$ = 14.9 Hz, 1H, Si-*C*H_2-P), 1.70 (dd, $^2J_{HH}$ = 6.9 Hz, $^2J_{HP}$ = 14.9 Hz, 1H, Si-*C*H_2-P), 1.66 (m, 2H, Cy-*H*), 1.58 (m, 2H, Cy-*H*), 1.51 (m, 2H, Cy-*H*), 1.44 (d, $^3J_{HH}$ = 6.9 Hz, 6H, L-iPr-*C*H_3), 1.43 (d, $^3J_{HH}$ = 6.9 Hz, 6H, L-iPr-*C*H_3), 1.41 (d, $^3J_{HH}$ = 8.5 Hz, 3H, Si-iPr-*C*H_3), 1.29 (m, 2H, Cy-*H*), 1.22 (d, $^3J_{HH}$ = 7.3 Hz, 6H, Si-iPr-*C*H_3), 1.17 (m, 4H, Cy-*H*), 1.13 (m, 2H, Si-iPr-*CH*), 1.03 (m, 6H, Cy-*H*), 0.80 (d, $^3J_{HH}$ = 6.6 Hz, 3H, Si-iPr-*C*H_3).

^{13}C{^{1}H} NMR (C_6D_6, 101 MHz, 298 K): δ = 148.3 (d, $^4J_{PC}$ = 5.3 Hz, L-^{ipso}*C*), 147.6 (d, $^5J_{PC}$ = 1.6 Hz, L-^{ortho}*C*H), 144.9 (s, L-^{ortho}*C*H), 144.9 (s, L-^{ortho}*C*H), 123.9 (s, L-^{meta}*C*H), 123.6 (s, L-^{meta}*C*H), 122.9 (s, L-^{para}*C*H), 36.9 (d, J_{PC} = 7.0 Hz, Cy-*C*), 34.4 (d, J_{PC} = 11.5 Hz, Cy-*C*), 30.5 (d, J_{PC} = 3.2 Hz, Cy-*C*), 30.4 (s, Cy-*C*), 30.1 (d, J_{PC} = 31.9 Hz, Cy-*C*), 29.6 (s, Cy-*C*), 29.6 (s, Cy-*C*), 27.7 (s), 27.7 (s, L-iPr-*C*H_3), 27.6 (s), 27.6 (s), 27.6 (s), 27.5 (s), 27.5 (s), 27.4 (s),

27.3 (d, $^{3}J_{PC}$ = 2.6 Hz, Cy-*C*), 26.2 (d, $^{2}J_{PC}$ = 6.4 Hz, Cy-*C*), 23.9 (s, L-iPr-*C*H), 23.5 (s, L-iPr-*C*H), 20.9 (s, Si-iPr-*C*H_3), 20.7 (s, Si-iPr-*C*H_3), 20.0 (s, Si-iPr-*C*H_3), 18.3 (s, Si-iPr-*C*H_3), 17.0 (d, $^{3}J_{PC}$ = 6.2 Hz, Si-iPr-*C*H_3), 16.2 (s, Si-iPr-*C*H_3), 3.7 (d, $^{1}J_{PC}$ = 6.7 Hz, Si-*C*H_2-P).

^{29}Si{^{1}H} NMR (C_6D_6, 99 MHz, 298 K): δ = 6.95 (d, $^{2}J_{Si,P}$ = 6.4 Hz, CH_2-*Si*-(^{i}Pr)$_2$).

^{31}P{^{1}H} NMR (C_6D_6, 162 MHz, 298 K): δ = 15.5 (s, CH_2-*P*-(Cy)$_2$, $^{1}J_{117SnP}$ = 1258 Hz, $^{1}J_{119SnP}$ = 1316 Hz).

MS/LIFDI-HRMS found (calcd.) m/z: 620.2874 (620.2870) for [^{Cy}LSn]$^{+}$.

Anal. calcd. for $C_{31}H_{56}NPSiSn$: C, 60.00%; H, 9.10%; N, 2.26%. **found**: C, 59.70 %; H, 9.38 %; N, 2.19 %.

General method for the preparation of ^{Ph}L(R)Sn:, 1.

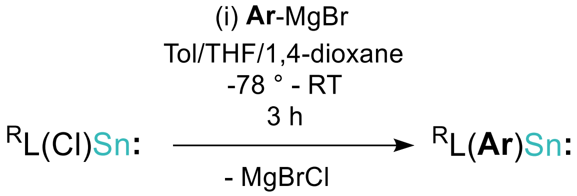

A solution of freshly prepared aryl-Grignard reagent in THF (1.00 eq.) was filtered dropwise onto ^{Ph}L(Cl)Sn: (1.00 eq.) and 1,4-dioxane (4.00 eq.) dissolved in toluene at -78 °C and stirred for 30 min. The mixture was then allowed to warm to ambient temperature and stirred for additional 2 h. Subsequently, all volatiles were removed *in vacuo* and the residue was extracted with toluene. The solution can be used in subsequent reactions without further purification.

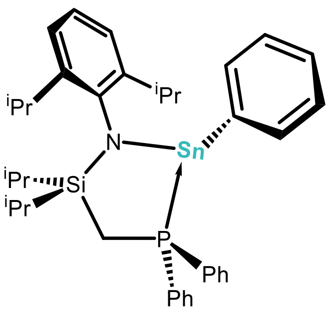

^{Ph}L(Ph)Sn:, 1a-Ph. The stannylene was synthesized according to the general method using ^{Ph}L(Cl)Sn: (0.66 g, 1.02 mmol, 1.00 eq.), 1,4-dioxane (0.70 mL, 4.09 mmol, 4.00 eq.), and a freshly prepared PhMgBr in THF using PhBr (0.16 g, 1.02 mmol, 1.00 eq.), Mg (0.37 g, 1.54 mmol, 1.50 eq), and a crystal of iodide. The toluene solution was used without further purification assuming full conversion, backed up by NMR. Separately, crystalline compound for further analysis were obtained from a concentrated pentane solution at ambient temperature after 1 day.

^{1}H NMR (C_6D_6, 400 MHz, 298 K): δ = 7.70 (m, 4H, Ar-*H*), 7.23 (t, $^1J_{HH}$ = 7.1 Hz, 2H, Ar-*H*), 7.13 (m, 7H, Ar-*H*), 6.86 (m, 5H, Ar-*H*), 4.51 (sept, $^3J_{HH}$ = 6.7 Hz, 1H, L-iPr-*CH*), 3.34 (sept, $^3J_{HH}$ = 6.7 Hz, 1H, L-iPr-*CH*), 1.93 (d, $^2J_{HH}$ = 4.4 Hz, 1H, Si-C*H*$_2$-P), 1.90 (s, 1H, Si-C*H*$_2$-P), 1.76 (sept, $^3J_{HH}$ = 7.4 Hz, 1H, Si-iPr-*CH*), 1.46 (d, $^3J_{HH}$ = 6.8 Hz, 3H, L-iPr-*CH$_3$*), 1.37 (d, $^3J_{HH}$ = 7.4 Hz, 3H, Si-iPr-*CH$_3$*), 1.28 (d, $^3J_{HH}$ = 6.7 Hz, 3H, L-iPr-*CH$_3$*), 1.19 (d, $^3J_{HH}$ = 7.5 Hz, 3H, Si-iPr-*CH$_3$*), 1.17 (d, $^3J_{HH}$ = 6.7 Hz, 3H, L-iPr-*CH$_3$*), 1.07 (d, $^3J_{HH}$ = 6.7 Hz, 3H, L-iPr-*CH$_3$*), 0.99 (d, $^3J_{HH}$ = 6.0 Hz, 3H, Si-iPr-*CH$_3$*), 0.87 (m, 4H, Si-iPr-*CH* + Si-iPr-*CH$_3$*).

^{13}C{^{1}H} NMR (C_6D_6, 101 MHz, 298 K): δ = 166.8 (s, L-^{ipso}*C*), 147.8 (d, J_{PC} = 1.6 Hz, Ar-*C*), 147.5 (d, J_{PC} = 5.4 Hz, Ar-*C*), 146.2 (s, Ar-*C*), 136.6 (d, J_{PC} = 4.2 Hz, Sn-Ar-*C*), 134.2 (d, J_{PC} = 12.5 Hz, Ar-*C*), 133.4 (d, J_{PC} = 18.8 Hz, P-Ar-*C*) 131.7 (d, J_{PC} = 10.8 Hz, Ar-*C*), 131.4 (d, J_{PC} = 15.9 Hz, Ar-*C*), 131.3 (d, J_{PC} = 13.2 Hz, Ar-*C*), 129.7 (d, J_{PC} = 1.7 Hz, Ar-*C*), 129.0 (d, J_{PC} = 9.7 Hz, Ar-*C*), 128.6 (d, J_{PC} = 9.2 Hz, Ar-*C*), 127.14 (d, J_{PC} = 1.6 Hz, Ar-*C*), 124.3 (s, Ar-*C*), 123.3 (d, J_{PC} = 5.2 Hz, Ar-*C*), 28.4 (s, L-iPr-*CH$_3$*), 28.3 (s, L-iPr-*CH$_3$*), 28.2 (s, L-iPr-*CH*), 27.8 (s, L-iPr-*CH*), 23.4 (s, L-iPr-*CH$_3$*), 23.0 (s, L-iPr-*CH$_3$*), 21.1 (s, Si-iPr-*CH$_3$*), 20.7 (s, Si-iPr-*CH$_3$*), 20.3 (d, $^3J_{PC}$ = 2.5 Hz, Si-iPr-*CH*), 20.2 (s, Si-iPr-*CH$_3$*), 19.5 (s, Si-iPr-*CH$_3$*), 17.7 (d, $^3J_{PC}$ = 3.8 Hz, Si-iPr-*CH*), 10.2 (d, $^1J_{PC}$ = 5.7 Hz, Si-*CH$_2$*-P).

^{29}Si{^{1}H} NMR (C_6D_6, 99 MHz, 298 K): δ = 6.5 (d, $^2J_{Si,P}$ = 10.3 Hz, CH_2-*Si*-(^{i}Pr)$_2$).

^{31}P{^{1}H} NMR (C_6D_6, 162 MHz, 298 K): δ = - 4.3 (s, Ph$_2$-*P*-CH$_2$, $^1J_{117SnP}$ = 1285 Hz, $^1J_{119SnP}$ = 1344 Hz).

MS/LIFDI-HRMS found (calcd.) m/z: 685.2314 (685.2324) for [^{Ph}L(Ph)Sn:]$^+$.

Anal. calcd. for $C_{37}H_{48}SnNPSi$: C, 64.92 %; H, 7.07 %; N, 2.05; **found**: C, 64.49 %; H, 6.96 %; N, 2.06.

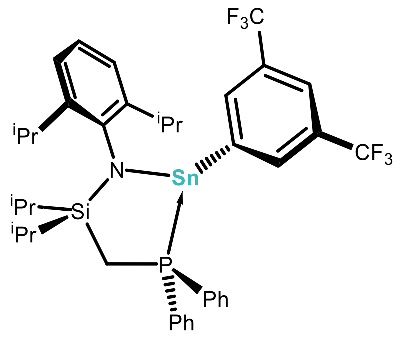

^{Ph}L[*m*-(CF$_3$)$_2$-Ph]Sn:, 1a-CF$_3$. The stannylene was synthesized according to the general method A using ^{Ph}L(Cl)Sn: (395 mg, 0.61 mmol, 1.00 eq.), and a freshly prepared *m*-(CF$_3$)$_2$-PhMgBr in THF using *m*-(CF$_3$)$_2$-PhBr (180 mg, 0.61 mmol, 1.00 eq.), Mg (22.4 mg, 0.92 mmol, 1.50 eq), and a crystal of iodide. The toluene solution was used without further purification assuming full conversion, backed up by NMR. Separately, crystalline compound for further analysis were obtained from a concentrated pentane solution at ambient temperature after 1 day.

^{1}H NMR (C_6D_6, 400 MHz, 298 K): δ = 8.02 (s, 2H, Ar-^{ortho}C*H*-CCF$_3$), 7.70 (s, 1H, Ar-^{para}C*H*-CCF$_3$), 7.60 (m, 2H, Ar-*H*), 7.10 (m, 6H, Ar-*H*), 6.96 (m, 1H, Ar-*H*), 6.88 (t, $^3J_{HH}$ = 6.7 Hz, 2H, Ar-*H*), 6.79 (m, 2H, Ar-*H*), 4.47 (sept, $^3J_{HH}$ = 6.9 Hz, 1H, L-iPr-*CH*), 3.21 (sept, $^3J_{HH}$ = 6.7 Hz, 1H, L-iPr-*CH*), 1.75 (virt dt, $^2J_{PH}$ = 14.7 Hz, J = 43.9 Hz, 2H, Si-C*H$_2$*-P), 1.47 (m, 4H, Si-iPr-*CH* + L-iPr-*CH$_3$*), 1.31 (d, $^3J_{HH}$ = 7.2 Hz, 3H, Si-iPr-*CH$_3$*), 1.21 (d, $^3J_{HH}$ = 6.7 Hz, 3H, L-iPr-*CH$_3$*), 1.16 (d, $^3J_{HH}$ = 7.6 Hz, 3H, Si-iPr-*CH$_3$*), 1.14 (d, $^3J_{HH}$ = 6.9 Hz, 3H, L-iPr-*CH$_3$*), 1.01 (d, $^3J_{HH}$ = 6.6 Hz, 3H, L-iPr-*CH$_3$*), 0.91 (d, $^3J_{HH}$ = 6.8 Hz, 3H, Si-iPr-*CH$_3$*), 0.82 (p, $^3J_{HH}$ = 7.2 Hz, 1H, Si-iPr-*CH*), 1.01 (d, $^3J_{HH}$ = 5.7 Hz, 3H, Si-iPr-*CH$_3$*).

^{13}C{^{1}H} NMR (C_6D_6, 101 MHz, 298 K): δ = 171.0 (s, L-^{ipso}*C*), 147.4 (d, J_{PC} = 1.7 Hz, Ar-*C*), 146.6 (d, J_{PC} = 5.2 Hz, Ar-*C*), 145.9 (d, J_{PC} = 1.2 Hz, Ar-*C*), 136.2 (s, Sn-Ar-*C*), 134.1 (d, J_{PC} = 12.2 Hz, Ar-*C*), 131.8 (d, J_{PC} = 1.7 Hz, Ar-*C*), 131.7 (d, $^2J_{FC}$ = 21.9 Hz, Ar-*C*), 130.9 (q, $^1J_{FC}$ = 11.0 Hz, Ar-*CF3*), 130.4 (d, J_{PC} = 2.2 Hz, Ar-*C*), 130.1 (s, Ar-*C*), 129.8 (s, Ar-*C*), 129.2 (d, J_{PC} = 9.7 Hz, Ar-*C*), 129.1 (d, J_{PC} = 9.7 Hz, Ar-*C*), 126.2 (s, Ar-*C*), 124.5 (s, Ar-*C*), 123.9 (s, Ar-*C*), 123.6 (s, Ar-*C*), 123.5 (s, Ar-*C*), 120.6 (s, Ar-^{ortho}*C*-CCF$_3$), 28.4 (s, L-iPr-*CH*), 28.4 (s, L-iPr-*CH$_3$*), 27.9 (s, L-iPr-*CH$_3$*), 27.6 (s, L-iPr-*CH*), 23.5 (s, L-iPr-*CH$_3$*), 22.7 (s, Si-iPr-*CH$_3$*), 20.9 (s, Si-iPr-*CH$_3$*), 19.8 (d, $^3J_{PC}$ = 2.3 Hz, Si-iPr-*CH*), 19.7 (s, Si-iPr-*CH$_3$*), 19.1 (s, Si-iPr-*CH$_3$*), 17.4 (d, $^3J_{PC}$ = 4.4 Hz, Si-iPr-*CH*), 10.2 (d, $^1J_{PC}$ = 3.8 Hz, Si-*CH$_2$*-P).

^{29}Si{^{1}H} NMR (C_6D_6, 99 MHz, 298 K): δ = 9.2 (d, $^2J_{Si,P}$ = 10.4 Hz, CH$_2$-*Si*-(^{i}Pr)$_2$).

^{31}P{^{1}H} NMR (C_6D_6, 162 MHz, 298 K): δ = - 1.0 (s, Ph$_2$-*P*-CH$_2$, $^1J_{117SnP}$ = 1316 Hz, $^1J_{119SnP}$ = 1374 Hz).

MS/LIFDI-HRMS found (calcd.) m/z: 821.2026 (821.2085) for [^{Ph}L[m-(CF$_3$)$_2$-Ph]Sn:]$^+$.

Anal. calcd. for $C_{39}H_{46}F_6NPSiSn$: C, 57.09%; H, 5.65%; N, 1.71%; **found**: C, 57.26%; H, 5.67%; N, 1.78%.

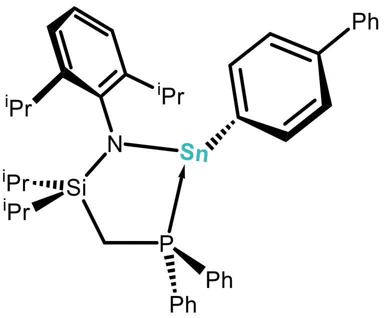

^{Ph}L(*p*-BPh)Sn:, 1a-*p*BP. The stannylene was synthesized according to the general method A using ^{Ph}LSnCl (0.40 g, 0.62 mmol, 1.00 eq.), and a freshly prepared *p*-BPhMgBr in THF using *p*-BPhBr (0.15 g, 0.62 mmol, 1.00 eq.), Mg (0.02 g, 0.93 mmol, 1.50 eq), and a crystal of iodide. The toluene solution was used without further purification assuming full conversion, backed up by NMR. Separately, crystalline compound for further analysis were obtained from a concentrated pentane solution at ambient temperature after 1 day.

^{1}H NMR (C_6D_6, 400 MHz, 298 K): δ = 7.77 (m, 2H, Ar-*H*), 7.70 (m, 2H, Ar-*H*), 7.50-7.56 (m, 4H, Ar-*H*), 7.19-7.26 (m, 3H, Ar-*H*), 7.10-7.15 (m, 6H, Ar-*H*), 6.84-6.88 (m, 5H, Ar-*H*), 4.56 (sept, $^{3}J_{HH}$ = 6.9 Hz, 1H, L-iPr-*CH*), 3.37 (sept, $^{3}J_{HH}$ = 6.9 Hz, 1H, L-iPr-*CH*), 1.96 (d, $^{2}J_{PH}$ = 14.1 Hz, 2H, Si-C*H*$_2$-P), 1.79 (sept, $^{3}J_{HH}$ = 7.5 Hz, 1H, Si-iPr-*CH*), 1.50 (d, $^{3}J_{HH}$ = 7.9 Hz, 3H, L-iPr-*CH$_3$*), 1.40 (d, $^{3}J_{HH}$ = 7.5 Hz, 3H, Si-iPr-*CH$_3$*), 1.32 (d, $^{3}J_{HH}$ = 6.8 Hz, 3H, L-iPr-*CH$_3$*), 1.23 (m, 6H, Si-iPr-*CH$_3$* + L-iPr-*CH$_3$*), 1.00 (d, $^{3}J_{HH}$ = 6.2 Hz, 3H, Si-iPr-*CH$_3$*), 0.85-0.89 (m, 7H, Si-iPr-*CH* + Si-iPr-*CH$_3$* + L-iPr-*CH$_3$*).

^{13}C{^{1}H} NMR (C_6D_6, 101 MHz, 298 K): δ = 165.9 (s, L-^{ipso}*C*), 147.8 (d, J_{PC} = 1.7 Hz, Ar-*C*), 147.6 (d, J_{PC} = 5.5 Hz, Ar-*C*), 146.2 (d, J_{PC} = 1.1 Hz, Ar-*C*), 142.2 (s, Ar-*C*), 140.0 (d, J_{PC} = 2.3Hz, Ar-*C*), 137.0 (d, J_{PC} = 4.2 Hz, Ar-*C*), 134.4 (s, Ar-*C*), 134.2 (s, Ar-*C*), 131.8 (s, Ar-*C*), 131.7 (s, Ar-*C*), 131.46 (s, Ar-*C*), 129.8 (d, J_{PC} = 2.8 Hz, Ar-*C*), 129.1 (s, Ar-*C*), 129.0 (s, Ar-*C*), 128.6 (s, Ar-*C*), 128.5 (s, Ar-*C*), 127.4 (s, Ar-*C*), 127.2 (s, Ar-*C*), 126.7 (d, J_{PC} = 1.5 Hz, Ar-*C*), 124.4 (s, Ar-*C*), 123.5 (s, Ar-*C*), 123.4 (s, Ar-*C*), 28.5 (s, L-iPr-*CH$_3$*), 28.3 (s, L-iPr-*CH*), 28.3 (s, L-iPr-*CH$_3$*), 27.9 (s, L-iPr-*CH*), 23.5 (s, iPr-*CH$_3$*), 23.0 (s, Si-iPr-*CH$_3$*), 21.2 (s, Si-iPr-*CH$_3$*), 20.8 (s, iPr-*CH$_3$*), 20.6 (d, $^{3}J_{PC}$ = 2.5 Hz, Si-iPr-*CH*), 20.3 (s, Si-iPr-*CH$_3$*), 19.5 (s, iPr-*CH$_3$*), 17.8 (d, J_{PC} = 3.8 Hz, Si-iPr-*CH*), 14.3 (s,), 10.3 (d, $^{1}J_{PC}$ = 5.3 Hz, Si-*CH$_2$*-P).

^{29}Si{^{1}H} NMR (C_6D_6, 99 MHz, 298 K): δ = 6.5 (d, $^{2}J_{Si,P}$ = 10.2 Hz, CH$_2$-*Si*-(^{i}Pr)$_2$).

^{31}P{^{1}H} NMR (C_6D_6, 162 MHz, 298 K): δ = - 4.2 (s, Ph$_2$-*P*-CH$_2$, $^{1}J_{117SnP}$ = 1283 Hz, $^{1}J_{119SnP}$ = 1345 Hz).

MS/LIFDI-HRMS found (calcd.) m/z: 761.2649 (761.2638) for [^{Ph}L(*p*-BPh)Sn:]$^{+}$.

Anal. calcd. for $C_{43}H_{52}NPSiSn$: C, 67.90%; H, 6.89%; N, 1.84%; **found**: C, 68.01%; H, 7.26%; N, 1.90%.

^{Ph}L(*o*-BPh)Sn:, 1a-*o*BP. The stannylene was synthesized according to the general method A using ^{Ph}L(Cl)Sn: (400 mg, 0.62 mmol, 1.00 eq.), and a freshly prepared *o*-BPhMgBr in THF using *o*-BPhBr (145 mg, 0.62 mmol, 1.00 eq.), Mg (22.7 mg, 0.93 mmol, 1.50 eq), and a crystal of iodide. The toluene solution was used without further purification assuming full conversion, backed up by NMR. Separately, crystalline compound for further analysis were obtained from a concentrated pentane solution at ambient temperature after 1 day.

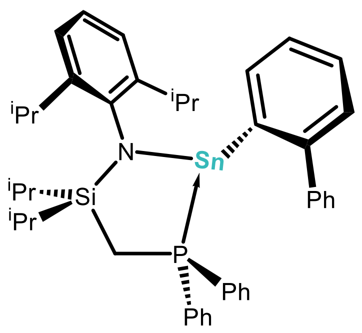

^{1}H NMR (C_6D_6, 400 MHz, 298 K): δ = 8.74 (d, $^3J_{HH}$ = 7.1 Hz, 1H, Sn-Ar-^{ortho}C*H*), 7.53 (t, $^3J_{HH}$ = 7.6 Hz, 1H, Sn-Ar-^{meta}C*H*), 7.27 (t, $^3J_{HH}$ = 7.4 Hz, 1H, Sn-Ar-^{para}C*H*), 7.13-6.84 (m, 17H, Ar-*H*), 6.57 (t, $^3J_{HH}$ = 9.2 Hz, 2H, Ar-*H*), 4.82 (q, $^3J_{HH}$ = 6.5 Hz, 1H, L-iPr-*CH*), 3.09 (q, $^3J_{HH}$ = 5.9 Hz, 1H, L-iPr-*CH*), 1.87 (q, $^3J_{HH}$ = 6.9 Hz, 1H, Si-iPr-*CH*), 1.78 (s, 1H, Si-CH_2-P), 1.75 (d, $^3J_{HP}$ = 2.7 Hz, 1H, Si-CH_2-P), 1.61 (d, $^3J_{HH}$ = 6.9 Hz, 3H, Si-iPr-CH_3), 1.55 (d, $^3J_{HH}$ = 6.4 Hz, 3H, L-iPr-CH_3), 1.30 (d, $^3J_{HH}$ = 5.9 Hz, 3H, L-iPr-CH_3), 1.09 (d, $^3J_{HH}$ = 6.4 Hz, 6H, L-iPr-CH_3 + Si-iPr-CH_3), 0.98 (m, 7H, Si-iPr-*CH* + L-iPr-CH_3 + Si-iPr-CH_3), 0.88 (s, 3H, Si-iPr-CH_3).

^{13}C{^{1}H} NMR (C_6D_6, 101 MHz, 298 K): δ = 165.0 (d, $^5J_{PC}$ = 2.5 Hz, L-^{ipso}*C*), 151.8 (d, J_{PC} = 3.3 Hz, Ar-*C*), 148.2 (d, J_{PC} = 5.8 Hz, Ar-*C*), 148.1 (s, Ar-*C*), 145.9 (s, Ar-*C*), 144.0 (s, Ar-*C*), 137.6 (d, J_{PC} = 2.7 Hz, Ar-*C*), 134.2 (s, Ar-*C*), 134.1 (s, Ar-*C*), 132.1 (d, J_{PC} = 10.5 Hz, Ar-*C*), 131.2 (s, Ar-*C*), 130.2 (d, J_{PC} = 2.1 Hz, Ar-*C*), 130.0 (s, Ar-*C*), 129.8 (d, J_{PC} = 2.6 Hz, Ar-*C*), 128.9 (s, Ar-*C*), 128.8 (s, Ar-*C*), 126.8 (s, Ar-*C*), 126.4 (d, J_{PC} = 1.8 Hz, Ar-*C*), 124.5 (s, Ar-*C*), 123.3 (s, Ar-*C*), 28.6 (s, L-iPr-*CH*), 28.2 (s, L-iPr-*CH*), 28.0 (s, 2C, iPr-CH_3), 23.5 (s, iPr-CH_3), 22.7 (s, L-iPr-CH_3), 21.0 (d, $^1J_{PC}$ = 9.0 Hz, Si-iPr-CH_3), 20.9 (s, L-iPr-CH_3), 20.2 (s, Si-iPr-CH_3), 19.5 (s, Si-iPr-CH_3), 18.0 (virt. m, 2C, Si-iPr-*CH*), 12.1 (d, $^1J_{PC}$ = 6.6 Hz, Si-CH_2-P).

^{29}Si{^{1}H} NMR (C_6D_6, 99 MHz, 298 K): δ = 6.8 (d, $^2J_{Si,P}$ = 11.8 Hz, CH_2-*Si*-(^{i}Pr)$_2$).

^{31}P{^{1}H} NMR (C_6D_6, 162 MHz, 298 K): δ = - 6.3 (s, Ph_2-*P*-CH_2, $^1J_{117SnP}$ = 1371 Hz, $^1J_{119SnP}$ =1435 Hz).

MS/LIFDI-HRMS found (calcd.) m/z: 761.2631 (761.2638) for [^{Ph}L(*o*-BPh)Sn:]$^+$.

Anal. calcd. for $C_{43}H_{52}NPSiSn$: C, 67.90%; H, 6.89%; N, 1.84%; **found**: C, 67.72%; H, 6.99%; N, 1.86%.

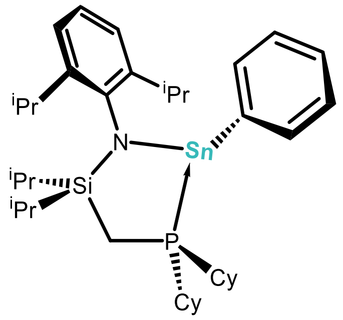

^{Cy}L(Ph)Sn:, 1b-Ph. The stannylene was synthesized according to the general method A using ^{Cy}L(Cl)Sn: (1.00 g, 1.52 mmol, 1.00 eq.) and a solution of freshly prepared PhMgBr in THF using PhBr (1.50 mL, 1.83 mmol, 1.20 eq.). The compound was not directly used in a subsequent reaction but was dried and crystallized in pentane at -32 °C to yield an off-white solid (890 mg, 1.28 mmol, 84%).

^{1}H NMR (C_6D_6, 400 MHz, 298 K): δ = 8.02 (d, $^3J_{HH}$ = 7.3 Hz, 2H, Ar-*H*), 7.36 (t, $^3J_{HH}$ = 7.1 Hz, 2H, Ar-*H*), 7.17-7.22 (m, 2H, Ar-*H*), 7.10-7.14 (m, 2H, Ar-*H*), 4.46 (sept, $^3J_{HH}$ = 7.3 Hz, 1H, L-iPr-*CH*), 3.74 (sept, $^3J_{HH}$ = 7.3 Hz, 1H, L-iPr-*CH*), 2.20-2.24 (m, 1H, Cy-*H*), 1.82-1.96 (m, 2H,

Cy-*H*), 1.66-1.73 (m, 3H, Si--iPr-*CH* + Cy-*H*), 1.54-1.61 (m, 6H, Cy-*H* + L-iPr-CH_3), 1.48-1.51 (m, 4H, Cy-*H* + L-iPr-CH_3), 1.43 (d, $^3J_{HH}$ = 7.2 Hz, 6H, L-iPr-CH_3 + Si-iPr-CH_3), 1.06-1.39 (m, 18H, Cy-*H* + L-iPr-CH_3 + Si-iPr-CH_3 + Si-iPr-*CH* + Si-CH_2-P), 1.02 (d, $^3J_{HH}$ = 6.8 Hz, L-iPr-CH_3), 0.92 (d, $^3J_{HH}$ = 7.2 Hz, Si-iPr-CH_3), 0.58-0.89 (m, 4H, Cy-*H*).

^{13}C{^{1}H} NMR (C_6D_6, 101 MHz, 298 K): δ = 166.3 (d, $^4J_{PC}$ = 3.5 Hz, L-^{ipso}C), 148.1 (d, $^5J_{PC}$ = 1.5 Hz, L-^{ortho}*C*H), 148.0 (d, J_{PC} = 6.2 Hz, Ar-*C*), 145.9 (s, Ar-*C*), 136.6 (d, $^5J_{PC}$ = 3.6 Hz, Sn-Ar-*C*), 127.2 (d, J_{PC} = 2.0 Hz, Ar-*C*), 124.2 (s, Ar-*C*), 123.4 (s, Ar-*C*), 123.2 (s, Ar-*C*), 35.4 (s, Cy-*C*), 35.3 (s, Cy-*C*), 35.2 (s, Cy-*C*), 35.0 (s, Cy-*C*), 32.2 (d, J_{PC} = 2.8 Hz, Cy-*C*), 29.9 (s, Alkyl-*C*), 29.7 (d, J_{PC} = 3.3 Hz, Cy-*C*), 29.3 (d, J_{PC} = 1.7 Hz, Cy-*C*), 28.6 (s, L-iPr-*C*H), 28.4 (s, L-iPr-CH_3), 28.2 (s, Alkyl-*C*), 28.0 (s, Alkyl-*C*), 27.7 (s, Alkyl-*C*), 27.7 (s, Alkyl-*C*), 27.6 (s, L-iPr-*C*H), 27.5 (s, Alkyl-*C*), 27.4 (s, Alkyl-*C*), 27.4 (s, Alkyl-*C*), 26.4 (s, Cy-*C*), 26.0 (s, Cy-*C*), 24.0 (s, L-iPr-CH_3), 23.0 (s, L-iPr-CH_3), 21.3 (s, Si-iPr-CH_3), 21.2 (d, J_{PC} = 2.0 Hz, Si-iPr-*C*H), 21.0 (s, Si-iPr-CH_3), 20.6 (s, Si-iPr-CH_3), 19.7 (s, Si-iPr-CH_3), 18.7 (d, J_{PC} = 4.3 Hz, Cy-*C*), 4.0 (d, J_{PC} = 8.1 Hz, Si-CH_2-P).

^{29}Si{^{1}H} NMR (C_6D_6, 99 MHz, 298 K): δ = 6.37 (d, $^2J_{Si,P}$ = 7.9 Hz, CH_2-*Si*-(^{i}Pr)$_2$).

^{31}P{^{1}H} NMR (C_6D_6, 162 MHz, 298 K): δ = 8.2 (s, CH_2-*P*-(Cy)$_2$, $^1J_{117SnP}$ = 1364 Hz, $^1J_{119SnP}$ = 1432 Hz).

MS/LIFDI-HRMS found (calcd.) m/z: 697.3288 (697.3263) for [^{Cy}L(Ph)Sn:]$^+$.

Anal. calcd. for $C_{37}H_{60}NPSiSn$: C, 63.79 %; H, 8.68 %; N, 2.01 %. **found**: C, 63.35 %; H, 9.07 %; N, 2.00 %.

General Methods for preparation of [{^{Ph}LSn(R)}·Ni·IPr], 2.

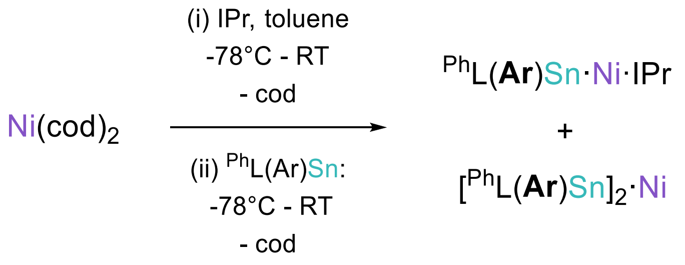

Method A: IPr (1.00 eq.) in toluene was added dropwise to the solution of Ni(cod)$_2$ (1.00 eq.) in toluene at -78°C and stirred for 30 min. Afterwards the yellow mixture allowed to warm up to RT and stirred for additional 20 min. The resulting orange solution was cooled to -78°C again and a toluene solution of **1** was added dropwise over 30 min. The mixture was

subsequently allowed to warm to ambient temperature and stirred for 5 h. The mixture was filtered, and all volatiles removed *in vacuo*. The residue was extracted with pentane and the solution stored at -32°C to form a crystalline solid of **2**.

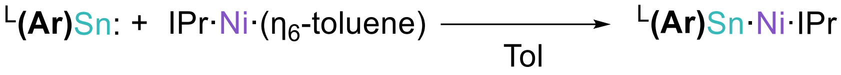

Method B: A toluene solution of **1** was added dropwise to a toluene solution IPr·Ni·(η^6-toluene) (1.00 eq.) at -80 °C. The mixture was subsequently allowed to slowly warm up to ambient temperature and stirred for 5 h. The mixture was filtered, and all volatiles removed *in vacuo*. The residue was extracted with pentane and the solution stored at -32°C to form a crystalline solid of **2**.

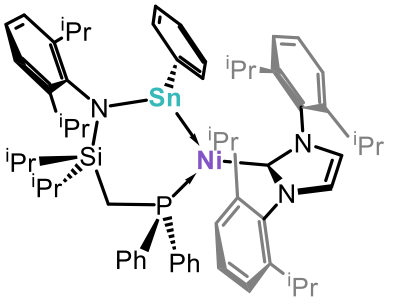

^{Ph}L(Ph)Sn·Ni·IPr, 2-Ph. The complex was synthesized according to **Method B** using **1a-Ph** (700 mg, 1.02 mmol, 1.01 eq.) and IPr·Ni·(η^6-toluene) (546 mg, 1.01 mmol, 1.00 eq.). The resulting deep orange-brown pentane solution was stored at -32°C for 7 days yielding dark orange crystals of **2-Ph** (331 mg, 0.37 mmol, 29%) suitable for X-Ray diffraction analysis.

^{1}H NMR (C_6D_6, 400 MHz, 298 K): δ = 7.65 (br m, 4H, Ar-*H*), 7.41 (t, $^3J_{HH}$ = 7.7 Hz, 2H, IPr-Ar-*H*), 7.23 (d, $^3J_{HH}$ = 7.7 Hz, 4H, IPr-Ar-*H*), 6.98-7.09 (m, 12H, Ar- *H*), 6.67 (s, 2H, N-C*H*=C*H*-N), 6.54 (d, $^3J_{HH}$ = 6.7 Hz, 2H, Ar-*H*), 3.20 (p, $^3J_{HH}$ = 5.7 Hz, 4H, IPr-L iPr-C*H*), 1.95 (d, $^3J_{HH}$ = 8.5 Hz, 2H, Si-CH_2-P), 1.11-1.30 (m, 21H), 1.01-1.05 (m, 25H, iPr-C*H* + iPr-CH_3).

^{13}C{^{1}H} NMR (C_6D_6, 101 MHz, 298 K): δ = 200.6 (d, $^2J_{PC}$ = 27.5 Hz, N-*C*-N), 167.4 (d, J_{PC} = 12.3 Hz, Ar-*C*), 148.6 (s, Ar-*C*), 146.9 (s, Ar-*C*), 144.4 (s, Ar-*C*), 142.2 (s, Ar-*C*), 142.0 (s, Ar-*C*), 138.0 (s, Ar-*C*), 136.2 (s, Ar-*C*), 133.1 (s, Ar-*C*), 132.6 (s, Ar-*C*), 132.5 (d, J_{PC} = 14.4 Hz, Ar-*C*), 129.6 (s, Ar-*C*), 128.8 (d, J_{PC} = 7.2, Ar-*C*), 127.6 (d, J_{PC} = 9.4, Ar-*C*), 127.2 (s, Ar-*C*), 127.1 (s, Ar-*C*), 125.0 (s, Ar-*C*), 124.0 (s, Ar-*C*), 123.6 (br s, N-*C*=*C*-N), 122.8 (s, Ar-*C*), 28.7 (s, L-iPr-*C*H_3), 27.7 (s, L-iPr-*C*H), 26.25 (s, L-iPr-*C*H_3), 24.0 (br s, iPr-*C*H), 22.5 (s, iPr-*C*H_3), 20.9 (s, iPr-*C*H), 19.7 (s, iPr-*C*H), 18.4 (d, J_{PC} = 2.1 Hz, Si- iPr-*C*H), 11.6 (d, $^1J_{PC}$ = 9.9 Hz, Si-*C*H_2-P).

^{29}Si{^{1}H} NMR (C_6D_6, 99 MHz, 298 K): δ = 2.2 (s, CH_2-*Si*-(^{i}Pr)$_2$).

$^{31}P\{^1H\}$ NMR (C_6D_6, 162 MHz, 298 K): δ = 16.1 (s, CH_2-*P*-$(Ph)_2$), $^1J_{117SnP}$ = 669 Hz, $^1J_{119SnP}$ = 694 Hz).

$^{119}Sn\{^1H\}$ NMR (C_6D_6, 112 MHz, 298 K): δ = 688 (d, $^2J_{SnP}$ = 698 Hz).

MS/LIFDI-HRMS found (calcd.) m/z: 1131.4472 (1131.4548) for $[^{Ph}L(Ph)Sn{\cdot}Ni{\cdot}IPr]^+$.

Anal. calcd. for $C_{64}H_{84}N_3NiPSiSn$: C, 67.92%; H, 7.48%; N, 3.71%; **found**: C, 66.26%, H, 7.57%; N, 3.76%.

λ_{max}, nm (*ε*, $Lcm^{-1}mol^{-1}$): 330 (11269), 406 (10568), 567 (5285), 818 (3357).

N.B. Repeated elemental analysis gave variable but consistently low values for C, possibly due to Si-carbide and Ni-carbide formation.[9]

$^{Ph}L\{$*m*$\text{-}(CF_3)_2\text{-}Ph\}Sn{\cdot}Ni{\cdot}IPr$, 2-CF$_3$. The complex was synthesized according to **Method B** using **1a-CF$_3$** (400 mg, 0.49 mmol, 1.01 eq.) and IPr·Ni·(η^6-toluene) (260 mg, 0.48 mmol, 1.00 eq.). The resulting deep green-brown pentane solution was stored at -32°C for 10 days yielding deep green-black crystals of **2-CF$_3$** (253 mg, 0.21 mmol, 43%) suitable for X-Ray diffraction analysis.

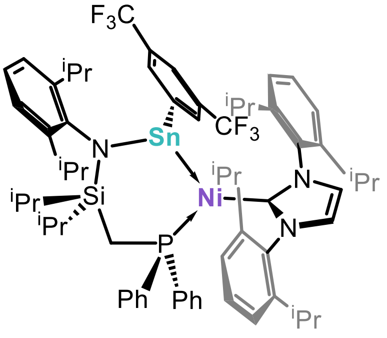

^1H NMR (C_6D_6, 400 MHz, 298 K): δ = 7.67 (s, 1H, Ar-*H*), 7.50 (m, 6H, Ar-*H* + IPr-Ar-*H)*, 7.26 (d, J_{HH} = 7.8 Hz, 4H, IPr-Ar-*H*), 7.09 (s, 4H, Ar-*H*), 7.01 (m, 7H, Ar-*H*), 6.64 (s, 2H, N-C*H*=C*H*-N), 3.09 (sept, J_{HH} = 6.7 Hz, 4H, L-iPr-*CH*), 1.81 (br s, 2H, Si-C*H*$_2$-P), 0.85-1.45 (m, 50H).

$^{13}C\{^1H\}$ NMR (C_6D_6, 101 MHz, 298 K): δ = 197.6 (d, $^2J_{PC}$ = 26.7 Hz, N-*C*-N), 170.0 (d, J_{PC} = 12.6 Hz, Ar-*C*), 147.3 (s, Ar-*C*), 146.7 (s, Ar-*C*), 144.0 (s, Ar-*C*), 141.1 (s, Ar-*C*), 140.8 (s, Ar-*C*), 137.5 (s, Ar-*C*), 135.5 (s, Ar-*C*), 132.2 (d, J_{PC} = 15.0 Hz, Ar-*C*), 130.4 (s, IPr-Ar-*C*), 129.3 (s, Ar-*C*), 129.00 (s, Ar-*C*), 128.8 (s, Ar-*C*), 126.2 (s, Ar-*C*), 125.0 (s, Ar-*C*),124.4 (s, Ar-*C*), 123.9 (s, Ar-*C*), 123.5 (s, Ar-*C*), 120.4 (br s, Ar-*C*), 28.7 (s, L-iPr-CH), 27.5 (s, iPr-C*H*$_3$), 26.2 (s, iPr-C*H*$_3$), 24.4 (s, iPr-C*H*$_3$), 22.6 (s, iPr-C*H*), 20.9 (s, iPr-C*H*$_3$), 19.5 (s, iPr-C*H*), 18.4 (s, iPr-C*H*$_3$), 14.3 (s, iPr-C*H*$_3$), 11.4 (d, $^1J_{PC}$ = 10.6 Hz, Si-*C*H$_2$-P).

$^{29}Si\{^1H\}$ NMR (C_6D_6, 99 MHz, 298 K): δ = 3.4 (d, J_{SiP} = 8.9 Hz, CH_2-*Si*-(^{i}Pr)$_2$).

$^{31}P\{^1H\}$ NMR (C_6D_6, 162 MHz, 298 K): δ = 19.5 (s, CH_2-*P*-$(Ph)_2$, $^1J_{117SnP}$ = 708 Hz, $^1J_{119SnP}$ = 740 Hz).

MS/LIFDI-HRMS found (calcd.) m/z: 1267.4192 (1267.4299) for $[^{Ph}L($*m*$\text{-}(CF_3)_2\text{-}Ph)Sn{\cdot}Ni{\cdot}IPr]^+$.

Anal. calcd. for $C_{66}H_{82}F_6N_3NiPSiSn$: C, 62.52%; H, 6.52%; N, 3.31%; **found**: C, 62.21%; H, 6.52%; N, 3.39%.

λ_{max}, nm (ε, $Lcm^{-1}mol^{-1}$): 343 (11662), 402 (11243), 573 (6205), 846 (3698).

N.B. Repeated elemental analysis gave variable but consistently low values for C, possibly due to Si-carbide and Ni-carbide formation.[9]

[^{Ph}L{*m*-(CF_3)$_2$-Ph}Sn]$_2$·Ni, 3. Employing **Method A**, using **1a-CF_3** (150 mg, 0.18 mmol), Ni(cod)$_2$ (98 mg, 0.18 mmol), and IPr (70 mg, 0.18 mmol), ^{31}P NMR analysis indicated the formation of a 2:1 mixture of **3** and **2-CF_3** (see Fig. S48). Filtration of this purple reaction mixture, removal of volatiles, and addition of pentane (5 mL) resulted in a purple solid beneath a red-brown solution. Filtration and drying *in vacuo* led to the isolation of **3** (63 mg, 41% based on **1-CF_3**).

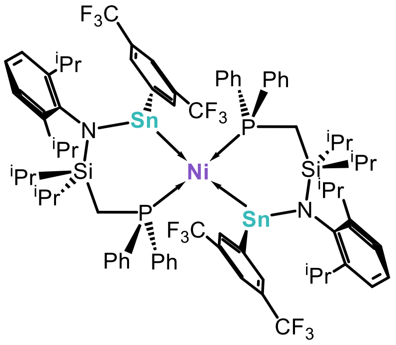

Compound **3** can also be accessed in a directly through the addition of **1-CF_3** to Ni(cod)$_2$: a mixture of those species in a 2:1 ratio (150 mg and 50 mg (0.18 mmol), respectively) is dissolved in toluene (20 mL) at ambient temperature, with rapid stirring, leading to the formation of deep purple solutions. Removal of all volatiles *in vacuo*, followed by the addition of pentane (5 mL), leads to formation of a purple solid, which is washed with a further 5 mL pentane, and dried *in vacuo*. The solid is identified as **3** (76%, 118 mg).

^{1}H NMR (C_6D_6, 400 MHz, 298 K): δ = 7.90 (t, J_{HH} = 8.3 Hz, 3H, Ar-*H*), 7.79 (s, 2H, Ar-*H*), 7.61 (m, 4H, Ar-*H*), 7.39 (s, 4H, Ar-*H*), 7.07-7.12 (m, 6H, Ar-*H*), 6.97 (m, 6H, Ar-*H*), 6.89 (t, J_{HH} = 7.7 Hz, 5H, Ar-*H*), 6.83 (d, J_{HH} = 6.9 Hz, 2H, Ar-*H*), 3.50 (sept, J_{HH} = 6.9 Hz, 2H, L-iPr-*CH*), 3.39 (sept, J_{HH} = 6.9 Hz, 2H, L-iPr-*CH*), 1.13-1.22 (m, 16H, L-iPr-*CH$_3$* + Si-iPr-*CH$_3$* + Si-*CH$_2$*-P), 0.98-1.08 (m, 10H, L-iPr-*CH$_3$* + Si-iPr-*CH* + Si-iPr-*CH$_3$*), 0.93 (d, J_{HH} = 7.3 Hz, Si-iPr-*CH$_3$*), 0.58-0.67 (m, 24H, L-iPr-*CH$_3$* + Si-iPr-*CH$_3$*).

^{13}C{^{1}H} NMR (C_6D_6, 101 MHz, 298 K): δ = 176.0 (s, Ar-*C*), 145.7 (s, Ar-*C*), 144.6 (s, Ar-*C*), 144.3 (s, Ar-*C*), 144.2 (s, Ar-*C*), 142.1 (s, Ar-*C*), 134.6 (s, Ar-*C*), 133.1 (s, Ar-*C*), 132.9 (s, Ar-*C*), 132.8 (d, J_{PC} = 8.0 Hz, Ar-*C*), 132.2 (br s, Ar-*C*), 131.2 (s, Ar-*C*), 130.5 (s, Ar-*C*), 129.4 (s, Ar-*C*), 128.8 (s, Ar-*C*), 128.7 (s, Ar-*C*), 125.8 (s, Ar-*C*), 125.7 (s, Ar-*C*), 124.9 (s, Ar-*C*), 124.5 (s, Ar-*C*), 124.2 (s, Ar-*C*), 123.5 (s, Ar-*C*), 123.1 (s, Ar-*C*), 122.6 (br s, Ar-*C*), 27.8 (s, L-iPr-*C*H), 27.7 (s, L-iPr-C*H$_3$*), 26.6 (s, L-iPr-C*H$_3$*), 26.1 (s, L-iPr-C*H$_3$*), 24.1 (s, iPr-C*H$_3$*), 24.0

(s, iPr-CH_3), 23.9 (s, iPr-CH_3), 19.5 (s, iPr-CH_3), 19.1 (s, iPr-CH_3), 18.4 (s, iPr-CH_x), 18.4 (s, iPr-CH_x), 18.3 (s, iPr-CH_x), 18.1 (s, iPr-CH_3), 14.3 (s, Si-CH$_2$-P), 14.0 (s, Si-CH$_2$-P).

^{29}Si{^{1}H} NMR (C_6D_6, 99 MHz, 298 K): δ = 6.2 (s, CH$_2$-*Si*-(^{i}Pr)$_2$).

^{31}P{^{1}H} NMR (C_6D_6, 162 MHz, 298 K): δ = 25.4 (s, CH$_2$-*P*-(Ph)$_2$).

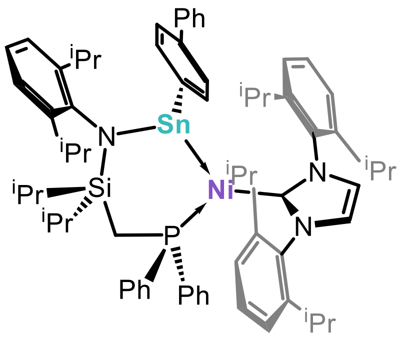

^{Ph}L(*p*-BPh)Sn·Ni·IPr, 2-*p*BP. The complex was synthesized according to the general **Method B** using **1a-*p*BP** (470 mg, 0.62 mmol, 1.00 eq.) and IPr·Ni·(η^6-toluene) (284 mg, 0.53 mmol, 1.00 eq.). The resulting brown pentane solution was stored at -32°C for 14 days yielding dark brown crystals of **2-*p*BP** (213 mg, 0.18 mmol, 36%) suitable for X-Ray diffraction analysis.

^{1}H NMR (C_6D_6, 400 MHz, 298 K): δ = 7.67 (br s, 3H, Ar-*H*), 7.48 (m, 2H, Ar-*H*), 7.43 (t, J_{HH} = 7.7 Hz, 2H, Ar-*H*), 7.32 (d, J_{HH} = 7.9 Hz, 2H, Ar-*H*), 7.25 (d, J_{HH} = 7.9 Hz, 4H, Ar-*H*), 7.09-7.14 (m, 6H, Ar-*H*), 6.99 (s, 7H, Ar-*H*), 6.68 (s, 2H, N-C*H*=C*H*-N), 6.64 (d, J_{HH} = 7.9 Hz, 4H, Ar-*H*), 3.20 (t, J_{HH} = 6.7 Hz, 4H, IPr-L-iPr-C*H*), 1.96 (br s, 2H, Si-CH_2-P), 0.85-1.45 (m, 50H).

^{13}C{^{1}H} NMR (C_6D_6, 101 MHz, 298 K): δ = 200.4 (d, $^2J_{PC}$ = 26.6 Hz, N-*C*-N), 167.0 (d, J_{PC} = 12.3 Hz, Ar-*C*), 148.7 (d, J_{PC} = 1.8 Hz, Ar-*C*), 146.9 (s, Ar-*C*), 144.5 (s, Ar-*C*), 142.5 (s, Ar-*C*), 142.3 (s, Ar-C), 142.0 (s, Ar-*C*), 142.0 (s, Ar-*C*), 139.8 (s, Ar-*C*), 138.0 (s, Ar-*C*), 136.7 (s, Ar-*C*), 132.6 (br s, Ar-*C*), 129.7 (s, Ar-*C*), 128.9 (s, Ar-*C*), 128.5 (s, Ar-*C*), 127.6 (s, Ar-*C*), 127.3 (s, Ar-*C*), 126.9 (s, Ar-*C*), 125.6 (s, Ar-*C*), 125.0 (s, Ar-*C*), 124.1 (s, Ar-*C*), 123.6 (br s, Ar-*C*), 122.8 (s, Ar-*C*), 28.7 (s, L-iPr-*C*H), 27.8 (s, iPr-CH_3), 26.3 (s, iPr-CH_3), 24.3 (br m, iPr-CH_3), 22.6 (s, iPr-C*H*), 20.9 (s, iPr-CH_3), 19.8 (br m, iPr-C*H*), 11.7 (d, $^1J_{PC}$ = 10.6 Hz, Si-*C*H$_2$-P).

^{29}Si{^{1}H} NMR (C_6D_6, 99 MHz, 298 K): δ = 2.2 (d, J_{SiP} = 9.7 Hz, CH$_2$-*Si*-(^{i}Pr)$_2$).

^{31}P{^{1}H} NMR (C_6D_6, 162 MHz, 298 K): δ = 16.4 (s, CH$_2$-*P*-(Ph)$_2$, $^1J_{117SnP}$ = 661 Hz, $^1J_{119SnP}$ = 690 Hz).

MS/LIFDI-HRMS found (calcd.) m/z: 1207.4830 (1207.4861) for [^{Ph}L(*p*-BPh)Sn·Ni·IPr]$^+$.

Anal. calcd. for $C_{70}H_{88}N_3NiPSiSn$: C, 69.60%; H, 7.34%; N, 3.48%; **found**: C, 68.13%; H, 7.33%; N, 3.53%.

λ$_{max}$, nm (*ε*, Lcm^{-1}mol^{-1}): 405 (13999), 569 (7314), 832 (4568).

N.B. Repeated elemental analysis gave variable but consistently low values for C, possibly due to Si-carbide and Ni-carbide formation.[9]

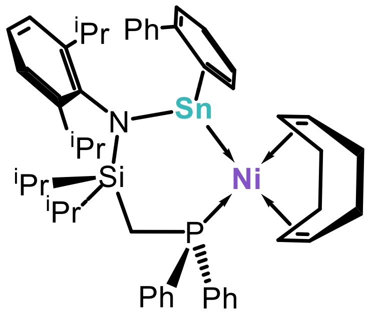

^{Ph}L(*o*-BPh)Sn·Ni(cod), 4. The complex was synthesized according to the general **Method A** using **1a-*o*BP** (470 mg, 0.62 mmol, 1.15 eq.) and Ni(cod)$_2$ (145 mg, 0.53 mmol, 1.00 eq.). The resulting deep purple pentane solution was stored at -32°C for 10 days yielding purple crystals of **4** (186 mg, 0.20 mmol, 38%) suitable for X-Ray diffraction analysis.

^{1}H NMR (C_6D_6, 400 MHz, 298 K): δ = 7.66 (d, J_{HH} = 7.3 Hz, 2H, Ar-*H*), 7.46-7.50 (m, 5H, Ar-*H*), 7.41 (t, J_{HH} = 7.7 Hz, 2H, Ar-*H*), 7.28 (t, J_{HH} = 7.7 Hz, 1H, Ar-*H*), 7.07-7.14 (m, 9H, Ar-*H*), 7.03 (t, J_{HH} = 7.3 Hz, 1H, Ar-*H*), 6.52 (d, J_{HH} = 7.3 Hz, 2H, Ar-*H*), 4.25 (br t, J_{HH} = 8.2 Hz, 2H, cod-C=C-*H*), 3.83 (sept, J_{HH} = 7.1 Hz, 2H, L-iPr-C*H*), 2.20 (d, J_{HH} = 10.7 Hz, 2H, Si-C*H*$_2$-P), 1.98 (br s, 2H, cod-C*H*$_2$), 1.84 (t, J_{HH} = 8.4 Hz, 2H, cod-C*H*$_2$), 1.45 (d, J_{PH} = 8.9 Hz, 2H, Si-iPr-C*H*), 1.30 (d, J_{HH} = 7.2 Hz, 6H, L-iPr-C*H*$_3$), 1.14-1.21 (m, 7H, L-iPr-C*H*$_3$ + Si-iPr-C*H*), 0.98 (d, J_{HH} = 7.3 Hz, 6H, Si-iPr-C*H*$_3$), 0.86-0.89 (m, 6H, Si-iPr-C*H*$_3$).

^{13}C{^{1}H} NMR (C_6D_6, 101 MHz, 298 K): δ = 173.5 (d, $^2J_{PC}$ = 12.0 Hz, N-*C*-N), 148.4 (s, Ar-*C*), 146.4 (s, Ar-*C*), 144.8 (s, Ar-*C*), 134.1 (s, Ar-*C*), 130.0 (s, Ar-*C*), 129.8 (s, Ar-*C*), 129.3 (s, Ar-*C*), 129.2 (s, Ar-*C*), 128.8 (s, Ar-*C*), 128.7 (s, Ar-*C*), 128.6 (s, Ar-*C*), 128.5 (s, Ar-*C*), 127.9 (s, Ar-*C*), 126.9 (s, Ar-*C*), 125.7 (s, Ar-*C*), 124.3 (br s, cod-*C*=*C*), 123.8 (s, Ar-*C*), 32.8 (s, cod-*C*H$_2$), 32.7 (s, cod-*C*H$_2$), 28.4 (s, Si-iPr-*C*H), 28.0 (s, L-iPr-*C*H), 25.0 (s, L-iPr-*C*H$_3$), 22.7 (s, L-iPr-*C*H$_3$), 21.4 (s, Si-iPr-*C*H), 19.0 (s, Si-iPr-*C*H$_3$), 18.5 (d, $^1J_{PC}$ = 5.3 Hz, Si-*C*H$_2$-P), 14.3 (s, Si-iPr-*C*H$_3$).

^{29}Si{^{1}H} NMR (C_6D_6, 99 MHz, 298 K): δ = 2.8 (s, CH$_2$-*Si*-(^{i}Pr)$_2$).

^{31}P{^{1}H} NMR (C_6D_6, 162 MHz, 298 K): δ = 23.2 (s, CH$_2$-*P*-(Ph)$_2$, $^1J_{117SnP}$ = 97 Hz, $^1J_{119SnP}$ = 108 Hz).

MS/LIFDI-HRMS found (calcd.) m/z: 819.1992 (819.1997) for [^{Ph}L(*o*-BPh)Sn·Ni]$^+$.

Anal. calcd. for $C_{51}H_{64}NNiPSiSn$: C, 66.04%; H, 6.96%; N, 1.15% **found**: C 64.52 %; H, 6.79 %; N, 1.46 %.

λ_{max}, nm (*ε*, Lcm^{-1}mol^{-1}): 551 (5992), 331 (20676).

N.B. Repeated elemental analysis gave variable but consistently low values for C, possibly due to Si-carbide and Ni-carbide formation.[9]

General method for hydrogen reactions of ^{Ph}L(Ar)Sn·Ni·IPr, forming 5.

^{Ph}L(**Ar**)Sn·Ni·IPr —[H_2, 1.5 bar, Toluene; - **Ar**-H]→ ^{Ph}LSn(μ–**H**)Ni·IPr

In a J. Young's NMR tube or pressure flask compounds **2-Ar** (20-300 mg) were dissolved in toluene(-d_8) or C_6D_6 and pressurized with dihydrogen (1.5 bar). After 16h the reaction yielded the desired product **5** quantitatively, as confirmed by ^{1}H and ^{31}P NMR.

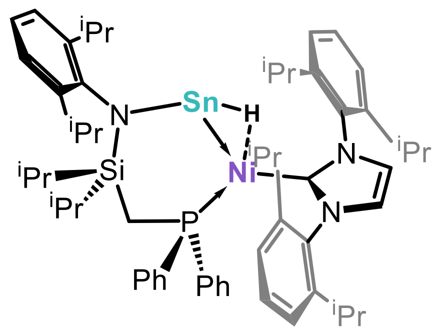

^{Ph}LSn(*μ*-H)Ni·IPr 5. Method A. The complex was synthesized according to the general method, using **2-Ph, 2-CF$_3$,** or **2-*p*BP** (30 mg, 0.05 mmol). In all cases, a deep orange-brown solution is formed and utilized for analysis.

Method B. To a solution of IPr·Ni·(η^6-toluene) (44.3 mg, 0.82 mmol, 1.00 eq.) in toluene (5 mL) cooled to -78 °C, is added ^{Ph}L(H)Sn: (50.0 mg, 0.82 mmol, 1.00 eq.) as a solution in toluene (2 mL). The reaction is warmed to RT and stirred for 1 h. All volatiles are subsequently removed, and the residue extracted into pentane, and filtered. The resulting deep orange-brown pentane solution, though largely consisting of **5**, did not yield crystals suitable for SC-XRD, which we hypothesize is due to the formation of **7** (see below).

^{1}H NMR (C_6D_6, 400 MHz, 298 K): δ = 7.40-7.45 (m, 4H, Ar-*H*), 7.21-7.26 (m, 3H, Ar-*H*), 7.11-7.14 (m, 6H, Ar-*H*), 7.01-7.04 (m, 1H, Ar-*H*), 6.93-6.96 (m, 5H, Ar-*H*), 6.70 (s, 2H, N-C*H*=C*H*-

N), 3.23 (br p, $^3J_{HH}$= 6.4 Hz, 4H, IPr-L-iPr-C*H*), 2.90 (p, $^3J_{HH}$= 6.9 Hz, 2H, L-iPr-C*H*), 2.13 (d, $^2J_{PH}$= 39.8 Hz, 1H, Sn-*H*-Ni), 2.05 (br d, $^2J_{PH}$= 10.7 Hz, 2H, Si-C*H*$_2$-P), 1.40 (d, $^3J_{HH}$= 7.0 Hz, 3H, iPr-C*H*$_3$), 1.36 (d, $^3J_{HH}$= 6.8 Hz, 6H, iPr-C*H*$_3$), 1.23 (d, $^3J_{HH}$= 6.8 Hz, 3H, iPr-C*H*$_3$), 1.20 (d, $^3J_{HH}$= 6.8 Hz, 3H, iPr-C*H*$_3$), 1.10-1.16 (m, 21H, iPr-C*H*$_3$ + Si-iPr-C*H*), 1.07 (d, $^3J_{HH}$= 6.9 Hz, 12H, iPr-C*H*$_3$), 1.00-1.02 (m, 2*H*, Si-iPr).

^{13}C{^{1}H} NMR (C_6D_6, 101 MHz, 298 K): δ = 209.05 (d, $^2J_{PC}$ = 15.8 Hz, N-*C*-N), 149.5 (s, Ar-*C*), 146.3 (s, Ar-*C*), 146.2 (s, Ar-*C*), 143.7 (s, Ar-*C*), 137.8 (s, Ar-*C*), 132.2 (br s, Ar-*C*), 129.5 (s, Ar-*C*), 128.2 (s, Ar-*C*), 127.3 (s, Ar-*C*), 124.3 (s, Ar-*C*), 123.4 (s, N-*C*=*C*-N), 123.1 (s, Ar-*C*), 123.0 (s, Ar-*C*), 122.9 (s, Ar-*C*), 122.2 (s, Ar-*C*), 119.1 (s, Ar-*C*), 28.8 (s, Alkyl-*C*), 28.4 (s, L-iPr-*C*H), 28.3 (s, IPr-L-iPr-*C*H), 28.1 (s, L-iPr-*C*H), 27.5 (s, Alkyl-*C*), 25.8 (s, Alkyl-*C*), 25.7 (s, Alkyl-*C*), 24.5 (s, Alkyl-*C*), 24.2 (s, Alkyl-*C*), 23.4 (s, iPr-*C*H$_3$), 23.2 (s, Alkyl-*C*), 22.8 (s, iPr-*C*H$_3$), 22.4 (s, iPr-*C*H$_3$), 20.5 (s, iPr-*C*H$_3$), 19.4 (d, J_{PC} = 13.2 Hz, Si-*C*H$_2$-P), 19.2 (br s, iPr-*C*H$_3$), 17.8 (d, J_{PC} = 3.7 Hz, Si-iPr).

^{29}Si{^{1}H} NMR (C_6D_6, 99 MHz, 298 K): δ = -0.1 (d, $^2J_{SiP}$ = 6.8 Hz, CH$_2$-*Si*-(^{i}Pr)$_2$).

^{31}P{^{1}H} NMR (C_6D_6, 162 MHz, 298 K): δ = -14.8 (s, CH$_2$-*P*-(Ph)$_2$).

MS/LIFDI-HRMS found (calcd.) m/z: 1055.4280 (1055.4235) for [^{Ph}LSn(*μ*-H)Ni]$^+$.

^{Cy}LSn(*μ*-H)Ni·IPr, 6. The complex was synthesized is the same manner as compound **5**, but using **1b-H** (139 mg, 0.22 mmol, 1.00 eq.) and IPr·Ni·(η^6-toluene) (121 mg, 0.22 mmol, 1.00 eq.). The resulting deep orange-brown pentane solution was stored at room temperature for 2 days yielding dark brown crystals of **6** (95.8 mg, 0.09 mmol, 40%) suitable for X-Ray diffraction analysis.

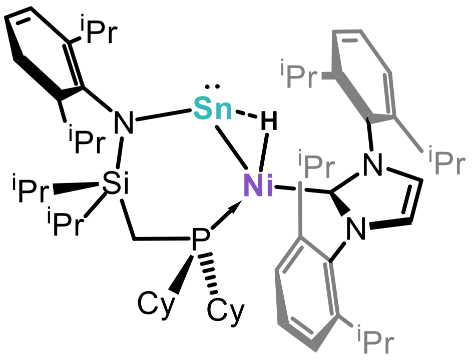

^{1}H NMR (C_6D_6, 400 MHz, 298 K): δ = 7.19-7.20 (m, 2H, Ar-*H*), 7.07-7.12 (m, 9H, Ar-*H*), 6.53 (s, 2H, N-C*H*=C*H*-N), 3.89 (p, $^3J_{HH}$= 7.0, 2H, L-iPr-C*H*), 3.33 (d, $^2J_{PH}$= 27.1, 1H, Sn-*H*-Ni), 3.21 (sept, $^3J_{HH}$= 6.9, 4H, IPr-L-iPr-C*H*), 1.97 (br s, 3H, Si-iPr-C*H* + Cy-*H*), 1.63-1.75 (m, 11H, Cy-*H*), 1.44-1.47 (m, 18H, L-iPr-C*H*$_3$ + IPr-L-iPr-C*H*$_3$), 1.32 (d, $^3J_{HH}$= 7.8, 6H, Si-iPr-C*H*$_3$), 1.28 (d, $^3J_{HH}$= 6.7, 6H, L-iPr-C*H*$_3$), 1.09-1.22 (m, 17 H, Si-iPr-C*H*$_3$ + Cy-*H*), 0.99-1.07(m, 16H, IPr-L-iPr-C*H*$_3$ + Cy-*H* + Si-C*H*$_2$-P).

^{13}C{^{1}H} NMR (C_6D_6, 101 MHz, 298 K): δ = 149.2 (s, Ar-*C*), 146.1 (s, Ar-*C*), 145.6 (s, Ar-*C*), 138.9 (s, Ar-*C*), 130.0 (s, Ar-*C*), 124.5 (s, Ar-*C*), 123.5 (s, Ar-*C*), 123.4 (s, Ar-*C*), 122.4 (s, Ar-

C), 30.7 (d, J_{CP} = 3.7 Hz, Cy-*C*), 29.7 (s, Cy-*C*), 28.6 (s, IPr-L-iPr-*C*H_3), 28.0 (s, Alkyl-*C*), 27.9 (s, Alkyl-*C*), 27.8 (s, Alkyl-*C*), 27.7 (s, Alkyl-*C*), 27.5 (s, Alkyl-*C*), 27.5 (s, L-iPr-*C*H_3), 26.9 (s, Alkyl-*C*), 25.9 (s, IPr-L-iPr-*C*H_3), 23.5 (s, iPr-*C*H_3), 23.4 (s, iPr-*C*H_3), 20.8 (s, Si-iPr-*C*H_3), 19.4 (s, Si-iPr-*C*H_3), 16.4 (d, J_{CP} = 4.1 Hz, Cy-*C*), 10.2 (d, J_{CP} = 22.0 Hz, Si-*C*H_2-P).

^{29}Si{^{1}H} NMR (C_6D_6, 99 MHz, 298 K): δ = -0.26 (d, $^2J_{SiP}$ = 5.4 Hz, CH_2-*Si*-(^{i}Pr)$_2$).

^{31}P{^{1}H} NMR (C_6D_6, 162 MHz, 298 K): δ = -8.8 (s, CH_2-*P*-(Cy)$_2$).

^{119}Sn{^{1}H} NMR (C_6D_6, 112 MHz, 298 K): δ = 1220 (br).

MS/LIFDI-HRMS found (calcd.) m/z: 1067.5184 (1067.5174) for [^{Ph}LSn(*μ*-H)Ni]$^+$.

Anal. calcd. for $C_{58}H_{92}N_3NiPSiSn$: C, 65.24 %; H, 8.68%; N, 3.94%; **found**: C, 63.79%; H, 9.05%; N, 3.89%.

N.B. Repeated elemental analysis gave variable but consistently low values for C, possibly due to Si-carbide and Ni-carbide formation.[9]

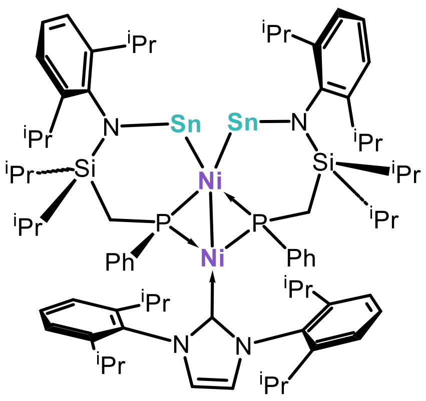

7. Method A. A toluene (30 mL) solution of **2-oBP** (500 mg, 0.41 mmol) was treated with a dihydrogen atmosphere (1.5 bar) at room temperature for 24 h. The volatiles were subsequently removed *in vacuo*, and the residue extracted in pentane (30 mL), and filtered. The resulting solution was concentrated to ~10 mL, and stored at -32°C for 5 weeks, yielding dark purple-brown crystals of **7** suitable for X-Ray diffraction analysis.

Method B. A toluene (20 mL) solution of **2-CF$_3$** (500 mg, 0.39 mmol) was treated with a dihydrogen atmosphere (1.5 bar) at room temperature for 24 h. The solution was subsequently heated to 60 °C for 10 days. ^{31}P NMR analysis indicated complete transformation to **7**. Subsequently, all volatile components were removed *in vacuo*, and the solid residue extracted in pentane (15 mL), filtered, concentrated to ~ 5 mL, and crystallized at 4 °C for 3 days, leading to the formation of purple crystals of **7** (46 mg, 0.09 mmol, 15%) suitable for X-ray diffraction analysis. We note that this species is highly soluble in aliphatic solvents, including pentane, hence the low isolated yield.

^{1}H NMR (C_6D_6, 400 MHz, 298 K): δ = 7.38-7.43 (m, 4H, *o-H*-Ph-P), 7.21-7.24 (m, 4H, Ar-*H*), 7.05-7.11 (m, 14H, Ar-*H*), 6.90-6.96 (m, 3H, Ar-*H*), 6.48 (s, 2H, N-C*H*=C*H*-N), 4.95 (p, $^3J_{HH}$= 6.5, 2H, IPr-L-iPr-C*H*), 3.69 (p, $^3J_{HH}$= 7.5, 2H, IPr-L-iPr-C*H*), 2.91 (p, $^3J_{HH}$= 6.9, 2H, L-iPr-

C*H*), 2.71 (p, $^3J_{HH}$= 6.7, 2H, L-iPr-C*H*), 1.96 (d, $^3J_{HH}$= 7.6, 6H, IPr-L-iPr-CH_3), 1.48 (d, $^3J_{HH}$= 7.0, 6H, IPr-L-iPr-CH_3), 1.45 (m, 6H, iPr-CH_3), 1.37-1.41 (m, 12H, iPr-CH_3), 1.30 (d, $^3J_{HH}$= 6.9, 6H, iPr-CH_3), 1.21-1.27 (m, 10H, Si-iPr-C*H*), 1.04 (d, $^3J_{HH}$= 7.2, 6H, Si-iPr-CH_3), 0.99-1.04 (m, iPr-CH_3 + Si-CH_2-P), 0.86-0.89 (m, 12H, iPr-CH_3), 0.79 (d, $^3J_{HH}$= 6.9, 6H, iPr-CH_3), 0.58 (d, $^3J_{HH}$= 6.9, 6H, iPr-CH_3), 0.28 (d, $^3J_{HH}$= 6.6, 6H, iPr-CH_3),

^{13}C{^{1}H} NMR (C_6D_6, 101 MHz, 298 K): δ = 151.1 (s, Ar-*C*), 145.8 (s, Ar-*C*), 145.6 (s, Ar-*C*), 145.0 (s, Ar-*C*), 138.5 (s, Ar-*C*), 131.2 (d, J_{PH} = 6.0, P-Ph-*o*-*C*), 130. 0 (s, Ar-*C*), 129.1 (s, Ar-*C*), 129.0 (s, Ar-*C*), 127.5 (s, Ar-*C*), 125.2 (s, Ar-*C*), 124.8 (s, N-*C*=*C*-N), 124.4 (s, Ar-*C*), 123.8 (s, Ar-*C*), 123.7 (s, Ar-*C*), 123.6 (s, Ar-*C*), 122.9 (s, Ar-*C*), 29.2 (s, iPr-CH_3), 28.7 (s, L-iPr-*C*H), 27.6 (s, IPr-L-iPr-*C*H), 25.2 (s, iPr-CH_3), 24.9 (s, iPr-CH_3), 24.7 (s, iPr-CH_3), 24.2 (s, iPr-CH_3), 23.6 (s, iPr-*C*H), 23.4 (s, iPr-CH_3), 23.0 (s, iPr-CH_3), 22.7 (s, iPr-CH_3), 22.4 (s, iPr-CH_3), 21.5 (s, alkyl-*C*), 21.2 (s, alkyl-*C*), 20.4 (s, IPr-L-iPr-*C*H), 18.5 (s, alkyl-*C*), 18.3 (s, alkyl-*C*), 17.1 (s, alkyl-*C*).

^{29}Si{^{1}H} NMR (C_6D_6, 99 MHz, 298 K): δ = 11.0 (d, $^2J_{SiP}$ = 4.0 Hz, CH_2-*Si*-(^{i}Pr)$_2$).

^{31}P{^{1}H} NMR (C_6D_6, 162 MHz, 298 K): δ = 89.0 (s, CH_2-*P*-Ph).

NMR, MS, UV/vis spectra of the synthesized compounds

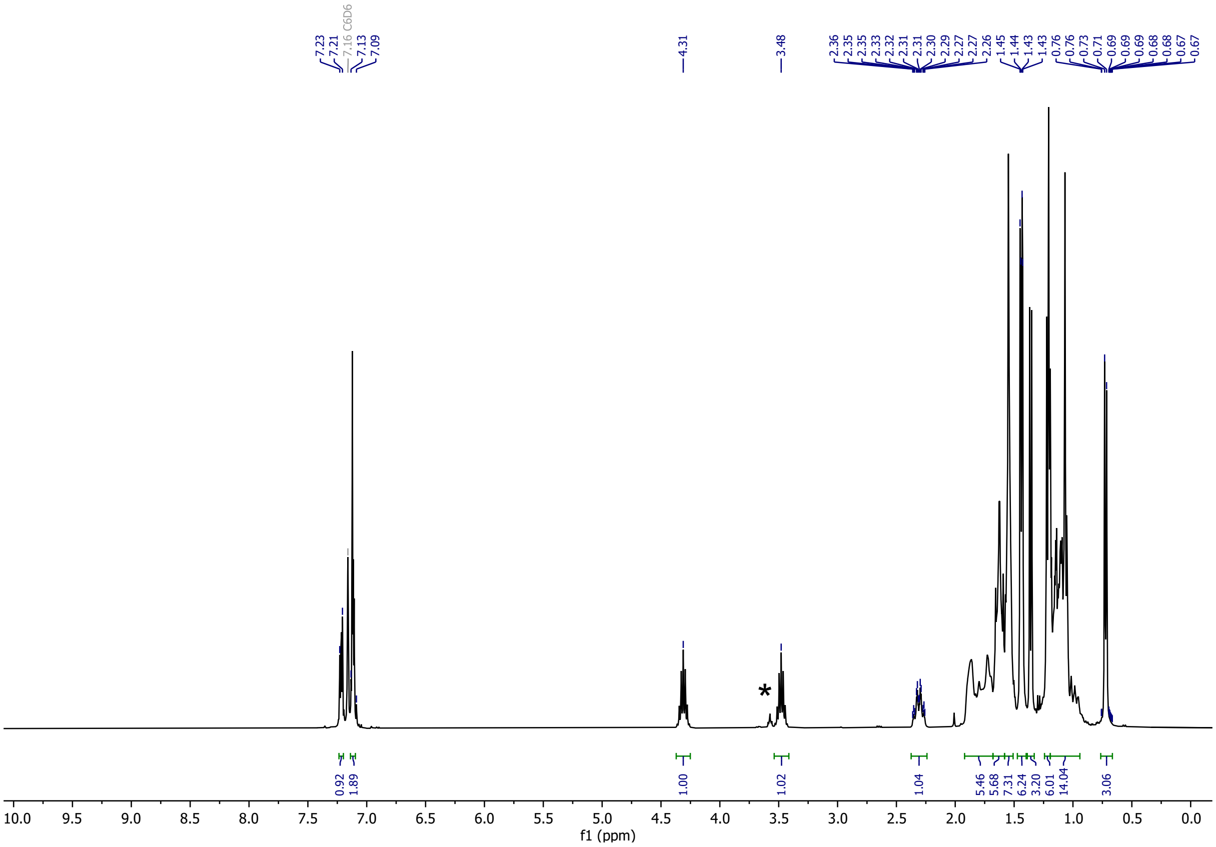

Figure S1. ^{1}H NMR spectrum (400 MHz, C_6D_6, 298K) of $^{Cy}L(Cl)Sn$:. * shows an unidentified impurity.

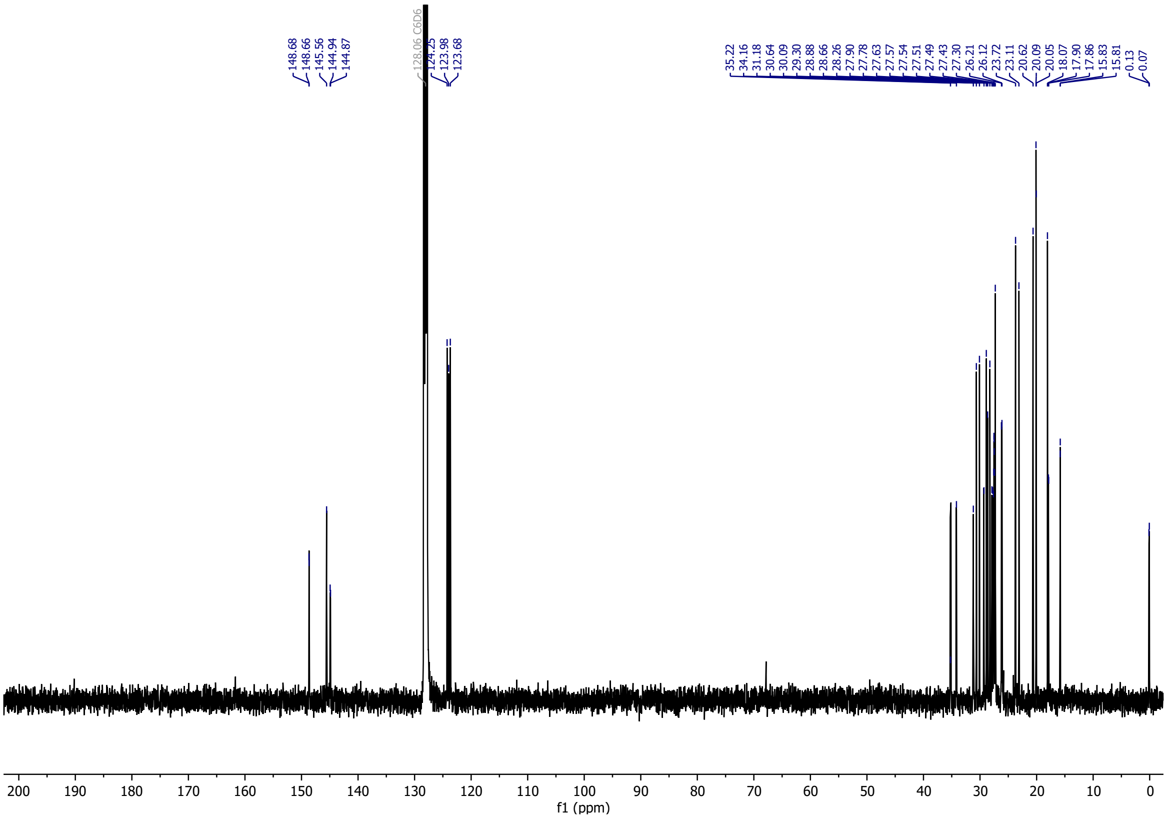

Figure S2. ^{13}C NMR spectrum (400 MHz, C_6D_6, 298K) of $^{Cy}L(Cl)Sn$:.

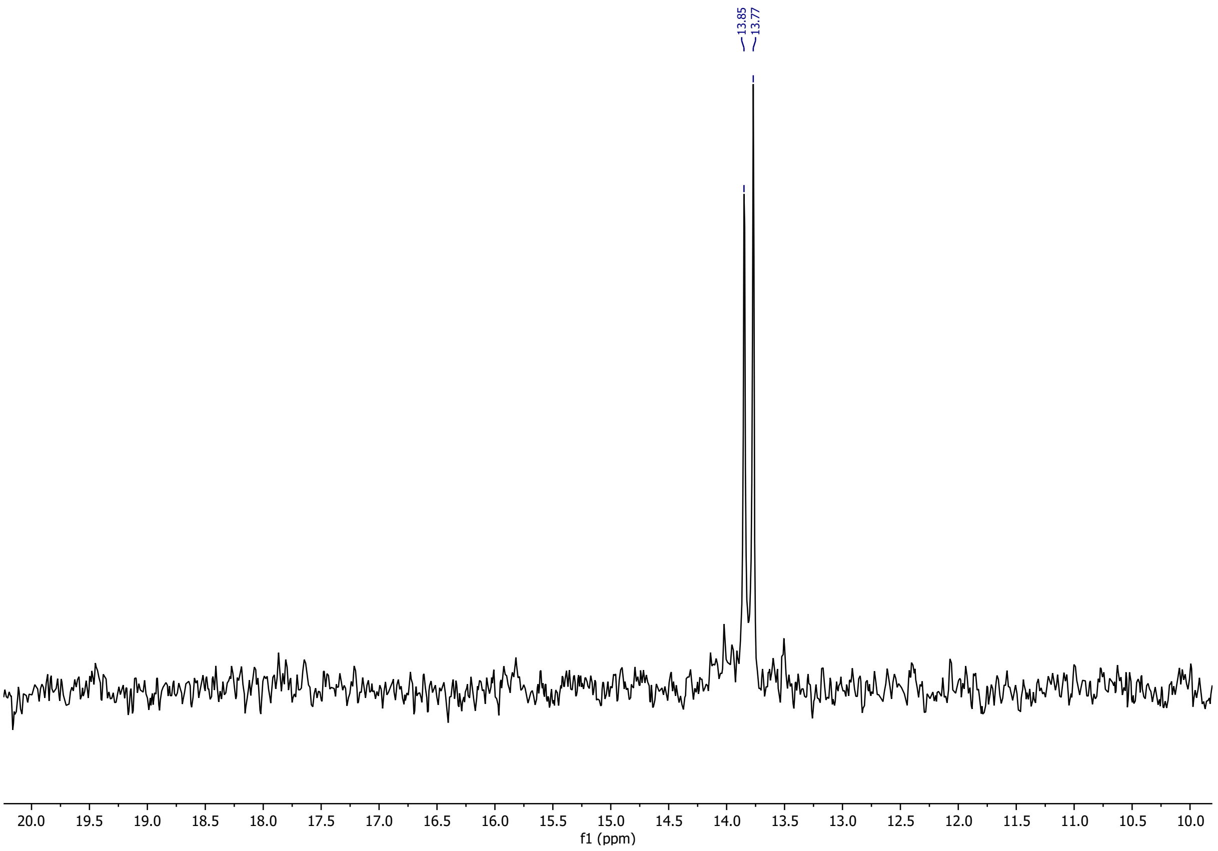

Figure S3. $^{29}Si\{^1H\}$ NMR spectrum (99 MHz, C_6D_6, 298K) of $^{Cy}L(Cl)Sn$:.

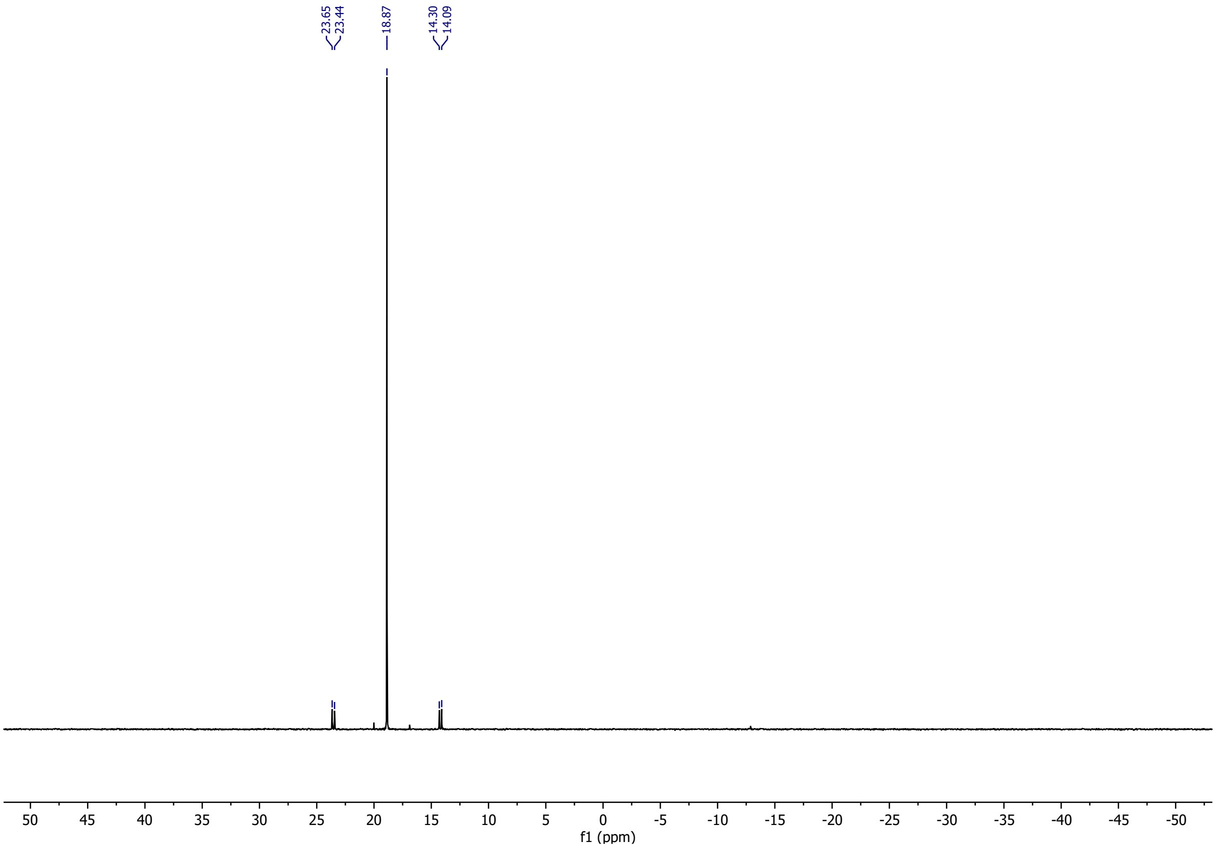

Figure S4. $^{31}P\{^1H\}$ NMR spectrum (162 MHz, C_6D_6, 298K) of $^{Cy}L(Cl)Sn$:.

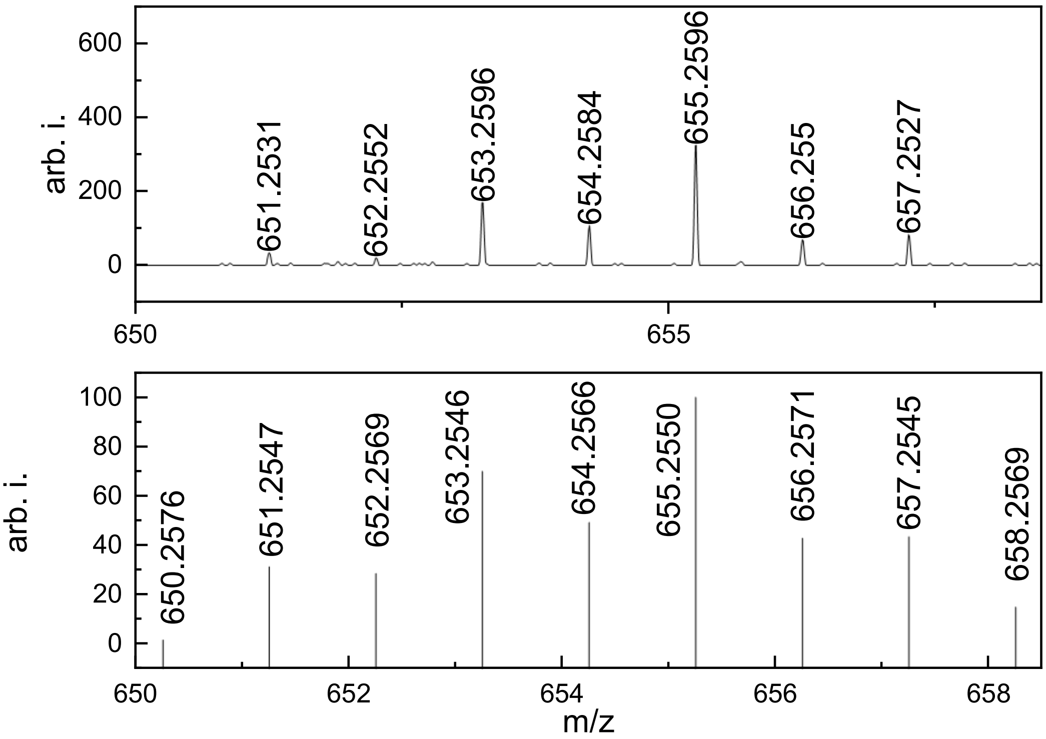

Figure S5. LIFDI-MS of **^{Cy}L(Cl)Sn:,** Top found MS for [^{Cy}L(Cl)Sn:]$^+$. Bottom. Calculated MS spectrum of [^{Cy}L(Cl)Sn:]$^+$.

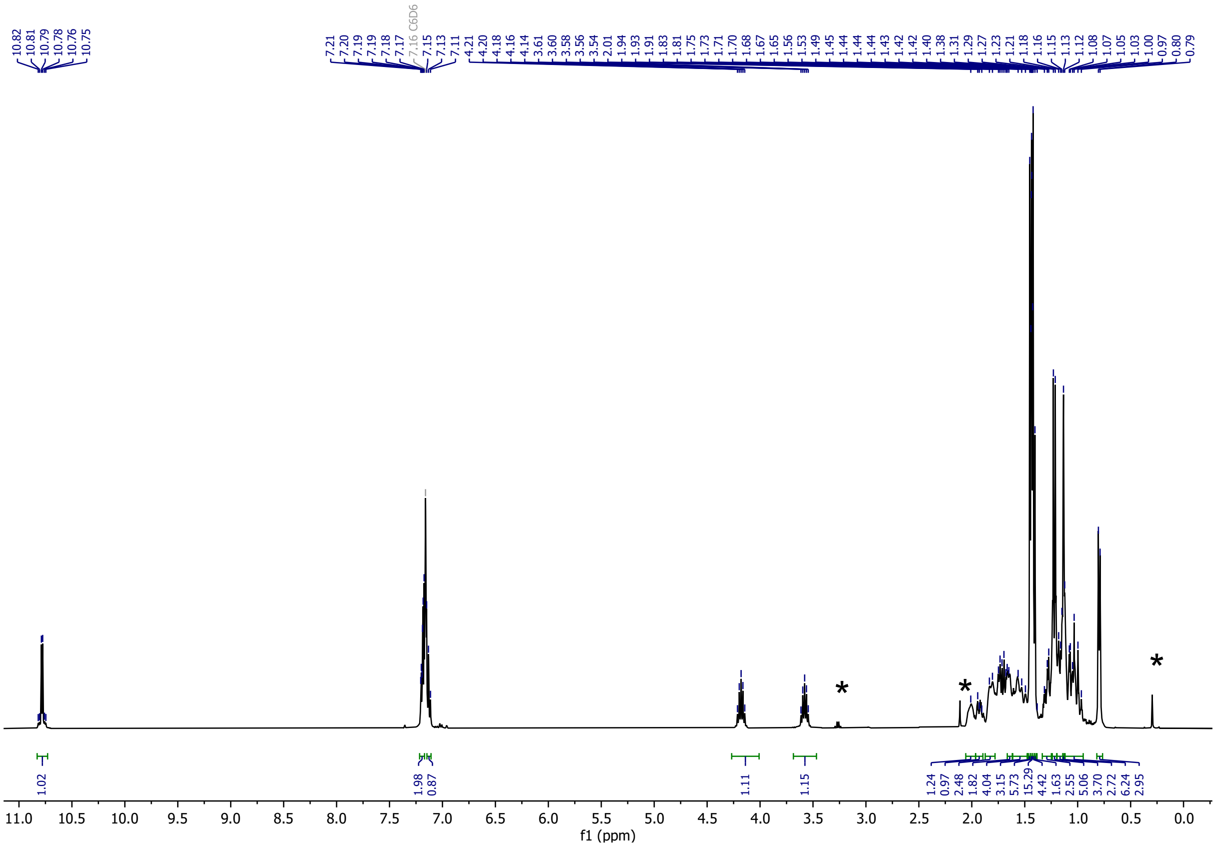

Figure S6. ^{1}H NMR spectrum (400 MHz, C_6D_6, 298K) of **1b-H**. * shows an unidentified impurity.

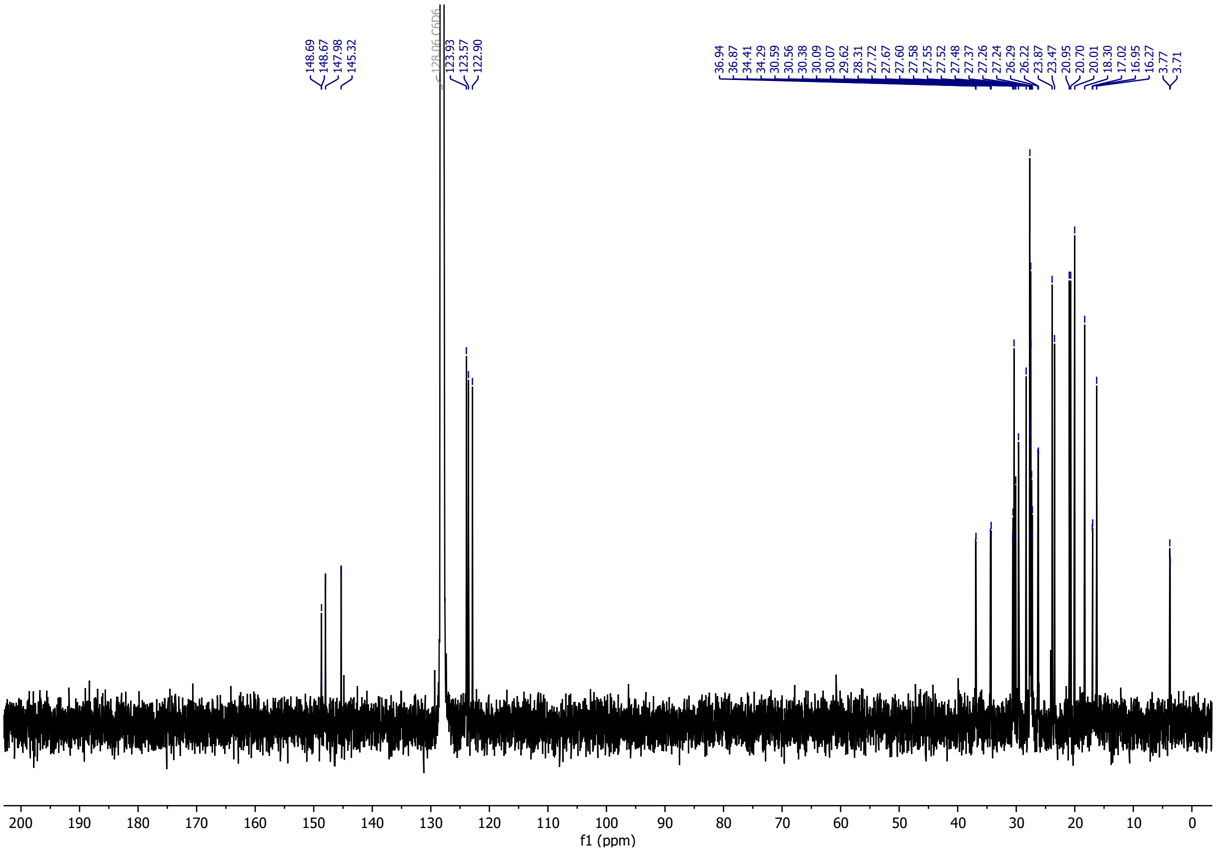

Figure S7. ^{13}C NMR spectrum (400 MHz, C_6D_6, 298K) of **1b-H**.

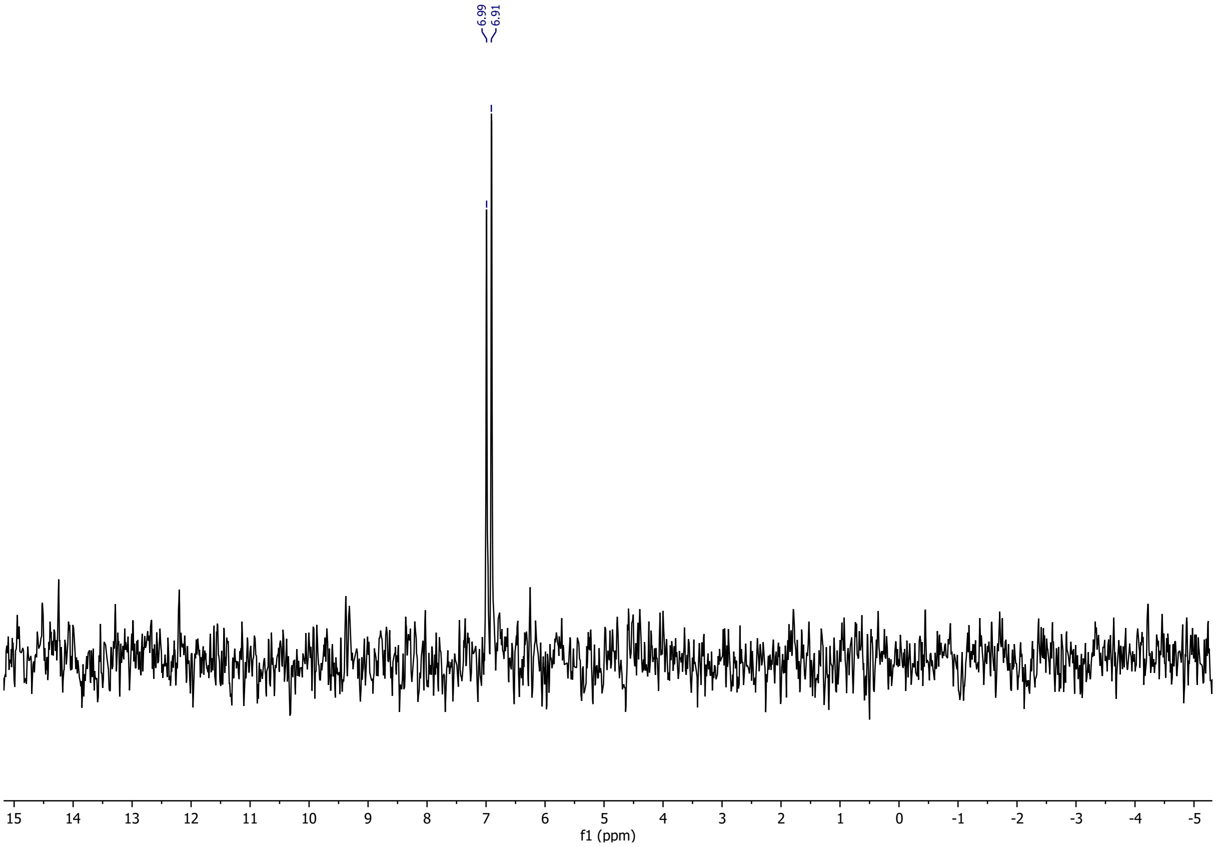

Figure S8. $^{29}Si\{^1H\}$ NMR spectrum (99 MHz, C_6D_6, 298K) of **1b-H**.

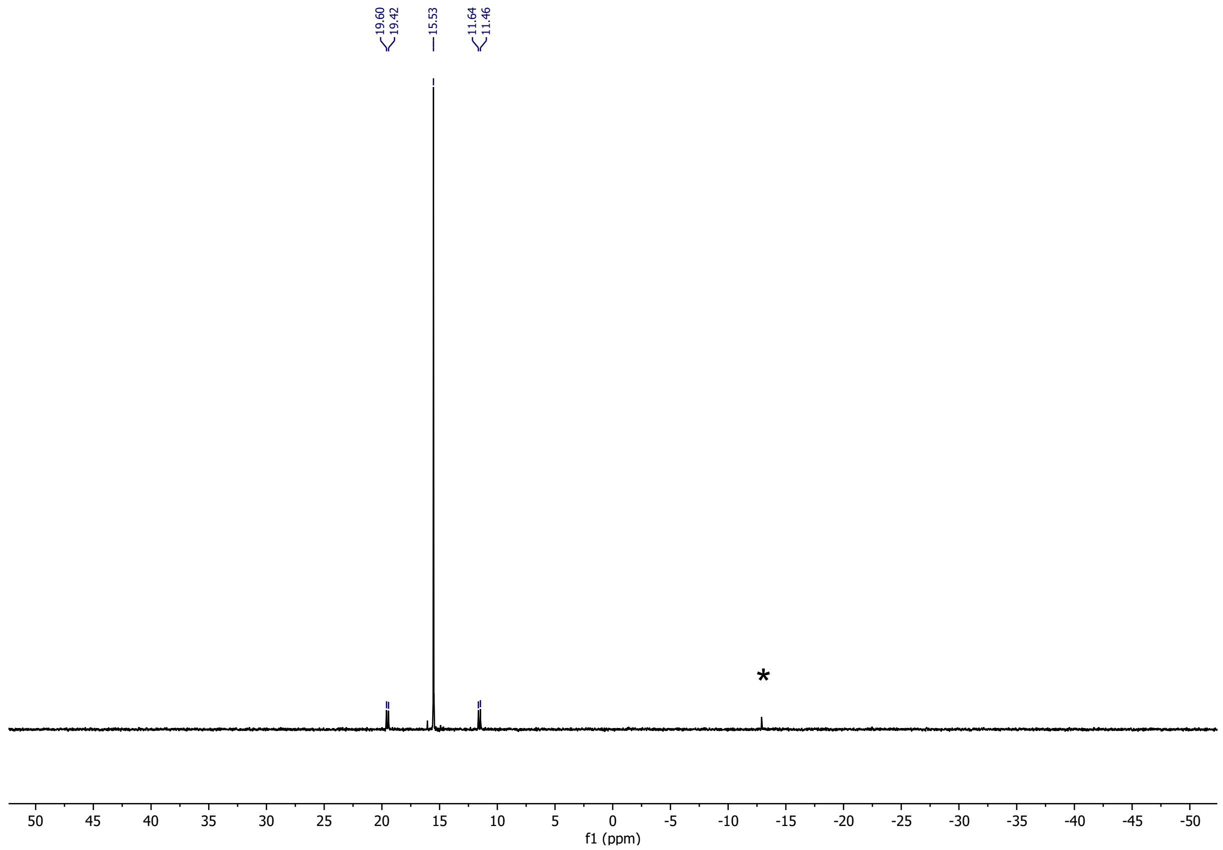

Figure S9. $^{31}P\{^{1}H\}$ NMR spectrum (162 MHz, C_6D_6, 298K) of **1b-H**.

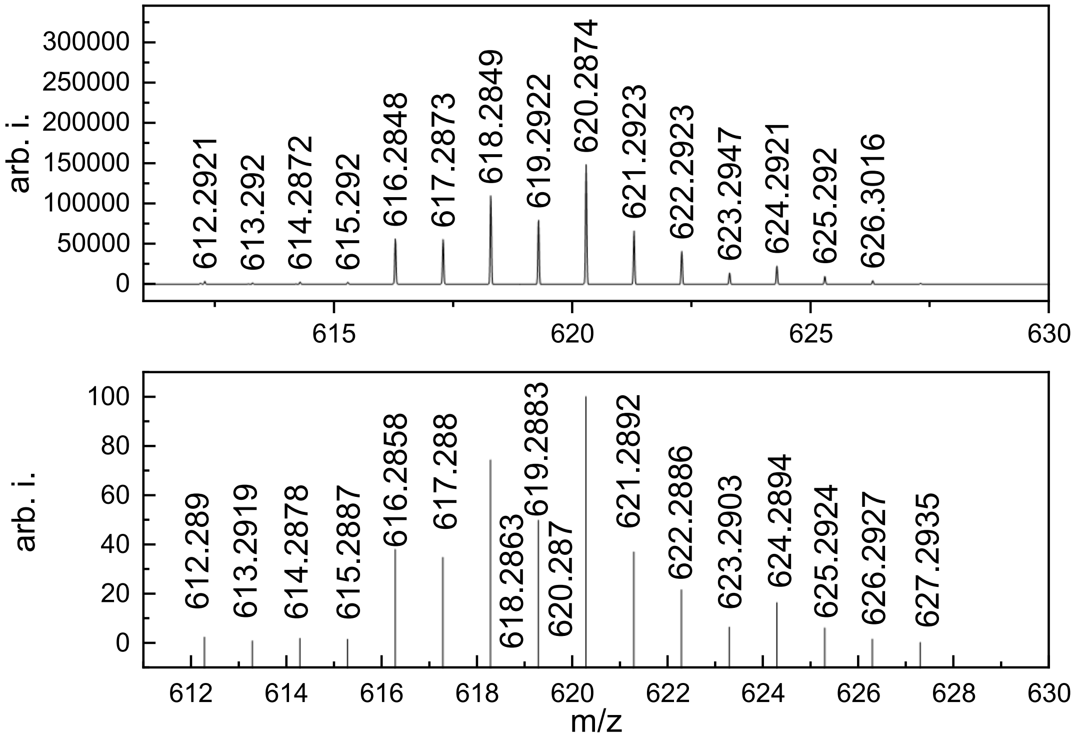

Figure S10. LIFDI-MS of **1b-H,** Top found MS for $[^{Ph}LSn]^{+}$. Bottom. Calculated MS spectrum of $[^{Ph}LSn]^{+}$.

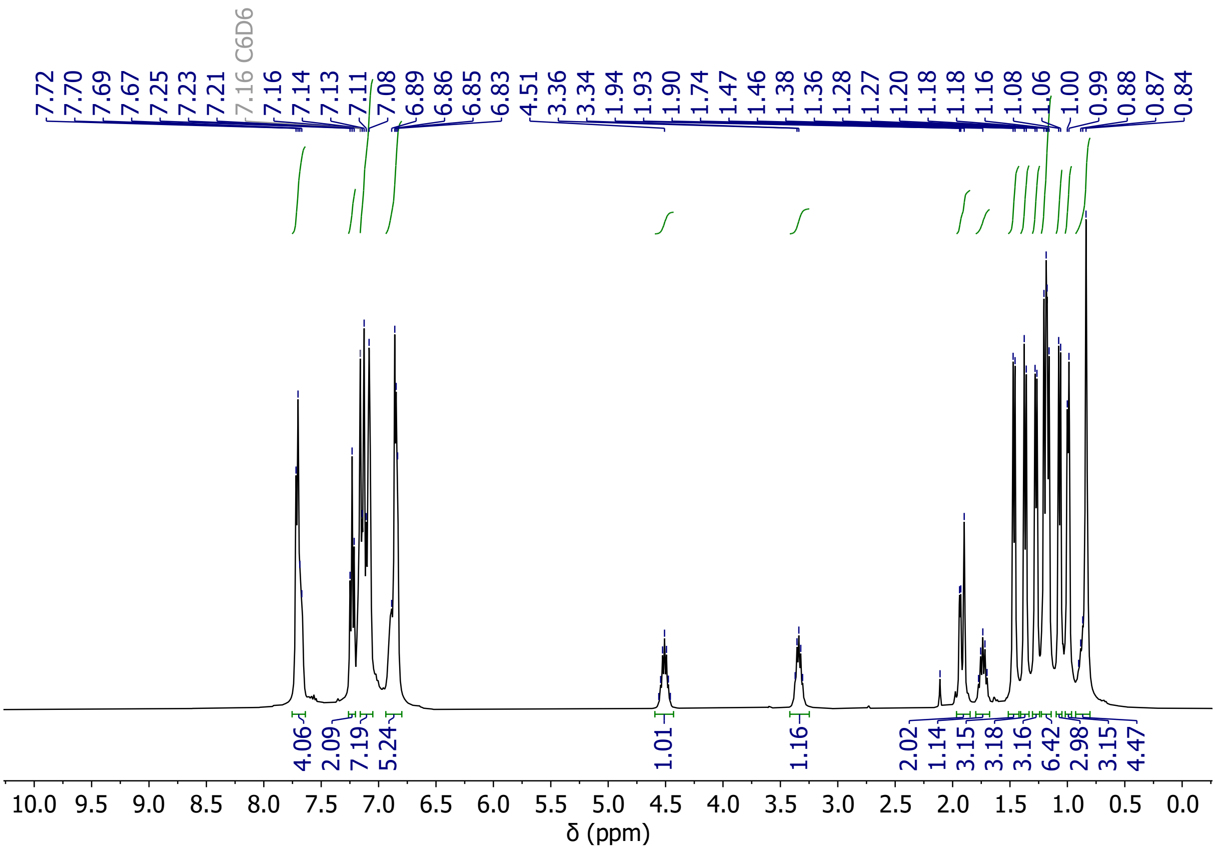

Figure S11. ^{1}H NMR spectrum (400 MHz, C_6D_6, 298K) of **1a-Ph**.

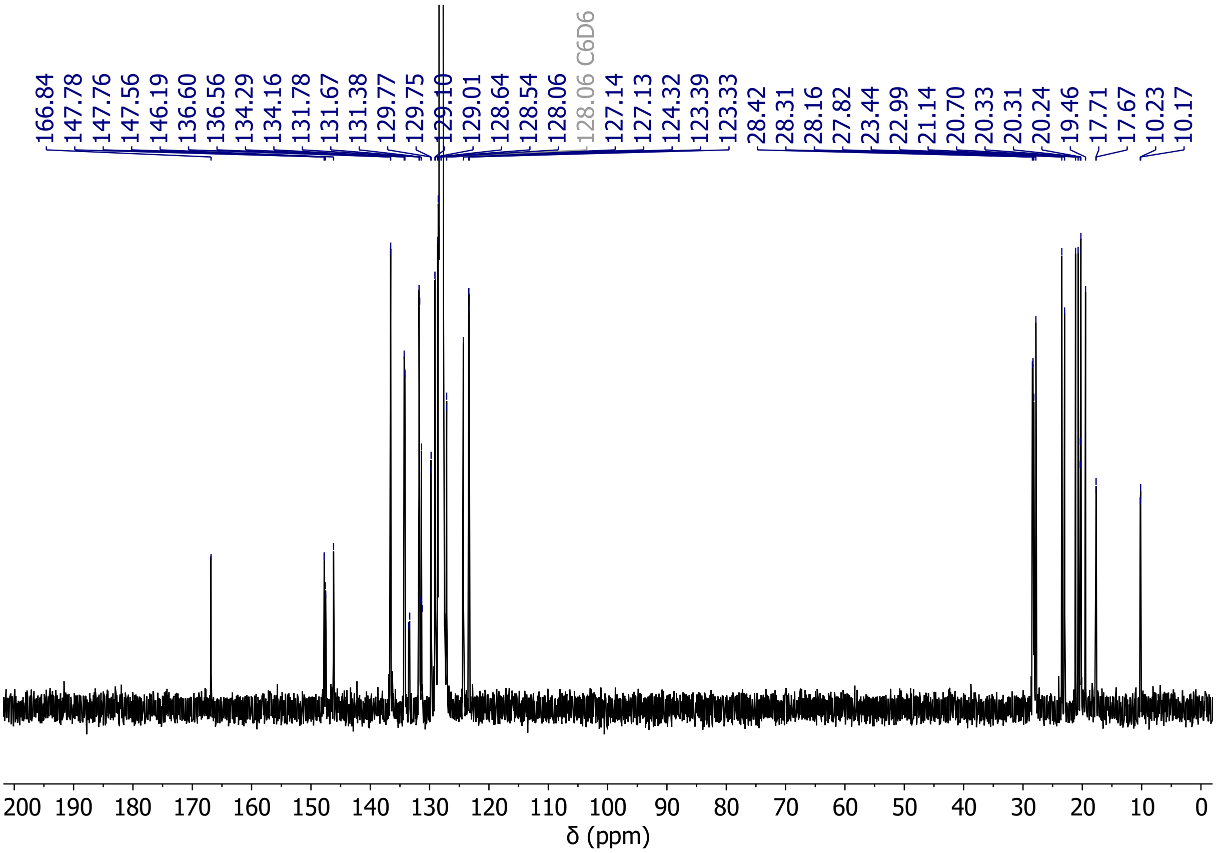

Figure S12. ^{13}C NMR spectrum (400 MHz, C_6D_6, 298K) of **1a-Ph**.

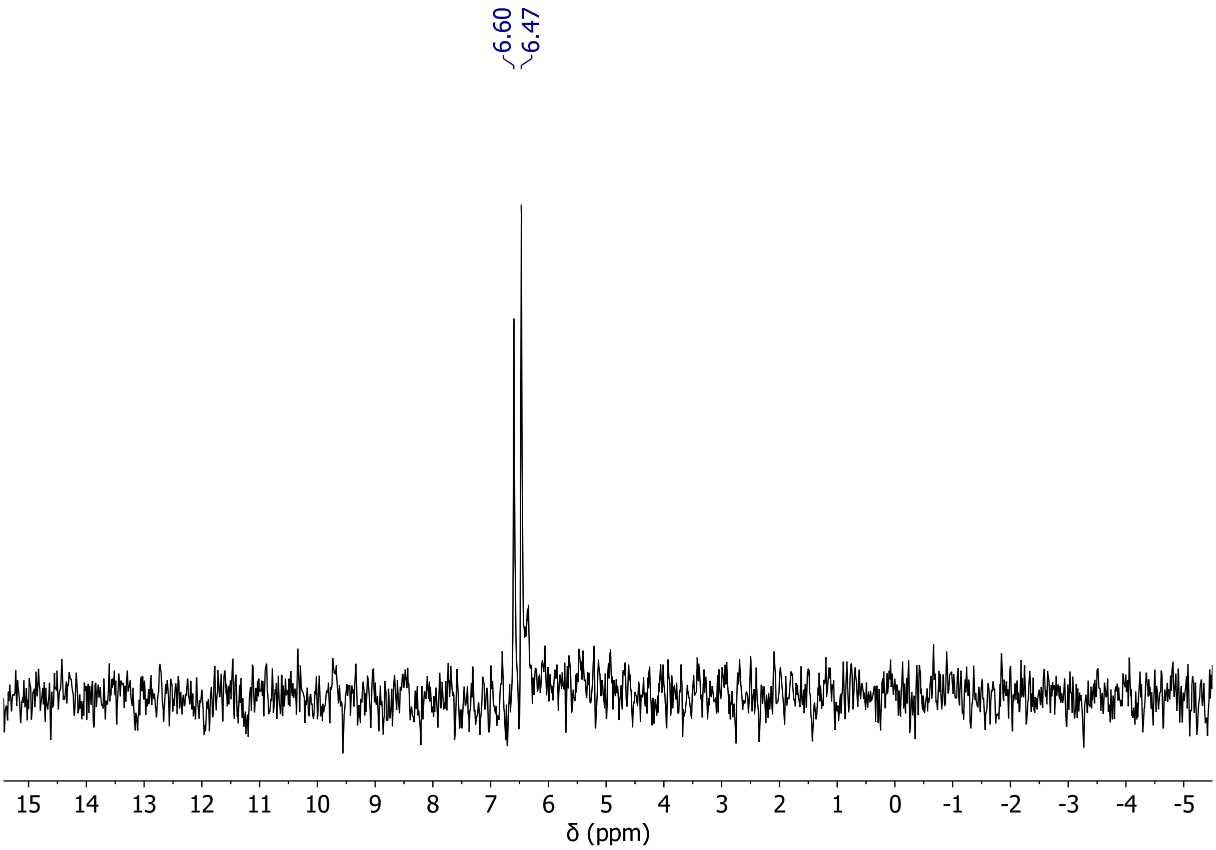

Figure S13. $^{29}Si\{^1H\}$ NMR spectrum (99 MHz, C_6D_6, 298K) of **1a-Ph**.

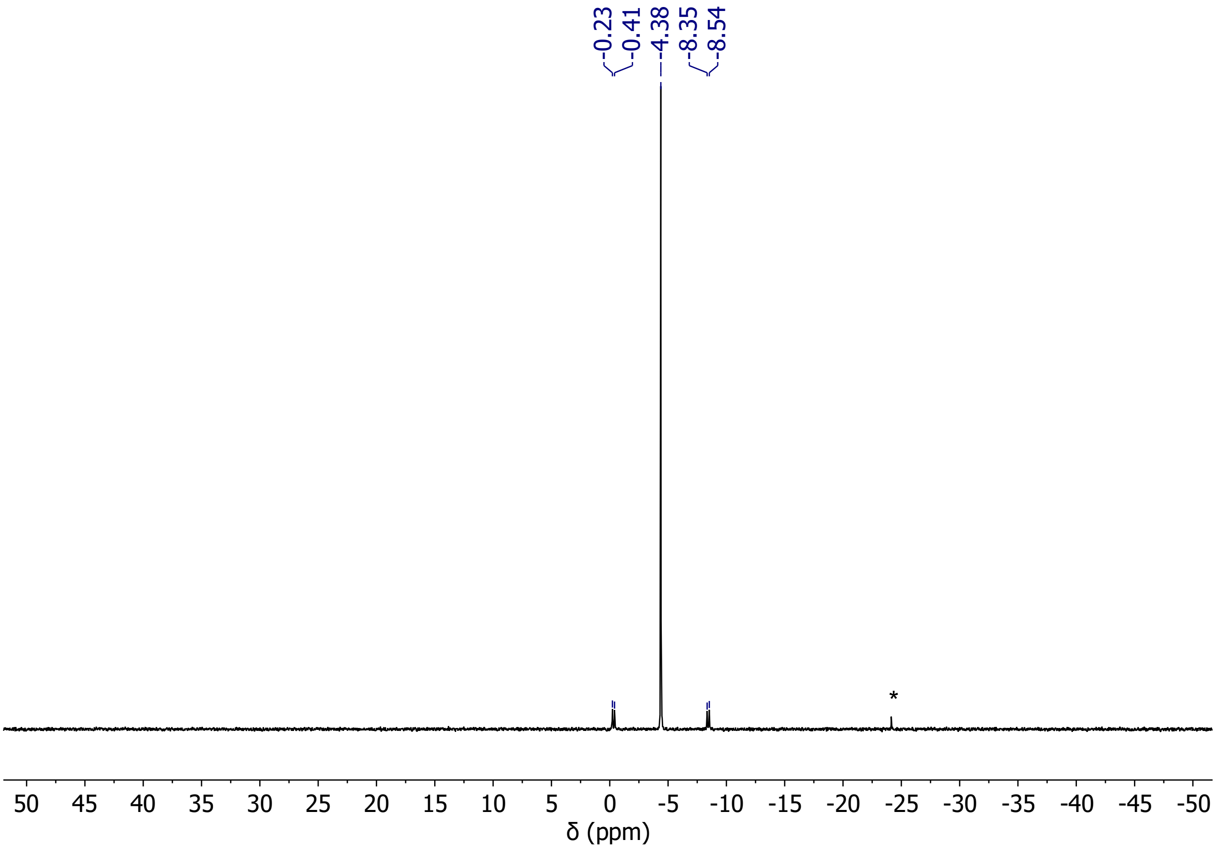

Figure S14. $^{31}P\{^1H\}$ NMR spectrum (162 MHz, C_6D_6, 298K) of **1a-Ph**. * marks ^{Ph}LH.

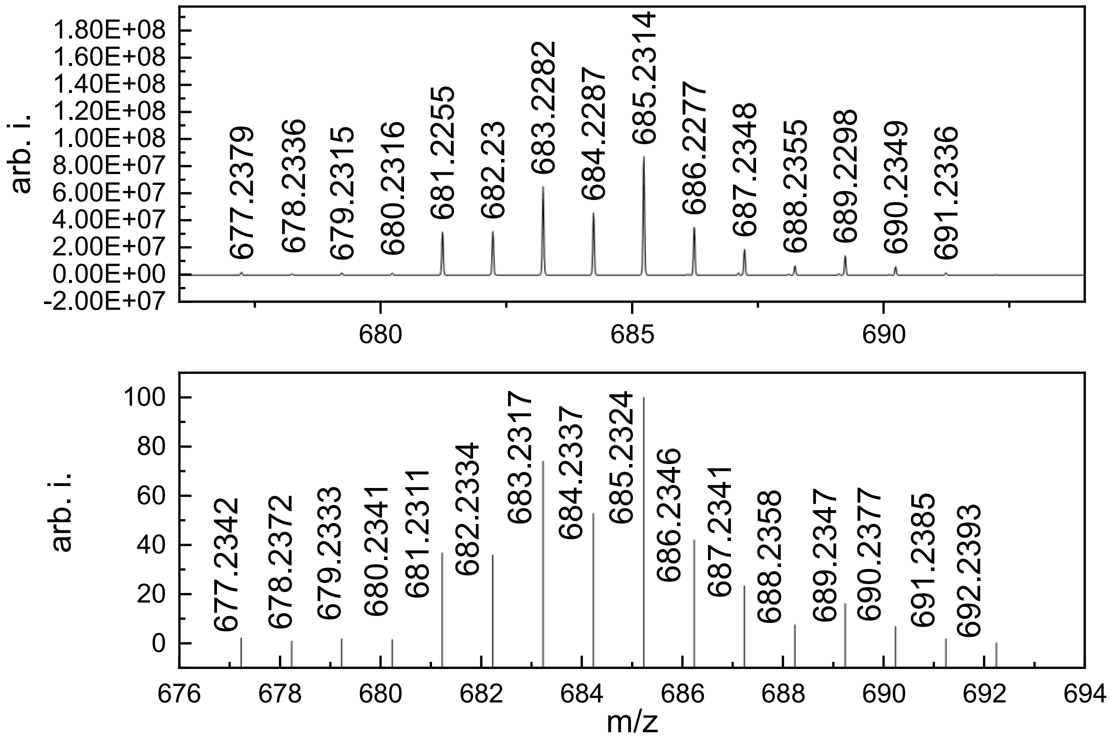

Figure S15. LIFDI-MS of **1a-Ph,** Top found MS for [^{Ph}L(Ph)Sn:]$^+$. Bottom. Calculated MS spectrum of [^{Ph}L(Ph)Sn:]$^+$.

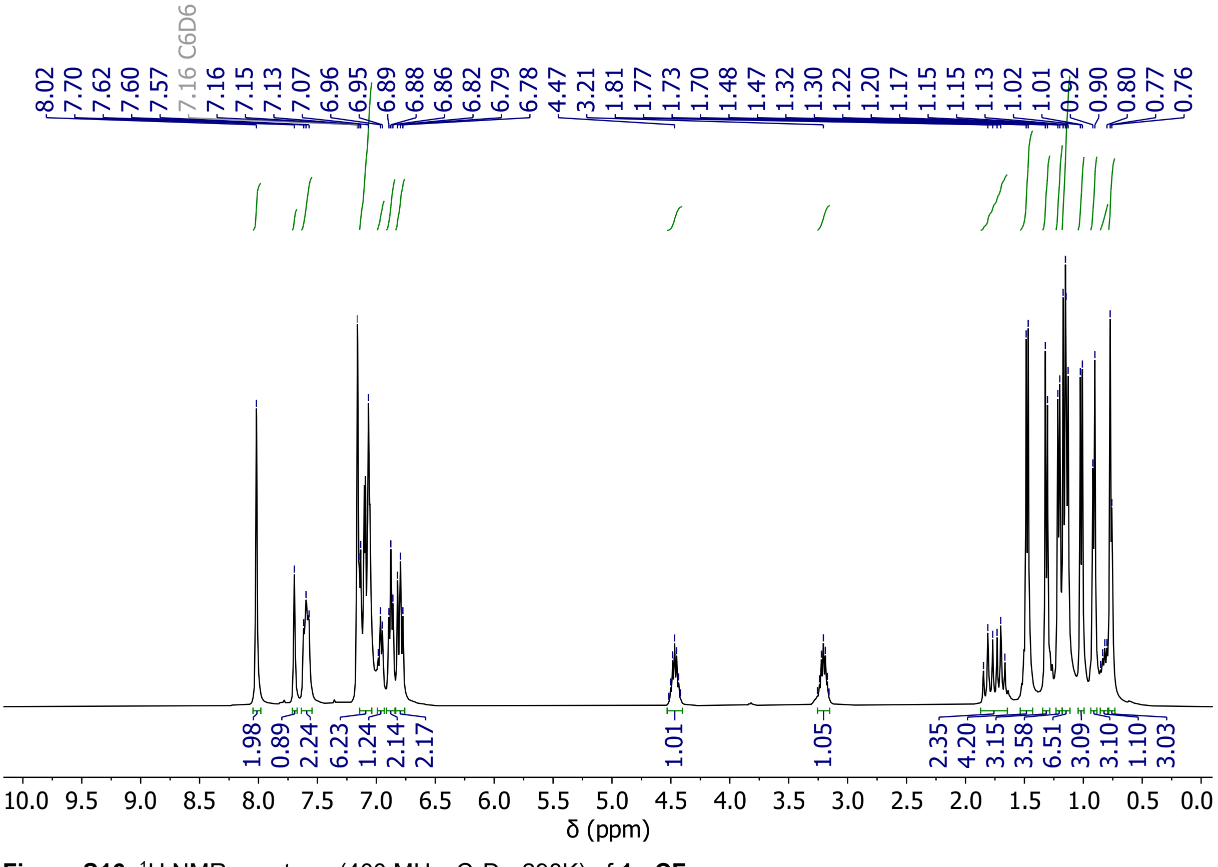

Figure S16. ^{1}H NMR spectrum (400 MHz, C_6D_6, 298K) of **1a-CF$_3$**.

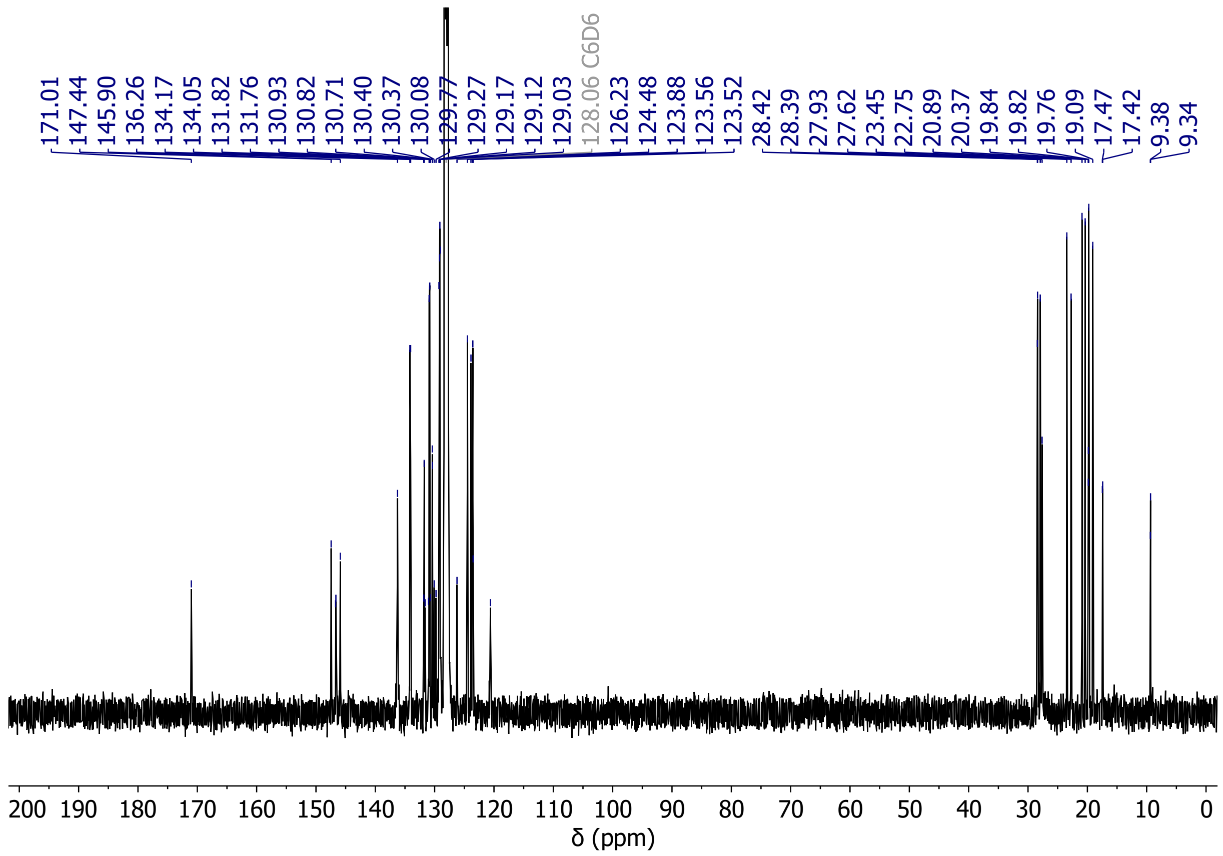

Figure S17. ^{13}C NMR spectrum (400 MHz, C_6D_6, 298K) of **1a-CF$_3$**.

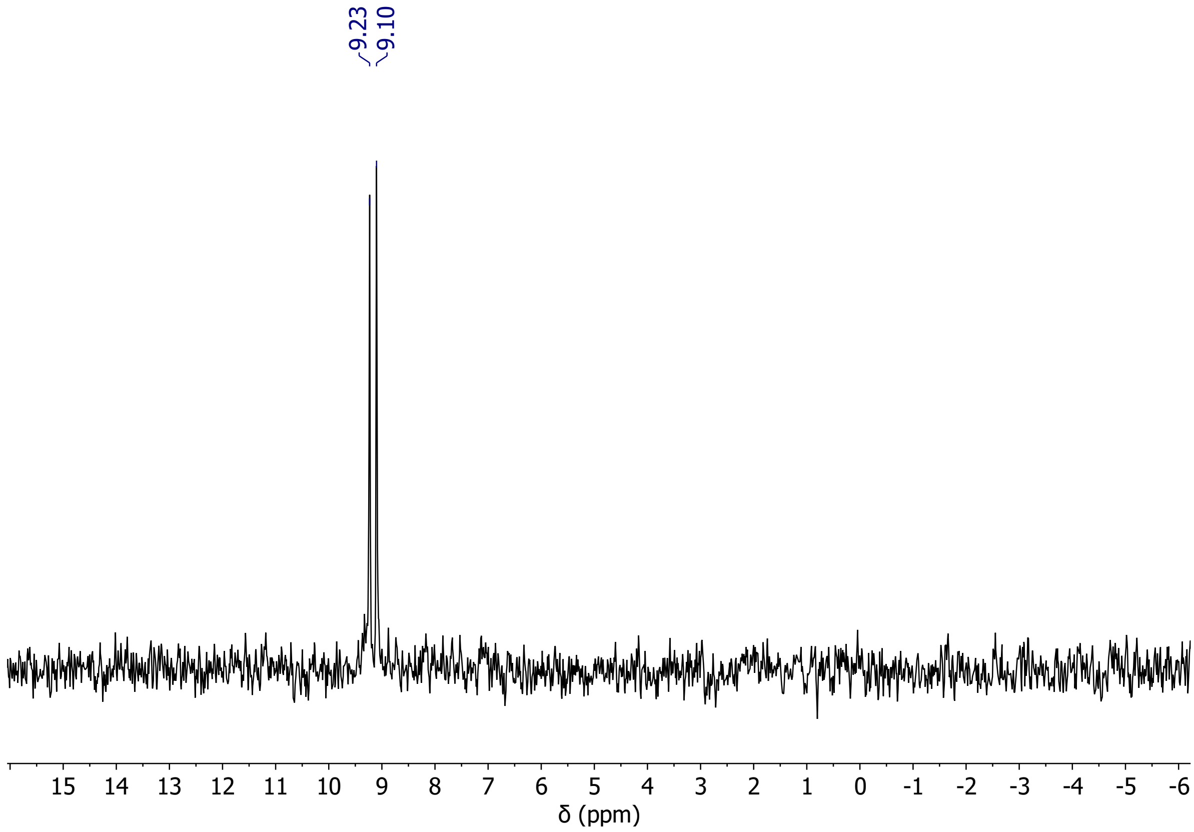

Figure S18. $^{29}Si\{^1H\}$ NMR spectrum (99 MHz, C_6D_6, 298K) of **1a-CF$_3$**.

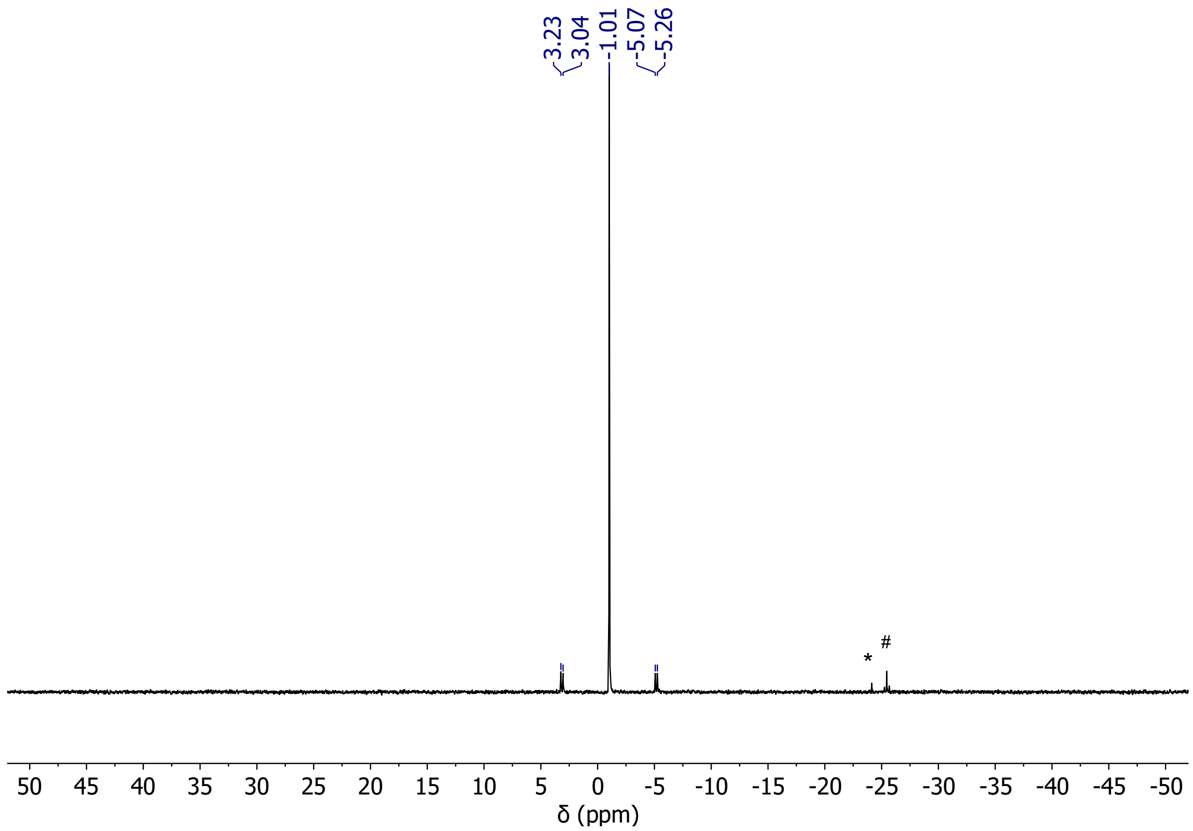

Figure S19. $^{31}P\{^{1}H\}$ NMR spectrum (162 MHz, C_6D_6, 298K) of **1a-CF₃**. * marks ^{Ph}LH, # shows unidentified impurity.

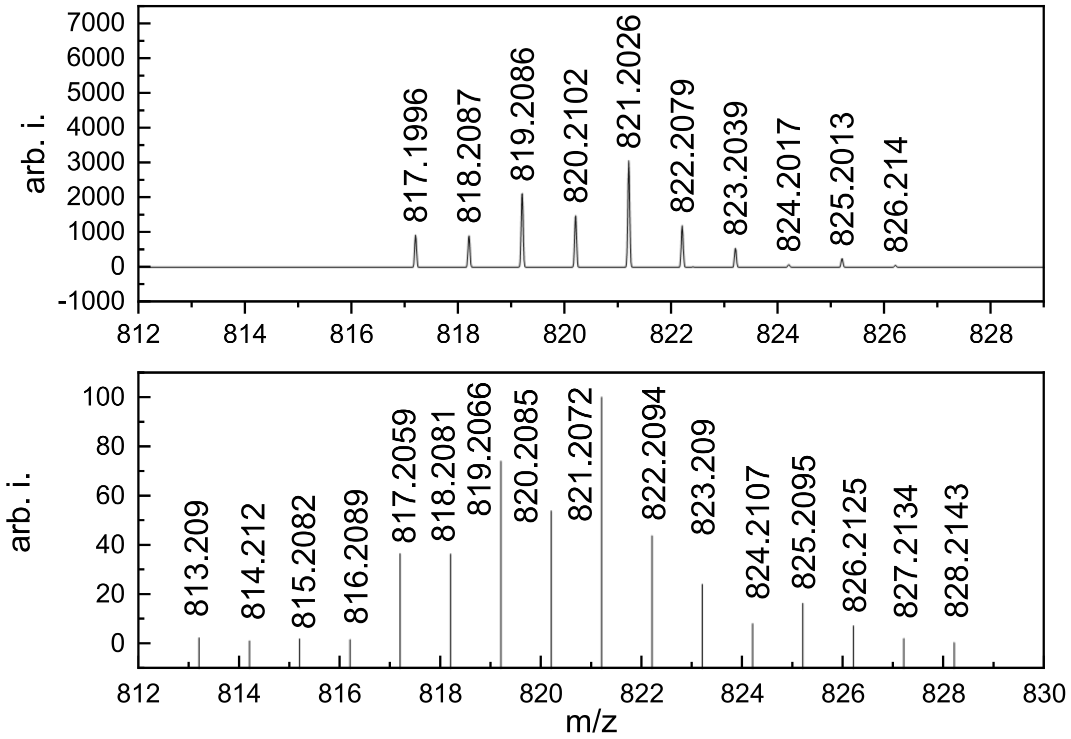

Figure S20. LIFDI-MS of **1a-CF_3,** Top found MS for $[^{Ph}LSn[m\text{-}(CF_3)_2\text{-}Ph]]^+$. Bottom. Calculated MS spectrum of $[^{Ph}L[m\text{-}(CF_3)_2\text{-}Ph]Sn:]^+$.

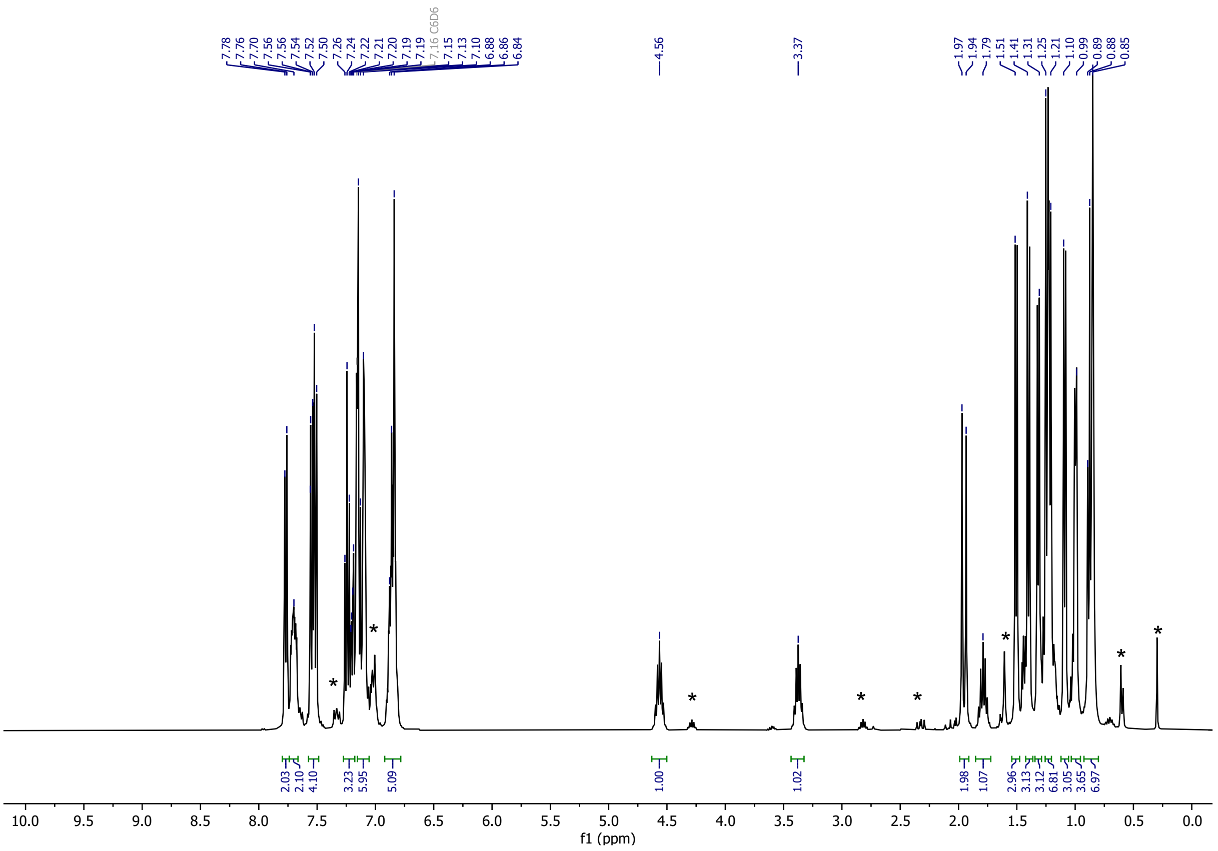

Figure S21. ^{1}H NMR spectrum (400 MHz, C_6D_6, 298K) of **1a-*p*BP**. * shows unidentified impurity.

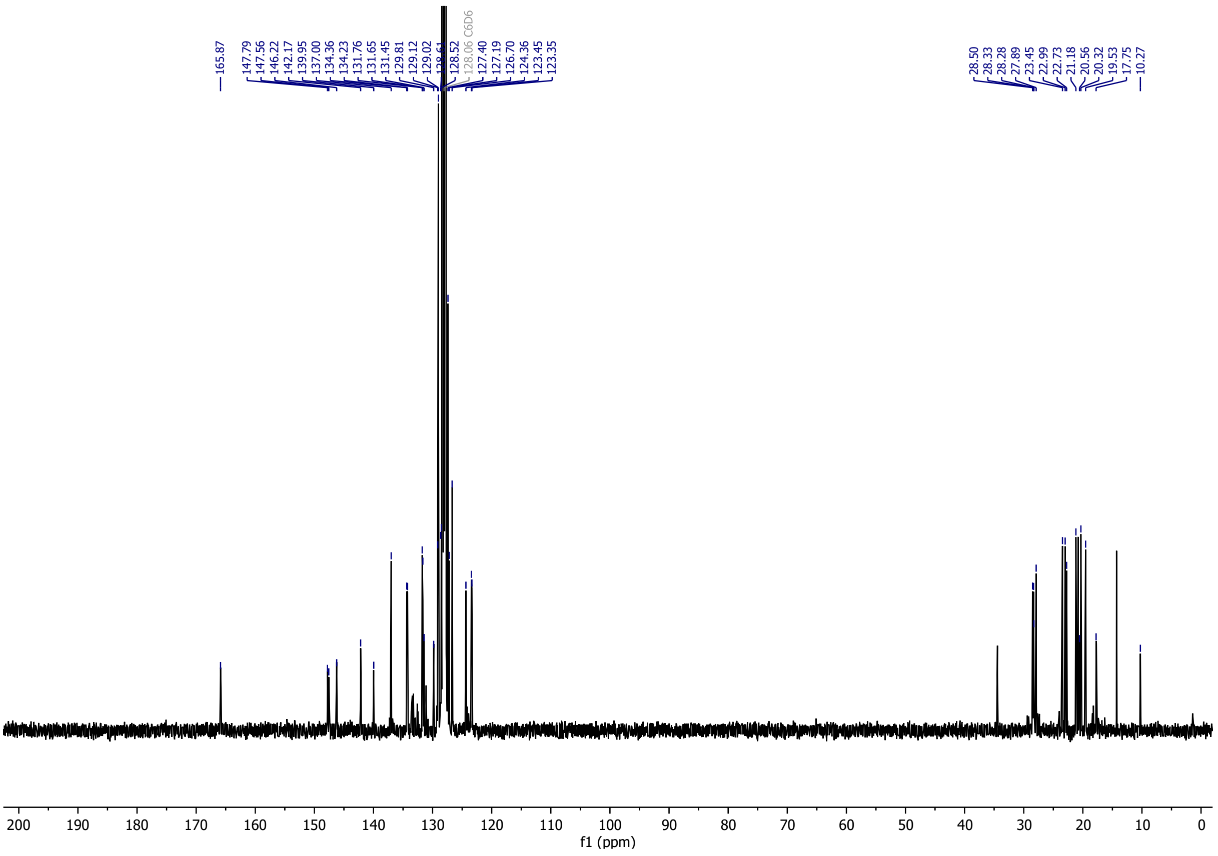

Figure S22. ^{13}C NMR spectrum (400 MHz, C_6D_6, 298K) of **1a-*p*BP**.

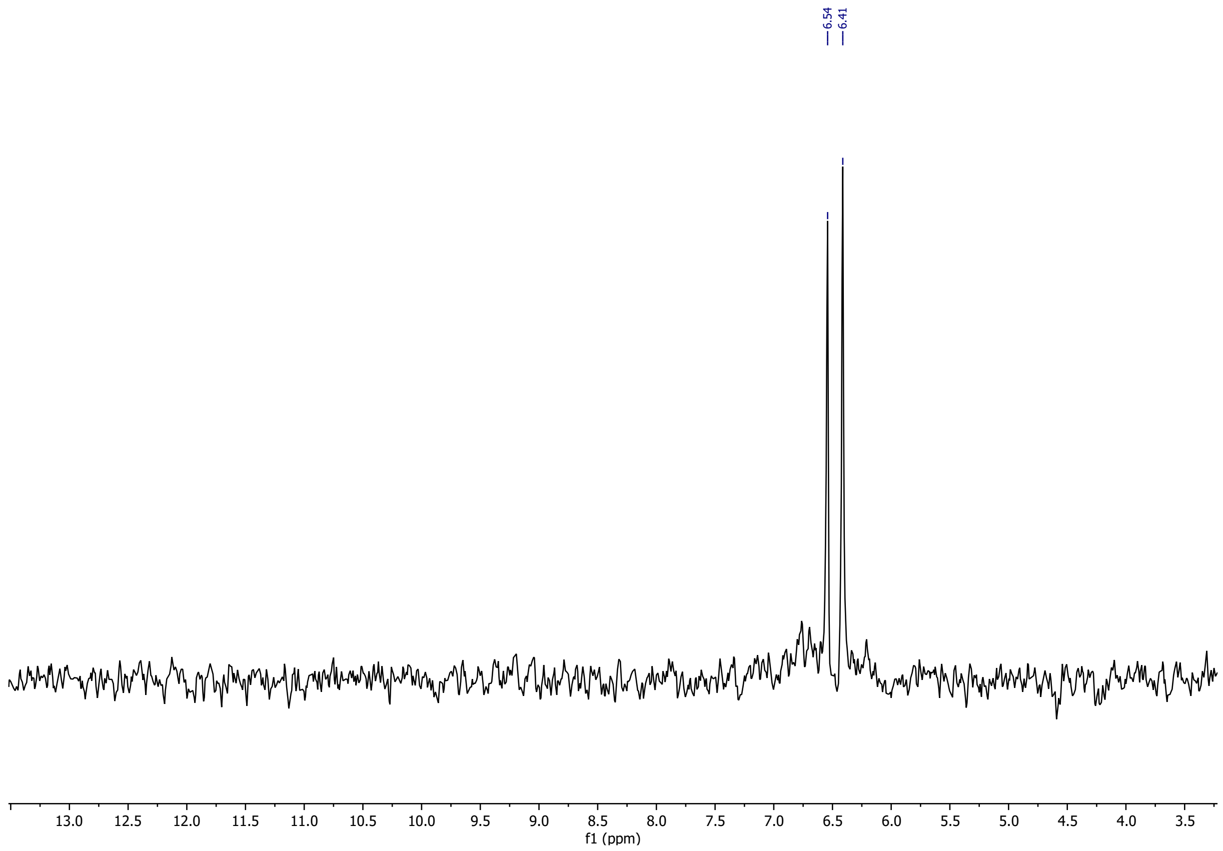

Figure S23. $^{29}Si\{^1H\}$ NMR spectrum (99 MHz, C_6D_6, 298K) of **1a-*p*BP**.

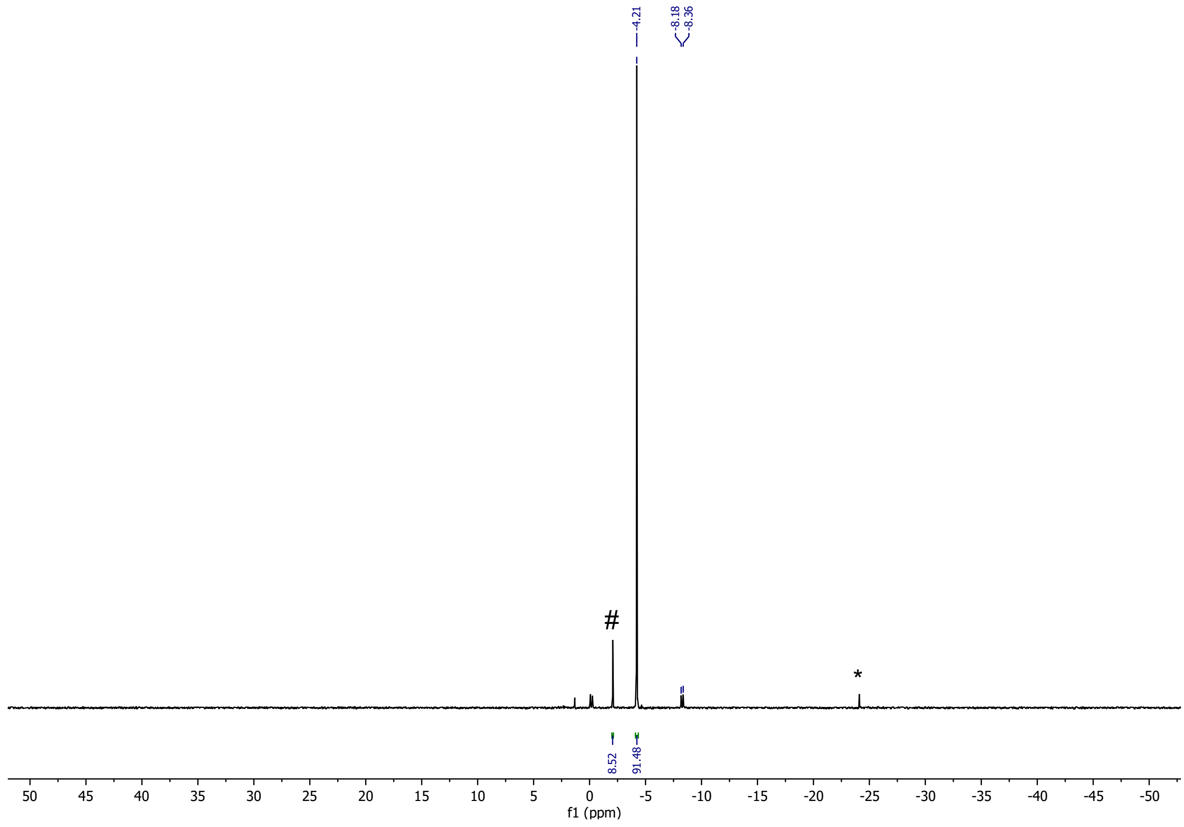

Figure S24. $^{31}P\{^1H\}$ NMR spectrum (162 MHz, C_6D_6, 298K) of **1a-pBP**. * marks ^{Ph}LH, # shows unidentified impurity.

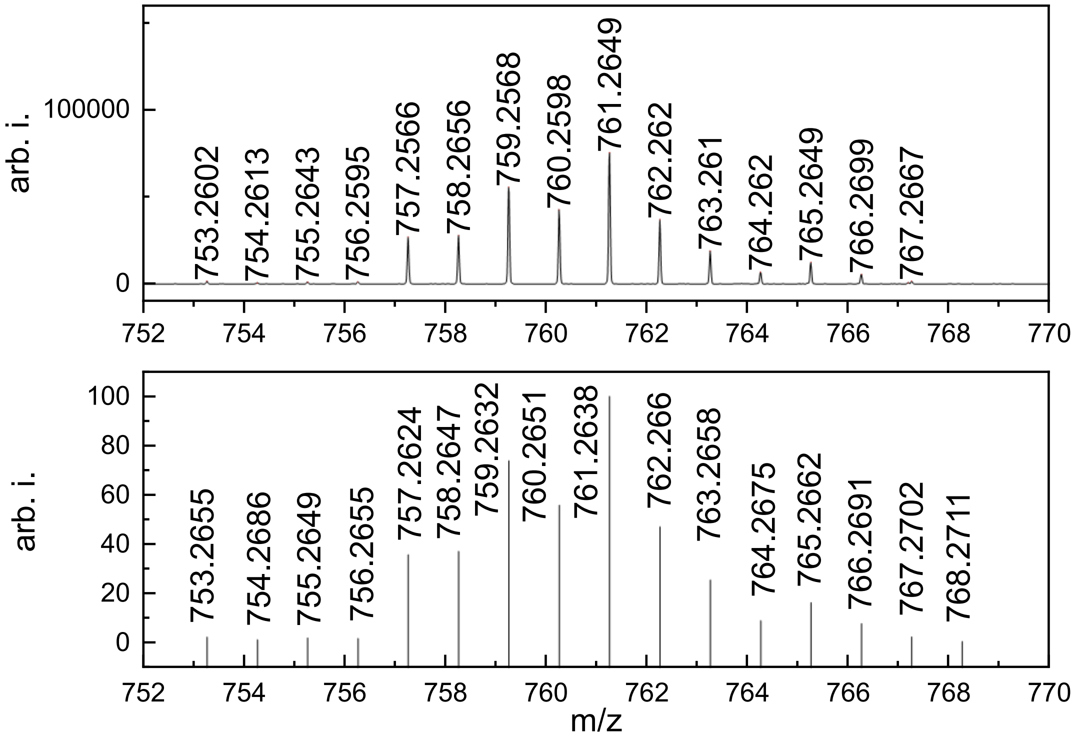

Figure S25. LIFDI-MS of **1a-*o*BP,** Top found MS for $[^{Ph}LSn(p\text{-BPh})]^+$. Bottom. Calculated MS spectrum of $[^{Ph}(p\text{-BPh})LSn:]^+$.

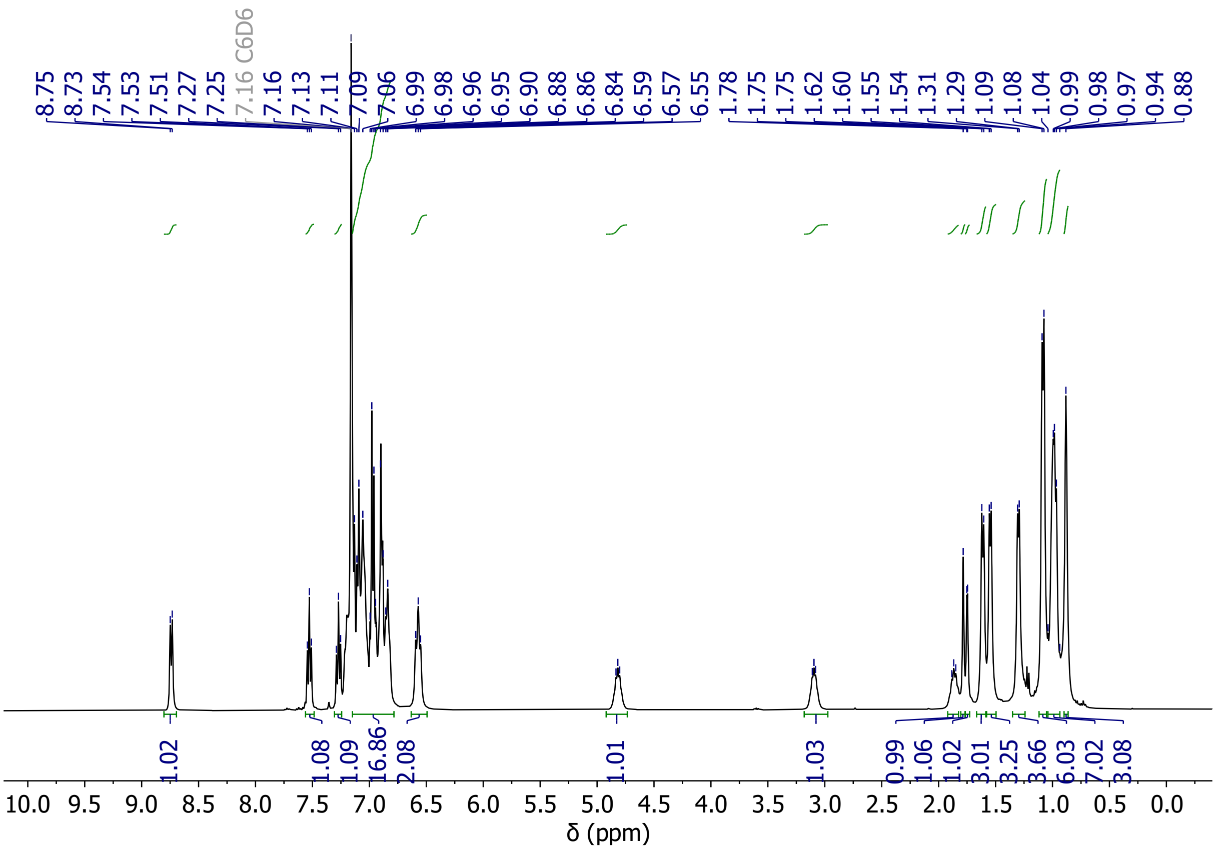

Figure S26. ^{1}H NMR spectrum (400 MHz, C_6D_6, 298K) of **1a-*o*BP**.

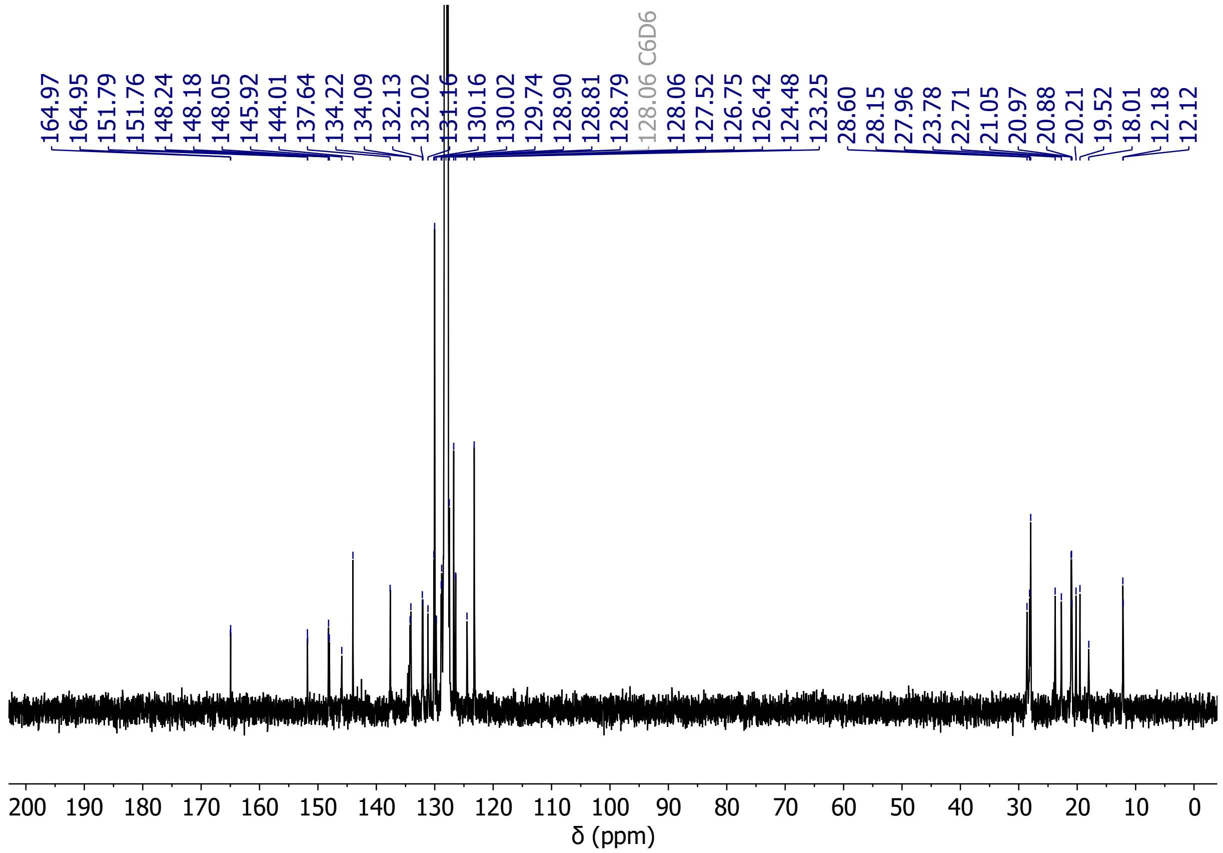

Figure S27. ^{13}C NMR spectrum (400 MHz, C_6D_6, 298K) of **1a-*o*BP**.

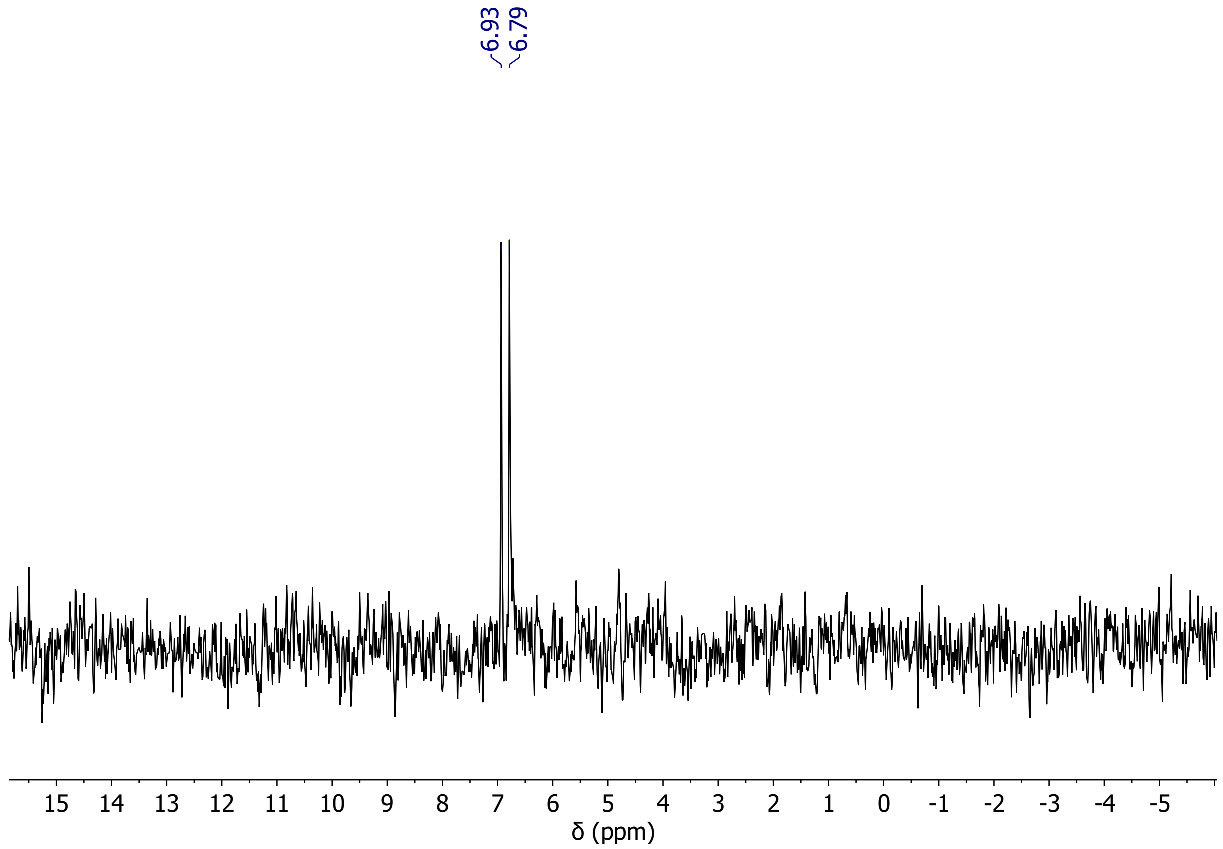

Figure S28. $^{29}Si\{^1H\}$ NMR spectrum (99 MHz, C_6D_6, 298K) of **1a-*o*BP**.

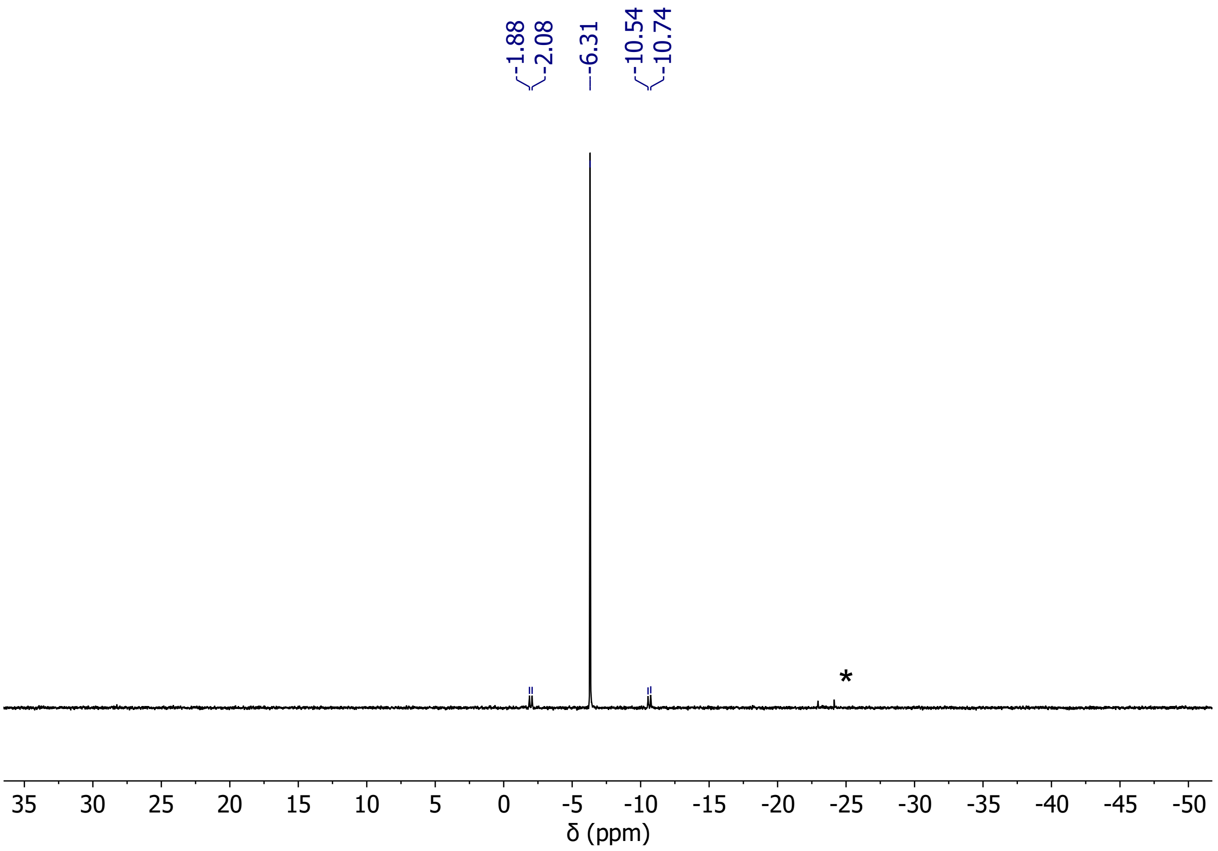

Figure S29. $^{31}P\{^1H\}$ NMR spectrum (162 MHz, C_6D_6, 298K) of **1a-*o*BP**. * marks ^{Ph}LH.

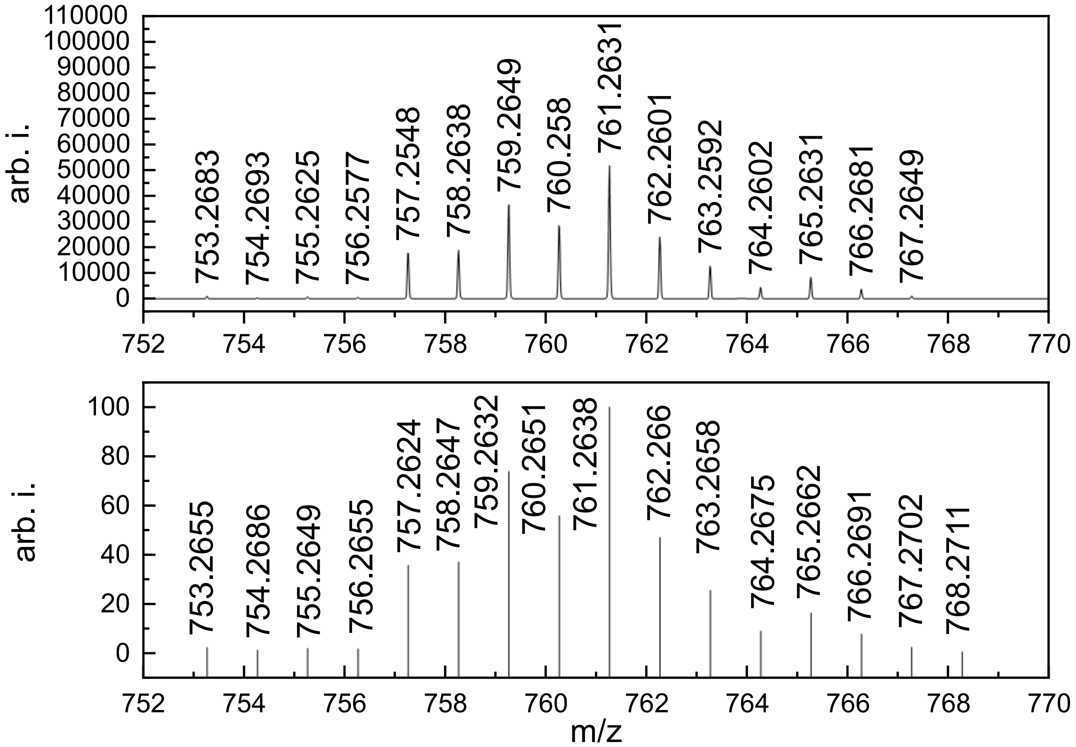

Figure S30. LIFDI-MS of **1a-*o*BP,** Top found MS for [^{Ph}L(o-BPh)Sn]$^+$. Bottom. Calculated MS spectrum of [^{Ph}L(o-BPh)Sn]$^+$.

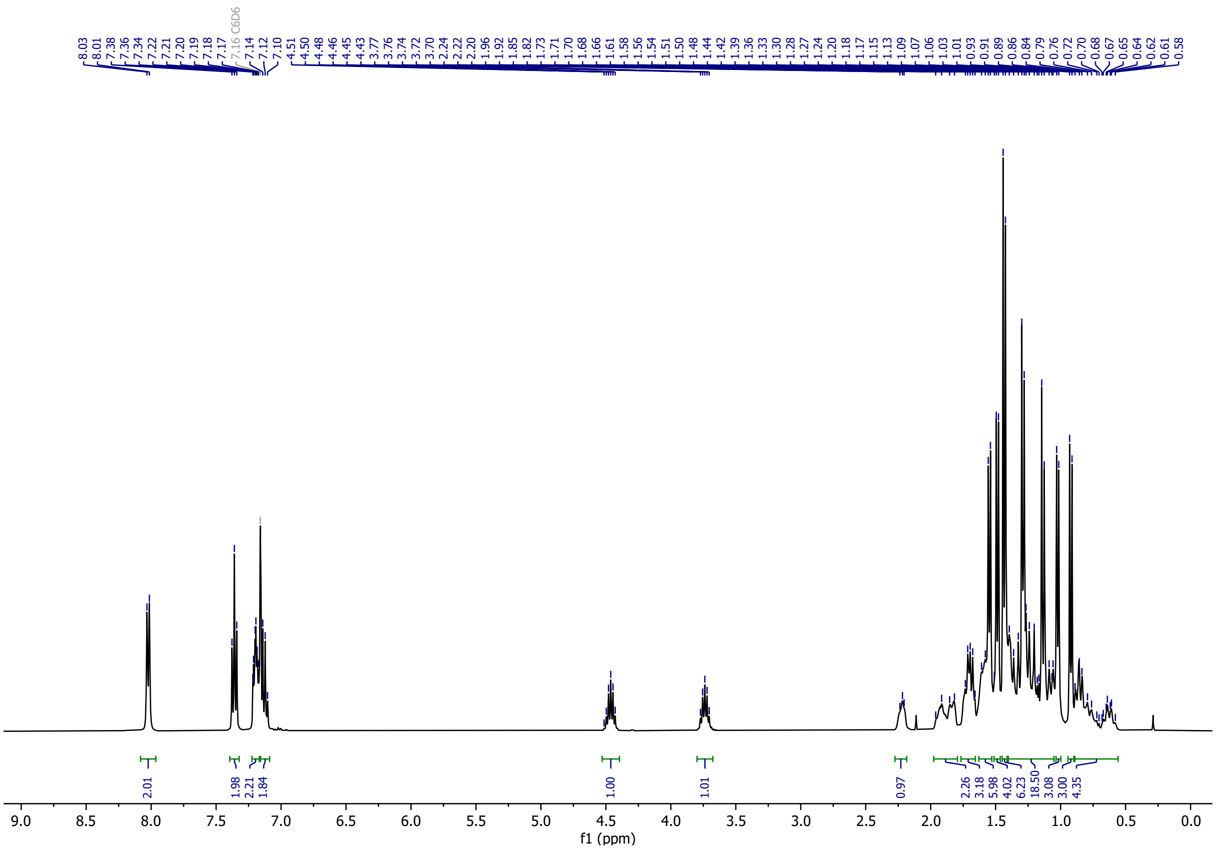

Figure S31. ^{1}H NMR spectrum (400 MHz, C_6D_6, 298K) of **1b-Ph**.

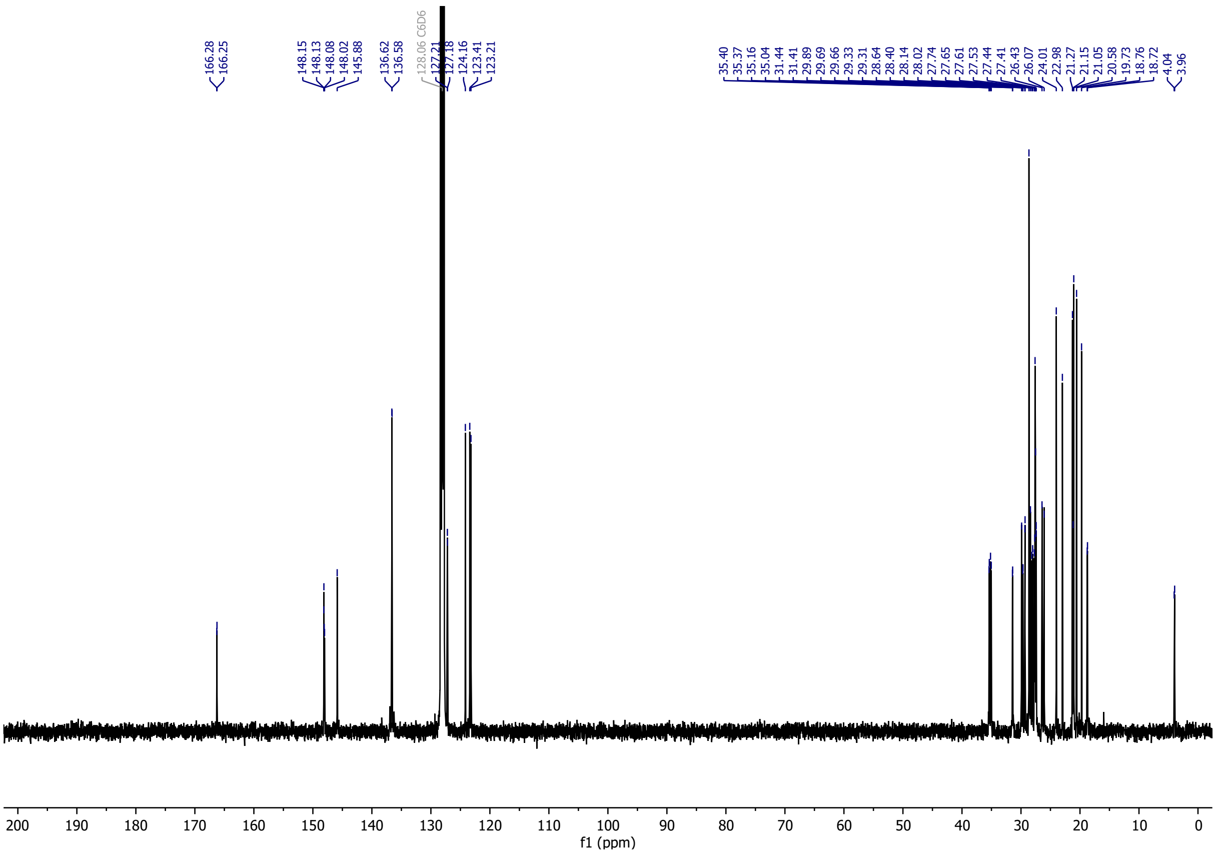

Figure S32. ^{13}C NMR spectrum (400 MHz, C_6D_6, 298K) of **1b-Ph**.

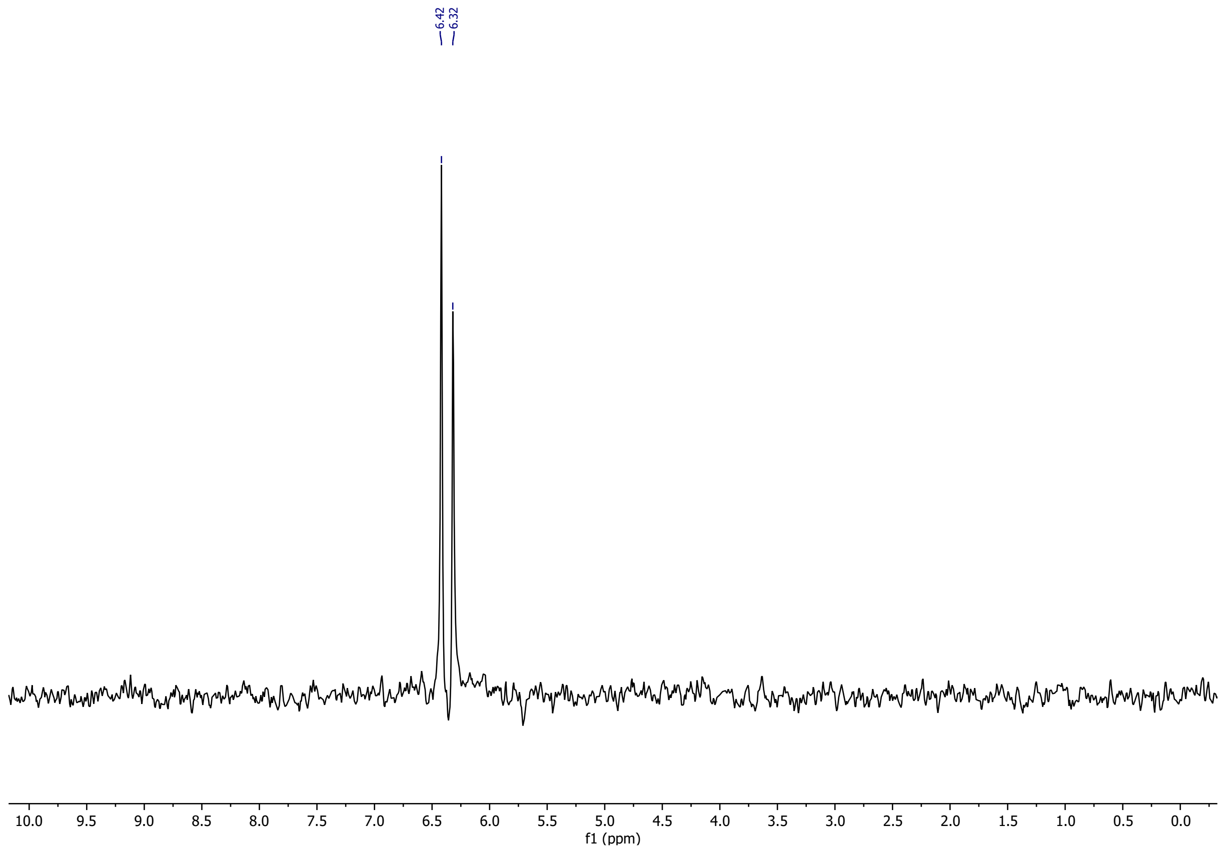

Figure S33. $^{29}Si\{^1H\}$ NMR spectrum (99 MHz, C_6D_6, 298K) of **1b-Ph**.

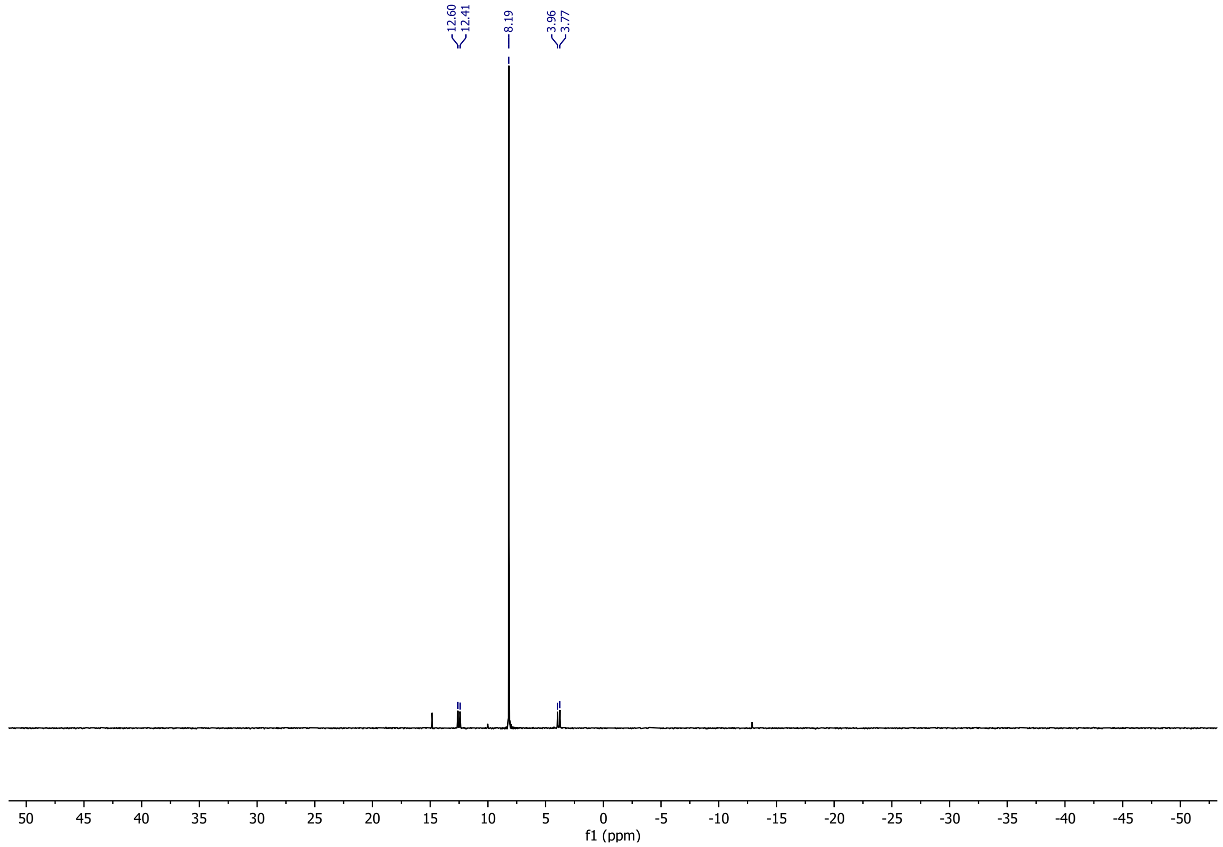

Figure S34. $^{31}P\{^1H\}$ NMR spectrum (162 MHz, C_6D_6, 298K) of **1b-Ph**.

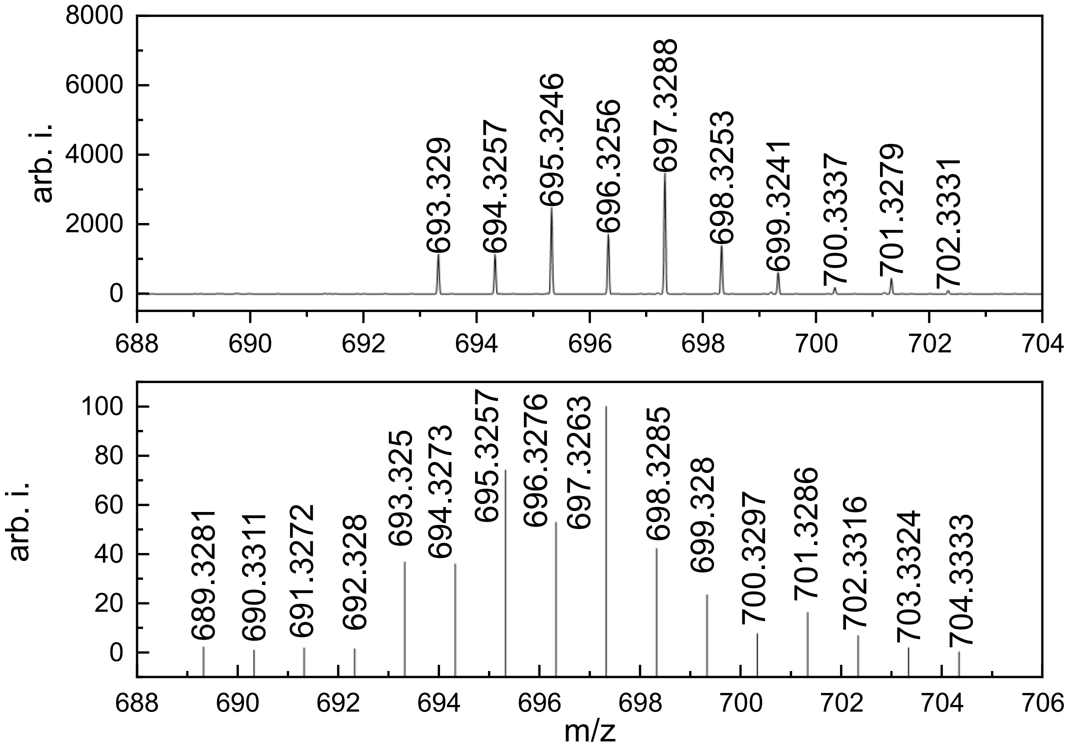

Figure S35. LIFDI-MS of **1b-Ph,** Top found MS for [^{Ph}L(Ph)Sn:]$^+$. Bottom. Calculated MS spectrum of [^{Ph}L(Ph)Sn:]$^+$.

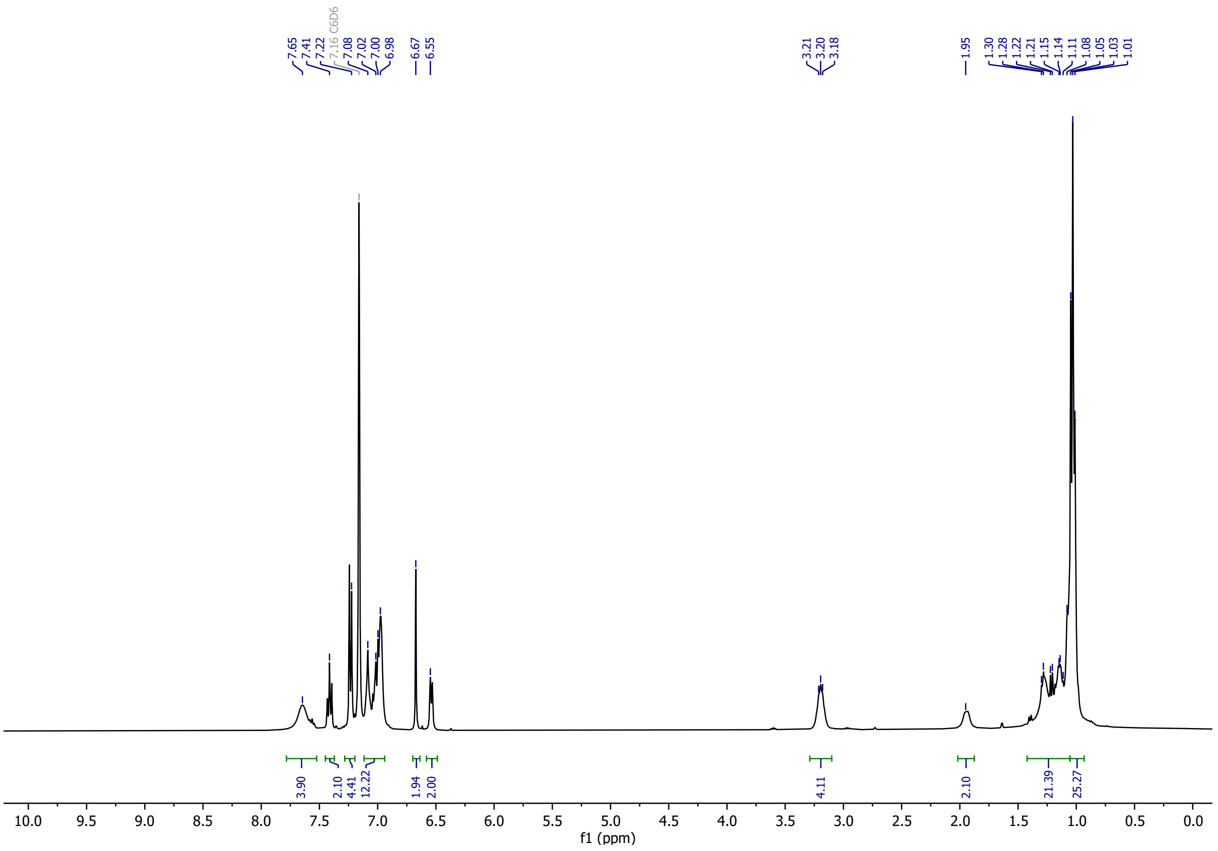

Figure S36. ^{1}H NMR spectrum (400 MHz, C_6D_6, 298K) of **2-Ph**.

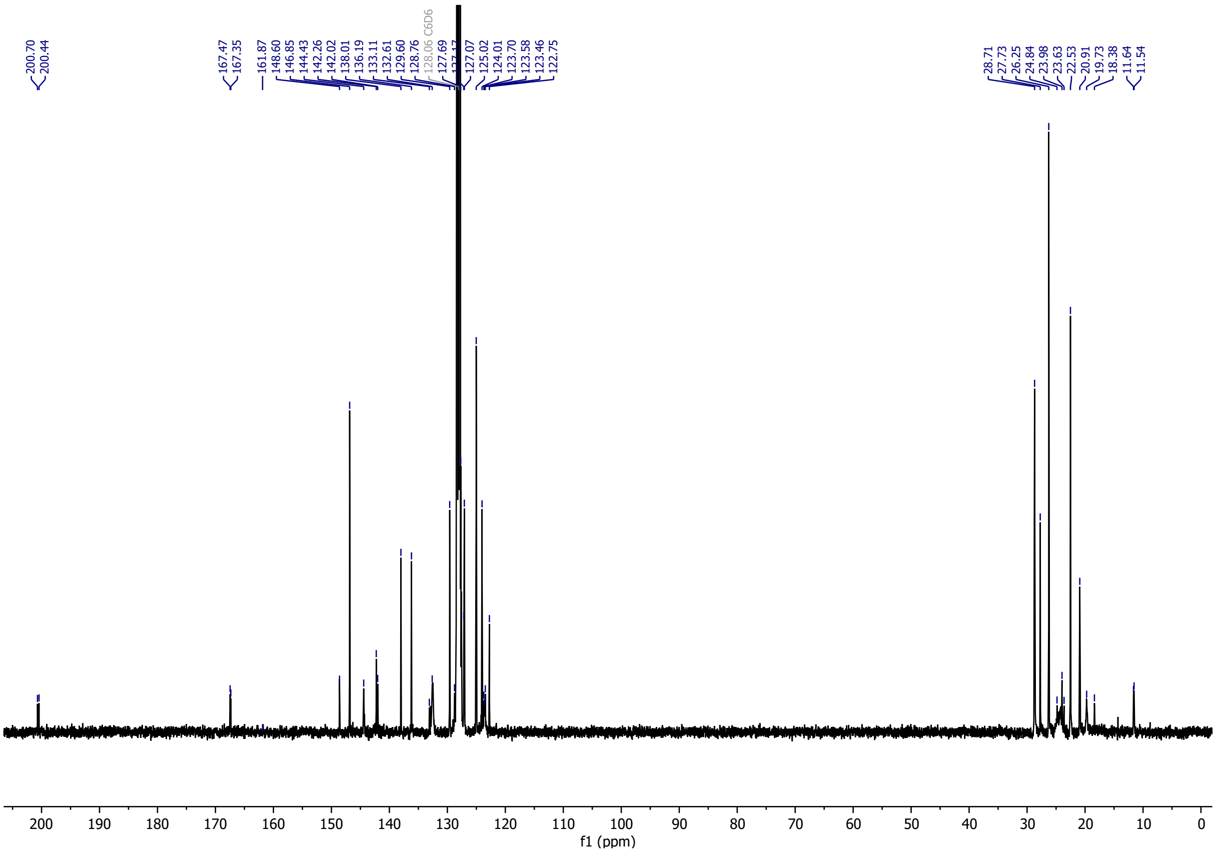

Figure S37. ^{13}C NMR spectrum (400 MHz, C_6D_6, 298K) of **2-Ph**.

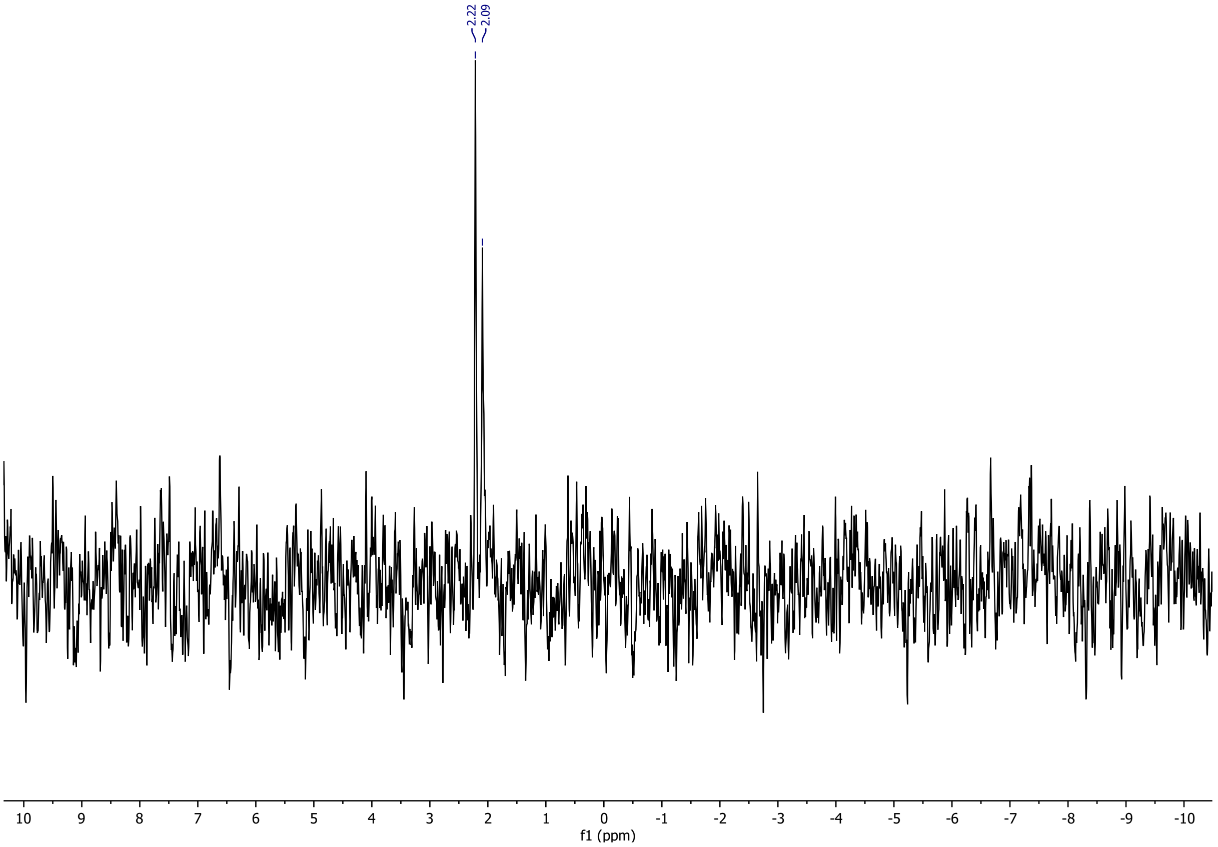

Figure S38. $^{29}Si\{^1H\}$ NMR spectrum (99 MHz, C_6D_6, 298K) of **2-Ph**.

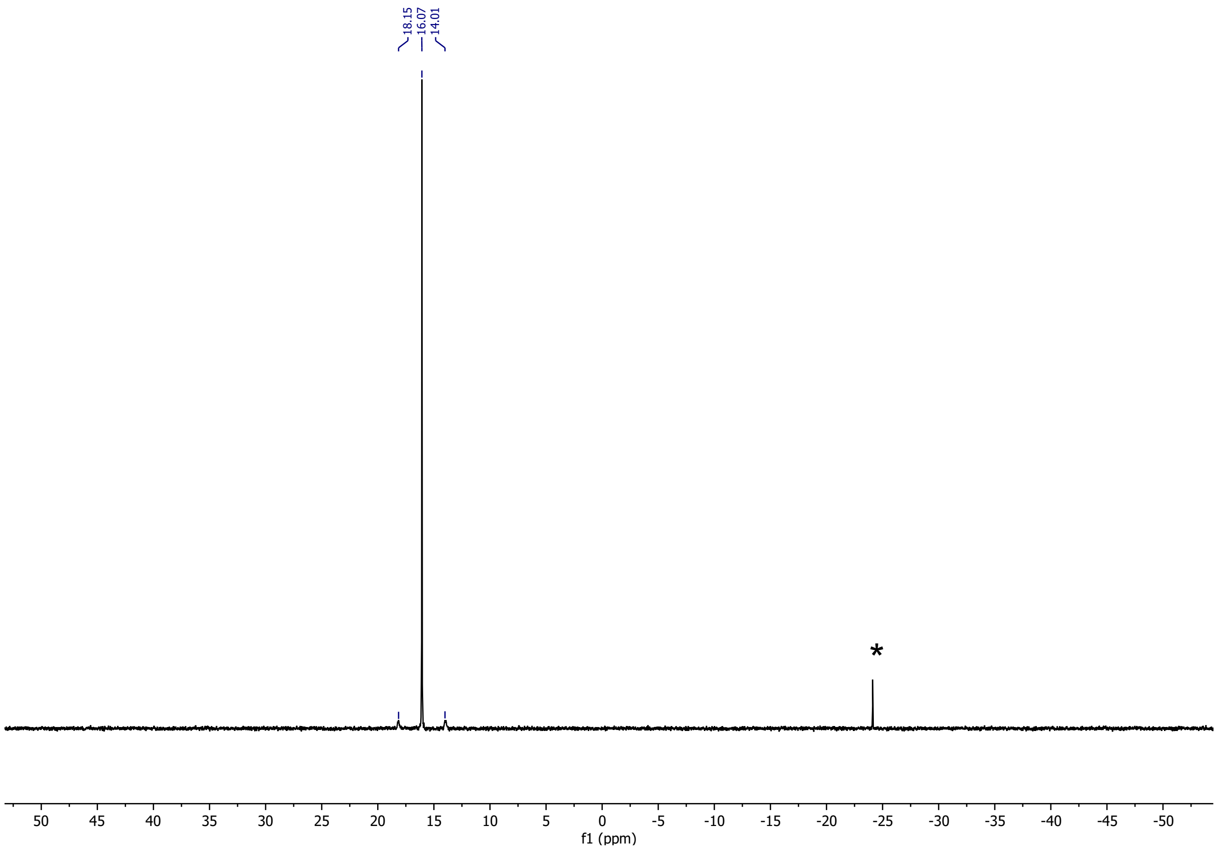

Figure S39. $^{31}P\{^{1}H\}$ NMR spectrum (162 MHz, C_6D_6, 298K) of **2-Ph**. * marks ^{Ph}LH.

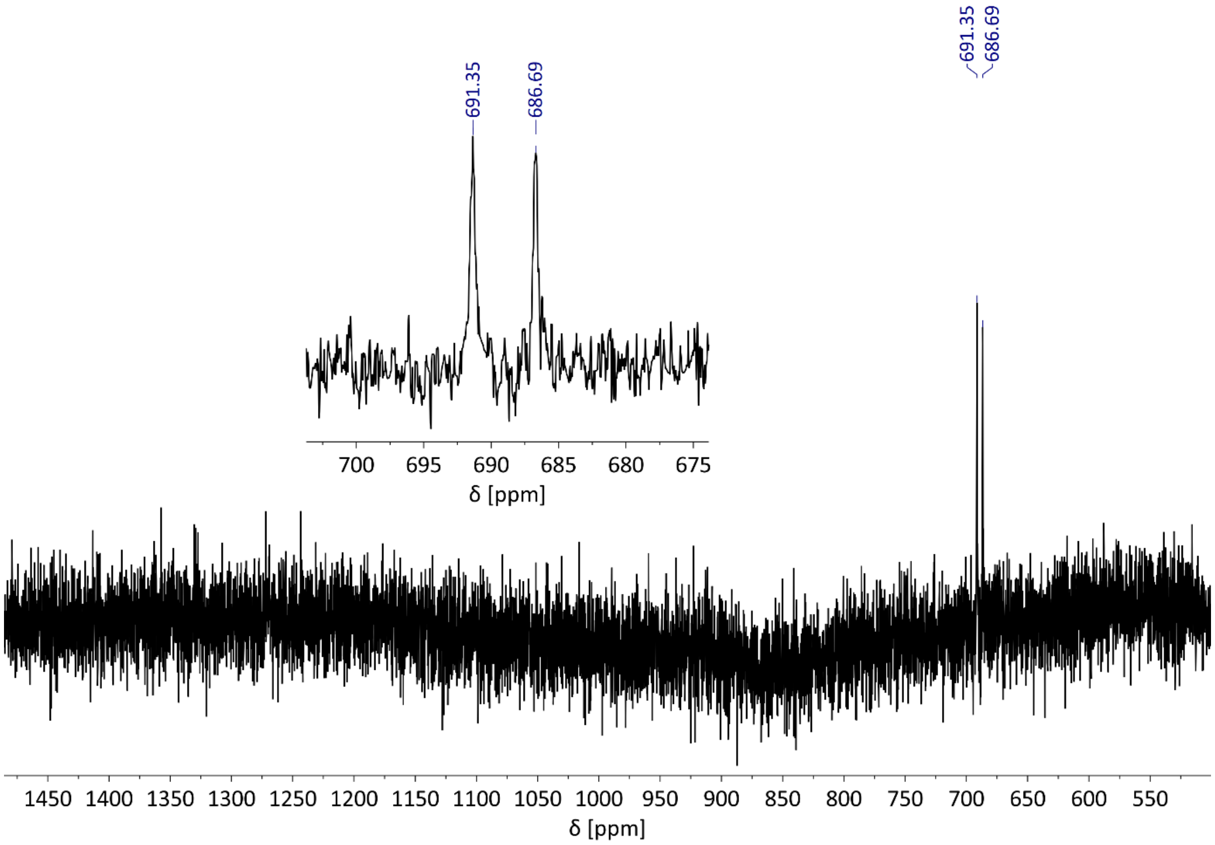

Figure S40. $^{119}Sn\{^{1}H\}$ NMR spectrum (C_6D_6, 298K) of **2-Ph**.

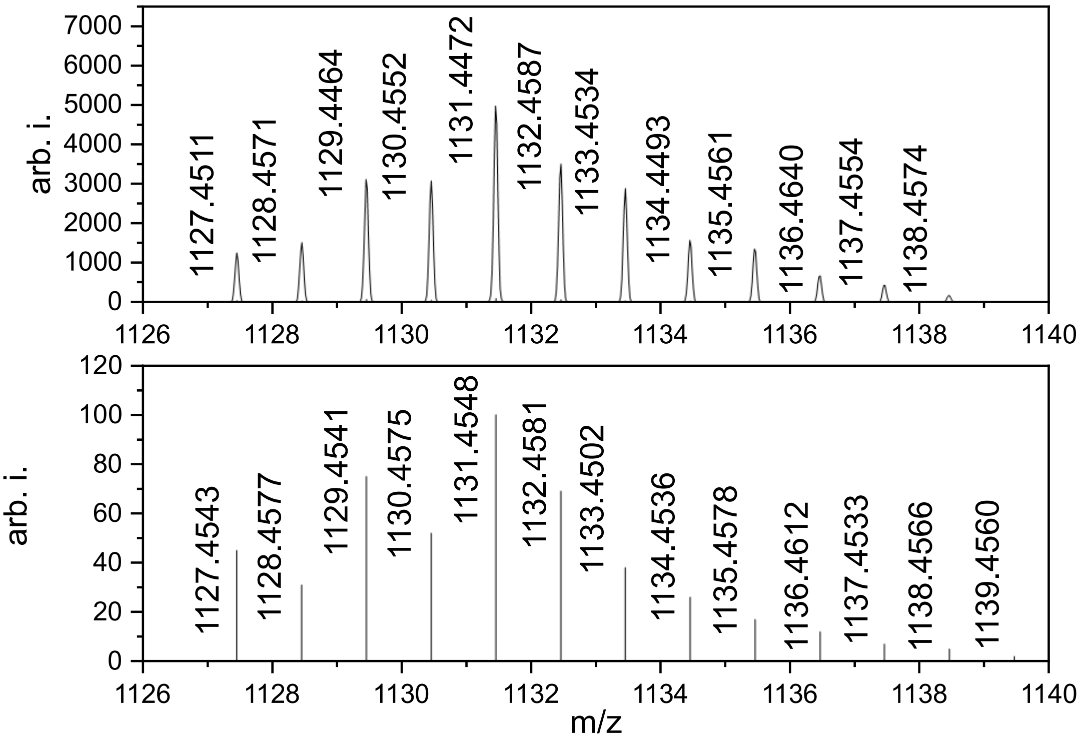

Figure S41. LIFDI-MS of **2-Ph,** Top found MS for [{^{Ph}L(Ph)Sn}·Ni·IPr]$^{+}$ Bottom. Calculated MS spectrum of [{^{Ph}L(Ph)Sn}·Ni·IPr]$^{+}$.

Figure S42. UV-Vis spectrum of **2-Ph** in toluene (1.0x10^{-4} M) at ambient temperature.

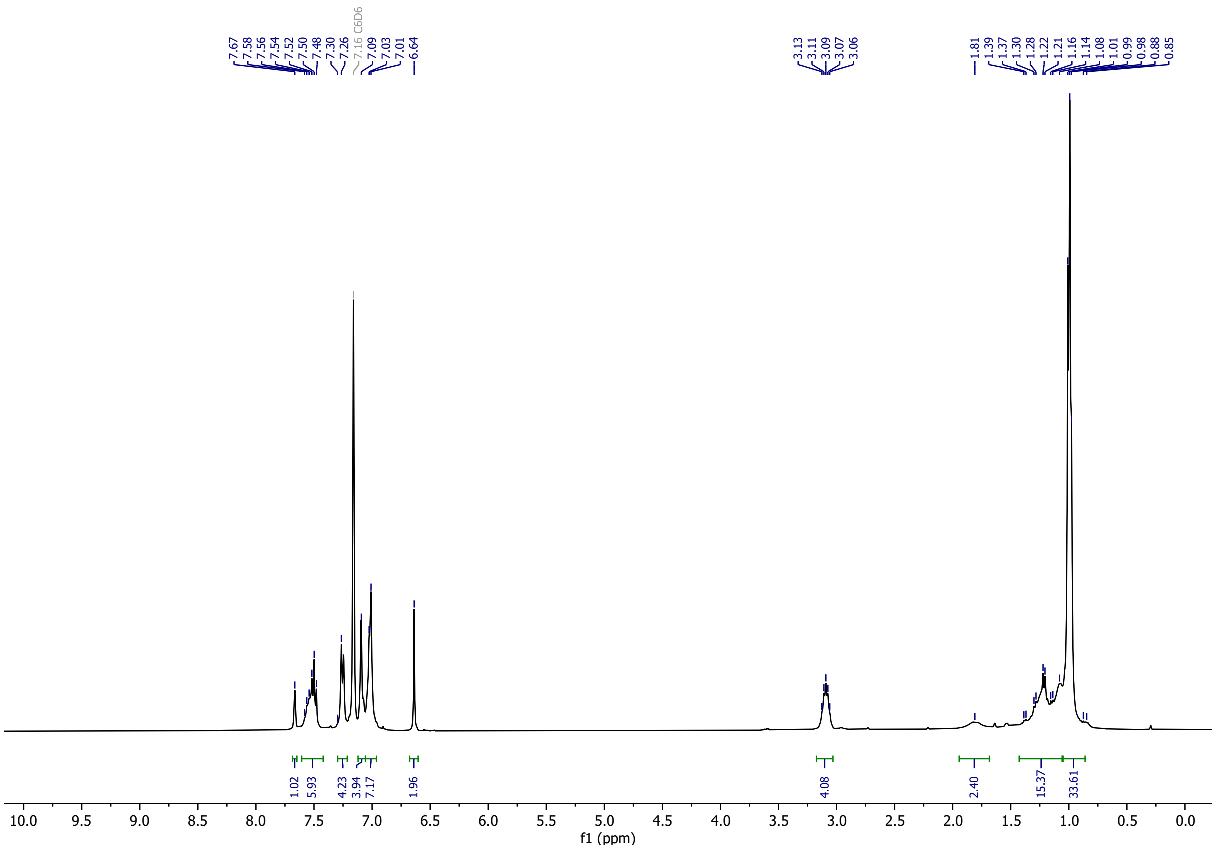

Figure S43. ^{1}H NMR spectrum (400 MHz, C_6D_6, 298K) of **2-CF₃**.

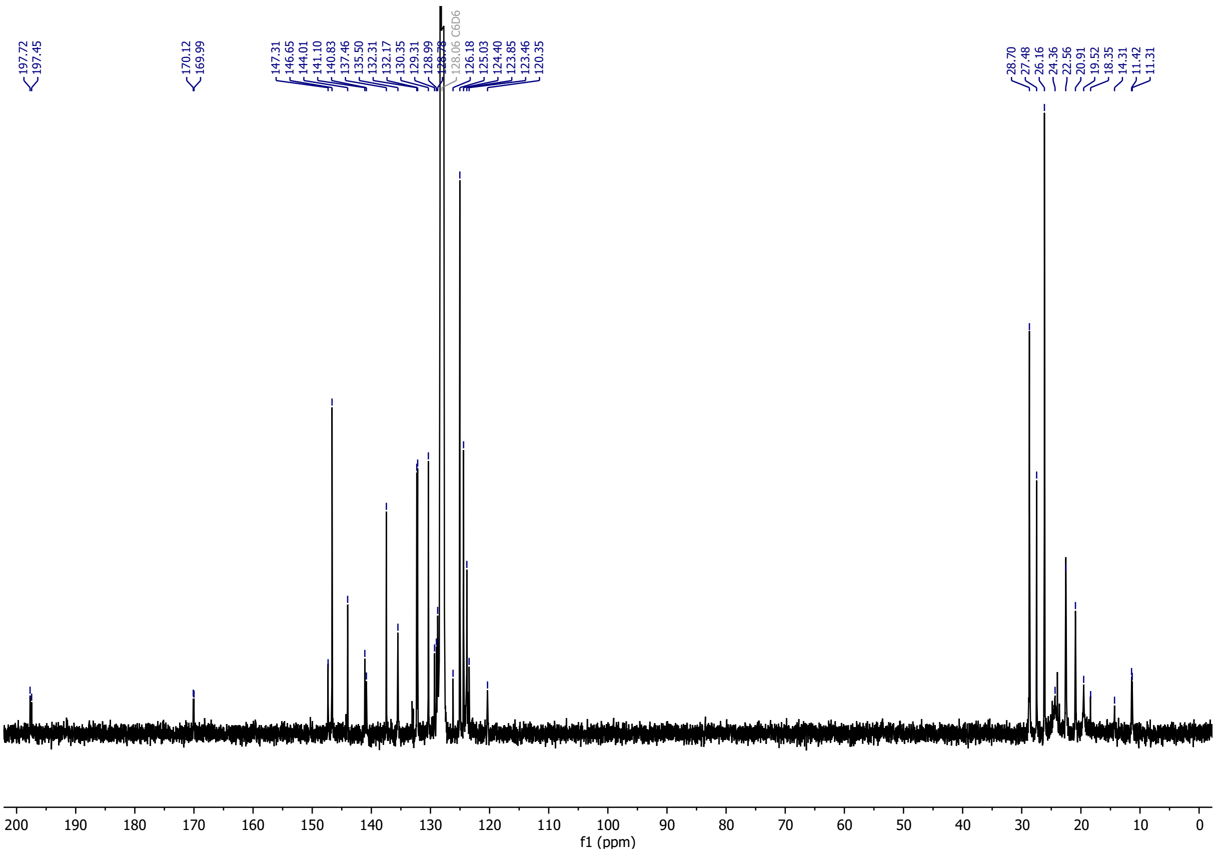

Figure S44. ^{13}C NMR spectrum (400 MHz, C_6D_6, 298K) of **2-CF₃**.

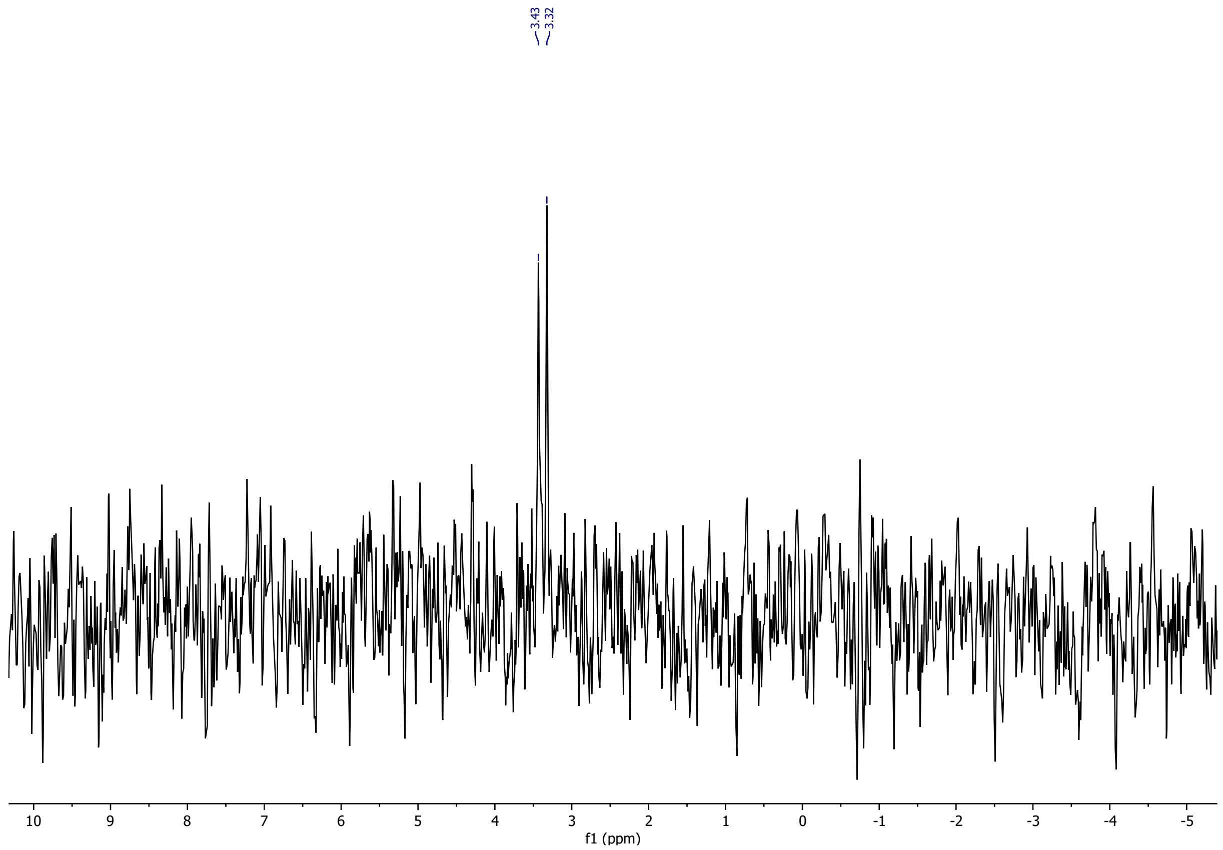

Figure S45. $^{29}Si\{^1H\}$ NMR spectrum (99 MHz, C_6D_6, 298K) of **2-CF₃**.

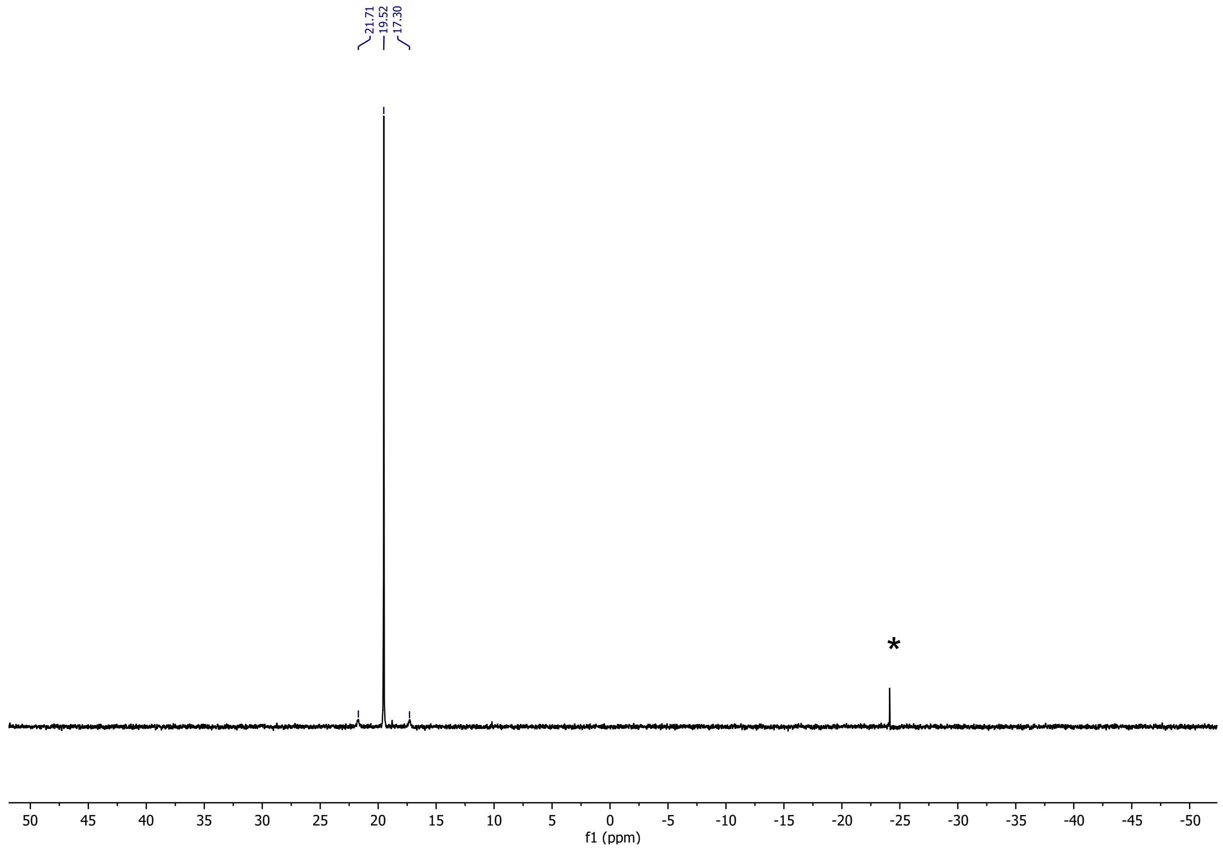

Figure S46. $^{31}P\{^1H\}$ NMR spectrum (162 MHz, C_6D_6, 298K) of **2-CF$_3$**. * marks ^{Ph}LH.

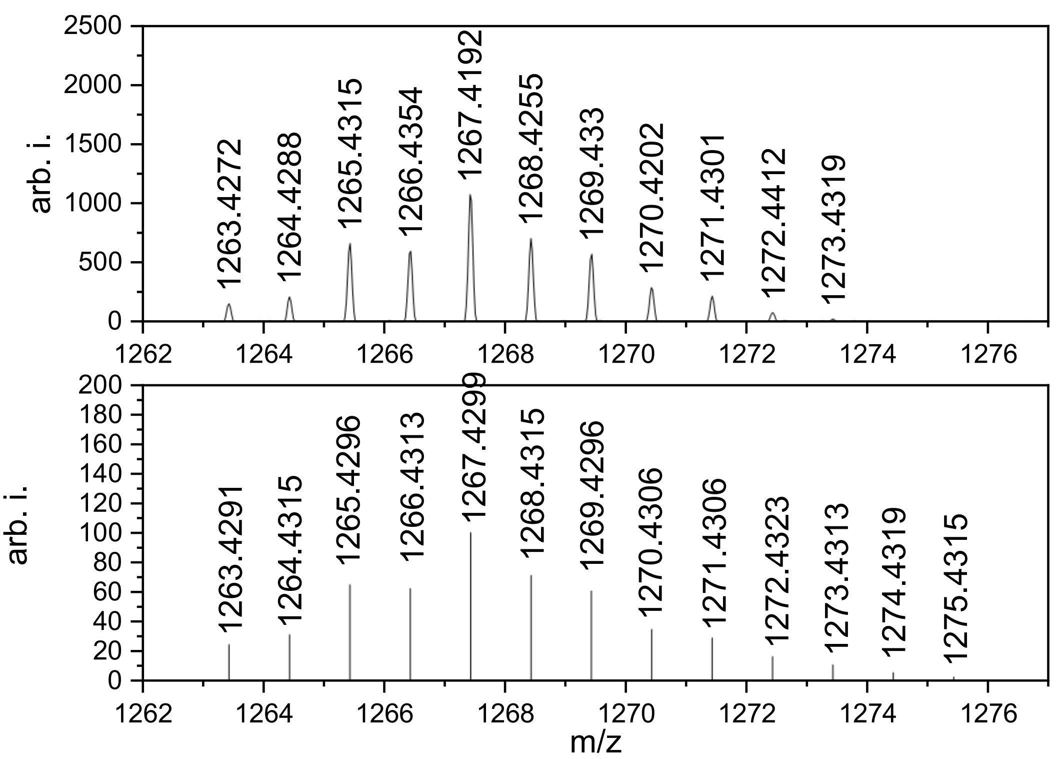

Figure S47. LIFDI-MS of **2-CF₃,** Top found MS for [^{Ph}L(m-$(CF_3)_2$-Ph)Sn·Ni·IPr]$^+$ Bottom Calculated MS spectrum of [^{Ph}L(m-$(CF_3)_2$-Ph)Sn·Ni·IPr]$^+$.

Figure S48. UV-Vis spectrum of **2-CF_3** in toluene (1.0x10^{-4} M) at ambient temperature.

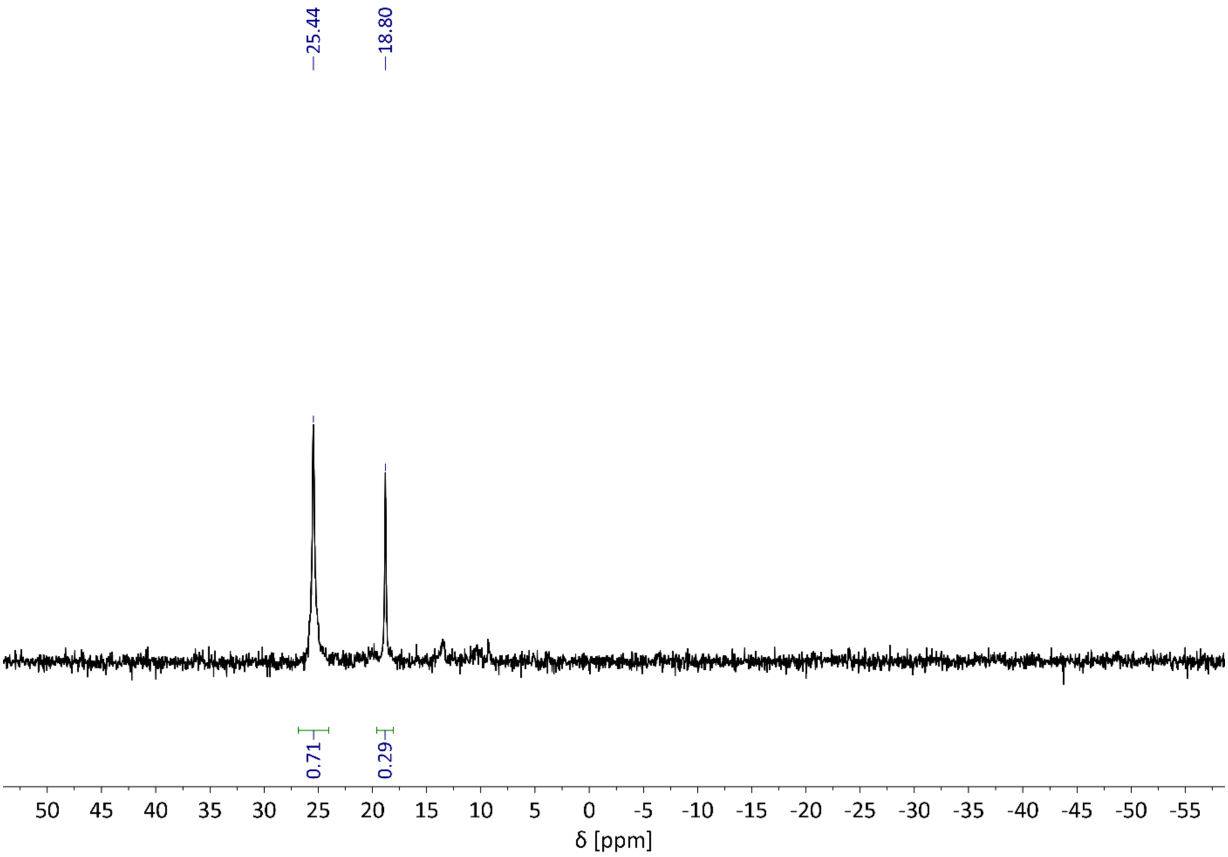

Figure S49. ^{31}P NMR spectrum for the reaction between a mixture of **1-CF$_3$**, IPr, and Ni(cod)$_2$, leading to a ~2:1 mixture of **3** (25.4 ppm) and **2-CF$_3$** (18.8 ppm).

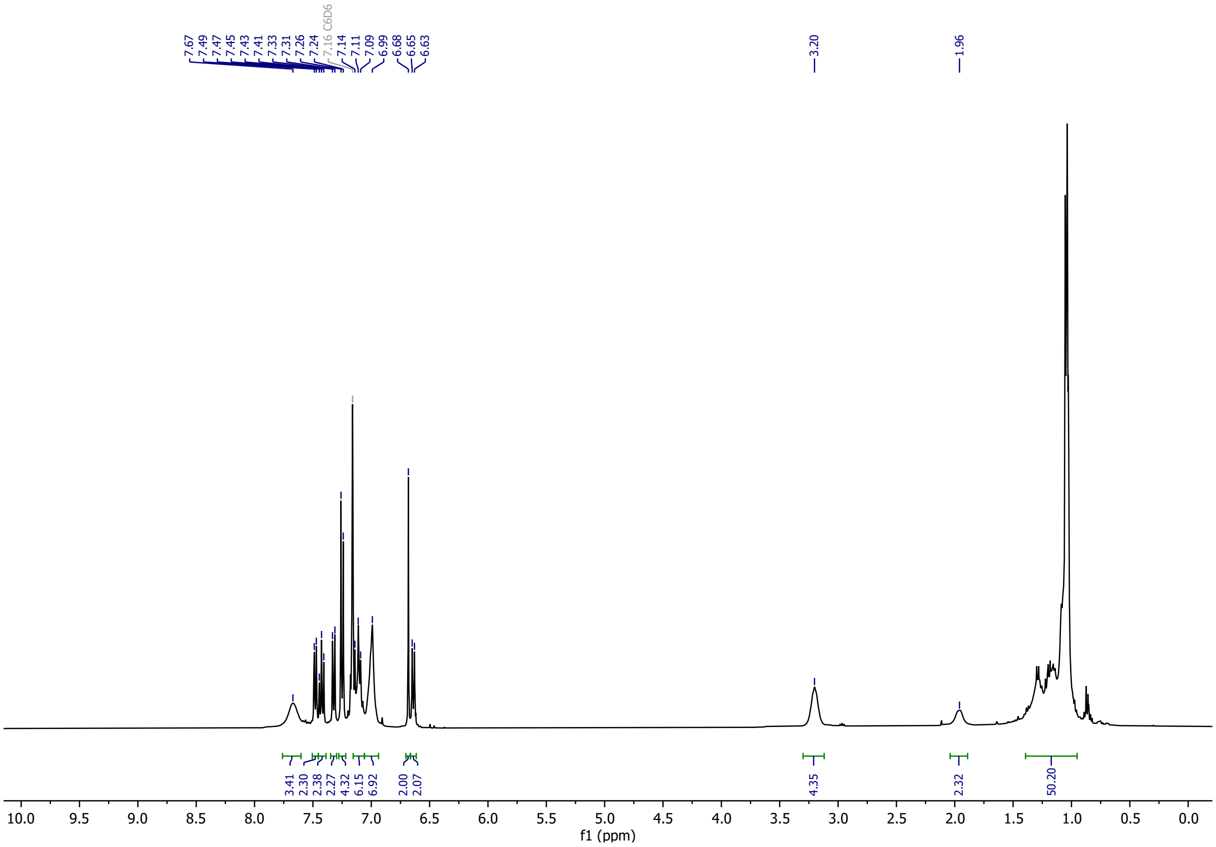

Figure S50. ^{1}H NMR spectrum (400 MHz, C_6D_6, 298K) of ***2-pBP***.

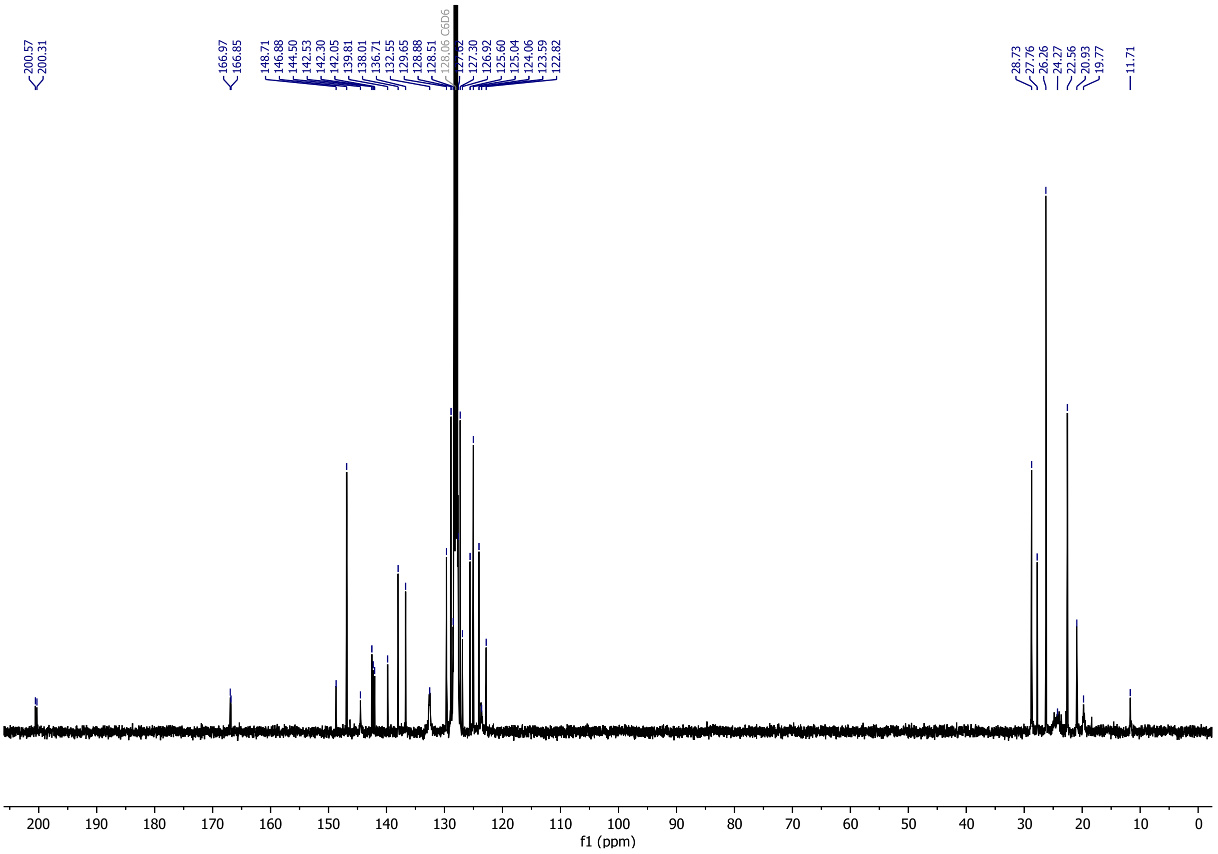

Figure S51. ^{13}C NMR spectrum (400 MHz, C_6D_6, 298K) of ***2*-p*BP***.

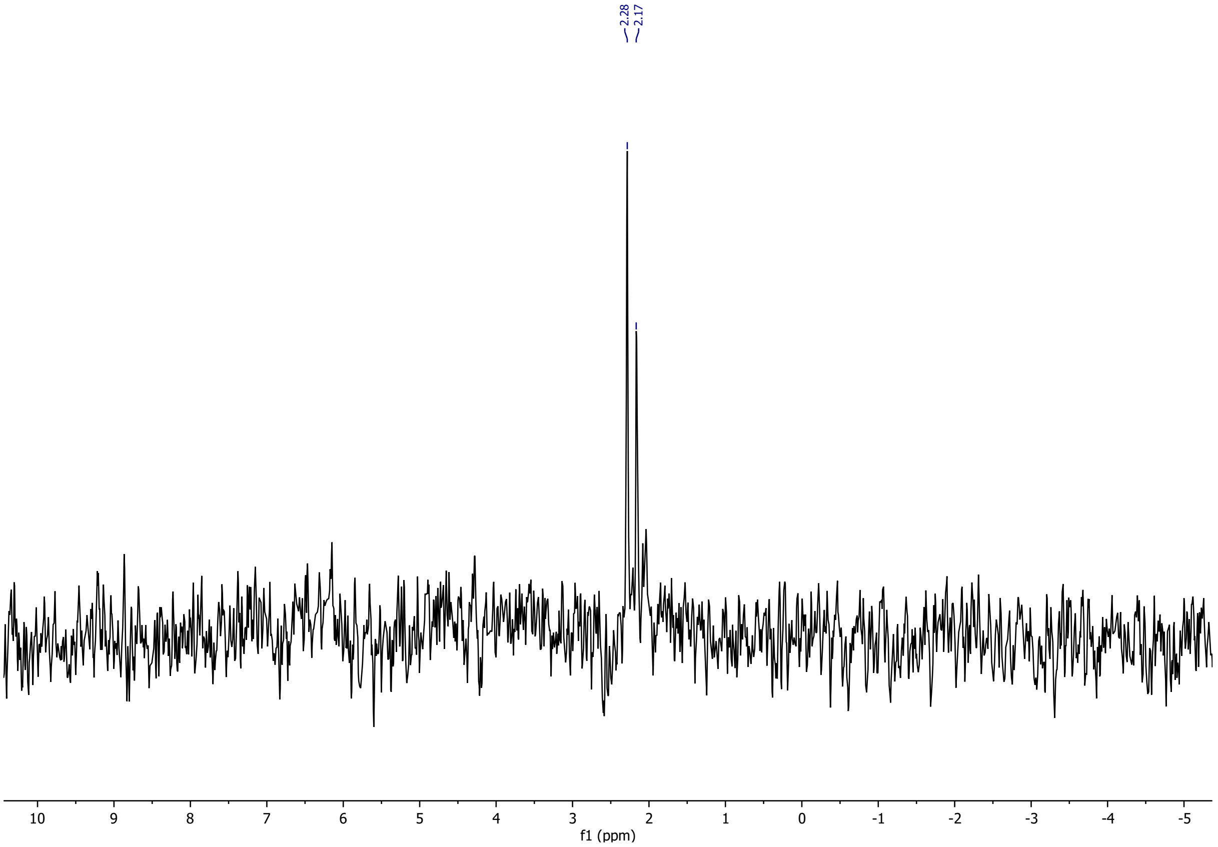

Figure S52. $^{29}Si\{^1H\}$ NMR spectrum (99 MHz, C_6D_6, 298K) of ***2*-p*BP***.

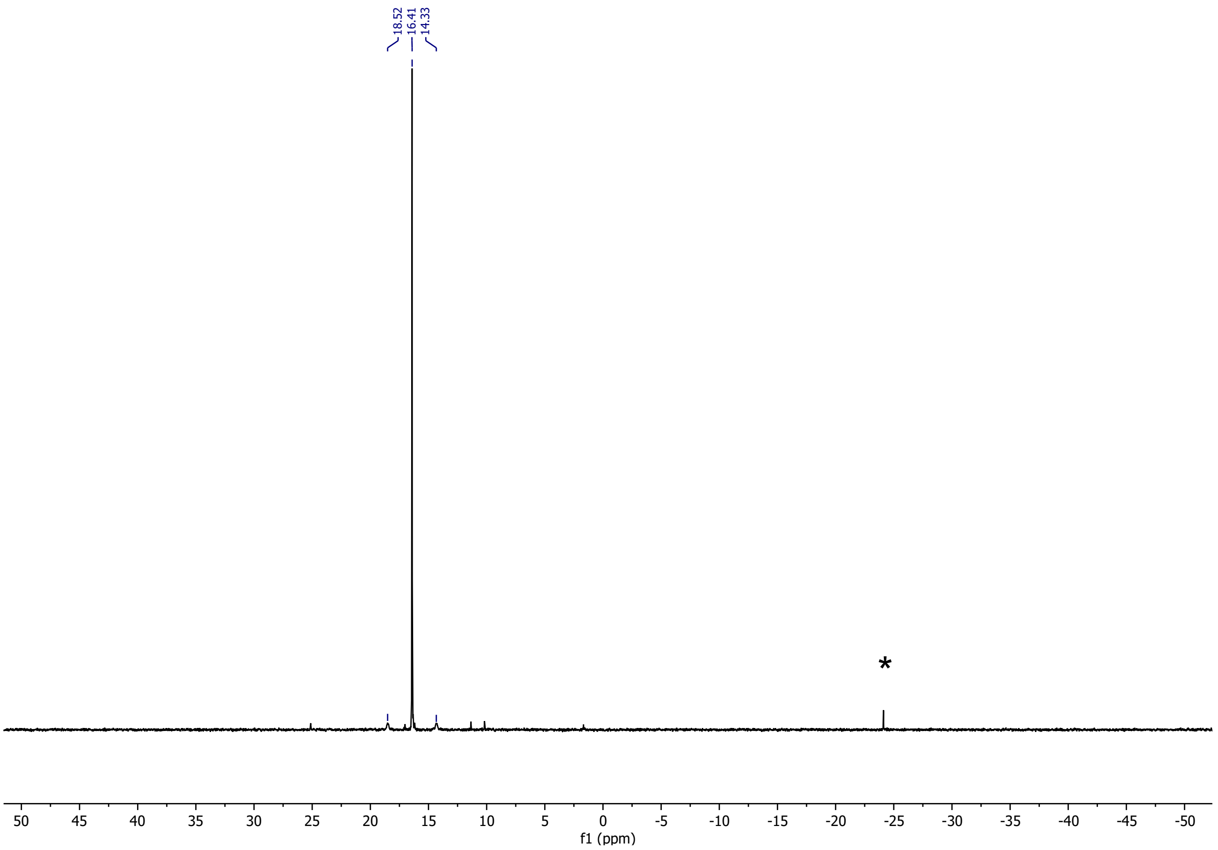

Figure S53. $^{31}P\{^{1}H\}$ NMR spectrum (162 MHz, C_6D_6, 298K) of ***2-pBP***. * marks ^{Ph}LH.

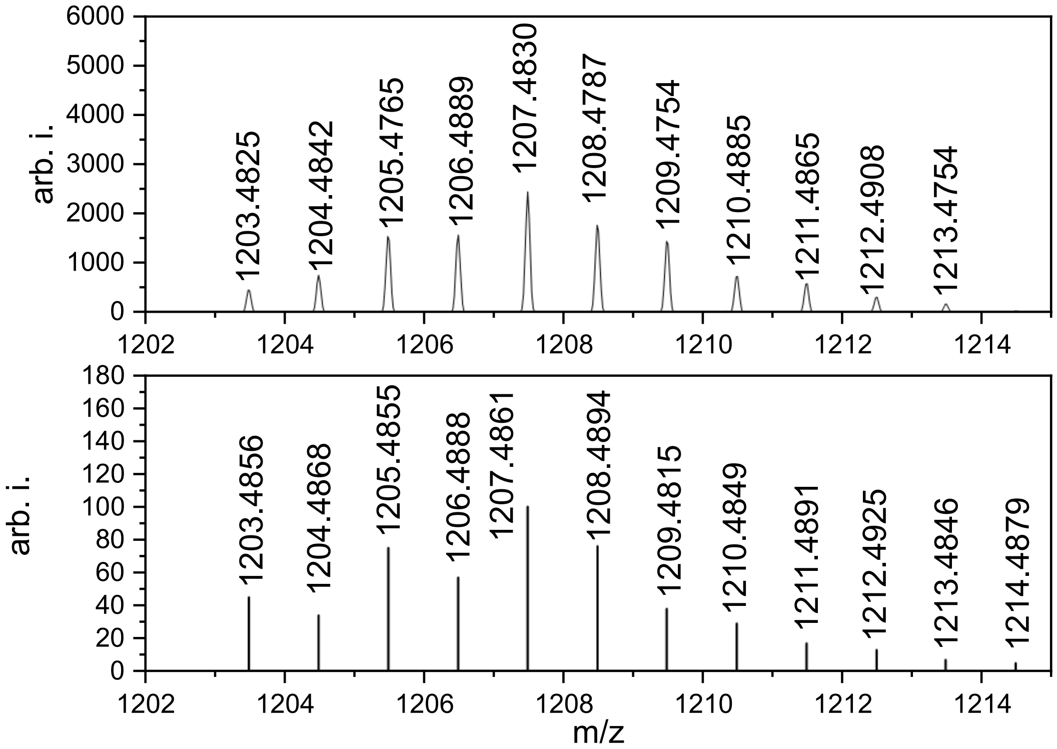

Figure S54. LIFDI-MS of ***2-pBP***, Top found MS for [^{Ph}L(*p*-BPh)Sn·Ni·IPr]+ Bottom. Calculated MS spectrum of [^{Ph}L(*p*-BPh)Sn·Ni·IPr]+.

Figure S55. UV-Vis spectrum of ***2*-p*BP*** in toluene (0.5x10^{-4} M) at ambient temperature.

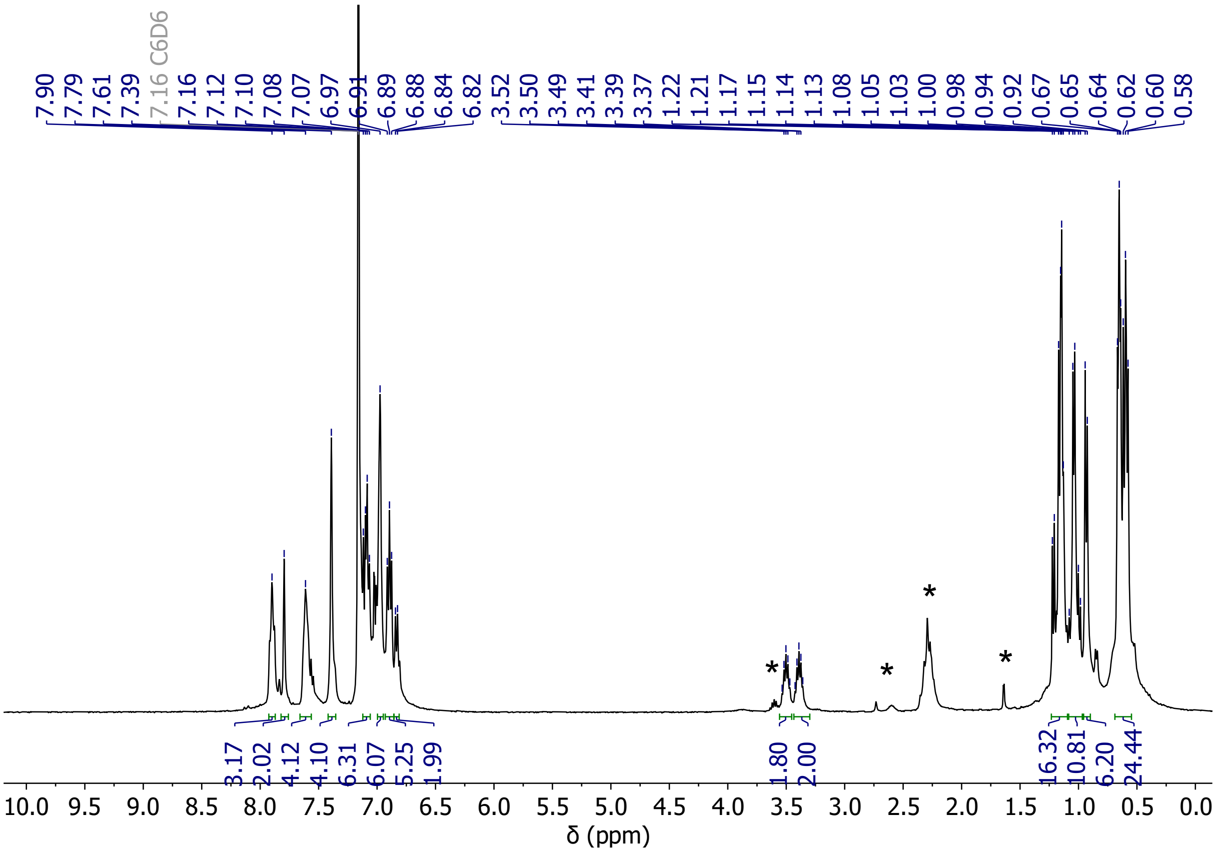

Figure S56. ^{1}H NMR spectrum (400 MHz, C_6D_6, 298K) of **3**. *marks unidentified impurities.

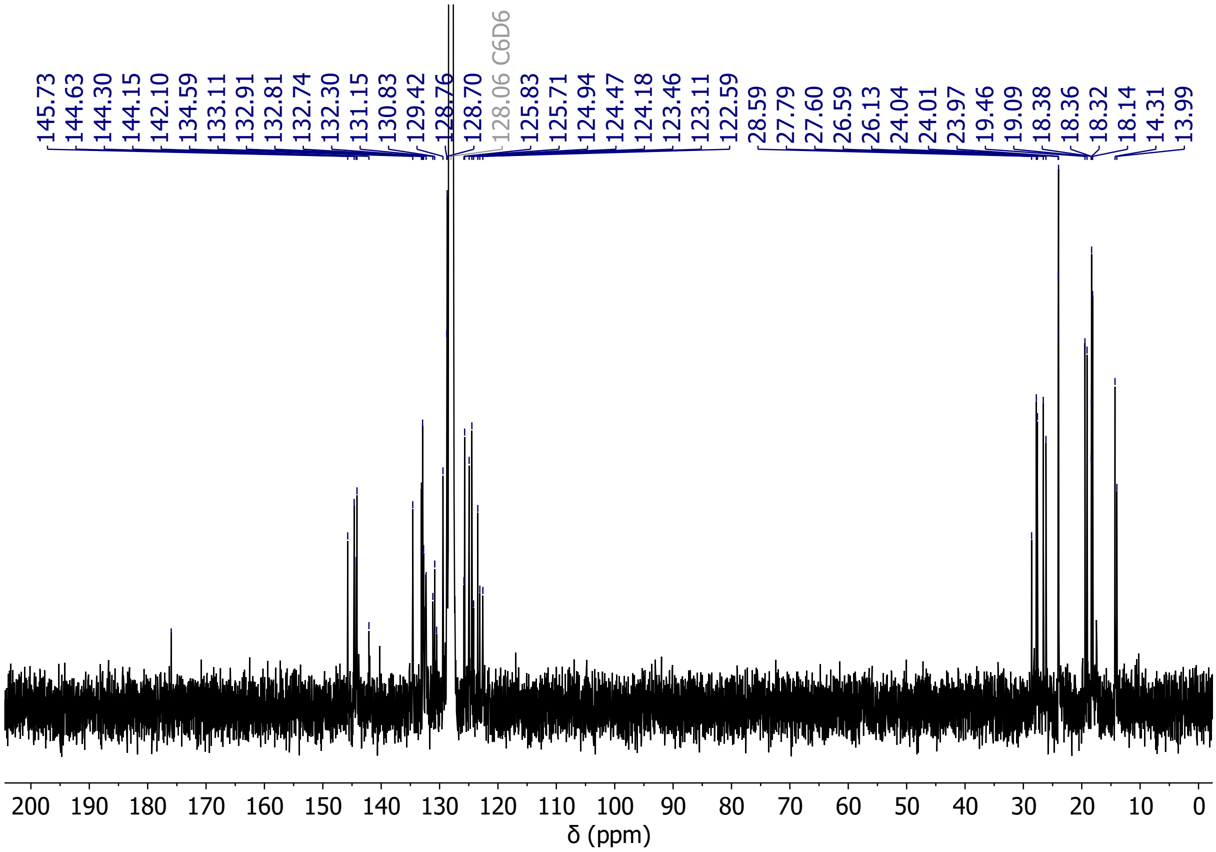

Figure S57. ^{13}C NMR spectrum (400 MHz, C_6D_6, 298K) of **3**.

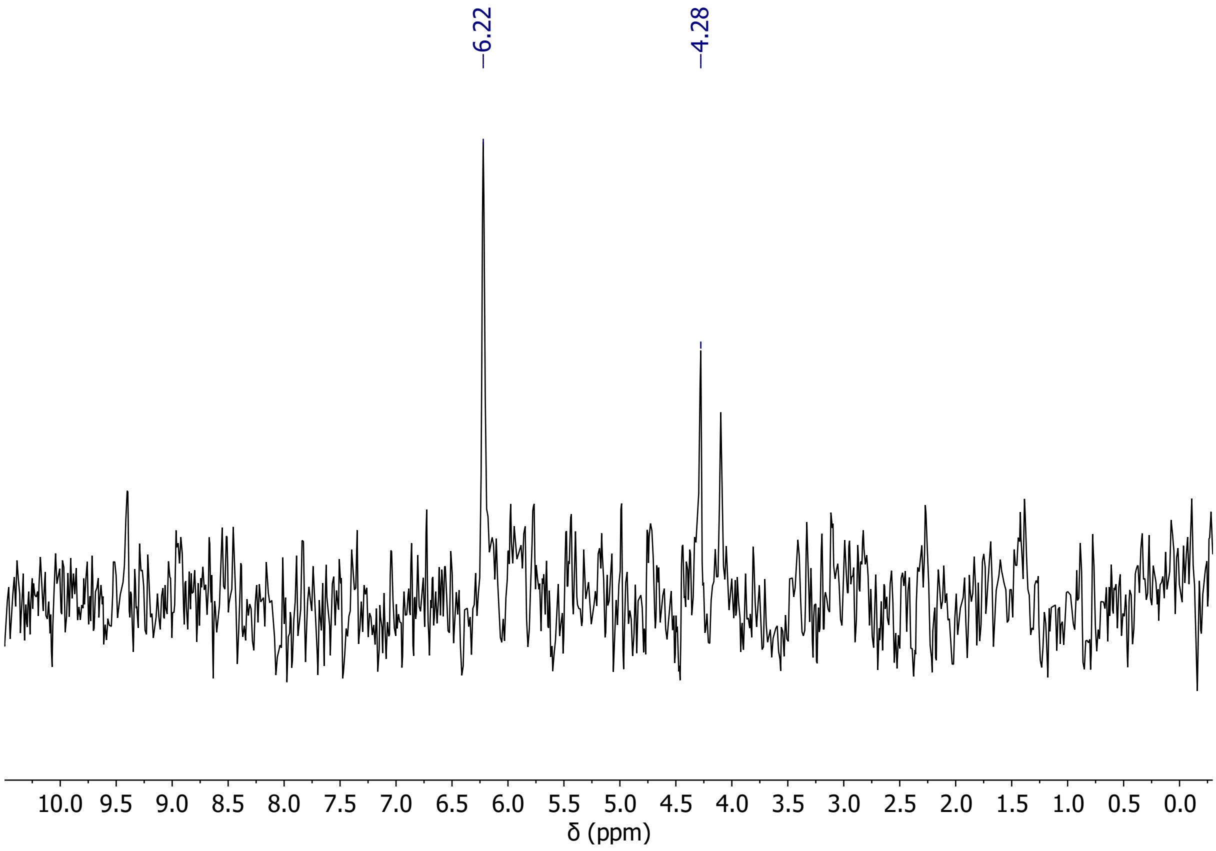

Figure S58. $^{29}Si\{^1H\}$ NMR spectrum (99 MHz, C_6D_6, 298K) of **3**.

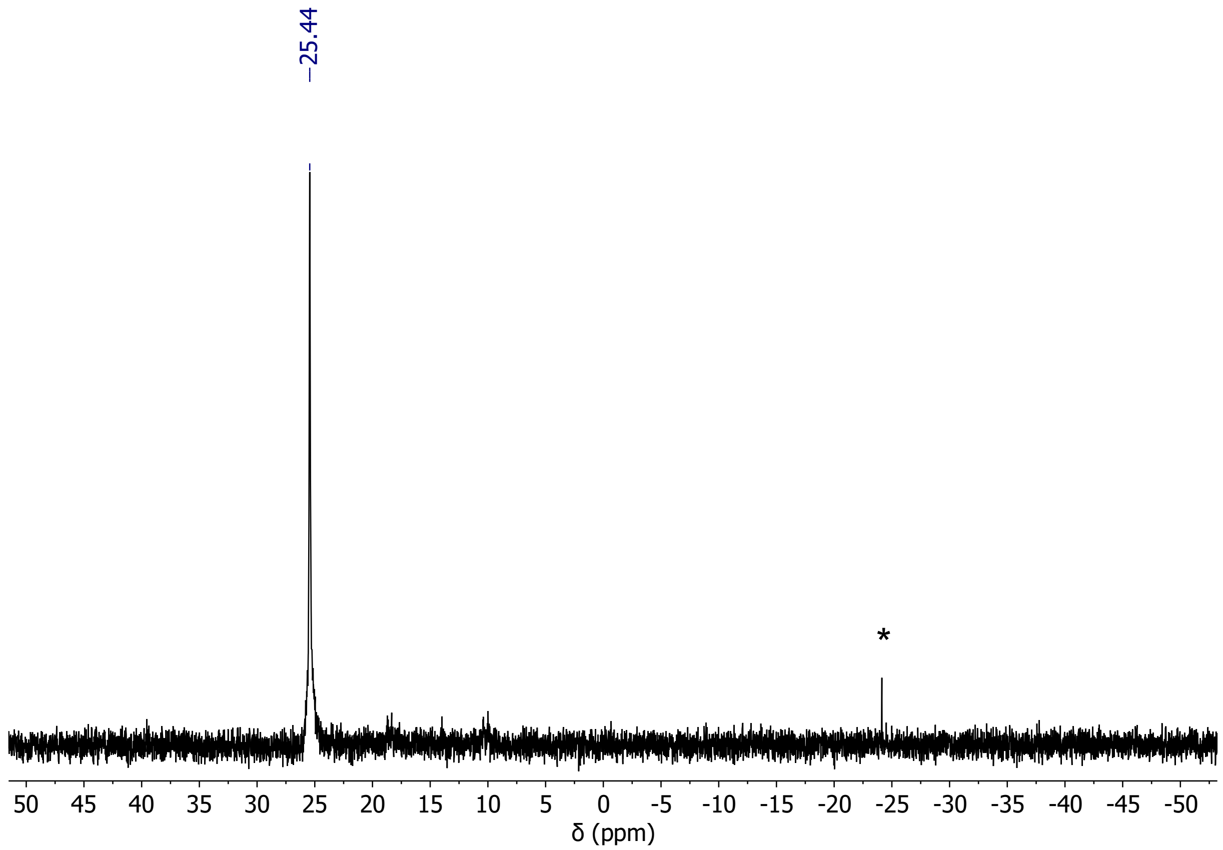

Figure S59. $^{31}P\{^{1}H\}$ NMR spectrum (162 MHz, C_6D_6, 298K) of **3**. * marks ^{Ph}LH.

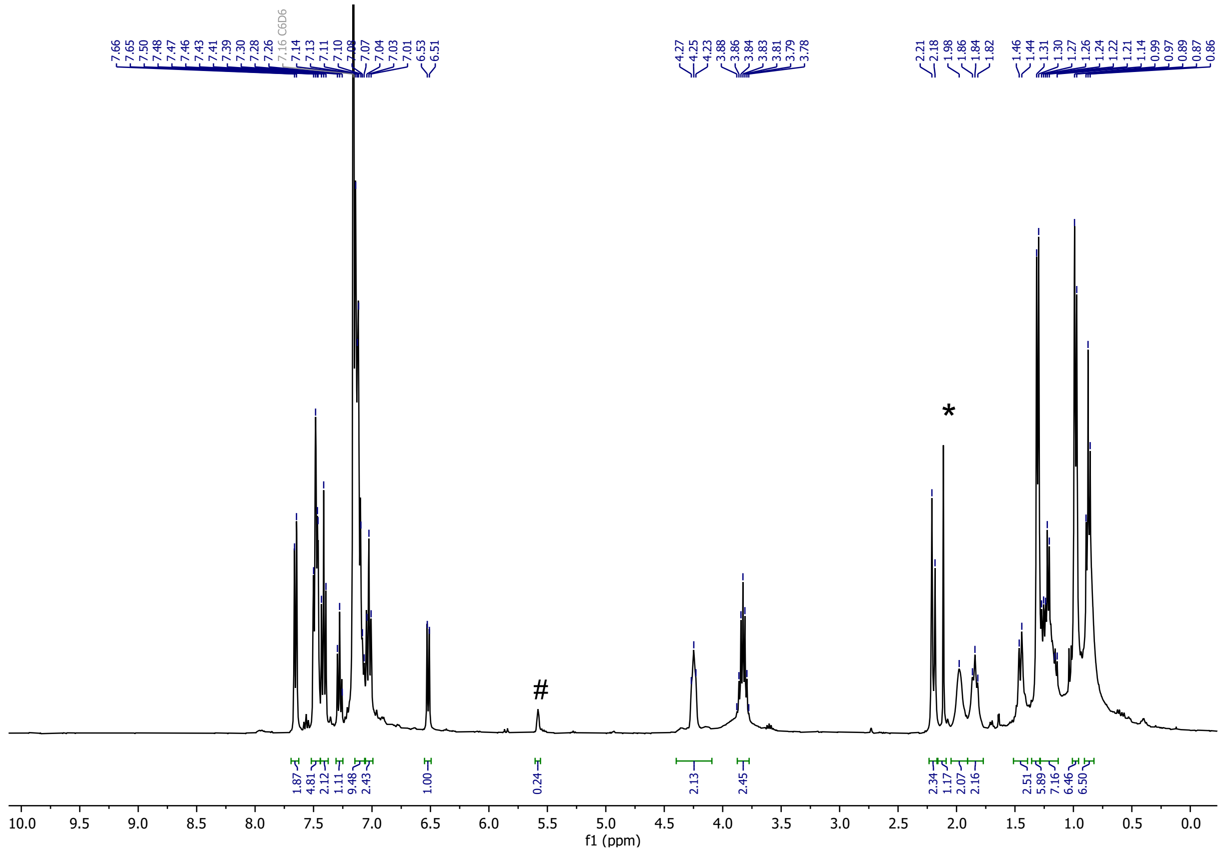

Figure S60. ^{1}H NMR spectrum (400 MHz, C_6D_6, 298K) of **4**. # marks free cod and * marks toluene.

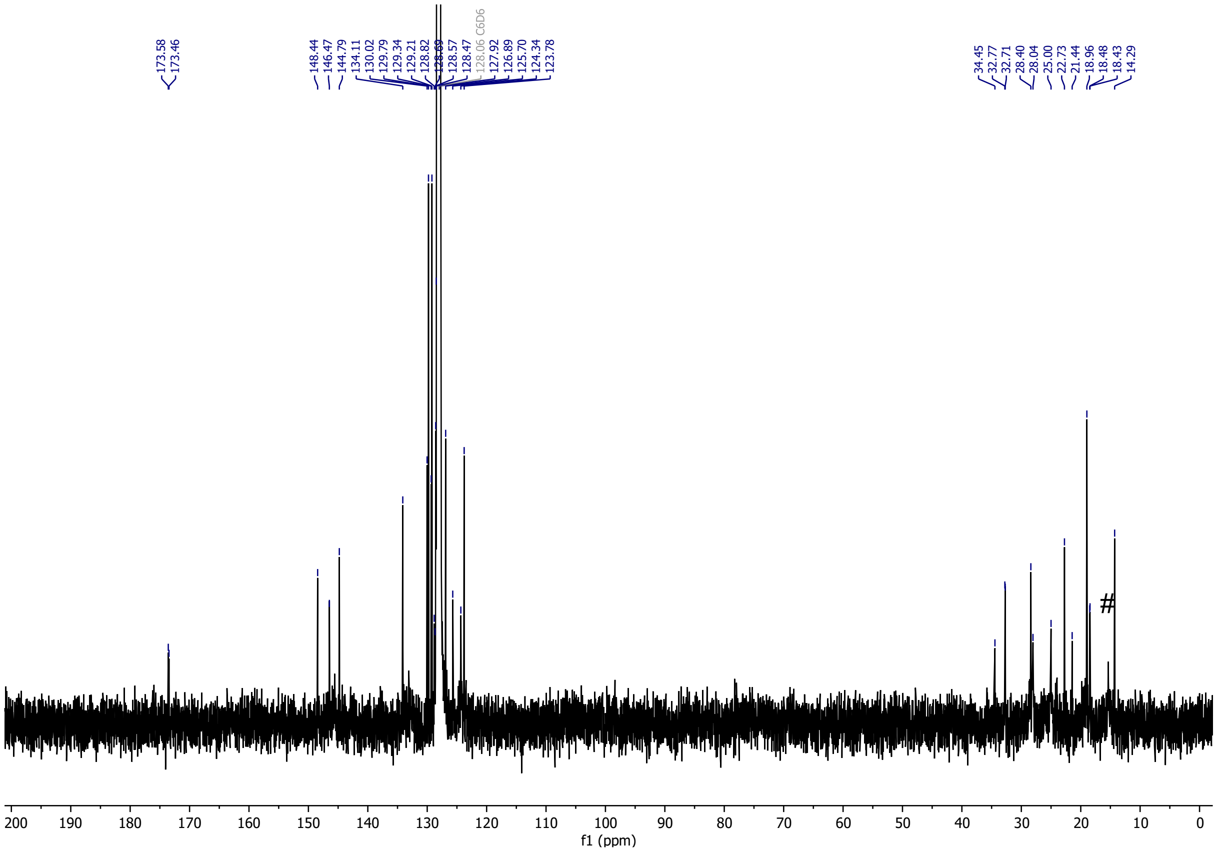

Figure S61. ^{13}C NMR spectrum (400 MHz, C_6D_6, 298K) of **4**. # shows unidentified impurity.

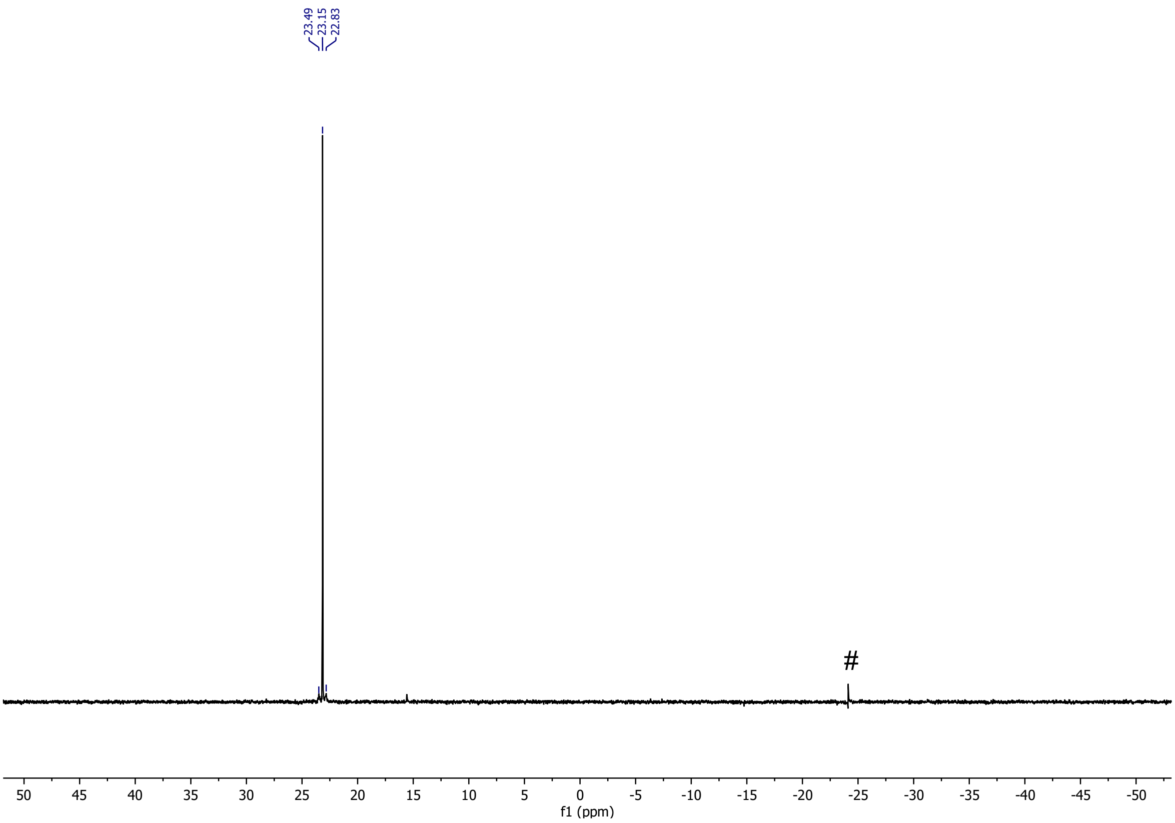

Figure S62 $^{31}P\{^1H\}$ NMR spectrum (162 MHz, C_6D_6, 298K) of **4**. # marks ^{Ph}LH.

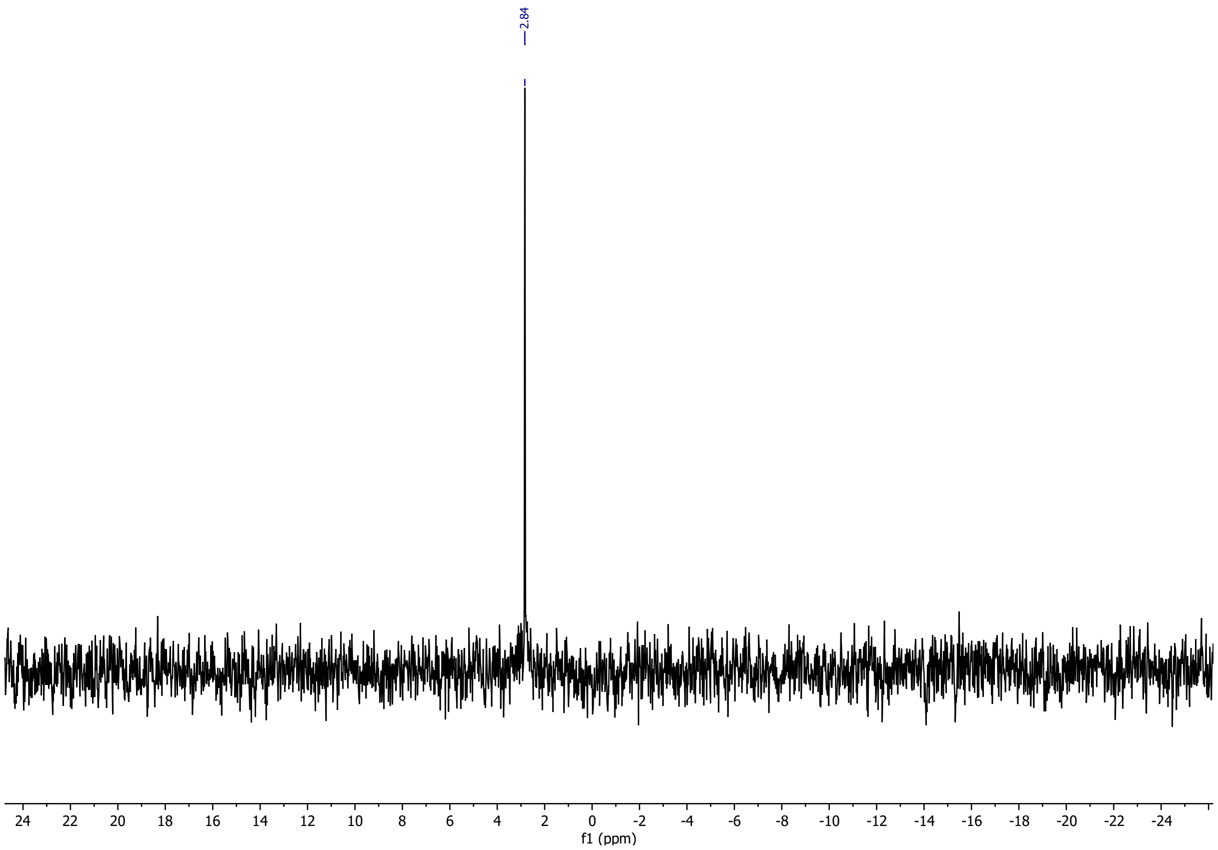

Figure S63. $^{29}Si\{^1H\}$ NMR spectrum (99 MHz, C_6D_6, 298K) of **4**.

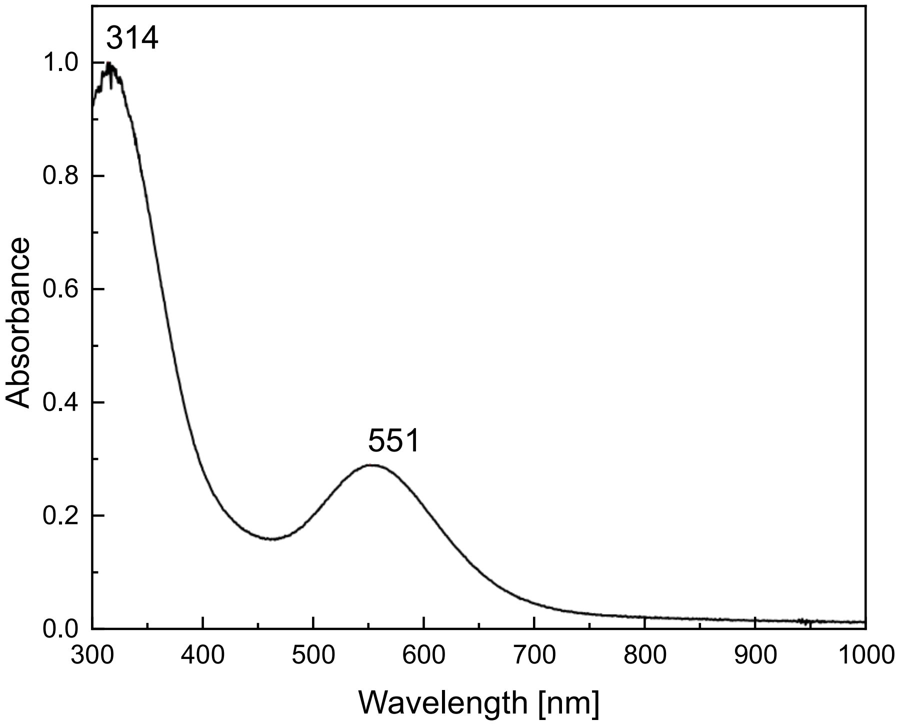

Figure S64: UV-Vis spectrum of **4** in toluene ($1.0x10^{-4}$ M) at ambient temperature.

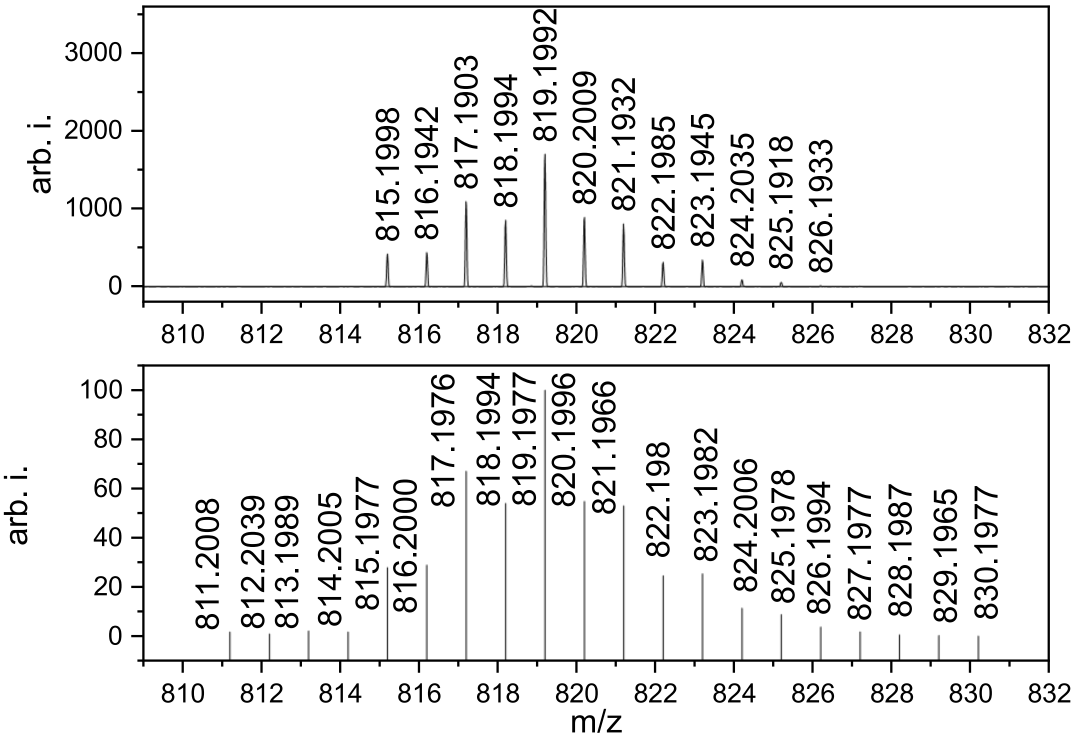

Figure S65. LIFDI-MS of **4**, Top found MS for [^{Ph}L(*o*-BPh)Sn·Ni]$^+$ Bottom. Calculated MS spectrum of [^{Ph}L(*o*-BPh)Sn·Ni]$^+$.

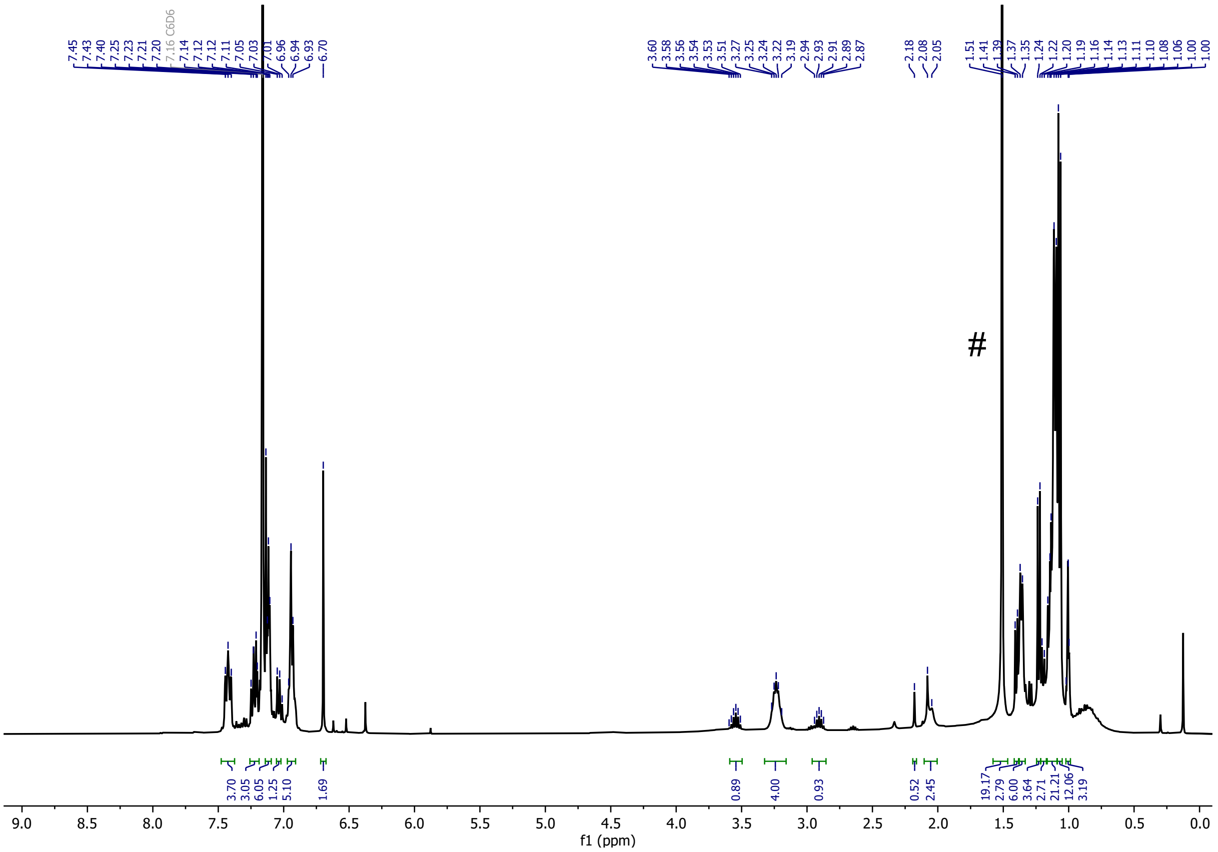

Figure S66. ^{1}H NMR spectrum (400 MHz, C_6D_6, 298K) of **5**. # marks an unknown spezies.

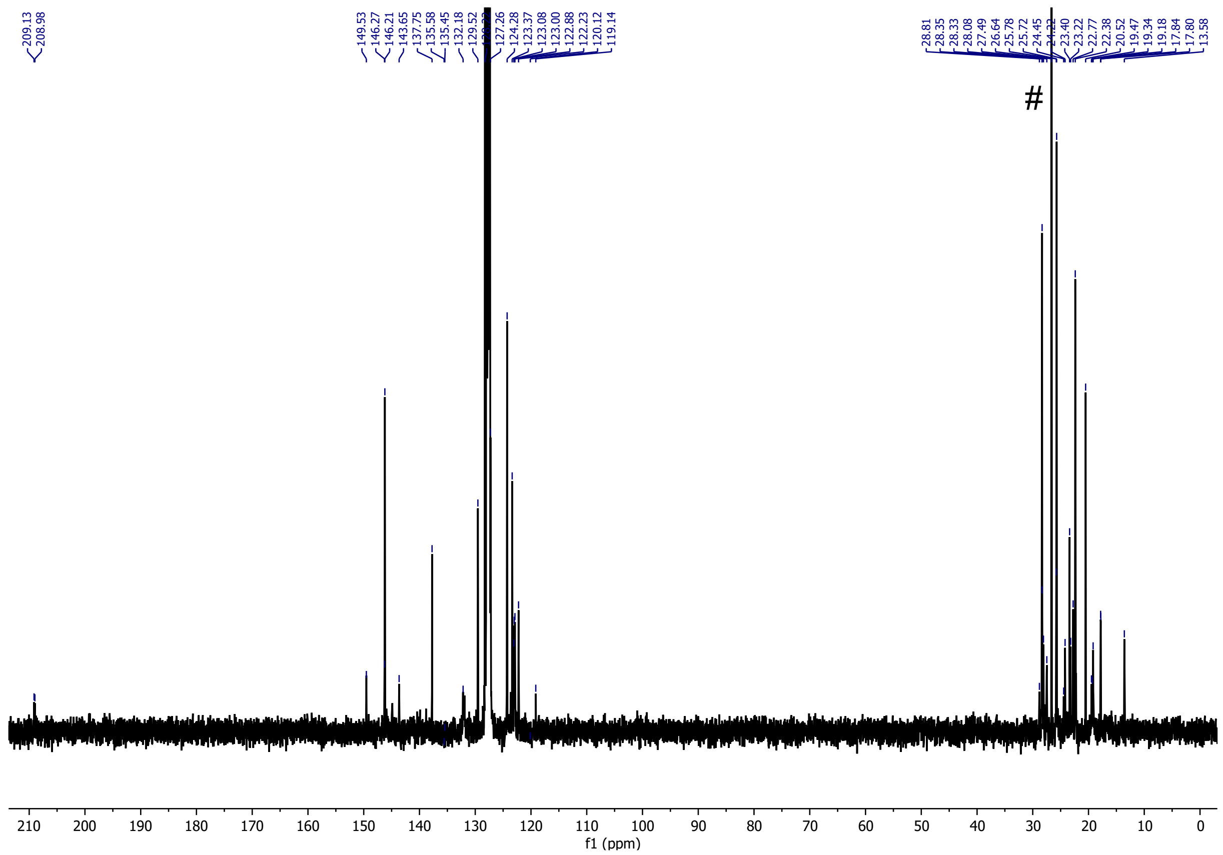

Figure S67. ^{13}C NMR spectrum (400 MHz, C_6D_6, 298K) of **5**. # marks an unknown spezies.

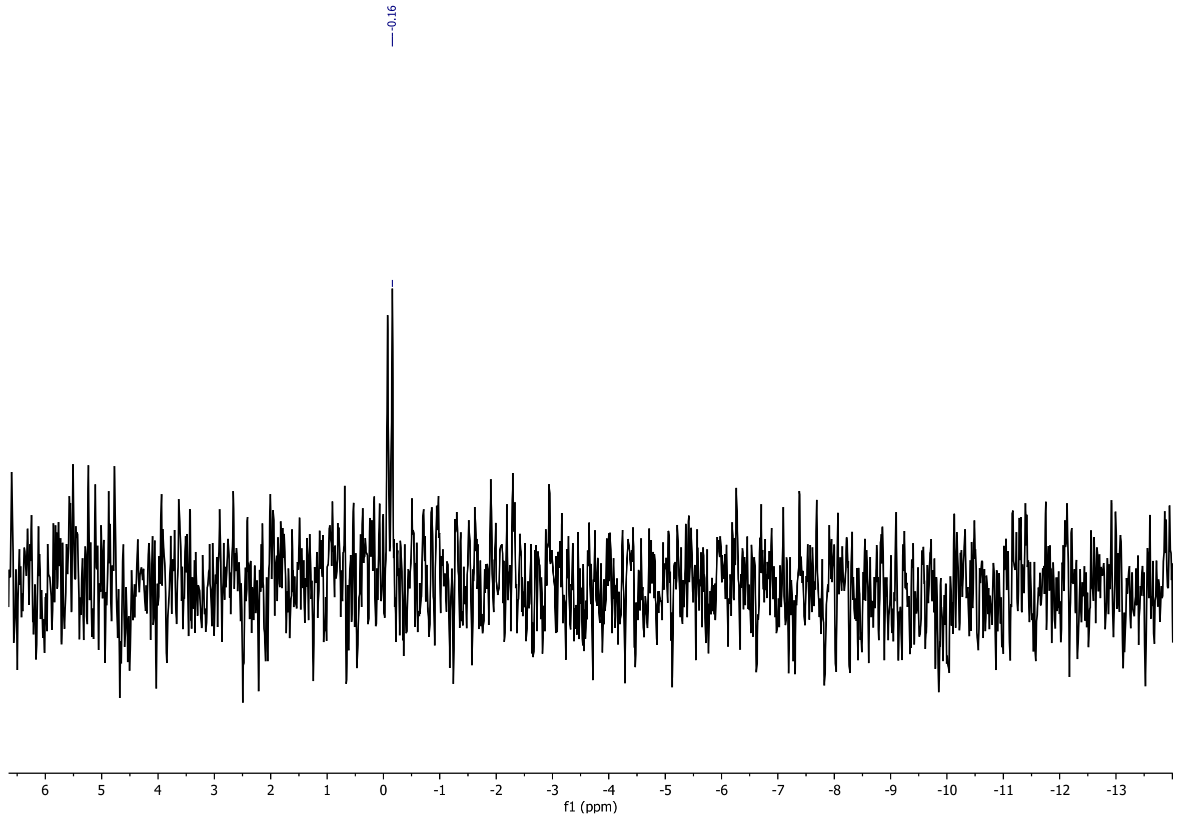

Figure S68. $^{29}Si\{^1H\}$ NMR spectrum (99 MHz, C_6D_6, 298K) of **5**.

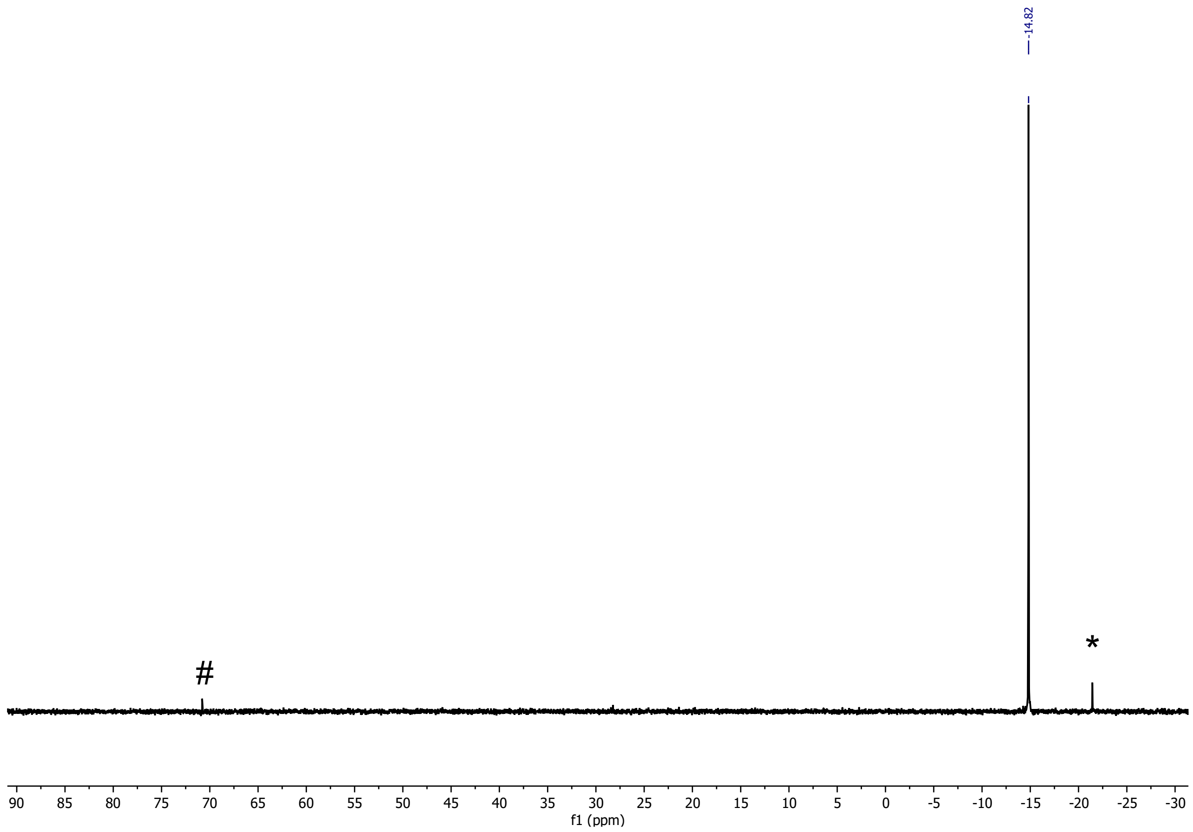

Figure S69. $^{31}P\{^{1}H\}$ NMR spectrum (162 MHz, C_6D_6, 298K) of **5**. * marks the unknown species **5** reacts to and # marks **7.**

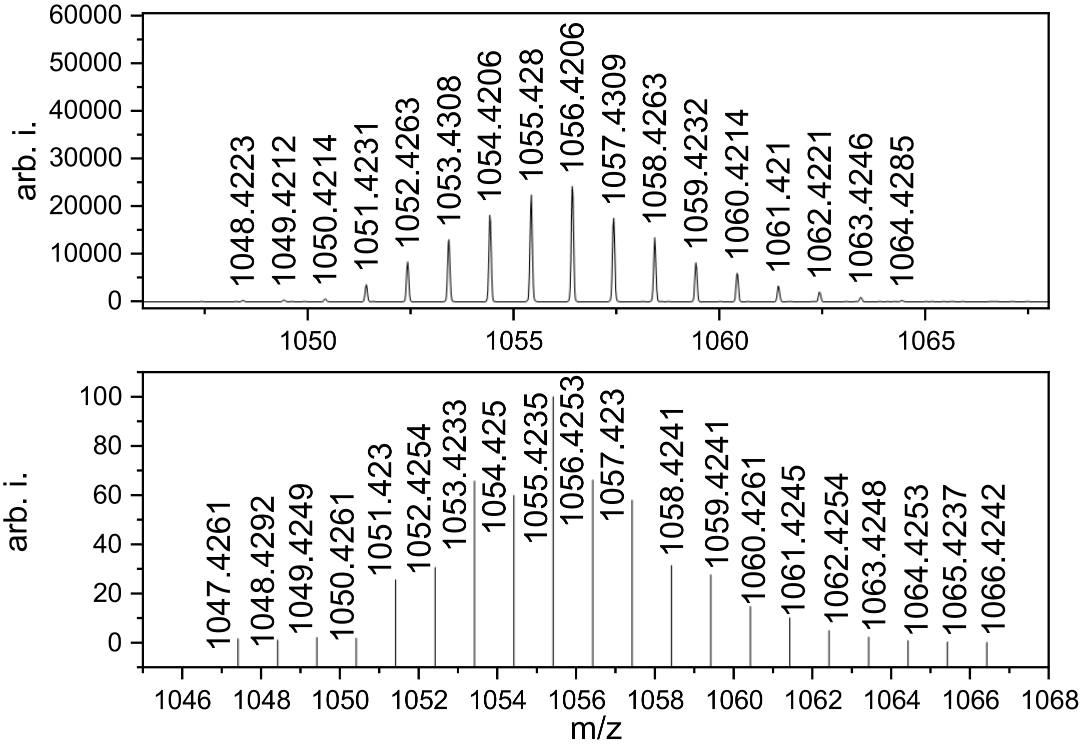

Figure S70. LIFDI-MS of **5**, Top found MS for $[^{Ph}LSn(\mu\text{-}H)Ni{\cdot}IPr]^{+}$ Bottom. Calculated MS spectrum of $[^{Ph}LSn(\mu\text{-}H)Ni{\cdot}IPr]^{+}$.

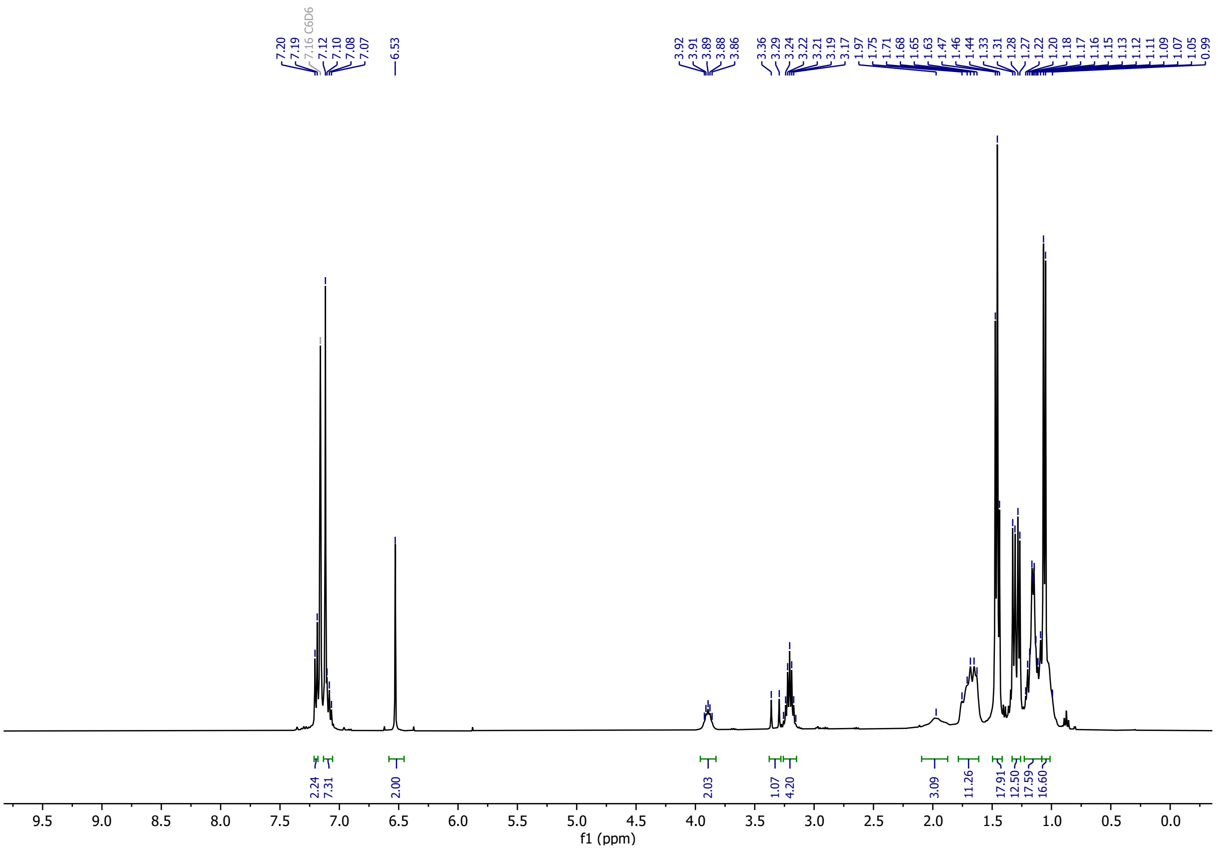

Figure S71. ^{1}H NMR spectrum (400 MHz, C_6D_6, 298K) of **6**.

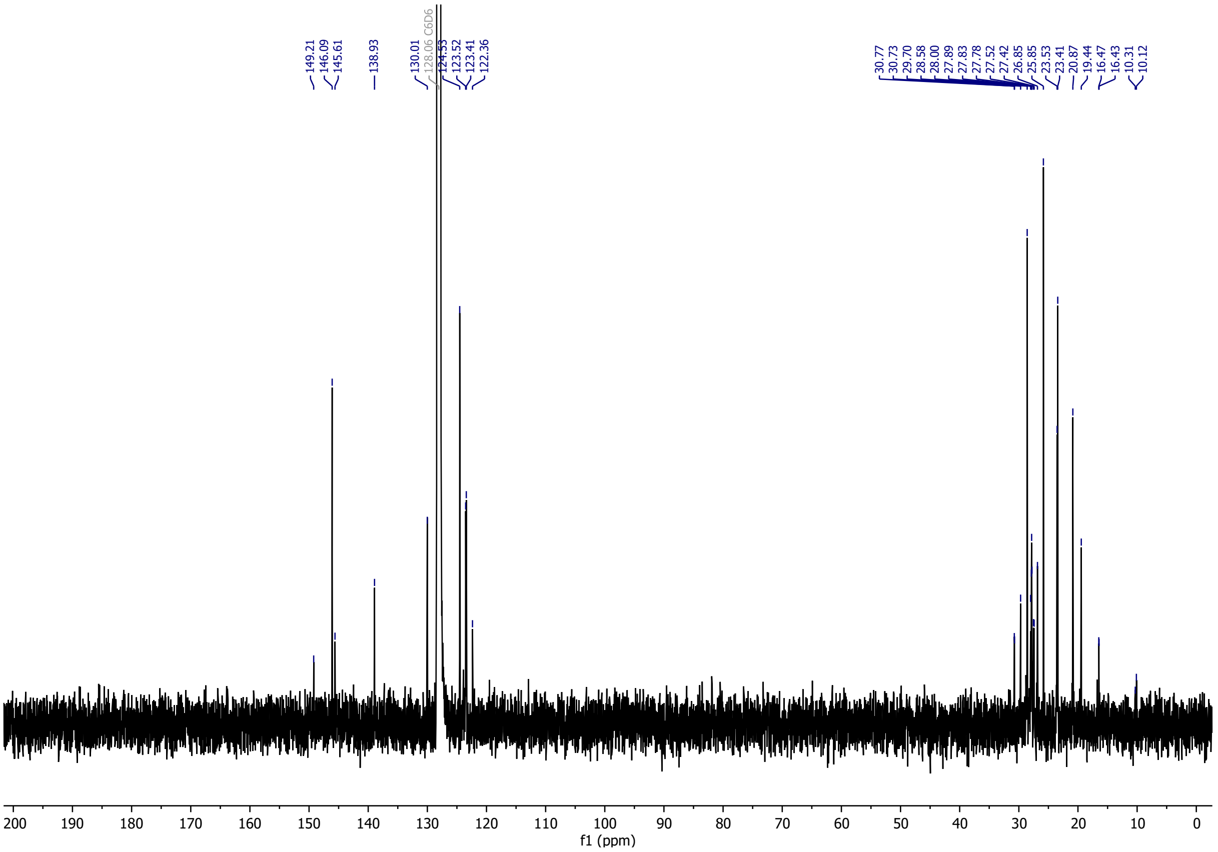

Figure S72. ^{13}C NMR spectrum (400 MHz, C_6D_6, 298K) of **6**.

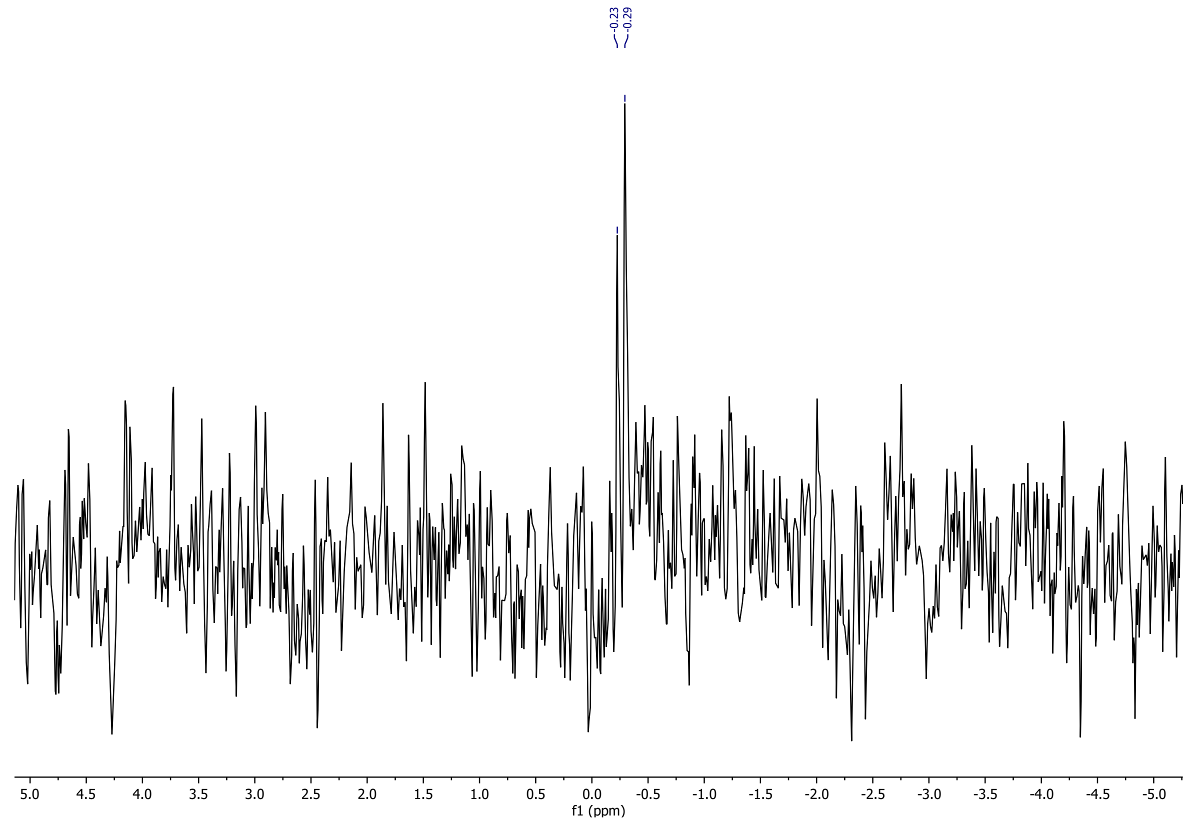

Figure S73. $^{29}Si\{^1H\}$ NMR spectrum (99 MHz, C_6D_6, 298K) of **6**.

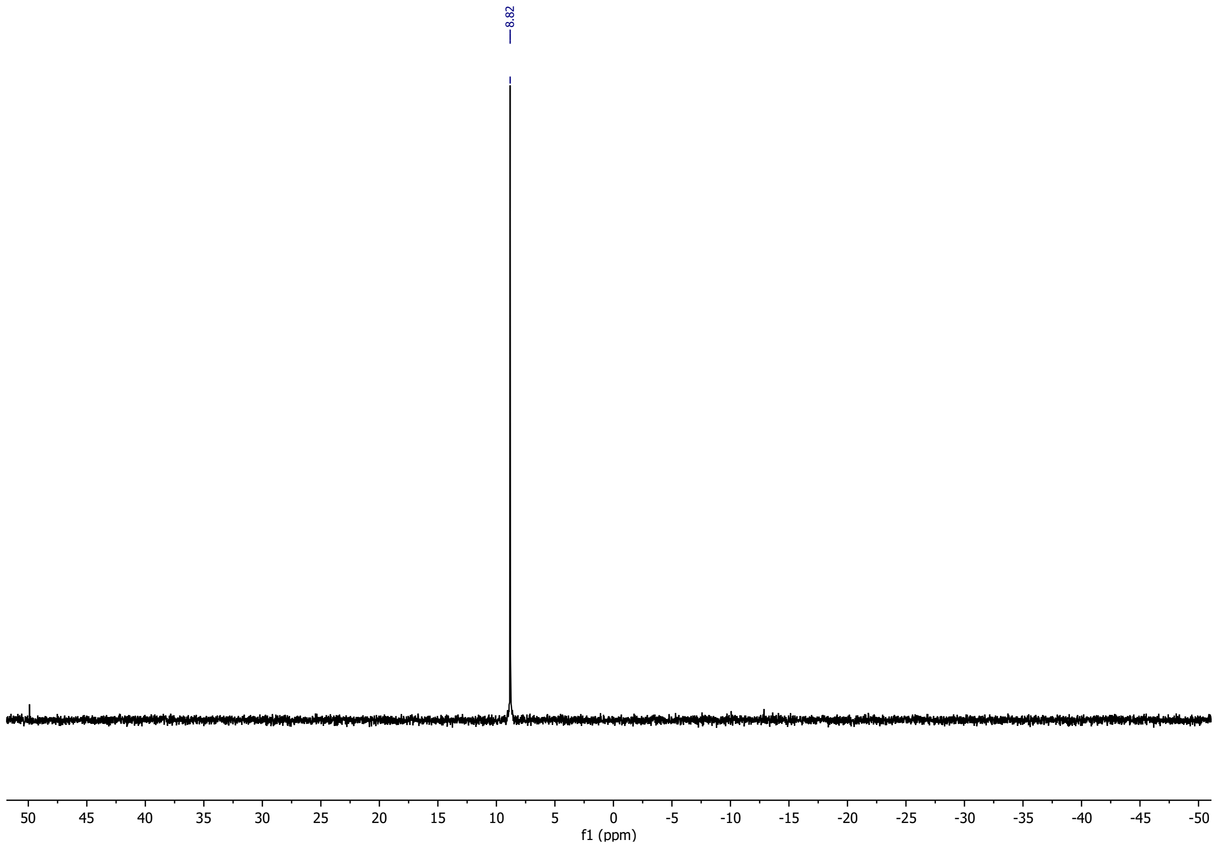

Figure S74. $^{31}P\{^1H\}$ NMR spectrum (162 MHz, C_6D_6, 298K) of **6**.

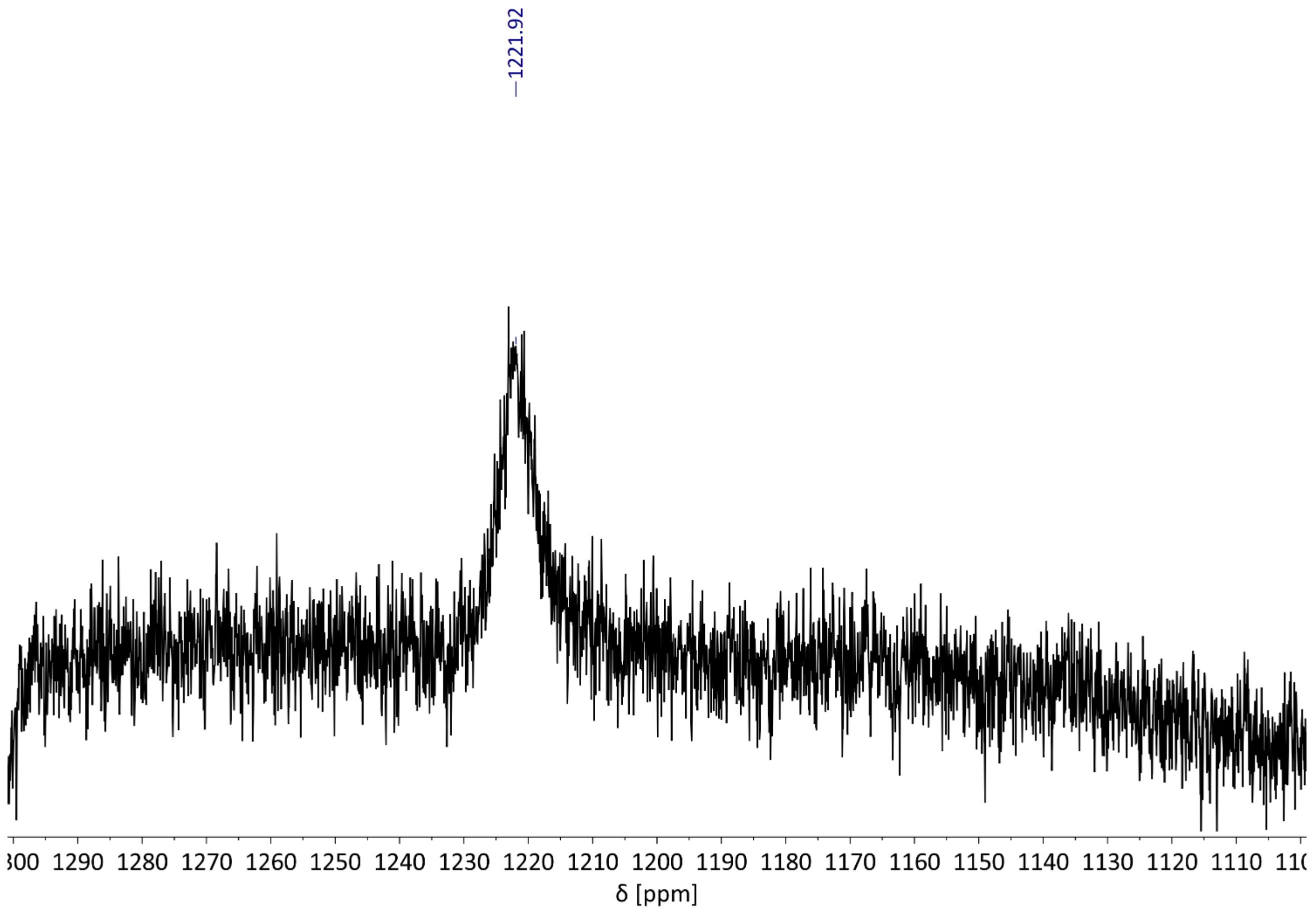

Figure S75. $^{119}Sn\{^{1}H\}$ NMR spectrum (C_6D_6, 298K) of **2-Ph**.

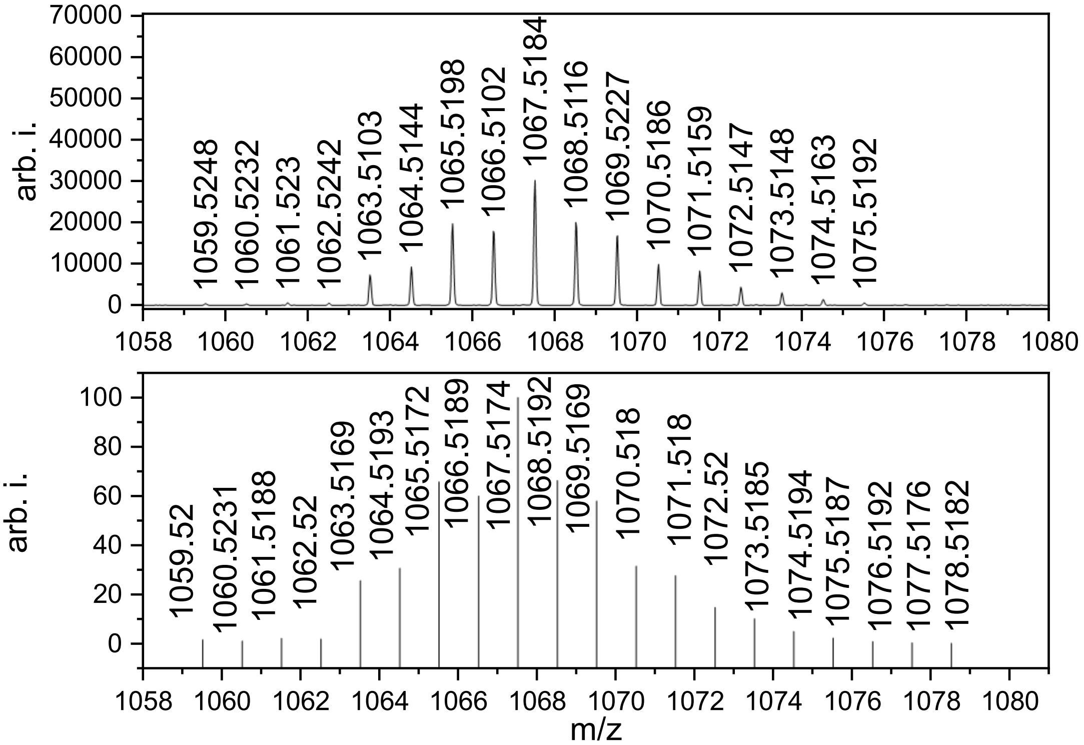

Figure S76. LIFDI-MS of **6**, Top found MS for $[^{Cy}LSn(\mu\text{-}H)Ni\cdot IPr]^+$ Bottom. Calculated MS spectrum of $[^{Cy}LSn(\mu\text{-}H)Ni\cdot IPr]^+$.

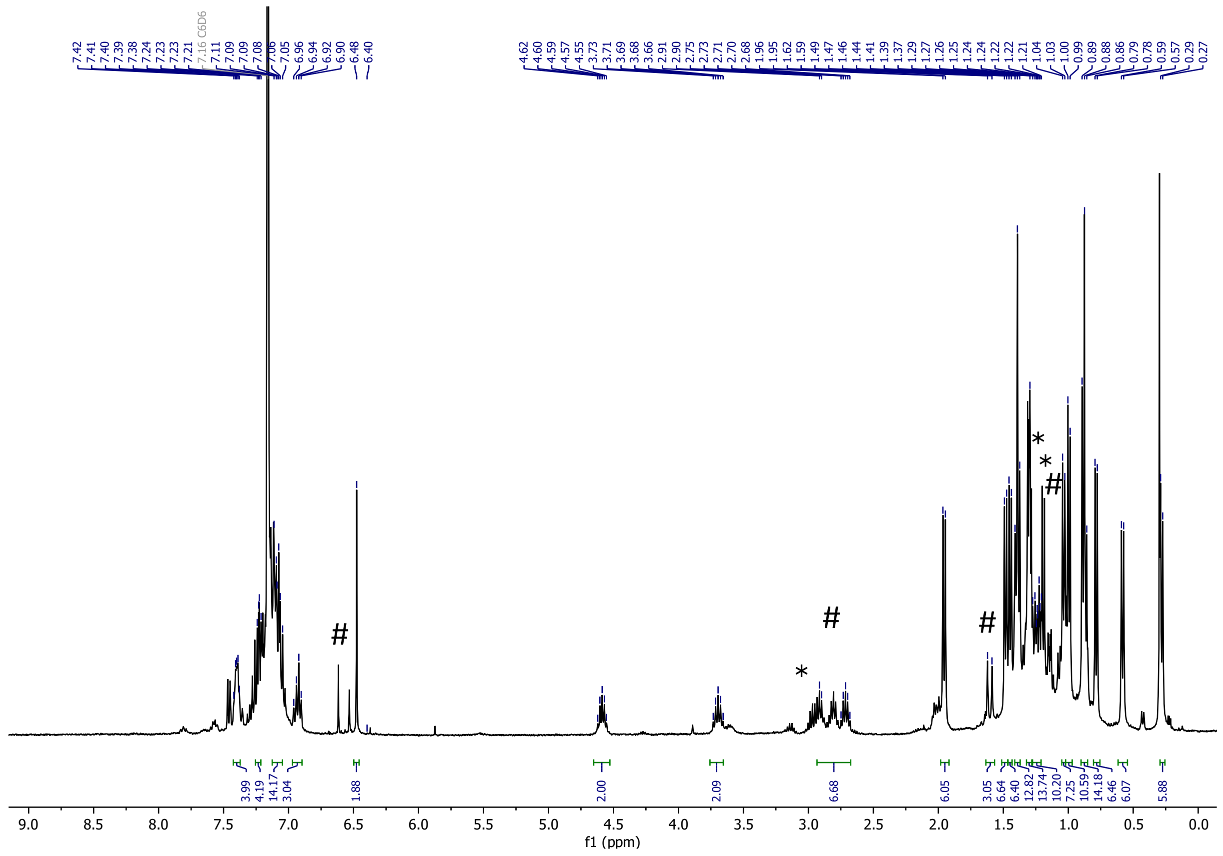

Figure S77. ^{1}H NMR spectrum (400 MHz, C_6D_6, 298K) of **7**. # marks the unknown species and * marks IPr.

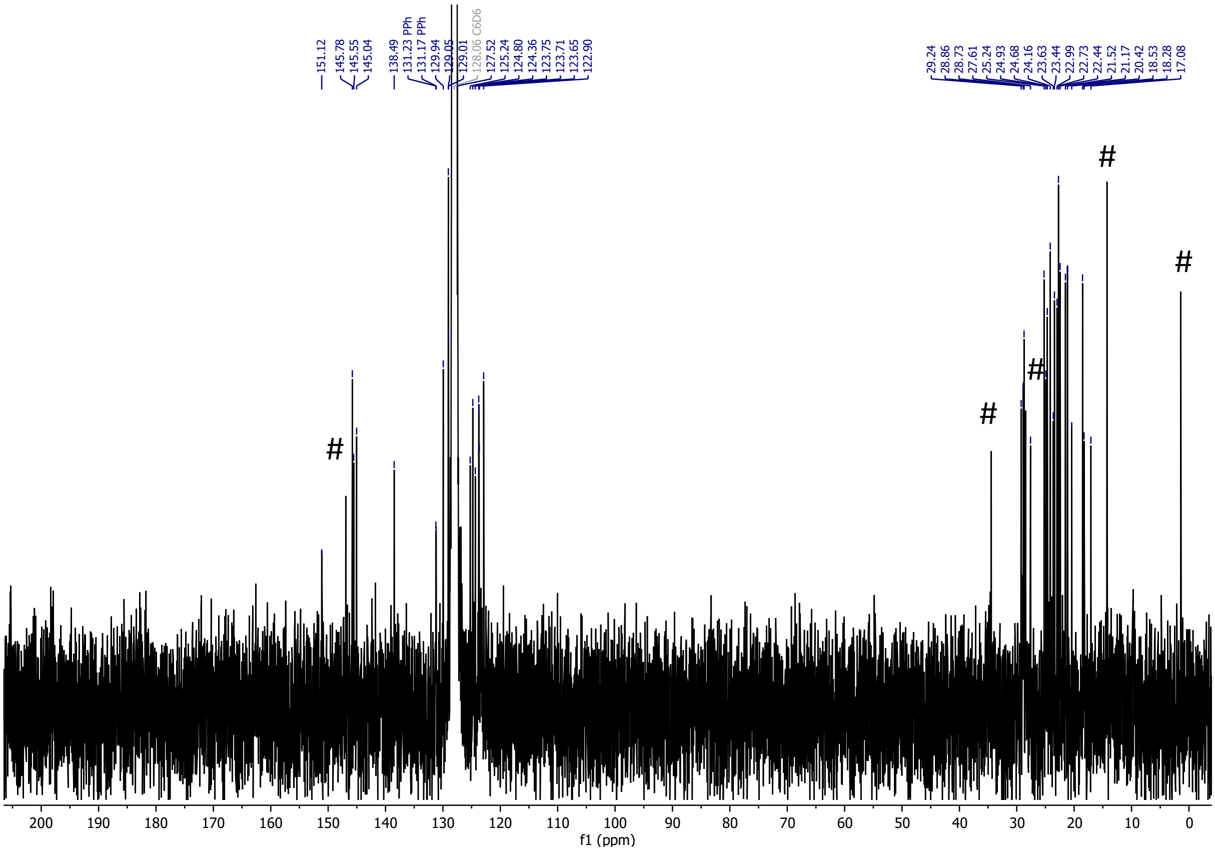

Figure S78. ^{13}C NMR spectrum (400 MHz, C_6D_6, 298K) of **7**. # marks impurities.

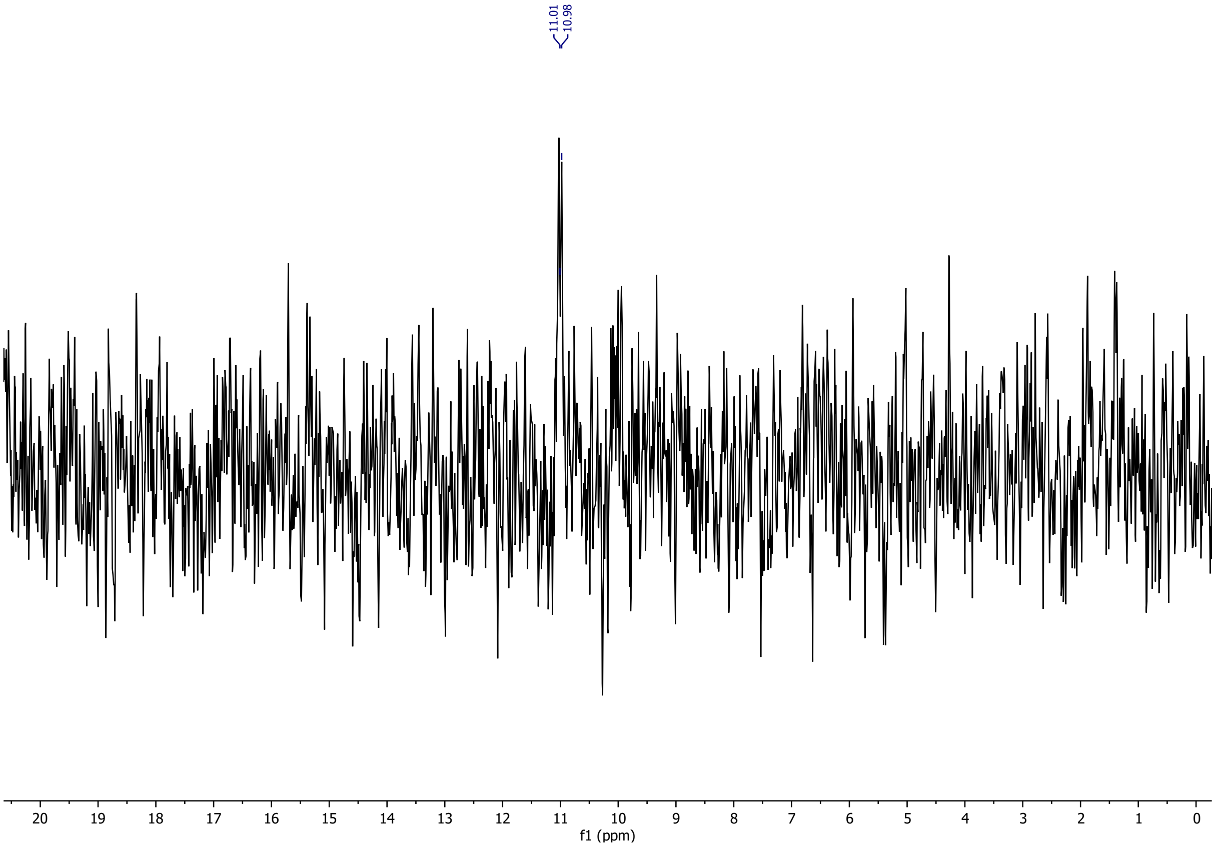

Figure S79. $^{29}Si\{^1H\}$ NMR spectrum (99 MHz, C_6D_6, 298K) of **7.**

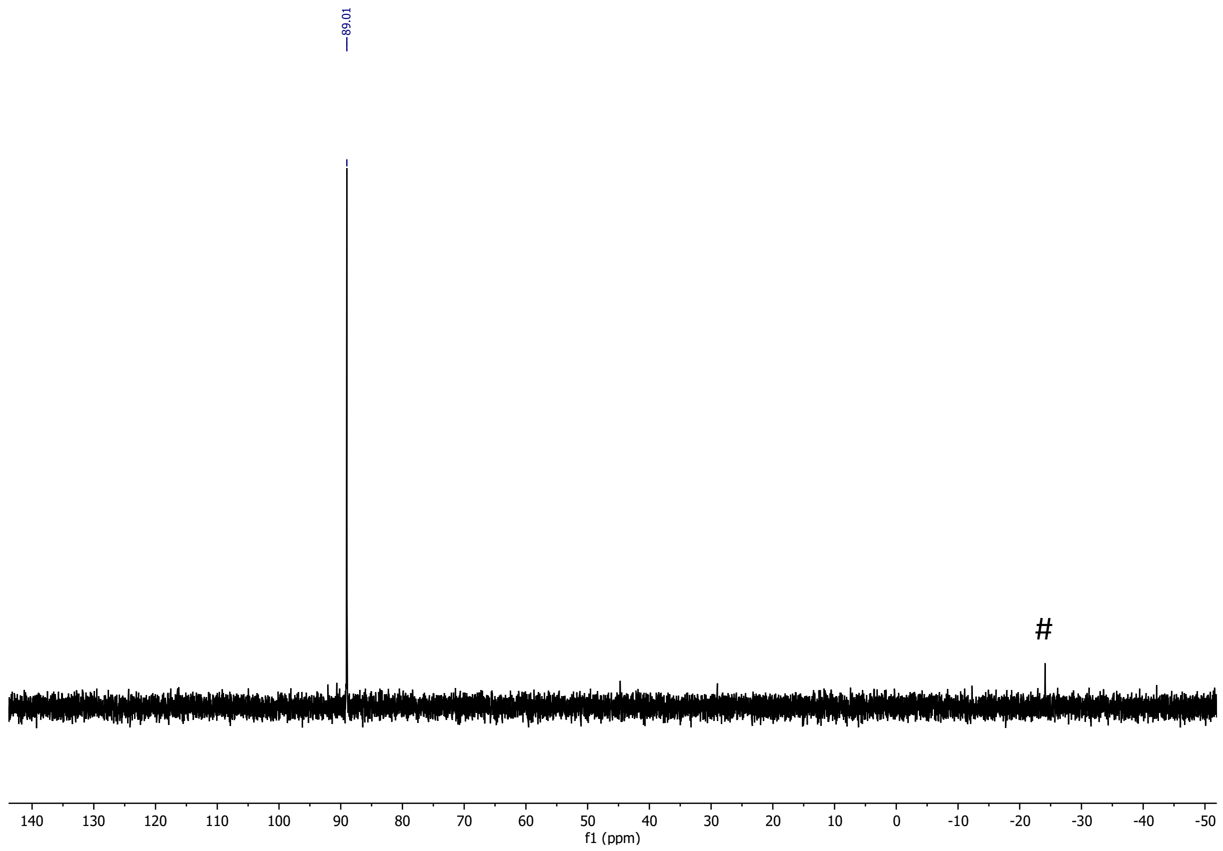

Figure S80. $^{31}P\{^{1}H\}$ NMR spectrum (162 MHz, C_6D_6, 298K) of **7**. $^{\#}$marks ^{Ph}LH.

.

3. Analysis of reactions leading to $^{Ph}L(H)Sn\cdot Ni\cdot IPr$, 5.

When pressurizing **2-Ph, 2-CF$_3$,** or **2-*p*BP** (30.0 mg) in C_6D_6 with H_2 (1.5 bar), after 16h the same single species is observed for all three starting compounds *via* $^{31}P\{^1H\}$ NMR spectroscopy (see **Figure S79**). Conducting the same experiment with D_2 leads to the same compound as evidenced by $^{31}P\{^1H\}$ NMR spectroscopy, but now with a clear and equal $^2J_{PD}$ coupling (**Figure S80**).

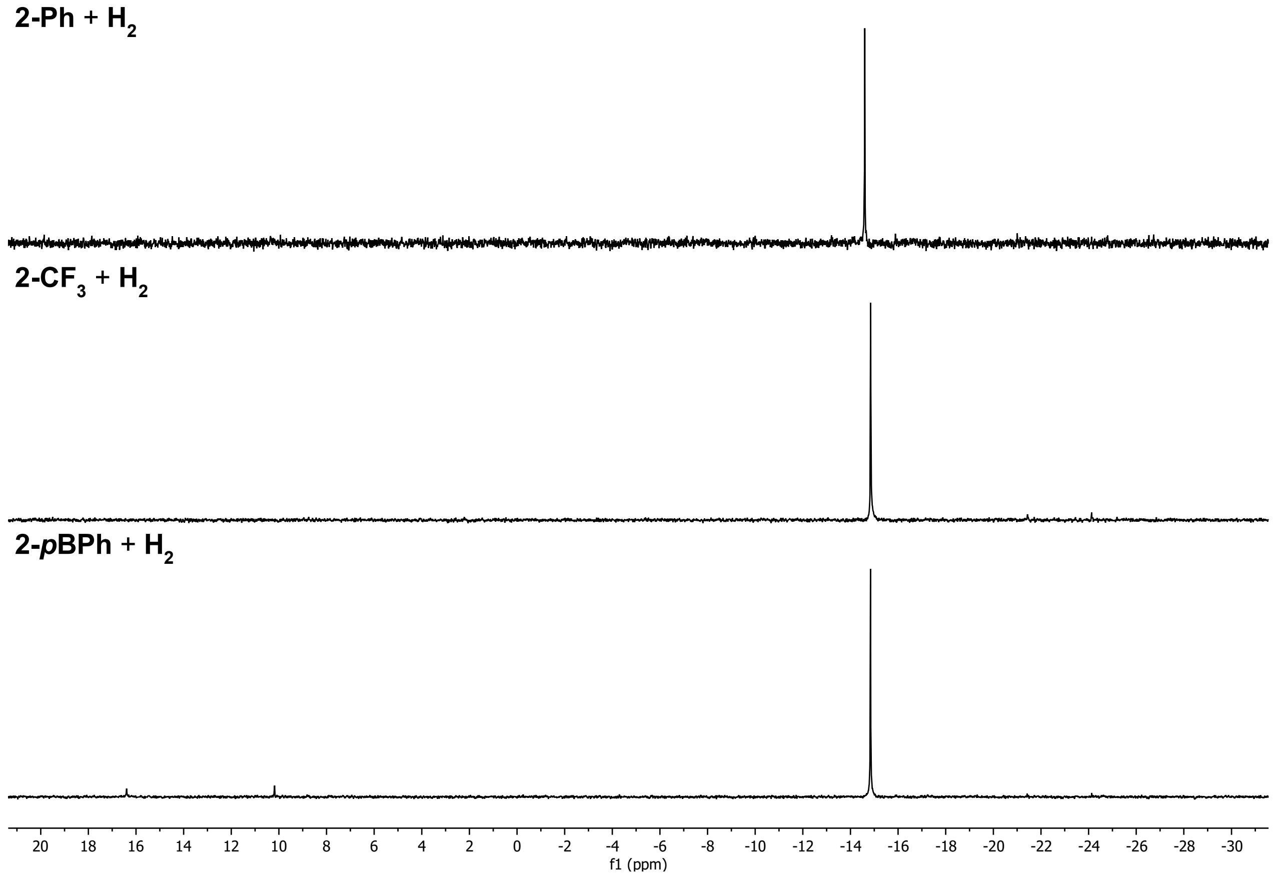

Figure S81. $^{31}P\{^1H\}$ NMR spectra (162 MHz, C_6D_6, 298K) of **2-Ph, -*p*BP, -CF$_3$** pressurized with H_2 after 16 h.

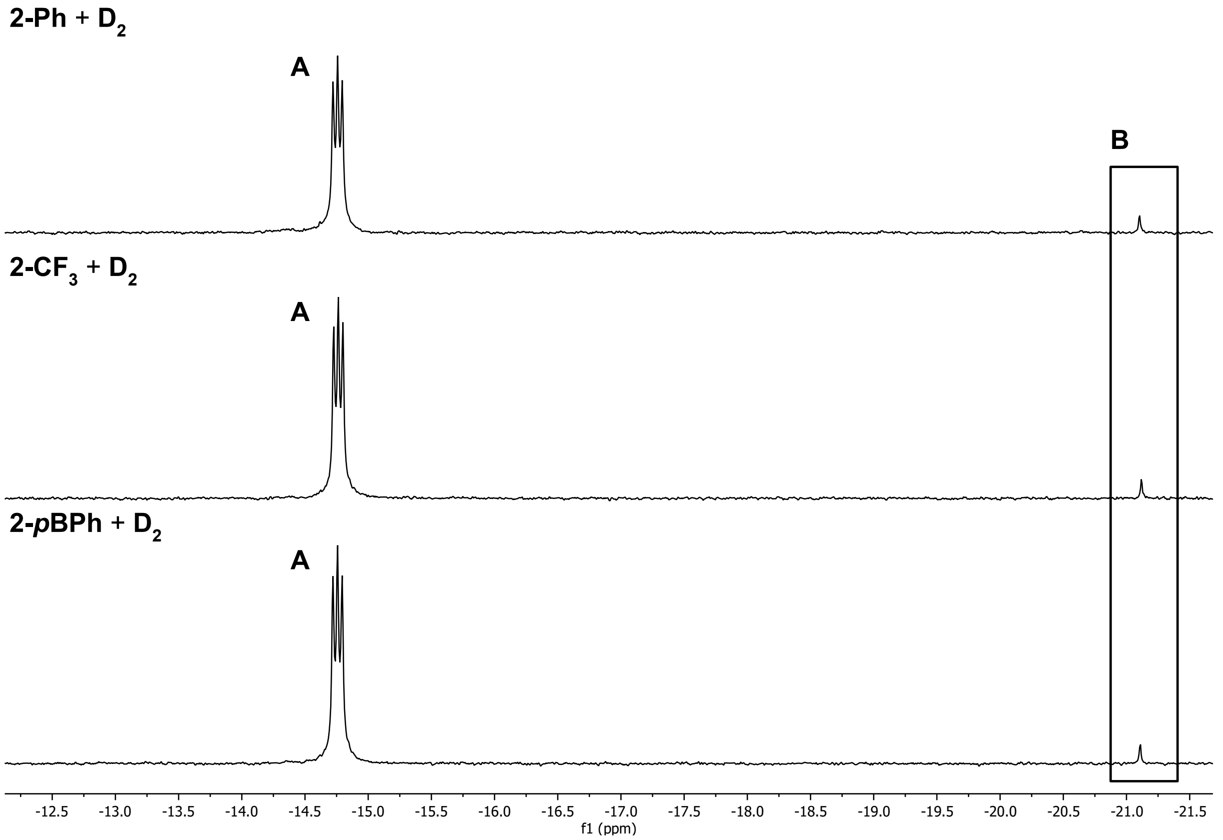

Figure S82. $^{31}P\{^{1}H\}$ NMR spectra (162 MHz, toluene-d_8, 298K) of **2-Ph, 2-*p*BP, 2-CF_3** pressurized with D_2 after 16 h. **A** shows **5** at -14.93 ppm and **B** shows the unidentified species **A** reacts to over time.

For more in depth comparison, additional samples were prepared in toluene-d_8, and VT-NMR spectroscopic data was collected, again for all compounds (*i.e.* **2-Ph, 2-CF_3, 2-*p*BP**; 30.0 mg). First, ^{1}H NMR and ^{31}P NMR spectra again confirm the same species is made in all cases (**Figures S81** and **S82**). No discernable differences are observed for the corresponding species generated from D_2 at -60 °C (**Figure S83**). Finally, no high-field signals are observed at -60 °C for the reaction with H_2 (**Figures S84-S86**), indicating that a terminal Ni-H is likely not formed.

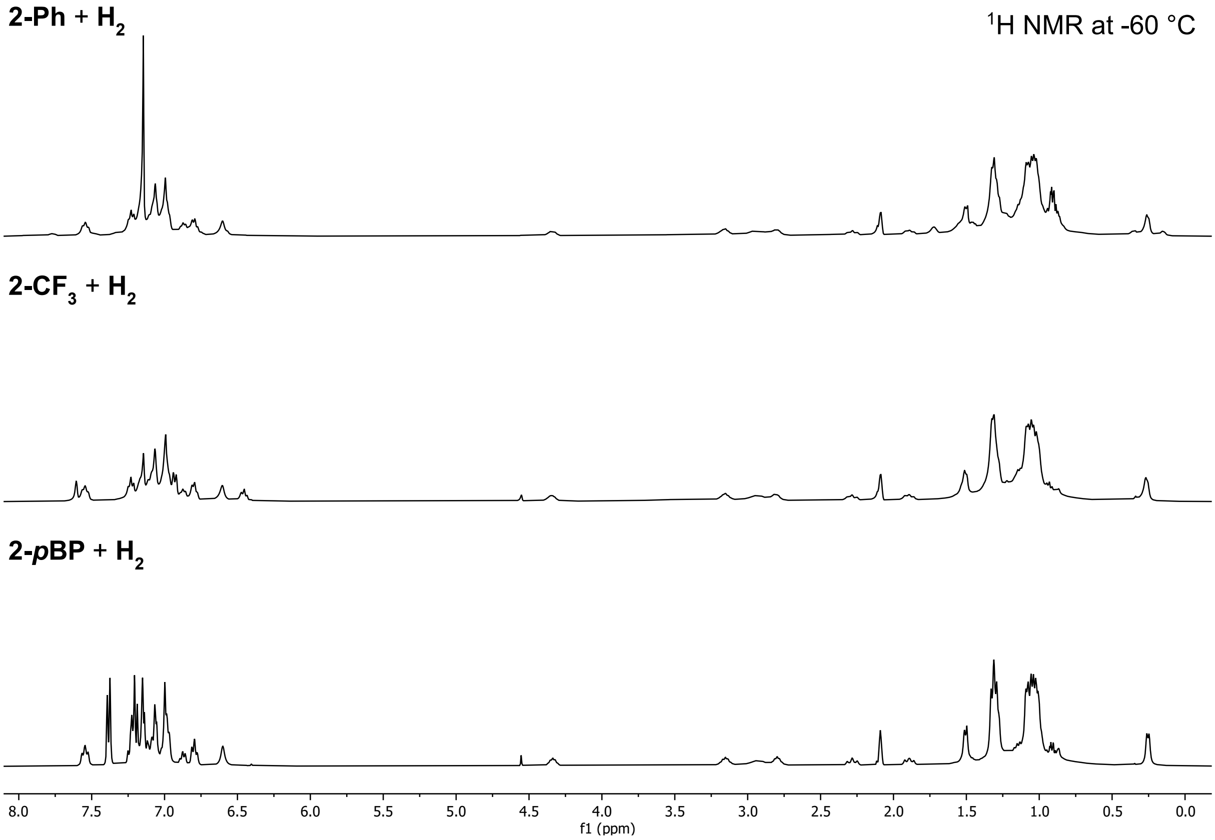

Figure S83. ^{1}H NMR spectra (400 MHz, toluene-d_8, 213K) of **2-Ph, -*p*BP, -CF$_3$** pressurized with H_2 after 16 h.

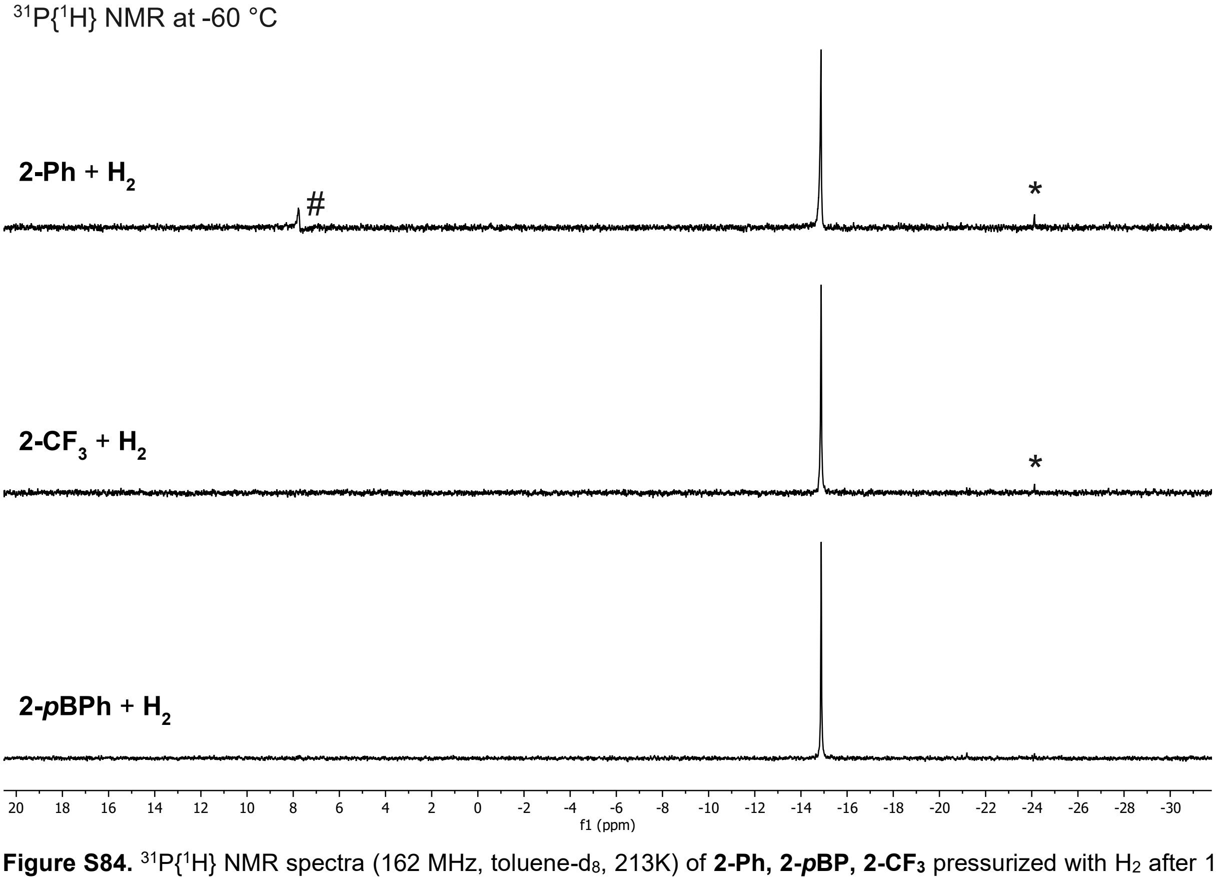

Figure S84. $^{31}P\{^{1}H\}$ NMR spectra (162 MHz, toluene-d_8, 213K) of **2-Ph, 2-*p*BP, 2-CF$_3$** pressurized with H_2 after 16 h. **5** is seen at -14.93 ppm, # shows educt, and * shows ^{Ph}LH.

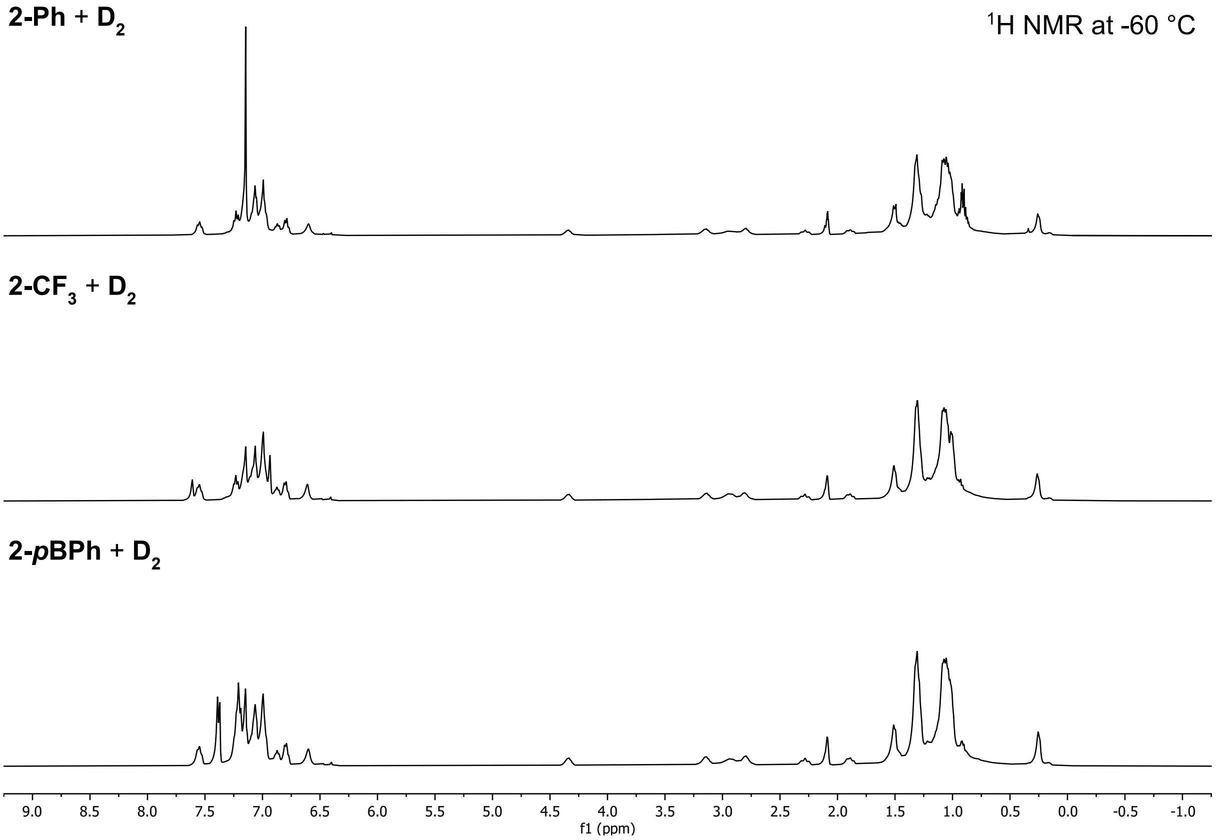

Figure S85. ^{1}H NMR spectra (162 MHz, toluene-d_8, 213K) of **2-Ph**, **2-*p*BP**, **2-CF3** pressurized with D_2 after 16 h, measured at -60 °C.

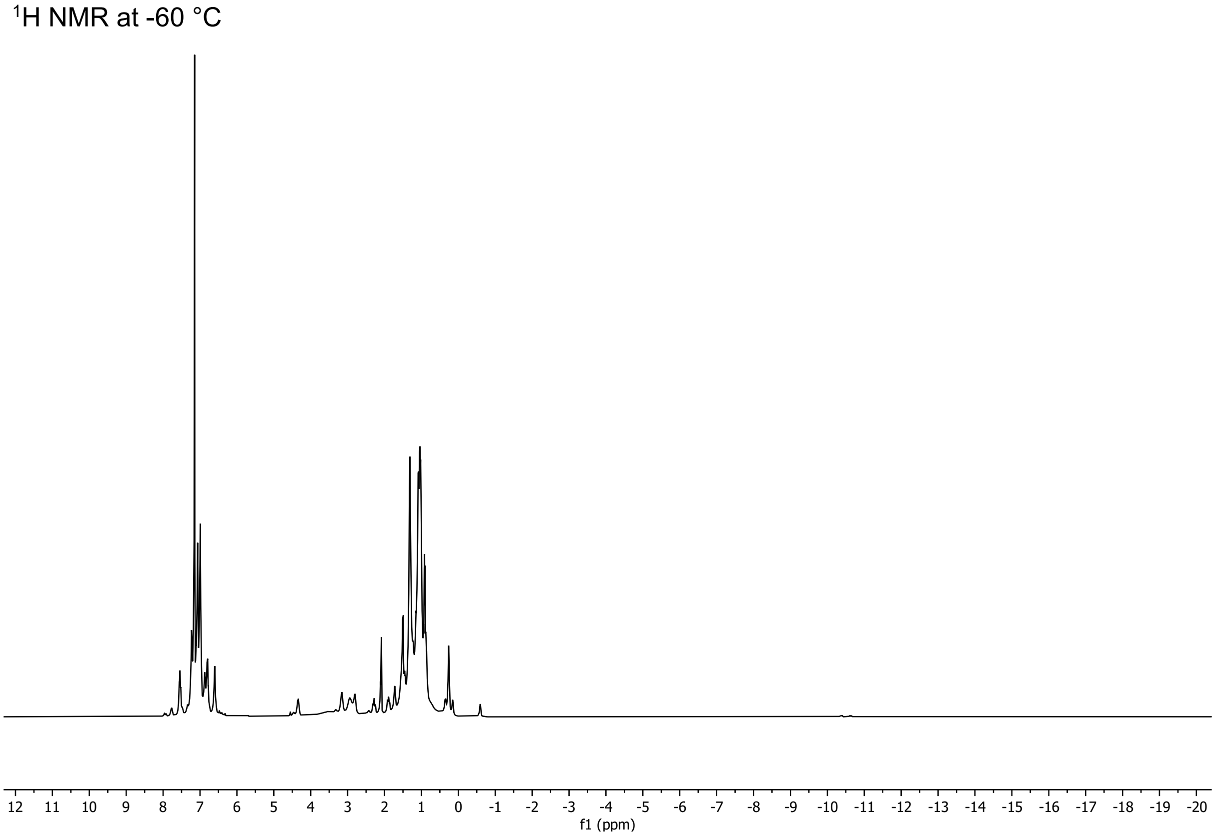

Figure S86. ^{1}H NMR spectrum (400 MHz, toluene-d_8, 213K) of **2-Ph** pressurized with H_2 after 16h, measured at -60 °C.

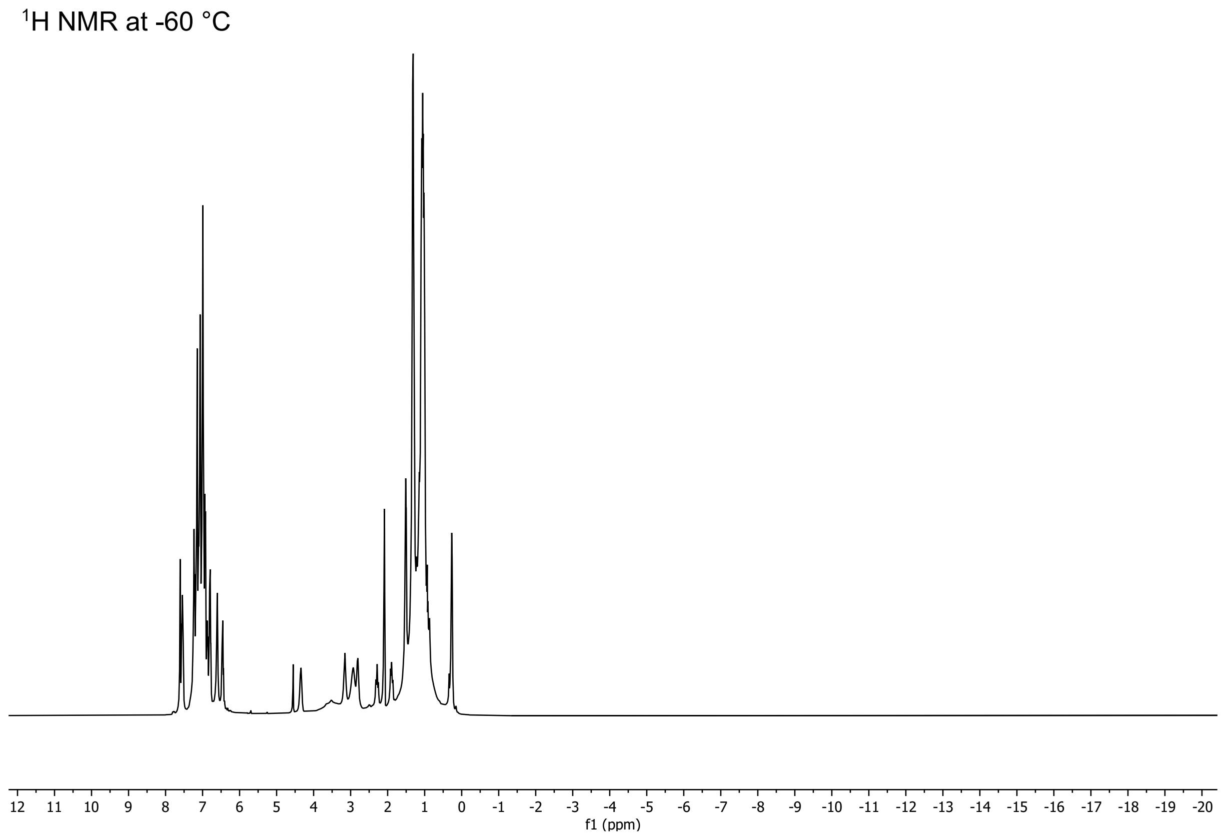

Figure S87. ^1H NMR spectrum (400 MHz, toluene-d_8, 213K) of **2-CF_3** pressurized with H_2 after 16h, measured at -60 °C.

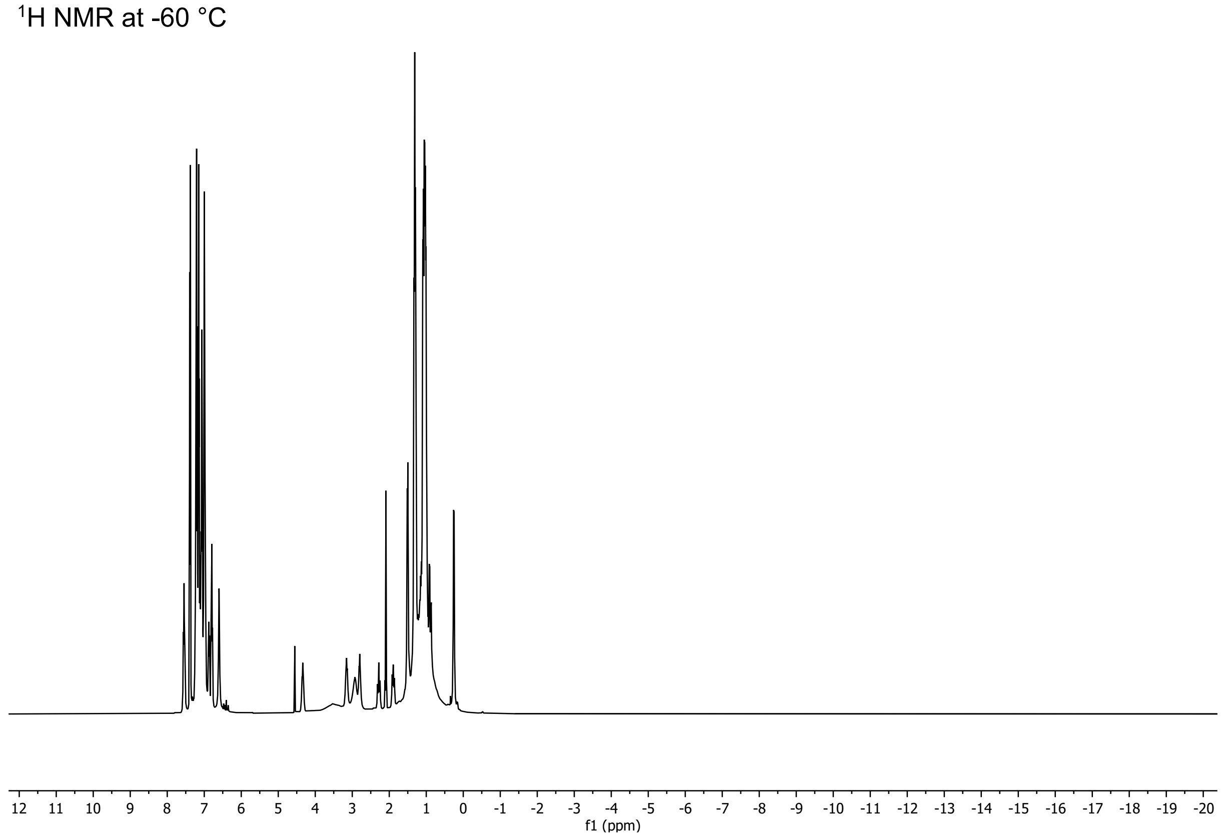

Figure S88. ^1H NMR spectrum (400 MHz, toluene-d_8, 213K) of **2-*p*BP** pressurized with H_2 after 16h, measured at -60 °C.

No crystalline material could be obtained from these reactions. We therefore utilised *(a)* high-res mass spectroscopy to identify the species formed following H_2 activation, and *(b)* ^{19}F NMR and mass spectroscopies to identify the eliminated aryl fragment using, both using compound **2-CF₃**.

(a) Firstly, a sample of **2-CF₃** (20.0 mg, 15.8 µmol) was treated with H_2 pressure (1.5 bar) in C_6D_6 according to general method, and analysed by LIFDI-MS after 5 h. Three clear species are observed: **2-CF₃** (1267.4368 m/z), [**2-CF₃** - (C_6H_5) - ($C_8H_3F_6$)] (977.3737 m/z; *viz*. **7**) and [**2-CF₃** - ($C_8H_3F_6$) + H] (1055.4262 m/z; *viz*. **5**) (**Figure S89**). Prolonging the reaction time up to 16 h lead to the same result besides the full conversion of **2-CF₃**. This suggests Ar-H elimination.

(b) Following the above, the quantitative formation of *m*-$(CF_3)_2$-C_6H_4D/H through reaction of **2-CF₃** with D_2/H_2 was shown. Two independent samples of **2-CF₃** (20.0 mg, 15.8 µmol) were dissolved in toluene (0.5 mL) and treated with D_2 or H_2 pressure (1.5 bar) in J-Young NMR tubes. ^{19}F NMR analysis indicated the clean formation of *m*-$(CF_3)_2$-C_6H_3D or *m*-$(CF_3)_2$-C_6H_4, but comparison with a commercial sample (**Figure S90**).

Both protonation and deuteration of the aryl was then confirmed using mass spectroscopy. The above described samples were quenched with H_2O (for the D_2 reaction) and D_2O (for the H_2 reaction), filtered over neutral Al_2O_3. Analysis by GC-MS clearly shows that the sample generated from D_2 contains *m*-$(CF_3)_2$-$C_6H_3\mathit{D}$, whilst the sample generated from H_2 contains *m*-$(CF_3)_2$-$C_6\mathit{H}_4$ (**Figure S91** and **Figure S92**).

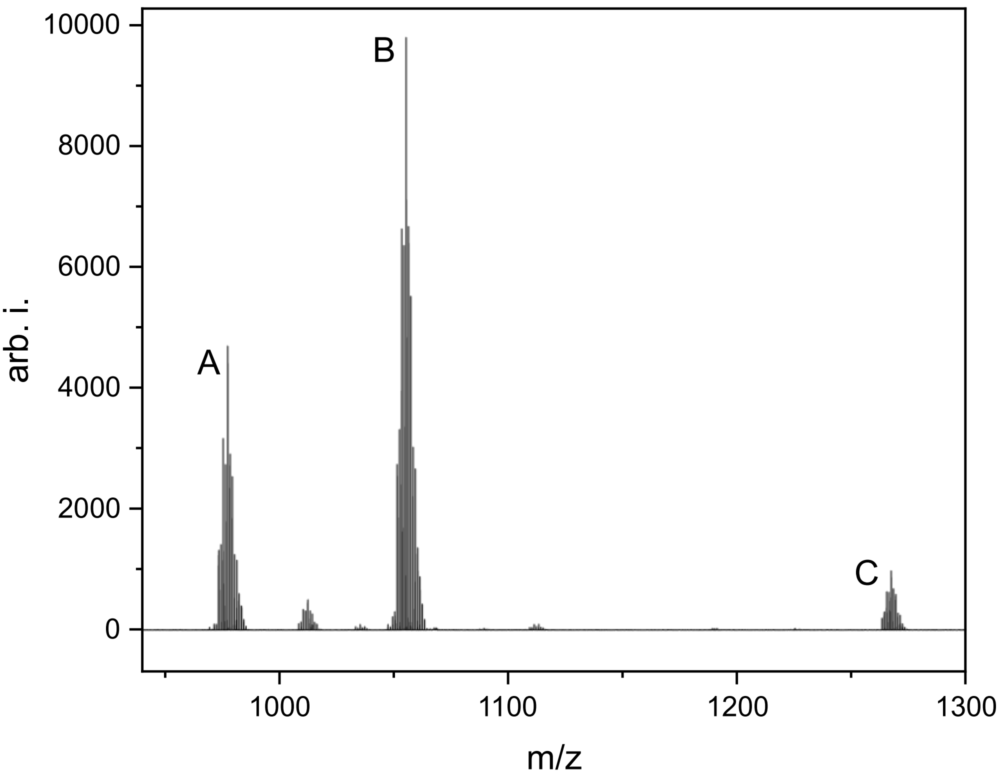

Figure S89. LIFDI-MS of **2-CF$_3$** reacting with H_2 for 5 h. **A** shows [**2-CF$_3$** - (C_6H_5) - ($C_8H_3F_6$)] (*i.e.* **7**) at 977.3737 m/z, **B** shows [**2-CF$_3$** - ($C_8H_3F_6$) + H] (*i.e.* **5**) at 1055.4262 m/z, and **C** shows **2-CF$_3$** at 1267.4368 m/z.

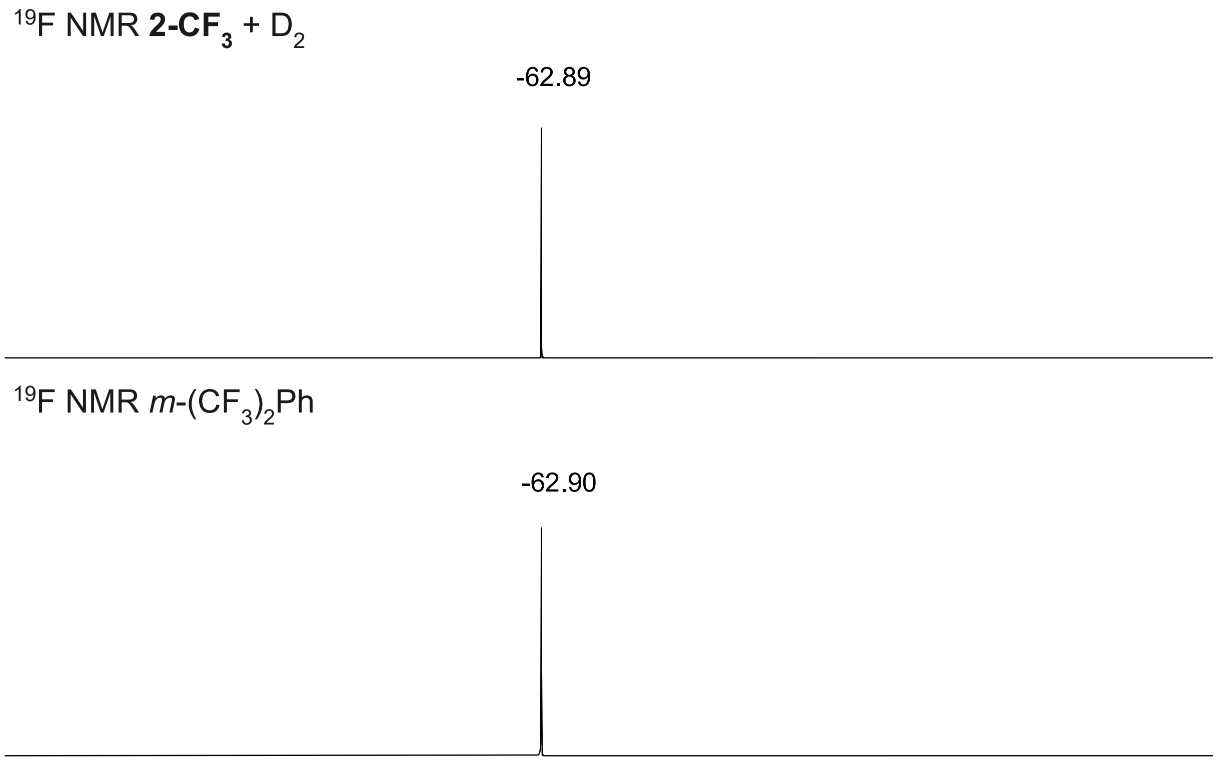

Figure S90. ^{19}F NMR spectra (376 MHz, toluene-h_8, 298K) of Top: **2-CF$_3$** + D_2; Bottom: *m*-(CF_3)$_2$-Ph.

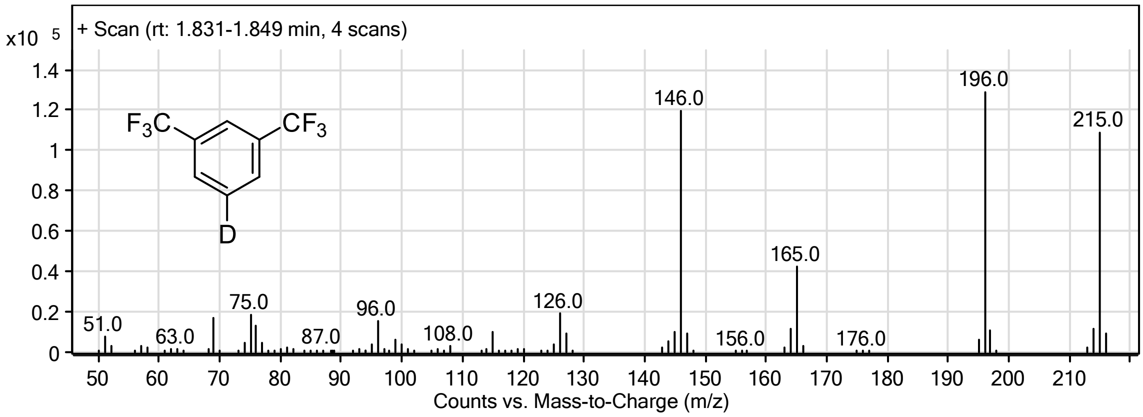

Figure S91. GC-MS spectrum (1.832-1.849 min retention time) of **2-CF₃** pressurized with D_2 in toluene-h_8, after 16h quenched with H_2O. *m*-$(CF_3)_2$-Ph-D can be identified as the pattern at 215.0 m/z.

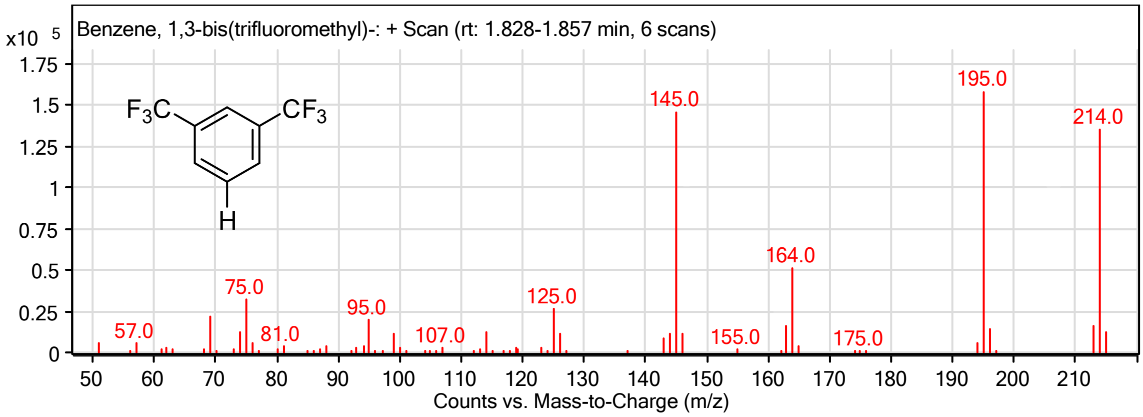

Figure S92. GC-MS spectrum (1.832-1.849 min retention time) of **2-CF₃** pressurized with H_2 in toluene-d_8, after 16h quenched with D_2O. *m*-$(CF_3)_2$-Ph-H can be identified as the pattern at 214.0 m/z.

The above data, indicating the loss of Ar-H, and the observation of the LIFDI-MS spectrum of reaction mixtures (**Figure S89**) aligns with the common product being ^{Ph}LSn(μ-H)Ni·IPr **5**. With this, we re-assessed ^{1}H NMR spectra of reactions between compounds **2** and H_2/D_2. This shows the absence of the hydride when pressurized with D_2. A shouldered doublet with a large J-coupling can be observed in solutions of **5** generated from H_2 at 2.12 ppm. This is absent in the reaction using D_2 (**Figure S93**).

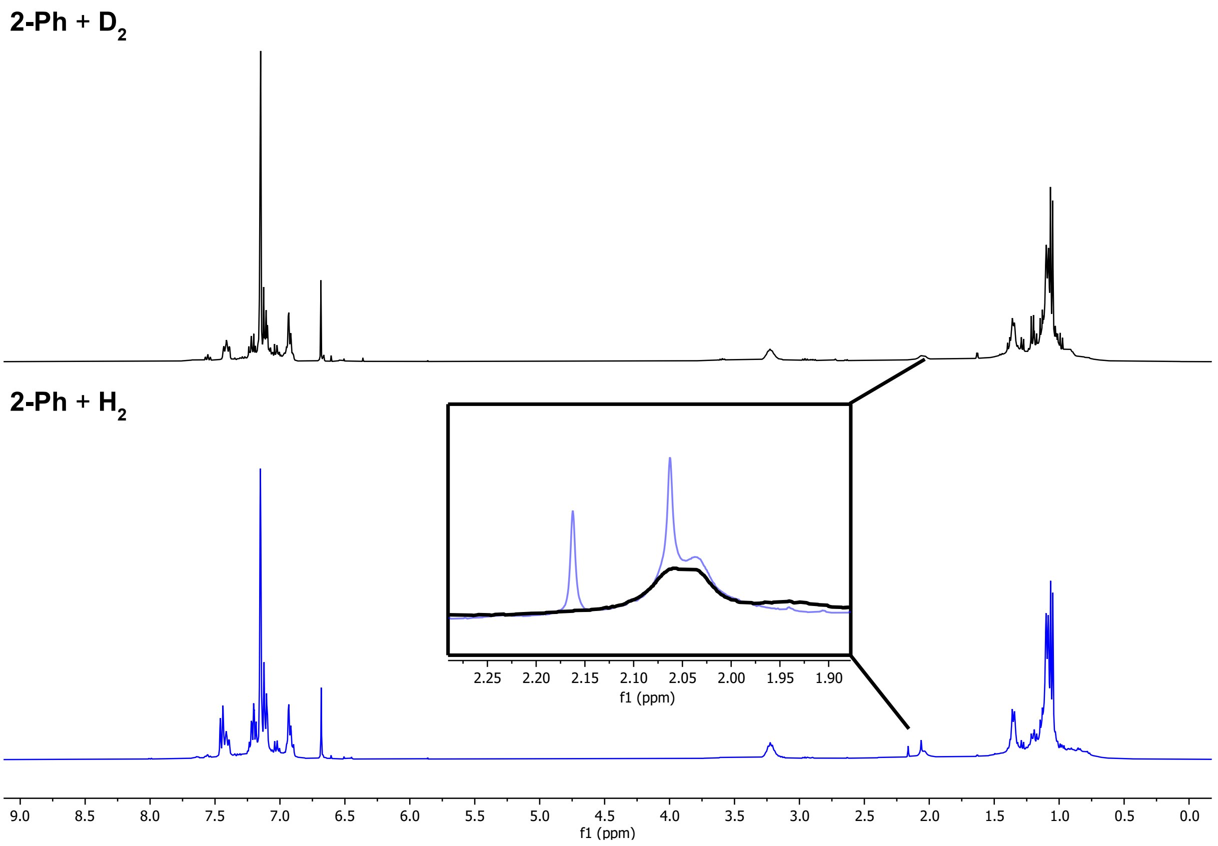

Figure S93. *Top (black)*: ^1H NMR spectrum (400 MHz, C_6D_6, 298K) of **2-Ph** pressurized with D_2 after 16h according to the general method.
Bottom (*blue*): ^1H NMR spectrum (400 MHz, C_6D_6, 298K) of **2-Ph** pressurized with H_2 after 16h according to the general method.
Inset: superimposed spectra at the region of the metal hydride.

To further confirm this, compound **5** was directly accessed through the addition of our previously reported Sn^{II} hydride compound $^{Ph}L(H)Sn$: (50.0 mg, 0.82 mmol, 1.00 eq.) to IPr·Ni·(η^6-toluene) (44.3 mg, 0.82 mmol, 1.00 eq.). This leads to a species identical to that formed through hydrogenation of compounds **2** (*i.e.* **5**; **Figure S94** and **S95**). We note that the H_2 activation route is a more selective route than this hydride addition pathway.

Finally, as no crystal of structure of **5** could be obtained in our hands, the novel complex $^{Cy}LSn(\mu\text{-}H)Ni\cdot IPr$ (**6**) was synthesized through the addition of $^{Cy}L(H)Sn$: (139 mg, 0.22 mmol, 1.00 eq.) to IPr·Ni·(η^6-toluene) (121 mg, 0.22 mmol, 1.00 eq.), affording crystals suitable for SC-XRD. Importantly, **5** and **6** both exhibit similar hydride signals and J-coupling values in the ^1H NMR, centred at 2.13 ($^2J_{PH}$ = 39.8 Hz) and 3.33 ppm ($^2J_{PH}$ = 27.1 Hz), respectively. This supports that compound **5** has a bridging hydride ligand (*i.e.* Sn-(μ-H)-Ni) as seen in **6**.

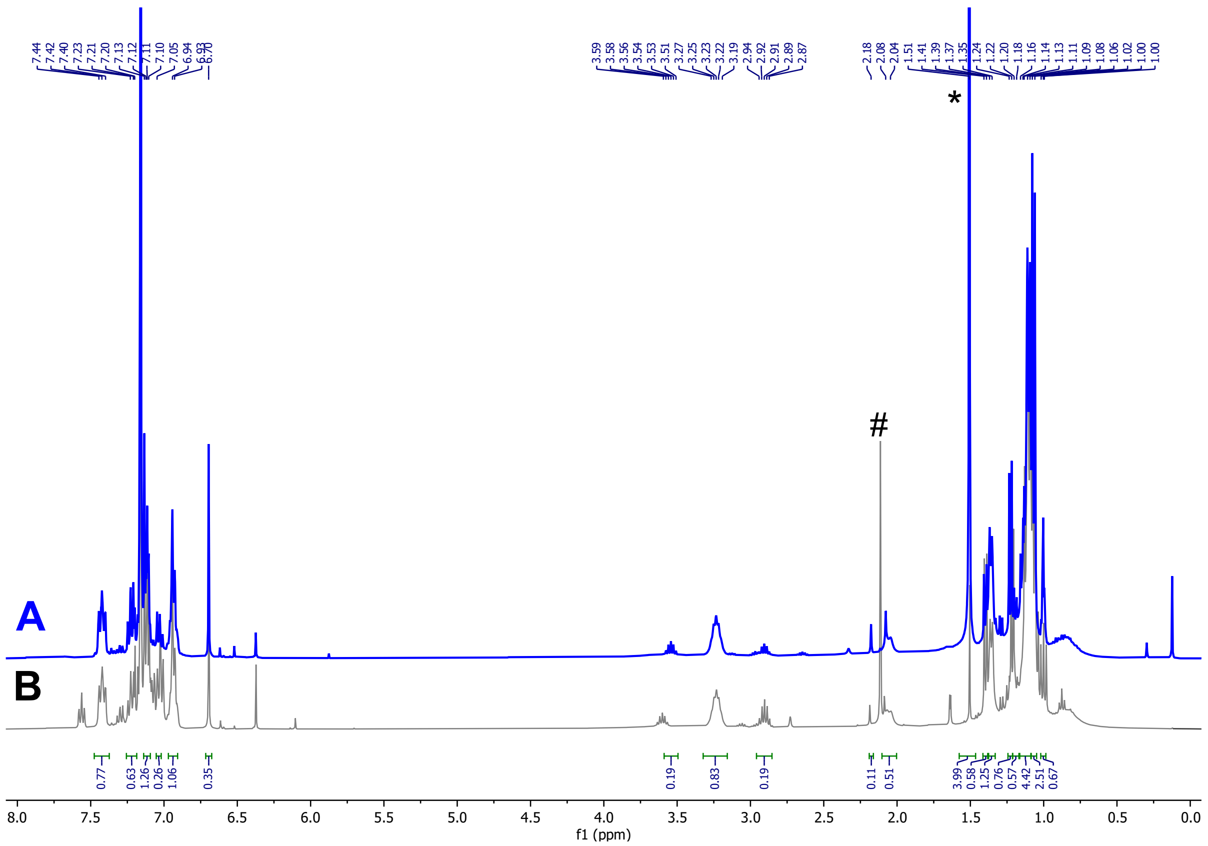

Figure S94. A: ^{1}H NMR spectrum (400 MHz, C_6D_6, 298K) of **2-Ph** pressurized with H_2 after 16h according to the general method. **B:** ^{1}H NMR spectrum (400 MHz, C_6D_6, 298K) of **5** sythesized *via* Method B. # marks toluene.

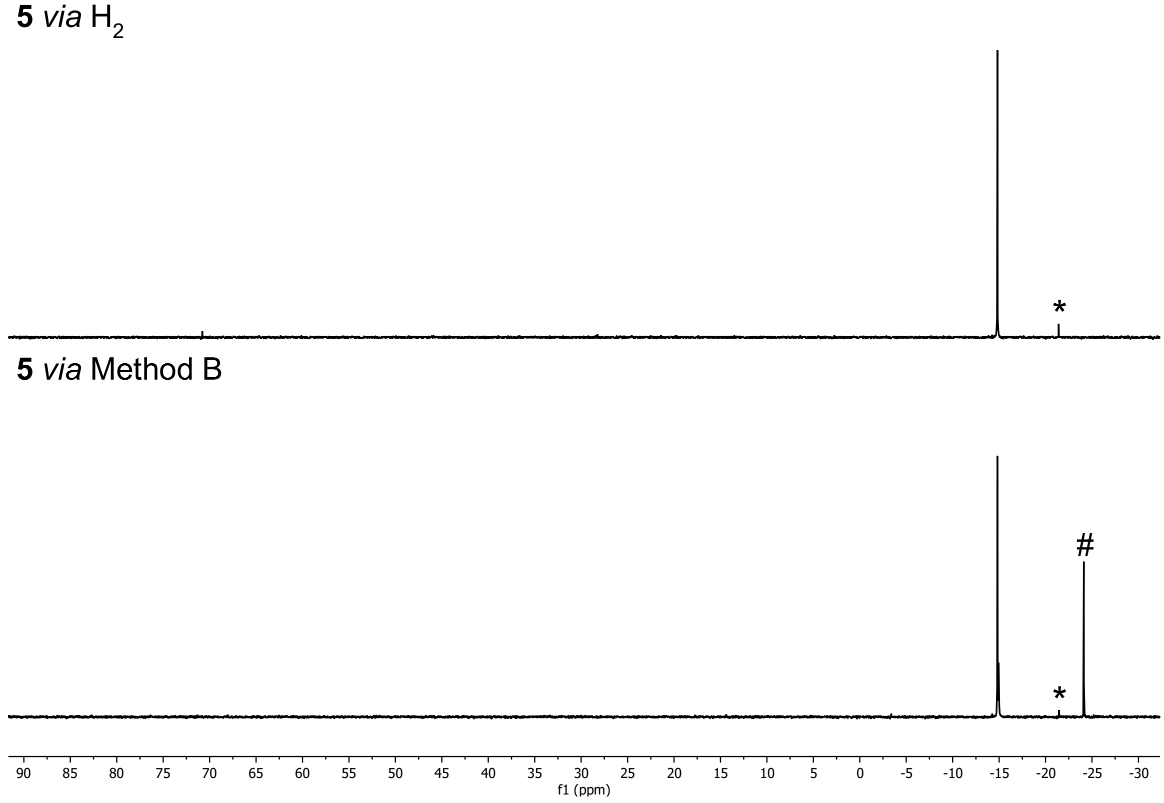

Figure S95. $^{31}P\{^1H\}$ NMR spectra (162 MHz, C_6D_6, 298K) of **5**. Top: **2-Ph** pressurized with H_2 after 16 h according to the general method. Bottom: reaction of $^{Ph}L(H)Sn$: and IPr·Ni·(η^6-toluene) (Methode B). * marks the species **5** reacts to over time and # shows ^{Ph}LH**.**

4. X-ray crystallographic details

Single crystals of **1a-Ph**, **1a-CF$_3$**, **1b-Ph**, **1b-*p*BP**, **2-Ph**, **2-*p*BP**, **3-CF$_3$**, **4**, **6**, and **7** suitable for X-ray structural analysis were mounted in perfluoroalkyl ether oil on a nylon loop and positioned in a 150 K cold N2 gas stream. Data collection was performed with a STOE StadiVari diffractometer (MoKα radiation) equipped with a DECTRIS PILATUS 300K detector. Structures were solved by Direct Methods (SHELXS-97),[10] or using SHELXT-16,[11] and refined by full-matrix least-squares calculations against F2 (SHELXL-2018).[12] The positions of the hydrogen atoms were calculated and refined using a riding model. All non-hydrogen atoms were treated with anisotropic displacement parameters. Crystal data, details of data collections, and refinements for all structures can be found in their CIF files, which are available free of charge *via* www.ccdc.cam.ac.uk/data_request/cif, and are summarized in Table S1 and S2.

Table S1. Summary of X-ray crystallographic data for compounds **1a-Ph**, **1a-CF$_3$**, **1b-Ph**, **1b-*p*BP** and **2-Ph**.

	**1a-Ph**	**1a-CF$_3$**	**1b-Ph**	**1b-*p*BP**	**2-Ph**
empirical form.	$C_{37}H_{48}NPSiSn$	$C_{39}H_{46}F_6NPSiSn$	$C_{37}H_{60}NPSiSn$	$C_{43}H_{64}NPSiSn$, $0.5(C_4H_{10}O)$	$C_{64}H_{84}N_3NiPSiSn$
formula wt	684.51	820.52	696.61	809.76	1131.80
crystal syst.	orthorhombic	monoclinic	triclinic	monoclinic	monoclinic
space group	*Pbca*	$P2_1/n$	*P*1	$P2_1/n$	$P2_1/c$
a (Å)	12.140(2)	13.802(3)	10.302(2)	15.824(3)	45.000(9)
b (Å)	15.050(3)	15.016(3)	11.250(2)	17.408(4)	13.690(3)
c (Å)	38.530(8)	19.310(4)	16.905(3)	17.563(4)	21.360(4)
α (deg.)	90	90	79.85(3)	90	90
β (δεγ)	90	106.58(3)	80.12(3)	111.74(3)	92.60(3)
γ (deg.)	90	90	74.86(3)	90	90
vol (Å^3)	7040(2)	3835.7(15)	1845.4(7)	4493.8(18)	13145(5)
Z	8	4	2	4	8
ρ(calc) (g.cm^{-3})	1.292	1.421	1.254	1.197	1.144
μ (mm^{-1})	0.830	0.796	0.793	0.661	0.745
F(000)	2848	1680	736	1716	4768
T (K)	150(2)	150(2)	150(2)	150(2)	150(2)
reflns collect.	45522	46271	18204	53775	83694
unique reflns	6905	7516	11432	8837	12891
R_{int}	0.0603	0.0243	0.0559	0.0279	0.0951
R1 [$I>2\sigma(I)$]	0.0665	0.0488	0.0367	0.0683	0.0520
*w*R2 (all data)	0.1945	0.1363	0.0784	0.1793	0.1350
CCDC No.	2492619	2492620	2492621	2492622	2492623

Table S2. Summary of X-ray crystallographic data for compounds **2-*p*BP**, **3-CF_3**, **4**, **6**, and **7**.

	**2-*p*BP**	**3-CF_3**	**4**	**6**	**7**
empirical form.	$C_{80}H_{112}N_3NiPSiSn$	$C_{78}H_{92}F_{12}N_2NiP_2Si_2Sn_2$	$C_{51}H_{64}NNiPSiSn$	$C_{58}H_{92}N_3NiPSiSn$	$C_{77}H_{112}N_4Ni_2P_2Si_2Sn_2$
formula wt	1352.18	1699.74	927.49	1067.80	1566.62
crystal syst.	triclinic	trigonal	tetragonal	monoclinic	monoclinic
space group	*P-1*	*R-3c*	*P1*	$P2_1/n$	$P2_1/c$
a (Å)	12.840(3)	33.475(5)	28.920(4)	12.766(3)	16.051(3)
b (Å)	13.880(3)	33.475(5)	28.920(4)	19.113(4)	26.835(5)
c (Å)	21.998(4)	43.630(9)	11.610(2)	23.772(5)	19.363(4)
α (deg.)	84.37(3)	90	90	90	90
β (δεγ)	81.89(3)	90	90	93.27(3)	97.22(3)
γ (deg.)	77.88(3)	120	90	90	90
vol (Å^3)	3785.3(14)	42341(15)	9710(3)	5791(2)	8274(3)
Z	2	18	8	4	4
ρ(calc) (g.cm^{-3})	1.186	1.200	1.269	1.225	1.258
μ (mm^{-1})	0.658	0.844	0.992	0.842	1.152
F(000)	1440	15624	3872	2272	3264
T (K)	150(2)	150(2)	150(2)	150(2)	150(2)
reflns collect.	50602	55017	69769	78166	106166
unique reflns	14872	9242	9541	11378	16230
R_{int}	0.0981	0.0423	0.0366	0.0224	0.0423
R1 [$I>2\sigma(I)$]	0.0658	0.0458	0.0342	0.0295	0.0618
*w*R2 (all data)	0.1802	0.1206	0.0861	0.0704	0.1656
CCDC No.	2492624	2492625	2492626	2492627	2492628

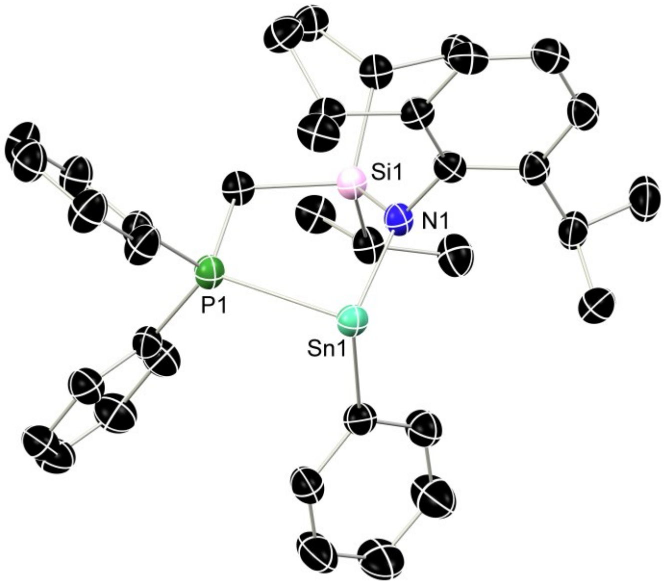

Figure S96. The molecular structure of **1a-Ph**, with thermal ellipsoids at 30% probability, and H atoms omitted for clarity.

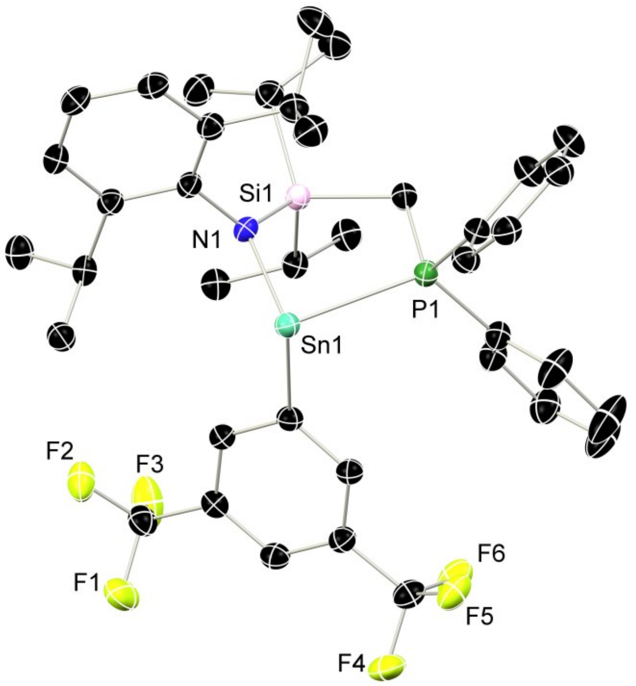

Figure S97. The molecular structure of **1a-CF₃**, with thermal ellipsoids at 30% probability, and hydrogen atoms omitted for clarity.

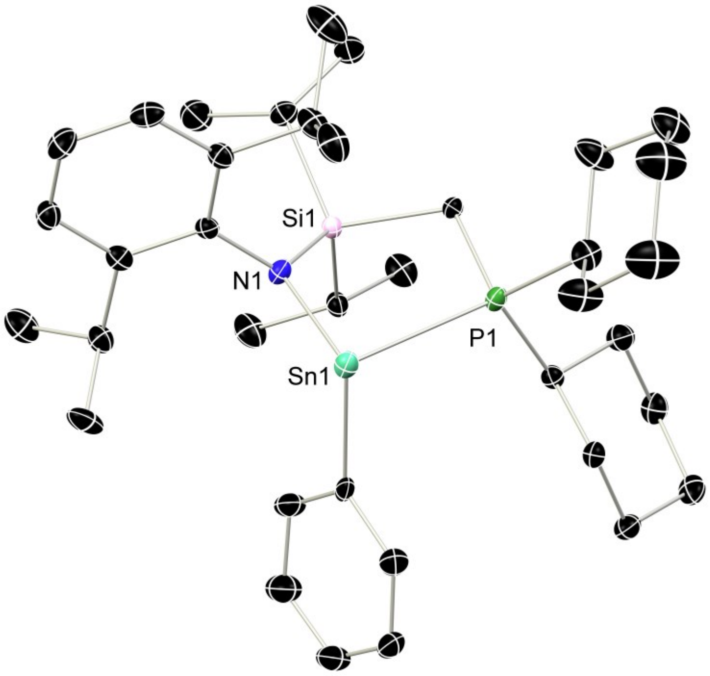

Figure S98. The molecular structure of **1b-Ph**, with thermal ellipsoids at 30% probability, and hydrogen atoms omitted for clarity.

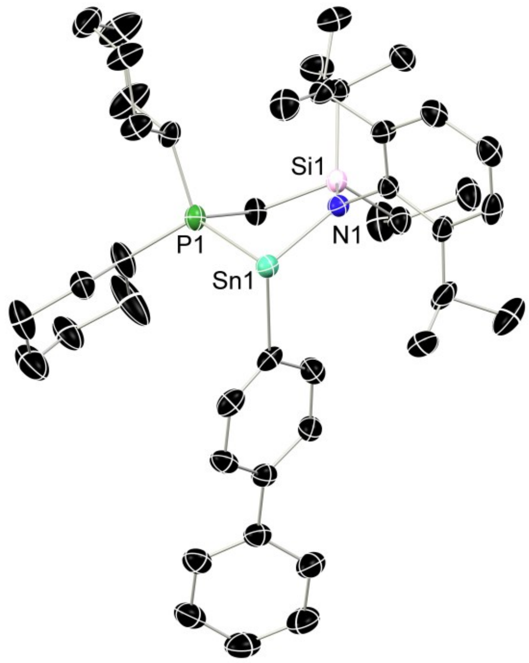

Figure S99. The molecular structure of **1b-*p*BP**, with thermal ellipsoids at 30% probability, and hydrogen atoms omitted for clarity.

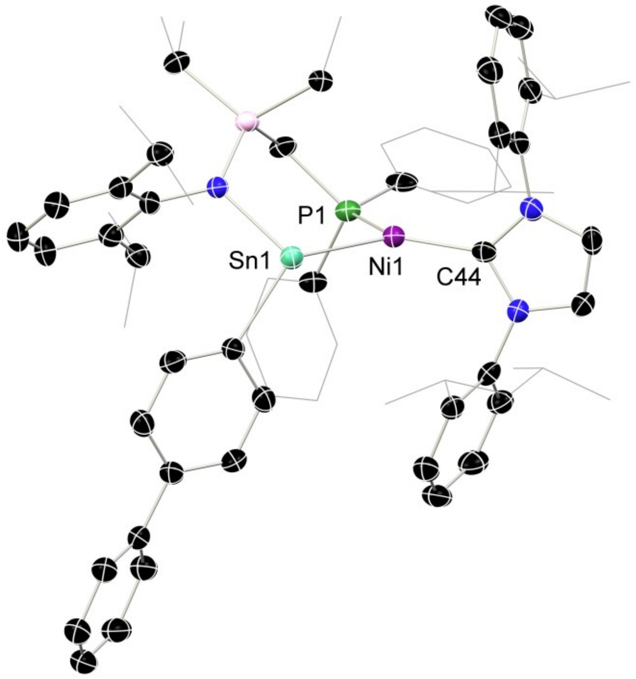

Figure S100. The molecular structure of **2-*p*BP**, with thermal ellipsoids at 30% probability, and hydrogen atoms omitted for clarity.

5. Computational methods and details

Computational Methods

We carried out all density functional theory (DFT) calculations with Gaussian 16[10] at the BP86-D3(SMD=Benzene)/def2-TZVP//B97-D3/def2-SVP[11–13] level of theory. To correct the Gibbs free energies from gas phase standard state to liquid phase standard state, an energy correction of 1.89 kcal/mol was applied to all the computed energies considering 298 K as reaction temperature.[14] Harmonic vibrational frequency calculations confirmed that the transition states had only one imaginary frequency, and the reactants, products and intermediates were local minima with no imaginary frequency. Intrinsic reaction coordinate (IRC) calculations validated the identified transition state structures and confirmed their connections to the associated minima. We performed Natural Bond Orbital (NBO) analysis using the NBO utility program (version 3.1)[15] in Gaussian 16. To generate molecular orbital surfaces, we employed an isovalue of 0.03 in Avogadro software (version 1.2.0)[16,17]. Time-dependent density functional theory (TD-DFT) excited state singlepoint calculations were performed at BP86-D3(SMD=Benzene)/def2-TZVP level of theory on DFT-optimized structures.

Optimized Structures

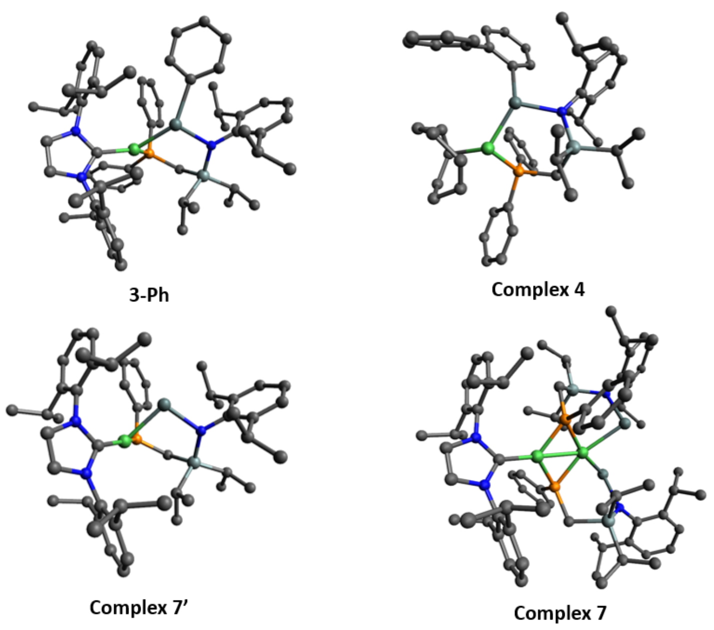

Figure S101. Optimized geometries for complexes **2-Ph**, **4**, **5**, **7’** and **7**. Hydrogen atoms are omitted for clarity, except for complex **5** due to the presence of an important bridging **Sn··H··Ni** coordination. Color code: C – gray, Sn – teal, N – blue, Ni – green, P – orange.

Electronic Structure Analysis

Table S3. Calculated bond length [Å], NPA, Wiberg Bond Index (WBI), and Mayer Bond Order (MBO) in complex **2-Ph**.

**Property**	**2-Ph**	
**Bond length [Å]**	Ni-Sn	2.408
**NPA charge**	Ni/Sn	-0.772/2.127
**Wiberg Bond Index**	Ni-Sn	0.737
**Mayer Bond Order**	Ni-Sn	1.023

Table S4. Calculated bond length [Å], NPA, WBI, and MBO in complex **4**.

**Property**	**Complex 4**	
**Bond length [Å]**	Ni-Sn	2.401
**NPA charge**	Ni/Sn	-0.872/1.737
**Wiberg Bond Index**	Ni-Sn	0.745
**Mayer Bond Order**	Ni-Sn	1.051

Table S5. Calculated bond length [Å], NPA, WBI, and MBO in complex **5**.

**Property**	**Complex 5**	
**Bond length [Å]**	Ni-Sn	2.509
**NPA charge**	Ni/Sn	-0.812/1.083
**Wiberg Bond Index**	Ni-Sn	0.501
**Mayer Bond Order**	Ni-Sn	0.576

Table S6. Calculated bond length [Å], NPA, WBI, and MBO in complex **7’**.

**Property**	**Complex 7’**	
**Bond length [Å]**	Ni-Sn	2.428
**NPA charge**	Ni/Sn	-0.646/1.184
**Wiberg Bond Index**	Ni-Sn	0.534
**Mayer Bond Order**	Ni-Sn	0.887

Table S7. Calculated bond length [Å], NPA, WBI, and MBO in complex **7**.

**Property**	**Complex 7**	
**Bond length [Å]**	Ni-Sn	2.566 (2.590)
**NPA charge**	Ni/Sn	-1.441/1.111 (-0.326/1.135)
**Wiberg Bond Index**	Ni-Sn	0.530 (0.509)
**Mayer Bond Order**	Ni-Sn	0.615 (0.638)

HOMO-LUMO Analysis

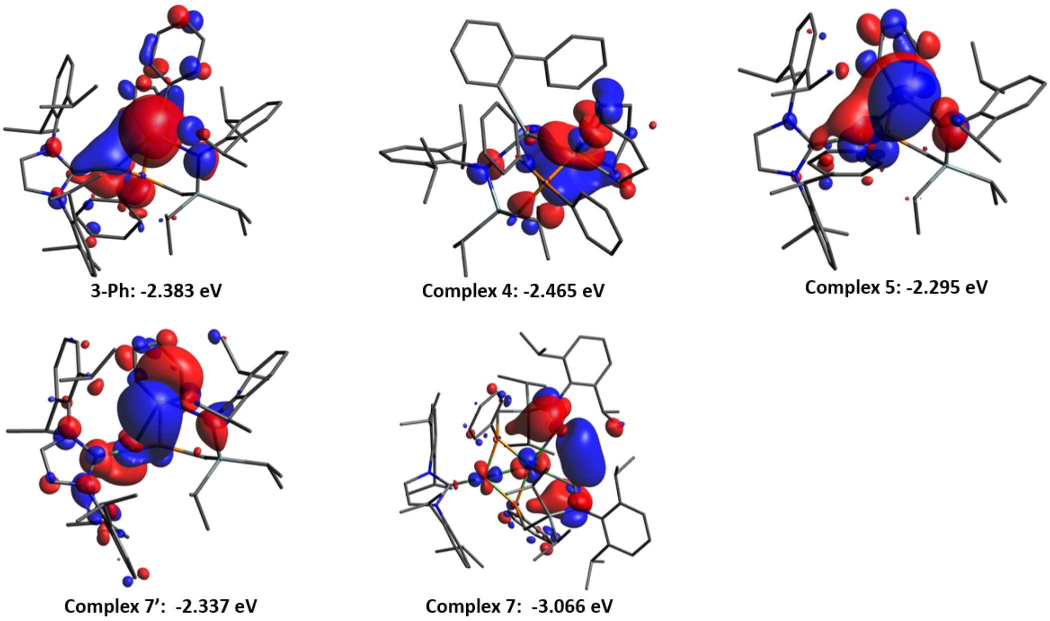

Figure S102. Lowest unoccupied molecular orbital (LUMO) of complexes **3-Ph**, **4**, **5**, **7’** and **7**. Orbital energies: **3-Ph**: -2.383 eV, complex **4**: -2.465 eV, complex **5**: -2.295 eV, complex **7’**: -2.337 eV, and complex **7**: -3.066 eV.

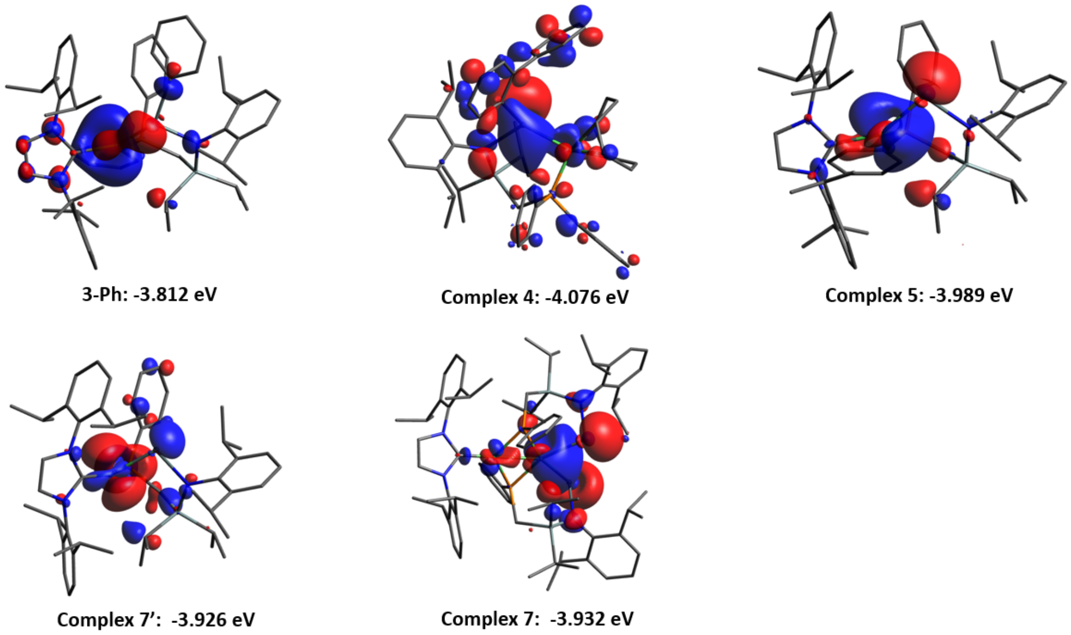

Figure S103. Highest occupied molecular orbital (HOMO) of complexes **3-Ph**, **4**, **5**, **7’** and **7**. Orbital energies (eV): **3-Ph**: -3.812 eV, complex **4**: -4.076 eV, complex **5**: -3.989 eV, complex **7’**: -3.926 eV, and complex **7**: -3.932 eV.

Natural Bond Order (NBO) Analysis

Table S8. NBO analysis of the Ni-Sn moiety of complex **2-Ph**.

Alpha orbitals	Occupation	Atom	Polarization	s-character	p-character	d-character
Bond	1.81	Ni	19.79%	33.47%	63.74%	2.78%
		P	80.21%	38.22%	61.68%	0.10%
Bond	1.87	Ni	17.89%	40.09%	54.94%	4.96%
		C	82.11%	49.54%	50.43%	0.02%
Lone Pair	1.87	Ni	19.42%	22.28%	76.51%	1.20%
Lone Pair	1.98	Sn	80.58%	93.12%	6.83%	0.03%
Lone Pair	1.97	Ni	-	0.39%	0.02%	99.60%
Lone Pair	1.95	Ni	-	0.63%	0.07%	99.30%
Lone Pair	1.94	Ni	-	0.15%	0.29%	99.56%
Lone Pair	1.87	Ni	-	2.03%	1.51%	96.46%
Empty Orbital	1.78	Ni	-	0.43%	2.49%	97.08%
Empty Orbital	0.04	Ni	-	0.56%	96.07%	3.36%
Empty Orbital	0.32	Sn	-	5.29%	94.62%	0.04%
Empty Orbital	0.27	Sn	-	2.18%	97.67%	0.12%
Empty Orbital	0.18	Sn	-	0.49%	99.11%	0.34%

Table S9. NBO analysis of the Ni-Sn moiety of complex **4**.

Alpha orbitals	Occupation	Atom	Polarization	s-character	p-character	d-character
Bond	1.83	Ni	24.52%	24.01%	75.10%	0.89%
		Sn	75.48%	93.78%	6.15%	0.06%
Bond	1.78	Ni	22.67%	18.61%	79.20%	2.18%
		P	77.33%	37.82%	62.06%	0.12%
Lone Pair	1.97	Ni	-	0.03%	0.01%	99.95%
Lone Pair	1.95	Ni	-	0.02%	0.30%	99.68%
Lone Pair	1.87	Ni	-	0.02%	1.96%	98.03%
Lone Pair	1.81	Ni	-	0.03%	0.11%	99.85%

Lone Pair	1.75	Ni	-	0.15%	1.78%	98.06%
Empty Orbital	0.28	Ni	-	0.85%	96.06%	3.09%
Empty Orbital	0.23	Ni	-	56.04%	43.71%	0.25%
Empty Orbital	0.31	Sn	-	1.36%	98.55%	0.06%
Empty Orbital	0.29	Sn	-	4.41%	95.39%	0.14%
Empty Orbital	0.20	Sn	-	1.38%	98.28%	0.29%

Table S10. NBO analysis of the Ni-Sn moiety of complex **5**.

Alpha orbitals	Occupation	Atom	Polarization	s-character	p-character	d-character
Bond	1.88	Ni	20.29%	32.75%	61.85%	5.40%
		C	79.71%	49.41%	50.57%	0.02%
Bond	1.81	Ni	22.08%	35.46%	61.72%	2.82%
		P	77.92%	38.08%	61.80%	0.10%
Bond	1.62	Ni	28.84%	29.46%	65.35%	5.19%
		H	71.16%	99.96%	0.04%	0.00%
Lone Pair	1.93	Sn	-	89.54%	10.45%	0.01%
Lone Pair	1.97	Ni	-	0.19%	0.09%	99.71%
Lone Pair	1.95	Ni	-	1.08%	0.26%	98.66%
Lone Pair	1.94	Ni	-	0.14%	0.11%	99.75%
Lone Pair	1.84	Ni	-	0.19%	0.31%	99.50%
Lone Pair	1.74	Ni	-	0.59%	8.87%	90.53%
Empty Orbital	0.48	Sn	-	9.44%	90.40%	0.12%
Empty Orbital	0.27	Sn	-	0.03%	99.79%	0.13%
Empty Orbital	0.22	Sn	-	1.94%	97.83%	0.17%
Empty Orbital	0.06	Ni	-	0.19%	97.12%	2.69%

Table S11. NBO analysis of the Ni-Sn moiety of complex **7'**.

Alpha orbitals	Occupation	Atom	Polarization	s-character	p-character	d-character
Bond	1.84	Ni	18.64%	47.60%	45.15%	7.24%
		C	81.36%	48.49%	51.46%	0.04%
Bond	1.70	Ni	28.64%	32.59%	59.19%	8.21%
		P	71.36%	17.63%	82.08%	0.27%
Lone Pair	1.98	Ni	-	0.13%	0.14%	99.73%
Lone Pair	1.97	Ni	-	0.16%	0.09%	99.75%
Lone Pair	1.94	Ni	-	0.01%	0.15%	99.83%
Lone Pair	1.85	Ni	-	0.56%	0.40%	99.04%
Lone Pair	1.77	Ni	-	9.75%	3.98%	86.27%
Lone Pair	1.91	Sn	-	93.71%	6.28%	0.01%
Empty Orbital	0.19	Ni	-	8.74%	89.47%	1.79%
Empty Orbital	0.04	Ni	-	0.37%	97.42%	2.20%
Empty Orbital	0.43	Sn	-	6.12%	93.84%	0.03%
Empty Orbital	0.25	Sn	-	0.72%	99.12%	0.13%
Empty Orbital	0.19	Sn	-	1.73%	98.02%	0.20%

Table S12. NBO analysis of the Ni-Sn moiety of complex **7**.

Alpha orbitals	Occupation	Atom	Polarization	s-character	p-character	d-character
Bond	1.61	Ni	26.07%	29.53%	67.67%	2.80%
		P	73.93%	44.66%	55.06%	0.26%
Bond	1.62	Ni	26.55%	30.50%	66.50%	3.00%
		P	73.45%	43.72%	56.00%	0.27%
Bond	1.90	Ni	24.58%	47.55%	21.55%	30.90%
		C	75.42%	46.89%	53.07%	0.03%
Bond	1.73	Ni	30.44%	16.05%	50.79%	33.17%
		P	69.56%	10.42%	89.30%	0.27%
Bond	1.74	Ni	31.16%	15.12%	50.63%	34.24%
		P	68.84%	11.35%	88.38%	0.26%
Lone Pair	1.90	Sn	-	91.64%	8.35%	0.01%
Lone Pair	1.97	Ni	-	0.10%	0.08%	99.82%

Lone Pair	1.95	Ni	-	0.01%	0.08%	99.91%
Lone Pair	1.95	Ni	-	0.21%	0.01%	99.77%
Lone Pair	1.89	Ni	-	0.00%	3.96%	96.04%
Lone Pair	1.78	Ni	-	1.40%	0.37%	98.23%
Lone Pair	1.98	Ni	-	0.77%	0.21%	99.01%
Lone Pair	1.95	Ni	-	0.01%	0.00%	99.99%
Lone Pair	1.94	Ni	-	0.00%	0.13%	99.86%
Lone Pair	1.86	Ni	-	0.01%	0.14%	99.86%
Lone Pair	1.90	Sn	-	92.25%	7.74%	0.01%
Lone Pair	0.50	Sn	-	7.84%	92.04%	0.09%
Empty Orbital	0.27	Sn	-	0.82%	99.03%	0.13%
Empty Orbital	0.18	Sn	-	1.73%	97.95%	0.24%
Empty Orbital	0.82	Ni	-	37.70%	59.91%	2.39%
Empty Orbital	0.12	Ni	-	0.00%	98.67%	1.32%
Empty Orbital	0.31	Ni	-	20.46%	75.74%	3.80%
Empty Orbital	0.03	Ni	-	0.01%	99.04%	0.94%
Empty Orbital	0.49	Sn	-	7.67%	92.23%	0.08%
Empty Orbital	0.26	Sn	-	0.79%	99.09%	0.10%
Empty Orbital	0.17	Sn	-	1.69%	97.97%	0.25%

Calculated Pathway for Stannylene H_2 Activation

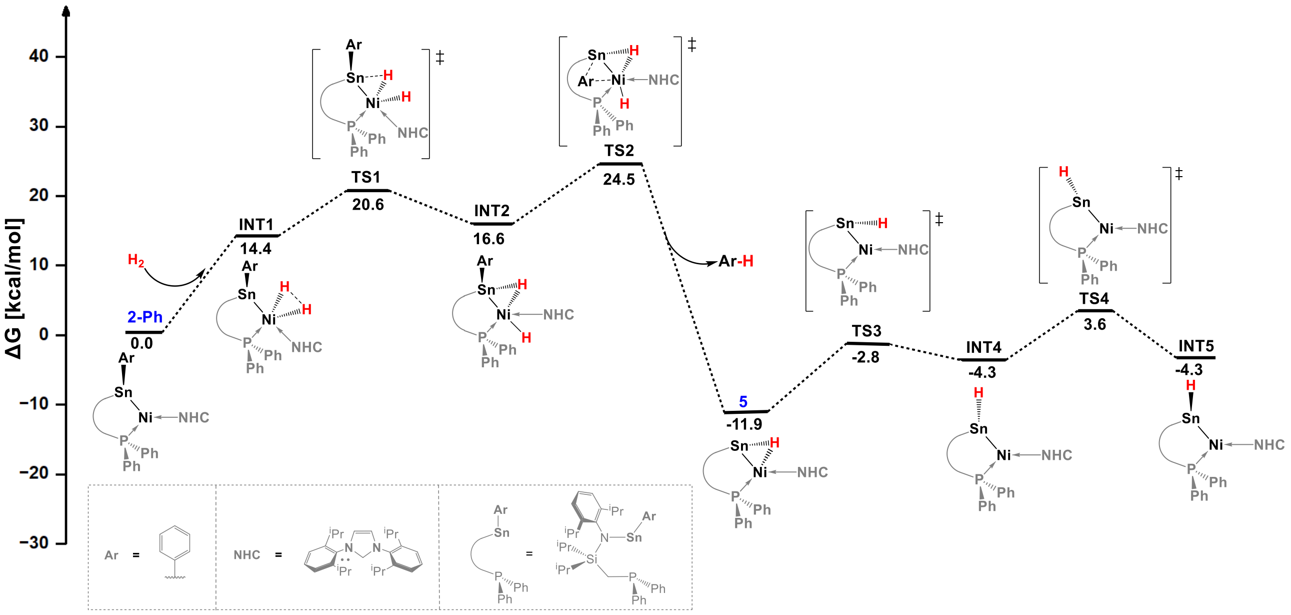

Figure S104. DFT-derived Gibbs free energy reaction profile for $\mathbf{H_2}$ activation of complex **2-Ph.** TS and INT denote transition state and intermediates, respectively.

Calculated Pathway for Benzene Elimination

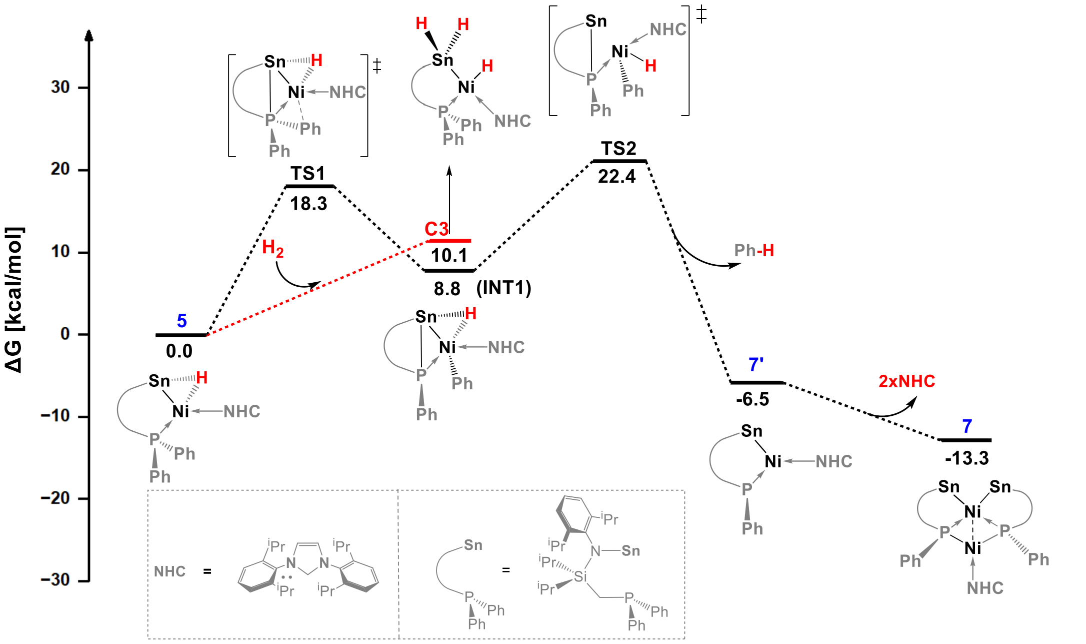

Figure S105. DFT-derived Gibbs free energy reaction profile for benzene elimination from complex **5,** yielding complex **7**, and complex **5** hydrogenation reaction towards stannylene-hydride formation (shown by the reaction coordinate in the dotted, red colored line). TS and INT denote transition state and intermediates, respectively.

Optimized Cartesian Coordinates of Reactants, Intermediates, Transition States, and Product

Table S13. Cartesian geometry of complex **2-Ph** in Figure S104 in Angstrom [Å].

Atomtype	X Coordinates	Y Coordinates	Z Coordinates
C	-0.13204	2.91505	1.19662
C	-0.22997	2.00297	2.26395
C	-0.73641	2.44568	3.50342
C	-1.15167	3.77688	3.66208
C	-1.06068	4.68	2.58528
C	-0.5497	4.24693	1.35185
P	0.42582	0.28716	1.9983
Ni	1.01129	-0.08435	-0.02046
C	2.62929	-0.04488	-0.94936
N	3.57262	-1.02761	-1.19641
C	4.67949	-0.53273	-1.89518
C	4.43796	0.79555	-2.11782
N	3.19043	1.07444	-1.55172
C	3.35779	-2.43145	-0.9715
C	2.97484	-3.22527	-2.08017
C	2.86339	-4.61626	-1.87829
C	3.11727	-5.18249	-0.62235
C	3.45221	-4.36499	0.46881
C	3.56995	-2.97	0.32163
C	2.47969	2.31164	-1.73531
C	1.56615	2.40815	-2.82189
C	0.92714	3.64558	-3.02375
C	1.1806	4.73894	-2.18047
C	2.07833	4.61436	-1.11316
C	2.74971	3.39827	-0.8702
C	2.60683	-2.59619	-3.42047
C	3.19546	-3.35064	-4.62369
C	3.89698	-2.04568	1.48669
C	3.5809	-2.64573	2.86118
C	1.309	1.22094	-3.74587
C	2.40143	1.09882	-4.82903
C	3.77158	3.28265	0.25324
C	3.29346	3.92441	1.5658
C	1.77266	0.34746	3.28756
C	3.02333	0.83982	2.86724
C	4.08166	0.98761	3.77526
C	3.90803	0.62009	5.12226
C	2.66819	0.11398	5.54949
C	1.60408	-0.01618	4.63944
C	-0.84223	-0.8114	2.79321
Si	-1.74158	-1.93385	1.52448
C	-0.30628	-2.99447	0.82689
C	-0.58111	-3.69189	-0.51025
C	-3.09113	-2.91431	2.4684
C	-2.74343	-3.20189	3.94019
N	-2.5291	-1.02476	0.2378
C	-3.92881	-0.83464	0.13819
C	-4.68018	-1.63976	-0.77687
C	-6.06273	-1.41745	-0.91531

C	-6.71503	-0.41919	-0.17511
C	-5.97459	0.37773	0.70745
C	-4.58794	0.19749	0.87405
C	-3.96384	-2.67457	-1.63736
C	-3.40505	-2.0423	-2.92695
C	-3.81349	1.12075	1.80576
C	-4.07999	0.77661	3.28366
C	-3.52996	-4.19229	1.73254
Sn	-1.19007	0.12252	-0.97437
C	-2.58762	1.71551	-1.61509
C	-3.93253	1.53939	-2.01126
C	-4.72607	2.64246	-2.37298
C	-4.19027	3.94223	-2.35127
C	-2.84767	4.13204	-1.98047
C	-2.05847	3.02556	-1.62353
C	-4.81739	-3.90727	-1.9723
C	-4.08926	2.60977	1.53493
C	0.19106	-3.99506	1.88372
C	1.07507	-2.45486	-3.52721
C	5.35837	-1.559	1.43487
C	-0.07996	1.24575	-4.40024
C	5.12831	3.87001	-0.18853
H	-0.79288	1.7508	4.35625
H	-1.55611	-0.22271	3.40096
H	-0.30952	-1.49738	3.48381
H	-2.737	0.94498	1.60537
H	3.14907	1.08251	1.80046
H	0.51214	-2.23446	0.64949
H	5.03237	1.56223	-2.6228
H	-3.08703	-3.01008	-1.04834
H	-6.64286	-2.0347	-1.61873
H	2.26512	5.47425	-0.45254
H	-6.48197	1.17633	1.27215
H	-3.95971	-2.21517	2.45498
H	5.52316	-1.17195	-2.17204
H	-1.54995	4.11418	4.63204
H	-1.00882	3.1963	-1.34542
H	1.35474	0.31722	-3.10241
H	3.9232	2.19807	0.43583
H	0.30216	2.56175	0.24613
H	0.33564	-4.19013	-0.89001
H	-0.9117	-2.9707	-1.28342
H	-1.37101	-4.46663	-0.4123
H	0.63683	-0.40494	4.99335
H	-5.14584	0.95087	3.54436
H	-3.46002	1.41022	3.95253
H	-3.85782	-0.28646	3.50679
H	-3.9082	2.86585	0.47408
H	-3.42155	3.23847	2.1574
H	-5.13696	2.88534	1.78128
H	-7.79838	-0.25934	-0.29331
H	-3.58423	-3.72105	4.45184
H	-2.5342	-2.27426	4.51191
H	-1.85222	-3.85776	4.03617
H	-2.41229	5.14487	-1.96511

H	2.40624	1.99456	-5.48571
H	2.21167	0.20992	-5.46673
H	3.41273	0.98689	-4.3928
H	-4.38868	0.53963	-2.02547
H	5.05075	1.38146	3.42963
H	3.22638	-1.16778	1.35123
H	-0.56929	-4.77987	2.08295
H	0.42489	-3.51113	2.85494
H	1.11205	-4.50912	1.53984
H	0.66478	5.69596	-2.35705
H	-0.88974	1.40757	-3.66118
H	-0.27371	0.27981	-4.90957
H	-0.16363	2.04276	-5.16928
H	-4.21793	-1.62248	-3.55627
H	-2.84444	-2.78806	-3.52927
H	-2.70296	-1.20799	-2.70549
H	-1.39263	5.72243	2.71158
H	-5.26285	-4.35479	-1.06014
H	-4.19736	-4.68298	-2.46828
H	-5.64911	-3.66287	-2.66675
H	3.02666	-1.57009	-3.44037
H	0.20451	3.75295	-3.84477
H	-2.71826	-4.9501	1.70507
H	-3.82761	-3.98521	0.68704
H	-4.40116	-4.66524	2.23748
H	-5.77676	2.48178	-2.66322
H	-0.46978	4.94327	0.50364
H	-4.81756	4.80532	-2.62638
H	2.52588	-0.18071	6.60144
H	4.73984	0.72206	5.83675
H	4.02346	3.73203	2.37771
H	2.31483	3.51756	1.88525
H	3.18986	5.02569	1.47053
H	5.03552	4.95452	-0.40917
H	5.5123	3.37418	-1.10274
H	5.88851	3.74746	0.61119
H	2.56522	-5.26273	-2.71781
H	3.62459	-4.81938	1.45497
H	2.53207	-2.99404	2.92001
H	3.72676	-1.87871	3.64671
H	4.24513	-3.50221	3.10625
H	3.0325	-6.27182	-0.48427
H	6.05957	-2.41657	1.51922
H	5.55884	-0.86928	2.27988
H	5.58797	-1.0193	0.49522
H	4.29632	-3.45918	-4.53744
H	2.97628	-2.80401	-5.56425
H	2.76205	-4.36746	-4.72937
H	0.57901	-3.44763	-3.50784
H	0.79327	-1.94788	-4.47373
H	0.67499	-1.85737	-2.68216

Table S14. Cartesian geometry of complex **5** in Figure S104 in Angstrom [Å].

Atomtype	X Coordinates	Y Coordinates	Z Coordinates
C	4.79644	-1.31084	-0.02995

C	4.27529	-0.02769	-0.37903
C	5.12558	0.92376	-1.02702
C	6.47277	0.5951	-1.26313
C	6.99532	-0.65102	-0.88385
C	6.15341	-1.59478	-0.28197
N	2.91755	0.30714	-0.11905
Sn	1.46654	-0.38393	-1.55068
Ni	-0.63135	0.24553	-0.32765
C	-2.45453	0.61793	-0.56996
N	-3.26746	1.65543	-0.14927
C	-4.59015	1.49499	-0.58018
C	-4.62841	0.33426	-1.3047
N	-3.32725	-0.18373	-1.29988
C	-2.75575	2.88115	0.40703
C	-2.25049	3.85653	-0.49041
C	-1.80562	5.077	0.0526
C	-1.86875	5.3217	1.43177
C	-2.36387	4.33716	2.29688
C	-2.8089	3.09422	1.80337
C	-2.88441	-1.32215	-2.06324
C	-3.2834	-2.62487	-1.67659
C	-2.83069	-3.71291	-2.45245
C	-1.98882	-3.51673	-3.55086
C	-1.60603	-2.21735	-3.91835
C	-2.0649	-1.09282	-3.20804
C	-2.2371	3.63319	-1.99667
C	-0.84656	3.86133	-2.61089
C	-3.32225	2.02265	2.75014
C	-4.66248	2.42031	3.39443
C	-4.18613	-2.89173	-0.47882
C	-5.5952	-3.31799	-0.93761
C	-1.75868	0.32103	-3.69333
C	-0.55058	0.41449	-4.63201
C	4.54588	2.25468	-1.48925
C	5.5425	3.42289	-1.45941
C	3.89648	-2.395	0.54936
C	3.75198	-3.57224	-0.4323
P	-0.14084	-1.06505	1.29115
C	1.29988	-0.53523	2.33873
Si	2.36677	0.81318	1.48132
C	3.92003	1.06771	2.57866
C	4.65322	2.38392	2.26704
C	0.21381	-2.83935	0.86086
C	-0.06984	-3.28428	-0.4466
C	0.11701	-4.6315	-0.80056
C	0.57938	-5.55173	0.15222
C	0.86119	-5.11782	1.46182
C	0.67921	-3.77188	1.81264
C	-1.44772	-1.38301	2.59103
C	-1.14761	-1.78203	3.91187
C	-2.17842	-2.06072	4.82393
C	-3.52462	-1.94342	4.43046
C	-3.83093	-1.54044	3.12017
C	-2.79565	-1.25908	2.21432
C	-3.32128	4.4904	-2.67834

C	-2.26562	1.65583	3.80318
C	-3.57274	-3.92604	0.48278
C	-3.00244	0.9483	-4.35643
C	1.19538	2.32617	1.40781
C	0.98875	2.94188	2.80199
C	1.56727	3.39756	0.37655
C	3.6661	0.90417	4.08825
C	3.91284	2.11662	-2.88761
C	4.34871	-2.86726	1.94123
H	0.12476	1.24523	-1.30368
H	0.89501	-3.44911	2.84251
H	1.91688	-1.4046	2.64302
H	0.89208	-0.08197	3.26658
H	2.88989	-1.94713	0.66356
H	-3.01627	-0.92076	1.19228
H	0.2206	1.87262	1.07016
H	-5.45085	-0.16276	-1.82743
H	3.71956	2.48727	-0.78865
H	7.13015	1.32995	-1.75339
H	-3.12734	-4.7337	-2.16754
H	6.55545	-2.58387	-0.00647
H	4.59031	0.23661	2.25816
H	-5.37016	2.22382	-0.34105
H	1.22895	-5.8338	2.21367
H	-1.50491	0.90649	-2.78426
H	-4.29469	-1.93877	0.07541
H	-0.46535	-2.56999	-1.18578
H	0.79273	4.193	0.3406
H	1.64655	2.96401	-0.63965
H	2.53745	3.8816	0.61757
H	-0.1008	-1.86699	4.24152
H	5.34933	-3.34866	1.90598
H	3.63432	-3.61195	2.35155
H	4.41373	-2.01961	2.65446
H	3.36758	-3.221	-1.41246
H	3.04345	-4.32819	-0.03821
H	4.72754	-4.0718	-0.61349
H	8.05495	-0.88823	-1.06882
H	4.61532	0.99953	4.66159
H	3.23166	-0.08631	4.33668
H	2.97285	1.67748	4.48077
H	-3.31082	0.36288	-5.24854
H	-2.78013	1.9834	-4.68902
H	-3.86571	0.99879	-3.66418
H	-4.88033	-1.43253	2.80237
H	-3.50128	1.11493	2.14356
H	1.9061	3.46253	3.15041
H	0.72711	2.18772	3.57264
H	0.1681	3.68909	2.78585
H	-1.62884	-4.37975	-4.1329
H	0.35335	-0.0557	-4.1891
H	-0.31077	1.4791	-4.82787
H	-0.73998	-0.06694	-5.61505
H	4.6782	1.85088	-3.64725
H	3.41898	3.0609	-3.20158

H	3.14669	1.30913	-2.90755
H	0.72688	-6.60791	-0.12258
H	6.03239	3.51741	-0.46834
H	5.02172	4.37898	-1.67624
H	6.34342	3.30935	-2.22086
H	-2.48983	2.5713	-2.17423
H	-0.95133	-2.07348	-4.78897
H	4.04535	3.27013	2.54828
H	4.89749	2.47141	1.19096
H	5.61105	2.45215	2.82911
H	-0.10963	-4.95468	-1.82731
H	-1.92955	-2.36722	5.85239
H	-4.33119	-2.15914	5.14868
H	-4.18952	-4.01706	1.39989
H	-2.54977	-3.63582	0.79047
H	-3.51244	-4.93113	0.01536
H	-5.55979	-4.2722	-1.50456
H	-6.05878	-2.55752	-1.59943
H	-6.26384	-3.46791	-0.06417
H	-1.40883	5.85246	-0.621
H	-2.4008	4.52989	3.38043
H	-1.32649	1.34226	3.30945
H	-2.61777	0.81323	4.43117
H	-2.03279	2.51123	4.4716
H	-1.51972	6.28484	1.83587
H	-4.55549	3.32398	4.03156
H	-5.04165	1.59704	4.03557
H	-5.43221	2.64118	2.62615
H	-4.3264	4.27283	-2.2619
H	-3.35236	4.28862	-3.7695
H	-3.12572	5.57478	-2.53874
H	-0.49222	4.90456	-2.47431
H	-0.87103	3.65917	-3.70199
H	-0.1091	3.175	-2.14894

Table S15. Cartesian geometry of INT1 (14.4 kcal/mol) in Figure S104 in Angstrom [Å].

Atomtype	X Coordinates	Y Coordinates	Z Coordinates
C	-2.81596	-1.81792	3.06426
C	-3.18339	-1.06106	1.92416
C	-3.87942	0.17475	2.03685
C	-4.19464	0.62968	3.33016
C	-3.87809	-0.12405	4.46787
C	-3.19471	-1.33517	4.33302
N	-2.98762	-1.65523	0.62379
C	-2.21295	-1.18411	-0.43137
N	-2.51579	-2.09884	-1.44579
C	-3.4274	-3.06981	-1.03306
C	-3.72293	-2.79466	0.27118
Ni	-1.00268	0.27073	-0.65346
P	-0.59807	2.13773	0.369
C	-1.58715	3.56045	-0.31664
C	-1.3485	4.91506	0.00496
C	-2.10695	5.93866	-0.58736
C	-3.10843	5.62577	-1.523
C	-3.35001	4.28256	-1.8564

C	-2.59771	3.26349	-1.25282
C	-2.08366	-1.96551	-2.81317
C	-0.96897	-2.70776	-3.27476
C	-0.59448	-2.55373	-4.62486
C	-1.2975	-1.69375	-5.47827
C	-2.3942	-0.96496	-4.99702
C	-2.80898	-1.08297	-3.65695
C	-0.20383	-3.64383	-2.35059
C	1.2978	-3.71093	-2.66504
C	-4.01209	-0.30549	-3.13548
C	-5.30809	-1.12153	-3.31841
C	-4.32648	0.95446	0.80654
C	-5.51325	0.25669	0.11029
C	-2.00635	-3.10721	2.97684
C	-0.6714	-2.95419	3.72696
C	1.11156	2.91532	0.48022
Si	2.43463	2.24746	-0.72923
C	1.65125	2.21153	-2.47586
C	0.93862	3.51379	-2.88259
N	2.79793	0.59315	-0.20736
Sn	1.18315	-0.78339	-0.51022
C	1.93183	-2.58737	0.54099
C	0.9281	-3.52532	0.87254
C	1.2442	-4.80952	1.34855
C	2.59145	-5.17956	1.50778
C	3.60637	-4.25897	1.18865
C	3.28121	-2.9785	0.70644
C	3.97817	0.24941	0.49586
C	3.98249	0.10414	1.91944
C	5.18153	-0.24835	2.56931
C	6.3666	-0.47125	1.85756
C	6.35838	-0.34983	0.46012
C	5.18677	0.00253	-0.23365
C	2.72753	0.31379	2.75773
C	2.43992	-0.88649	3.67534
C	5.17178	0.04801	-1.75561
C	6.4966	0.49745	-2.38673
C	4.70646	-1.30345	-2.33066
C	2.79389	1.61606	3.5784
C	4.00039	3.34816	-0.61277
C	3.82637	4.67347	-1.37697
C	4.46161	3.60245	0.83296
C	-1.01612	2.1311	2.17884
C	-0.72568	0.94297	2.88131
C	-0.85262	0.88979	4.27595
C	-1.31435	2.01084	4.98665
C	-1.65633	3.18105	4.28953
C	-1.50076	3.2453	2.89389
C	-0.80952	-5.06174	-2.33532
C	-4.15125	1.08849	-3.76662
C	-4.68387	2.41522	1.10477
C	-2.79911	-4.32582	3.48509
C	2.54147	1.66739	-3.60168
H	-1.04525	1.15423	-2.18076
H	-1.34532	0.40393	-2.39183

H	0.27115	-3.11217	-5.00976
H	-4.39984	-3.28225	0.97791
H	-1.75763	4.17423	2.36371
H	1.48827	2.68371	1.49607
H	1.02805	4.01953	0.42277
H	1.8795	0.40959	2.04759
H	-2.77617	2.20779	-1.50556
H	0.85197	1.45559	-2.30283
H	4.40003	0.78879	-2.0389
H	7.28448	-0.53845	-0.10461
H	-4.71092	1.59116	3.45053
H	5.18294	-0.35401	3.66613
H	4.79944	2.76282	-1.12176
H	-3.79055	-3.85234	-1.70478
H	-2.03934	4.05805	4.83548
H	-0.13444	-3.2488	0.7529
H	-1.76717	-3.28497	1.90898
H	-3.45466	0.94179	0.1107
H	-0.40473	0.05782	2.30897
H	1.99087	1.65575	-4.56864
H	2.86269	0.62631	-3.3984
H	3.45661	2.2803	-3.75361
H	-0.55191	5.18505	0.71539
H	3.60594	1.56629	4.33501
H	1.83601	1.79255	4.11028
H	2.99925	2.49443	2.9365
H	2.3538	-1.82575	3.09561
H	1.49634	-0.72583	4.23437
H	3.24303	-1.02364	4.42975
H	7.29239	-0.74467	2.38773
H	5.38862	4.21773	0.8488
H	4.67825	2.66024	1.37312
H	3.69357	4.16041	1.41174
H	0.43922	-5.52148	1.59524
H	-3.05665	-4.22097	4.56011
H	-2.20182	-5.25463	3.37273
H	-3.74915	-4.46109	2.92835
H	4.09721	-2.28199	0.46514
H	-4.12631	4.02466	-2.59363
H	-3.85167	-0.1599	-2.04763
H	0.30923	3.92966	-2.07106
H	0.26601	3.33563	-3.75118
H	1.65497	4.30522	-3.18448
H	-4.15204	0.24905	5.46671
H	-0.07377	-2.12165	3.31024
H	-0.05662	-3.87154	3.6372
H	-0.83342	-2.75686	4.80754
H	5.40634	-2.11876	-2.05029
H	4.64027	-1.26849	-3.43899
H	3.70495	-1.57828	-1.94053
H	-1.42457	1.96517	6.08137
H	6.84822	1.45594	-1.95127
H	6.37247	0.6416	-3.48038
H	7.30468	-0.25205	-2.24794
H	-0.31452	-3.23009	-1.3275

H	-2.92611	-1.91404	5.23048
H	2.96722	5.26454	-0.99033
H	3.6565	4.5145	-2.46067
H	4.7311	5.31278	-1.27245
H	4.66461	-4.5398	1.31481
H	-0.61177	-0.04184	4.80818
H	2.85044	-6.18414	1.87898
H	-1.90591	6.98915	-0.32377
H	-3.69409	6.42962	-1.99606
H	-4.89526	2.95508	0.16166
H	-3.8553	2.94486	1.61351
H	-5.59228	2.49413	1.73965
H	-6.39468	0.22208	0.78542
H	-5.27901	-0.78179	-0.19357
H	-5.80723	0.81797	-0.80093
H	-2.93508	-0.28653	-5.67324
H	-3.19989	1.65502	-3.71805
H	-4.92837	1.6709	-3.22971
H	-4.4631	1.03615	-4.83097
H	-0.98431	-1.58537	-6.52857
H	-5.49542	-1.32347	-4.39428
H	-6.18137	-0.56881	-2.91417
H	-5.25365	-2.0955	-2.79327
H	-1.87375	-5.05529	-2.02839
H	-0.25461	-5.70365	-1.61976
H	-0.74752	-5.52939	-3.34079
H	1.50025	-4.25009	-3.61437
H	1.83372	-4.24166	-1.85343
H	1.73739	-2.69478	-2.75056

Table S16. Cartesian geometry of INT2 (16.6 kcal/mol) in Figure S104 in Angstrom [Å].

Atomtype	X Coordinates	Y Coordinates	Z Coordinates
C	-3.89326	1.49167	-1.39847
C	-3.76707	0.19944	-0.78633
C	-4.68468	-0.83438	-1.18565
C	-5.67391	-0.56205	-2.14929
C	-5.80287	0.707	-2.72719
C	-4.91516	1.71782	-2.3405
N	-2.7367	-0.10166	0.1395
Si	-2.61469	0.52628	1.75882
C	-1.38081	2.00273	1.99115
P	0.265	1.99225	1.15387
Ni	1.1147	0.19523	0.31723
Sn	-1.13614	-1.42514	-0.67115
C	-0.94808	-0.11899	-2.5069
C	0.19651	0.67684	-2.70105
C	0.39243	1.41344	-3.88244
C	-0.56202	1.34318	-4.91172
C	-1.71883	0.55954	-4.73608
C	-1.91204	-0.15861	-3.54183
C	-4.60801	-2.2449	-0.60566
C	-5.95245	-2.72704	-0.02868
C	-2.94286	2.64254	-1.08357
C	-2.3393	3.27749	-2.34662
C	0.28654	3.51222	0.08792

C	1.03897	3.51212	-1.10159
C	1.1626	4.68281	-1.86709
C	0.53846	5.86878	-1.4483
C	-0.20438	5.87996	-0.25377
C	-0.32718	4.70963	0.51095
C	1.43539	2.61694	2.4606
C	1.00534	3.38074	3.56479
C	1.93733	3.88989	4.48494
C	3.31083	3.64934	4.30884
C	3.74658	2.89637	3.2043
C	2.81505	2.38223	2.29016
C	2.3047	-1.21287	-0.00043
N	3.361	-1.28347	-0.886
C	4.25399	-2.29964	-0.54961
C	3.74106	-2.91775	0.55662
N	2.53925	-2.27342	0.85604
C	3.49909	-0.51144	-2.09428
C	2.87821	-1.00106	-3.27379
C	3.12123	-0.28994	-4.46451
C	3.92061	0.86185	-4.47197
C	4.48438	1.34285	-3.28262
C	4.28988	0.66084	-2.06472
C	1.66173	-2.78672	1.88012
C	0.97161	-3.99537	1.60446
C	0.23532	-4.57575	2.65673
C	0.18036	-3.97073	3.91728
C	0.8316	-2.74981	4.14441
C	1.58493	-2.12621	3.13207
C	1.96043	-2.22413	-3.25476
C	0.96088	-2.25244	-4.42115
C	4.95047	1.14388	-0.77837
C	6.42015	0.68012	-0.71181
C	1.02242	-4.67785	0.23786
C	-0.35885	-5.17958	-0.22078
C	2.27557	-0.78607	3.356
C	3.80687	-0.92156	3.46084
C	2.74449	-3.55438	-3.2221
C	4.8391	2.66649	-0.59702
C	2.03186	-5.84531	0.22381
C	1.72364	-0.014	4.55879
C	-1.9753	-0.82401	2.96554
C	-2.11662	-0.39801	4.43782
C	-4.33396	1.20605	2.28123
C	-5.32268	0.07835	2.62118
C	-2.59551	-2.21163	2.73068
C	-4.09176	-3.25858	-1.64731
C	-3.63136	3.73818	-0.24577
C	-4.31434	2.28719	3.37564
H	0.06985	-0.65404	1.03103
H	-1.19319	2.0453	3.08393
H	2.18243	1.10358	-0.19743
H	-0.89646	4.73889	1.45284
H	-1.90936	2.93884	1.72659
H	-2.10496	2.2072	-0.50226
H	3.13453	1.77378	1.4317

H	-0.88616	-0.90533	2.74241
H	5.15943	-2.48818	-1.1326
H	-3.87723	-2.20238	0.22744
H	-6.36655	-1.36611	-2.44784
H	5.09359	2.2592	-3.2984
H	-5.00073	2.71492	-2.80158
H	-4.70632	1.67534	1.34278
H	4.11524	-3.74553	1.16394
H	-0.69168	6.80717	0.08629
H	0.96382	0.71251	-1.9001
H	1.36849	-2.15592	-2.31321
H	4.40586	0.66595	0.06297
H	1.53373	2.58388	-1.41769
H	-2.22468	-2.94153	3.48092
H	-2.32906	-2.61268	1.73154
H	-3.70194	-2.19267	2.79912
H	-0.06526	3.58517	3.71745
H	-4.48604	4.17283	-0.80662
H	-2.9247	4.56449	-0.02626
H	-4.03181	3.35757	0.71283
H	-1.83259	2.52623	-2.97483
H	-1.59756	4.05176	-2.06848
H	-3.11783	3.77332	-2.9645
H	-6.58625	0.90557	-3.47557
H	-5.34053	2.66763	3.57777
H	-3.69362	3.16362	3.09562
H	-3.91882	1.89758	4.3373
H	1.29681	2.02972	-4.00392
H	3.3768	-3.65552	-4.1295
H	2.0372	-4.40965	-3.20419
H	3.39821	-3.64692	-2.33483
H	-2.83663	-0.74892	-3.42338
H	4.82056	2.70031	3.05721
H	2.04434	-0.18486	2.44083
H	-3.18045	-0.35298	4.75253
H	-1.67268	0.59936	4.64415
H	-1.61021	-1.1248	5.11059
H	4.08892	1.40283	-5.41645
H	0.4256	-1.29254	-4.53769
H	0.2018	-3.04022	-4.2402
H	1.46462	-2.49662	-5.38094
H	-4.79172	-3.34042	-2.5064
H	-3.97543	-4.26898	-1.20037
H	-3.1036	-2.96118	-2.05916
H	0.62892	6.78634	-2.05045
H	-6.36469	-2.00531	0.70439
H	-5.82964	-3.70481	0.48355
H	-6.71564	-2.86412	-0.82402
H	1.36014	-3.91774	-0.49855
H	2.65091	-0.62749	-5.39815
H	-5.06014	-0.43303	3.57137
H	-5.35047	-0.68942	1.82376
H	-6.35595	0.47408	2.73827
H	-2.4824	0.51839	-5.52984
H	1.74509	4.66027	-2.8012

H	-0.41337	1.90914	-5.84539
H	1.58559	4.47976	5.34598
H	4.03958	4.04713	5.03226
H	5.24708	2.9705	0.38839
H	3.78404	3.00149	-0.64465
H	5.41229	3.21861	-1.37091
H	7.01131	1.11371	-1.546
H	6.50406	-0.42335	-0.7795
H	6.88984	0.99854	0.24242
H	-0.31165	-5.51339	2.47816
H	0.75016	-2.26986	5.12915
H	0.62871	0.13281	4.48218
H	2.19733	0.98378	4.61857
H	1.93573	-0.53613	5.51612
H	-0.39377	-4.44362	4.72927
H	4.08575	-1.54659	4.33581
H	4.26498	0.07939	3.59596
H	4.255	-1.37818	2.55716
H	3.05706	-5.52067	0.48677
H	2.07335	-6.31013	-0.78323
H	1.7313	-6.63041	0.94944
H	-0.71833	-6.02698	0.39975
H	-0.30199	-5.54076	-1.26857
H	-1.11076	-4.36608	-0.18269

Table S17. Cartesian geometry of INT4 (-4.3 kcal/mol) in Figure S104 in Angstrom [Å].

Atomtype	X Coordinates	Y Coordinates	Z Coordinates
C	-4.55201	1.32326	0.83752
C	-3.79363	0.12499	0.61589
C	-4.29416	-1.11026	1.13954
C	-5.55784	-1.14348	1.76041
C	-6.32606	0.01845	1.91273
C	-5.80638	1.2439	1.46999
N	-2.56247	0.19234	-0.08519
Sn	-0.82832	0.62744	1.26053
Ni	1.03739	-0.25423	-0.30271
C	2.10845	1.12326	0.33687
N	2.60335	2.18932	-0.38878
C	3.62503	2.85246	0.2945
C	3.76444	2.22094	1.49885
N	2.82764	1.18619	1.51789
C	2.14094	2.63817	-1.67506
C	1.59266	3.94259	-1.75369
C	1.3169	4.45932	-3.03652
C	1.54077	3.69295	-4.18439
C	2.01435	2.37656	-4.07079
C	2.32849	1.81432	-2.81895
C	2.60726	0.34961	2.66991
C	3.14977	-0.96112	2.68728
C	2.94804	-1.72787	3.85089
C	2.23506	-1.21335	4.94384
C	1.70306	0.08036	4.89523
C	1.87692	0.89436	3.7579
C	1.27897	4.78485	-0.51822
C	-0.21111	5.17257	-0.46481

C	2.82423	0.37615	-2.70005
C	4.28069	0.26857	-2.21543
C	3.90985	-1.5153	1.48446
C	5.30237	-0.87045	1.32804
C	1.32051	2.31738	3.73393
C	-0.06468	2.43443	4.39487
C	-3.44857	-2.37595	1.09609
C	-3.08181	-2.84475	2.51553
C	-3.97308	2.68111	0.45713
C	-4.96861	3.60947	-0.25808
P	0.12465	-2.10328	-0.9664
C	-1.49672	-2.11286	-1.83344
Si	-2.37981	-0.40483	-1.70736
C	-4.11032	-0.65636	-2.49072
C	-4.77285	0.67721	-2.87307
C	1.27628	-3.02712	-2.09132
C	2.62038	-3.12772	-1.67229
C	3.57326	-3.79114	-2.45714
C	3.19735	-4.35769	-3.68926
C	1.86274	-4.26175	-4.11647
C	0.90666	-3.60404	-3.32128
C	-0.02626	-3.33319	0.40208
C	0.40146	-2.9518	1.68805
C	0.37594	-3.87688	2.74463
C	-0.07428	-5.18792	2.51875
C	-0.50421	-5.57287	1.23383
C	-0.47954	-4.64954	0.1771
C	2.18174	6.03187	-0.44055
C	2.61528	-0.44885	-3.97217
C	4.0437	-3.04429	1.50754
C	2.30037	3.30406	4.40527
C	-1.19949	0.73956	-2.68482
C	-1.11118	0.38556	-4.17707
C	-1.49849	2.22622	-2.46087
C	-4.14522	-1.66304	-3.65396
C	-4.10439	-3.50587	0.28477
C	-3.39523	3.37658	1.70733
H	-0.57717	2.35682	0.6379
H	-0.79896	-4.95755	-0.83171
H	-2.12277	-2.94969	-1.46802
H	-1.31869	-2.28029	-2.91478
H	-2.50025	-2.09902	0.59589
H	2.9143	-2.67316	-0.71709
H	-0.19719	0.53122	-2.2152
H	4.44817	2.39858	2.33266
H	-3.13017	2.47269	-0.23205
H	-6.39097	2.16602	1.62152
H	3.34703	-2.75002	3.90098
H	-5.94239	-2.10296	2.14523
H	-4.70151	-1.08716	-1.64914
H	4.15833	3.6987	-0.14611
H	-0.85807	-6.60059	1.05573
H	1.20675	2.61188	2.66773
H	3.29728	-1.2333	0.59096
H	0.75567	-1.91925	1.83816

H	-0.79444	2.86548	-3.03128
H	-1.39208	2.48016	-1.38738
H	-2.52839	2.4957	-2.77444
H	-0.13479	-3.54612	-3.67201
H	-5.04843	-3.85128	0.75803
H	-3.42643	-4.38316	0.21462
H	-4.35518	-3.17591	-0.74448
H	-2.52581	-2.05172	3.05667
H	-2.43938	-3.74737	2.4765
H	-3.9876	-3.09247	3.10935
H	-7.31608	-0.02611	2.39388
H	-5.18257	-1.79473	-4.03538
H	-3.77848	-2.66666	-3.35399
H	-3.52609	-1.32746	-4.51336
H	2.44711	3.03913	5.47372
H	1.90015	4.33836	4.36155
H	3.29601	3.30693	3.92134
H	4.61697	-3.85918	-2.11123
H	2.16812	-0.09514	-1.90149
H	-2.07831	0.56355	-4.69432
H	-0.83585	-0.67662	-4.35131
H	-0.35044	1.01315	-4.69038
H	2.08747	-1.83381	5.84181
H	-0.76547	1.67027	3.99931
H	-0.49908	3.43374	4.18969
H	-0.00835	2.31852	5.49776
H	-4.1989	3.60849	2.43935
H	-2.88368	4.32475	1.43705
H	-2.65378	2.7308	2.22199
H	-0.09856	-5.91447	3.34618
H	-5.4155	3.12401	-1.14933
H	-4.45919	4.53699	-0.59479
H	-5.80308	3.91794	0.40774
H	1.47153	4.15906	0.377
H	1.1322	0.46695	5.75214
H	-4.24953	1.1665	-3.72233
H	-4.77115	1.38729	-2.02334
H	-5.83162	0.52871	-3.18149
H	0.70371	-3.56086	3.7464
H	1.55823	-4.70331	-5.07847
H	3.94371	-4.87151	-4.31486
H	4.54243	-3.39864	0.5832
H	3.05906	-3.54743	1.57994
H	4.67305	-3.38435	2.35665
H	5.93991	-1.10592	2.20644
H	5.25147	0.23005	1.22693
H	5.80856	-1.26457	0.42267
H	0.89959	5.47386	-3.12915
H	2.15338	1.77682	-4.98023
H	1.55589	-0.44464	-4.29249
H	2.91273	-1.49887	-3.79545
H	3.23576	-0.06806	-4.81141
H	1.31945	4.11034	-5.17894
H	4.97191	0.71938	-2.9588
H	4.55755	-0.79867	-2.09362

H	4.44748	0.77309	-1.24528
H	3.25966	5.76869	-0.44955
H	1.97827	6.6047	0.48806
H	2.00199	6.71155	-1.3002
H	-0.5149	5.77993	-1.3429
H	-0.41593	5.77169	0.44678
H	-0.84343	4.26413	-0.42248

Table S18. Cartesian geometry of product (-4.3 kcal/mol) in Figure S104 in Angstrom [Å].

Atomtype	X Coordinates	Y Coordinates	Z Coordinates
C	-2.59799	-2.15562	2.19104
C	-1.23226	-2.12337	2.53293
C	-0.83316	-2.6311	3.78673
C	-1.78437	-3.16504	4.67428
C	-3.14269	-3.20765	4.31528
C	-3.54748	-2.70283	3.06593
P	-0.0675	-1.44335	1.24563
C	0.26581	-3.03206	0.34084
C	0.00118	-3.09262	-1.0418
C	0.1719	-4.29389	-1.74964
C	0.59564	-5.45112	-1.07738
C	0.85574	-5.40137	0.3054
C	0.69148	-4.19889	1.01021
Ni	-0.80497	0.10101	-0.02129
C	-2.50325	0.73524	-0.45371
N	-3.28407	1.74676	0.07544
C	-4.53564	1.82365	-0.54622
C	-4.55459	0.84937	-1.50846
N	-3.31444	0.20693	-1.44959
C	-2.78568	2.74293	0.98556
C	-2.39607	3.99323	0.44691
C	-1.98035	4.99252	1.35066
C	-1.94659	4.7457	2.72895
C	-2.29423	3.48123	3.23012
C	-2.71136	2.44839	2.3696
C	-2.82858	-0.75268	-2.40571
C	-3.17686	-2.1158	-2.25895
C	-2.70358	-3.0243	-3.22764
C	-1.89399	-2.59228	-4.28408
C	-1.55146	-1.23625	-4.40323
C	-2.02331	-0.28301	-3.48141
C	-2.32944	4.22827	-1.05874
C	-2.94052	5.57271	-1.48646
C	-3.05854	1.05365	2.87141
C	-2.44795	0.71757	4.2364
C	-4.05744	-2.59485	-1.11316
C	-3.54216	-3.89836	-0.47986
C	-1.71565	1.20253	-3.64867
C	-2.9048	1.95676	-4.28038
Sn	1.17063	0.27063	-1.3908
N	2.85398	0.35103	-0.0799
Si	2.43077	0.41679	1.63181
C	1.16957	1.82405	1.96041
C	1.4135	3.13115	1.19691
C	4.17145	0.09944	-0.54232

C	4.97086	1.18765	-1.01698
C	6.29099	0.94058	-1.43527
C	6.83448	-0.35284	-1.40254
C	6.03719	-1.42205	-0.97415
C	4.70721	-1.2234	-0.5545
C	4.36382	2.58214	-1.11177
C	3.63633	2.76417	-2.45872
C	3.84951	-2.42238	-0.17322
C	4.36447	-3.13943	1.08692
C	-0.87261	4.07971	-1.54155
C	-4.58129	0.81803	2.88542
C	-5.52204	-2.74149	-1.57487
C	-0.43737	1.47771	-4.45258
C	3.69968	-3.39788	-1.35384
C	5.36438	3.72476	-0.88201
C	1.4774	-1.13744	2.23239
C	4.05658	0.52736	2.63484
C	3.94963	0.00243	4.0776
C	4.67968	1.9331	2.58423
C	1.02014	2.08365	3.46898
H	0.87813	-4.17317	2.09544
H	2.11939	-2.03957	2.26546
H	1.16548	-0.91738	3.27471
H	2.83742	-2.03253	0.05181
H	-2.89722	-1.717	1.22641
H	0.19629	1.38838	1.58307
H	-5.33074	0.55619	-2.22118
H	3.58974	2.63541	-0.3195
H	6.9107	1.77795	-1.79218
H	-2.95865	-4.09046	-3.13608
H	6.45284	-2.44335	-0.9734
H	4.73853	-0.15469	2.07527
H	-5.28542	2.56418	-0.25194
H	1.18816	-6.30675	0.83745
H	-1.55498	1.6002	-2.62476
H	-4.03165	-1.80058	-0.33765
H	-0.37478	-2.19024	-1.55244
H	0.57928	3.84548	1.36331
H	1.48809	2.95658	0.1052
H	2.35331	3.62554	1.5224
H	0.22748	-2.61251	4.08115
H	5.36913	-3.58333	0.91959
H	3.67725	-3.96272	1.37627
H	4.45178	-2.44223	1.94569
H	3.26571	-2.88132	-2.23392
H	3.02868	-4.23713	-1.0827
H	4.67938	-3.82538	-1.65676
H	7.87396	-0.52664	-1.72279
H	4.93926	0.03497	4.58542
H	3.59542	-1.04846	4.11662
H	3.25143	0.61022	4.6911
H	-3.1159	1.57569	-5.30199
H	-2.67349	3.03972	-4.36074
H	-3.82869	1.85739	-3.67884
H	-4.61044	-2.72695	2.77747

H	-2.59949	0.3612	2.13103
H	1.9415	2.53859	3.89101
H	0.81821	1.15686	4.04578
H	0.18463	2.78621	3.66834
H	-1.52178	-3.31728	-5.02509
H	0.42897	0.898	-4.07194
H	-0.17538	2.55335	-4.3916
H	-0.56029	1.23013	-5.52835
H	4.34965	2.70093	-3.3076
H	3.11479	3.74369	-2.50858
H	2.87694	1.96815	-2.62331
H	0.72835	-6.39483	-1.62941
H	5.92357	3.59284	0.06704
H	4.83413	4.69891	-0.83493
H	6.10906	3.79944	-1.70283
H	-2.91679	3.42906	-1.55453
H	-0.91196	-0.9125	-5.2365
H	4.05753	2.67792	3.12507
H	4.8006	2.29005	1.54344
H	5.68679	1.94295	3.05666
H	-0.03611	-4.31836	-2.82935
H	-1.46036	-3.55223	5.65344
H	-3.88595	-3.62707	5.01131
H	-4.13159	-4.14891	0.425
H	-2.47923	-3.81299	-0.18159
H	-3.63127	-4.75593	-1.17917
H	-5.6087	-3.50723	-2.37461
H	-5.92209	-1.78959	-1.97893
H	-6.17147	-3.05409	-0.73046
H	-1.66834	5.97511	0.96463
H	-2.23771	3.29166	4.31153
H	-1.35208	0.87418	4.23851
H	-2.63808	-0.34599	4.47993
H	-2.88731	1.32883	5.05376
H	-1.62419	5.53925	3.4212
H	-5.08083	1.52663	3.58006
H	-4.80352	-0.21243	3.22991
H	-5.03365	0.94511	1.88214
H	-3.98644	5.67556	-1.13067
H	-2.94342	5.65952	-2.59285
H	-2.36289	6.43544	-1.0929
H	-0.21644	4.84584	-1.07825
H	-0.81094	4.19604	-2.64386
H	-0.47038	3.08076	-1.27428
H	2.04785	-0.60427	-2.6812

Table S19. Cartesian geometry of H_2 in Figure S104 in Angstrom [Å].

Atomtype	X Coordinates	Y Coordinates	Z Coordinates
H	0	0	0
H	0	0	0.767382

Table S20. Cartesian geometry of TS1 (20.6 kcal/mol) in Figure S104 in Angstrom [Å].

Atomtype	X Coordinates	Y Coordinates	Z Coordinates
C	-0.62418	1.48577	2.87191
C	-1.13793	2.41695	1.94473

C	-1.79675	3.56312	2.43563
C	-1.89859	3.78899	3.81989
C	-1.33159	2.88394	4.73187
C	-0.69743	1.72553	4.25006
P	-0.76408	2.09049	0.14677
C	0.91372	2.93548	0.09065
Si	2.19034	2.17842	-1.11754
C	1.24948	1.88555	-2.75471
C	1.93858	1.00155	-3.80175
N	2.71666	0.64528	-0.37024
Sn	1.18891	-0.73661	0.15086
C	2.27831	-2.55908	0.71406
C	3.67672	-2.75461	0.73343
C	4.21569	-4.00169	1.09772
C	3.37036	-5.06833	1.452
C	1.97621	-4.88293	1.44848
C	1.44157	-3.635	1.08625
C	3.99926	0.51256	0.21953
C	4.17755	0.57065	1.64046
C	5.47196	0.41701	2.17553
C	6.58724	0.21179	1.3545
C	6.41208	0.14832	-0.03597
C	5.13994	0.28903	-0.61872
C	3.01748	0.78773	2.60887
C	3.0635	2.17223	3.28177
C	4.95442	0.12003	-2.1216
C	4.58602	-1.3375	-2.45654
C	3.6961	3.36696	-1.23837
C	4.16374	3.88891	0.13138
C	3.44322	4.534	-2.21
C	6.14432	0.60543	-2.96049
C	2.92402	-0.32949	3.66253
Ni	-0.96853	0.00786	-0.48173
C	-2.23573	-1.37506	-0.1799
N	-2.93285	-1.76228	0.94723
C	-3.63527	-2.95779	0.75061
C	-3.4	-3.33699	-0.54247
N	-2.56149	-2.36737	-1.09697
C	-3.03768	-1.00547	2.16646
C	-3.83131	0.17374	2.18153
C	-4.03341	0.80001	3.42473
C	-3.49664	0.26556	4.60488
C	-2.69517	-0.87981	4.55745
C	-2.4303	-1.52448	3.33281
C	-2.22013	-2.31616	-2.49625
C	-1.03994	-2.94736	-2.95481
C	-0.75734	-2.88298	-4.33357
C	-1.61084	-2.20841	-5.21634
C	-2.76159	-1.57126	-4.73349
C	-3.08675	-1.60525	-3.36373
C	-4.4675	0.71476	0.90472
C	-4.86652	2.19288	0.99407
C	-1.4847	-2.71845	3.28337
C	-2.05109	-3.94163	4.02674
C	-0.10377	-3.6636	-1.99148

C	-0.52675	-5.12805	-1.76312
C	-4.3327	-0.90451	-2.8361
C	-4.65381	0.39127	-3.59699
C	-1.82345	3.31403	-0.75743
C	-1.67465	4.71319	-0.63206
C	-2.50289	5.58833	-1.3535
C	-3.4864	5.07565	-2.2175
C	-3.63227	3.68543	-2.35842
C	-2.80392	2.81297	-1.63439
C	0.62667	3.14945	-3.37603
C	-5.68502	-0.13467	0.4836
C	-0.09475	-2.3262	3.81217
C	1.36709	-3.58464	-2.42336
C	-5.55059	-1.85128	-2.84637
H	-0.05468	-0.45696	-1.6299
H	-1.38834	0.25788	-1.86989
H	0.15509	-3.3591	-4.72019
H	-4.24261	-3.4097	1.53998
H	-2.22874	4.29498	1.73747
H	1.34473	2.83762	1.10746
H	0.77891	4.02213	-0.087
H	2.07672	0.76536	2.01383
H	-2.8796	1.71884	-1.74792
H	0.39818	1.28966	-2.35882
H	4.07657	0.73115	-2.40572
H	7.28559	-0.02529	-0.68309
H	-4.62603	1.72369	3.47335
H	5.60751	0.46256	3.26822
H	4.52331	2.75273	-1.65758
H	-3.75722	-4.19515	-1.11854
H	-2.41764	4.68949	4.18552
H	0.34551	-3.50328	1.08526
H	-1.36245	-2.99772	2.21627
H	-3.68556	0.62332	0.11714
H	-0.17683	0.55464	2.48931
H	1.2515	0.78887	-4.65068
H	2.23663	0.0239	-3.37153
H	2.84972	1.47701	-4.22656
H	-0.89974	5.13209	0.02883
H	3.96892	2.27584	3.9171
H	2.17034	2.32293	3.9231
H	3.088	2.9871	2.53314
H	2.85625	-1.32932	3.18939
H	2.03136	-0.1833	4.30495
H	3.81203	-0.33173	4.3288
H	7.58992	0.09783	1.79536
H	5.04394	4.55977	0.01637
H	4.46215	3.06604	0.80915
H	3.36971	4.48069	0.63655
H	1.30458	-5.71019	1.73006
H	-2.19418	-3.7263	5.10682
H	-1.35865	-4.80537	3.94467
H	-3.03399	-4.24977	3.615
H	4.3598	-1.93613	0.46762
H	-4.39061	3.2771	-3.04383

H	-4.12252	-0.62749	-1.78268
H	0.19039	3.83058	-2.61722
H	-0.20139	2.87087	-4.0645
H	1.36046	3.73463	-3.96718
H	-3.68429	0.76784	5.56622
H	0.31983	-1.48109	3.22828
H	0.61984	-3.16923	3.72581
H	-0.13302	-2.01803	4.878
H	5.40249	-2.03206	-2.16586
H	4.39682	-1.46132	-3.54384
H	3.67226	-1.64857	-1.91377
H	-1.4005	3.07063	5.81502
H	6.43058	1.64538	-2.69817
H	5.88712	0.58195	-4.03993
H	7.042	-0.03516	-2.82724
H	-0.20086	-3.13452	-1.01994
H	-2.24562	-1.27219	5.48318
H	2.55398	5.13432	-1.91846
H	3.28078	4.18699	-3.24943
H	4.31235	5.22876	-2.22234
H	5.30912	-4.13769	1.10608
H	-0.27934	0.98724	4.95135
H	3.79818	-6.0431	1.73573
H	-2.37416	6.677	-1.24563
H	-4.13191	5.76149	-2.78857
H	-5.18307	2.55969	-0.00246
H	-4.02175	2.82427	1.32808
H	-5.7168	2.3505	1.69185
H	-6.47314	-0.10041	1.26563
H	-5.42286	-1.19652	0.31067
H	-6.12295	0.26178	-0.45606
H	-3.41472	-1.0278	-5.43211
H	-3.75524	1.03039	-3.70334
H	-5.43182	0.96806	-3.05534
H	-5.04909	0.18842	-4.61466
H	-1.36934	-2.16752	-6.29015
H	-5.77627	-2.18548	-3.88124
H	-6.45015	-1.33869	-2.44644
H	-5.37535	-2.75292	-2.22779
H	-1.56013	-5.20219	-1.3698
H	0.15279	-5.61246	-1.03119
H	-0.48024	-5.7044	-2.71135
H	1.5603	-4.17391	-3.34418
H	2.02446	-3.98685	-1.62846
H	1.67103	-2.53616	-2.62017

Table S21. Cartesian geometry of TS2 (24.5 kcal/mol) in Figure S104 in Angstrom [Å].

Atomtype	X Coordinates	Y Coordinates	Z Coordinates
C	0	0	0
C	0	0	1.43901
C	1.274128	0	2.11135
C	2.460529	0.073792	1.354619
C	2.448475	0.099765	-0.04451
C	1.21582	0.040942	-0.70666
N	-1.22061	0.03008	2.158321

Si	-1.71423	1.449932	3.043292
C	-3.55344	1.905347	2.708575
C	-3.89871	3.289746	3.288004
C	1.407987	-0.13549	3.628404
C	2.324858	-1.30745	4.025709
C	-1.29818	-0.07925	-0.79526
C	-1.40635	0.975406	-1.91005
Sn	-2.77842	-1.55104	1.935143
C	-1.4856	-3.15901	2.807229
C	-0.11732	-3.24521	2.456911
C	0.680858	-4.31359	2.900822
C	0.138592	-5.30716	3.737747
C	-1.21808	-5.24698	4.092296
C	-2.02109	-4.19882	3.601902
Ni	-3.34665	-1.87347	4.703075
P	-2.04532	-0.4522	5.675558
C	-1.58906	1.195287	4.951802
C	-5.02639	-2.73806	4.763884
N	-6.22919	-2.09595	4.51373
C	-7.31288	-2.77768	5.069799
C	-6.80954	-3.90936	5.646796
N	-5.42992	-3.88431	5.441498
C	-6.44586	-0.92246	3.701624
C	-6.61286	0.323444	4.347513
C	-7.01719	1.42002	3.560085
C	-7.21902	1.278634	2.182796
C	-6.99373	0.042888	1.558881
C	-6.61037	-1.09017	2.30034
C	-6.37962	0.482458	5.843175
C	-7.70471	0.504868	6.629014
C	-6.42389	-2.44629	1.622106
C	-6.02126	-2.33949	0.143372
C	-4.64159	-5.07085	5.679636
C	-4.62448	-6.0541	4.654319
C	-3.95276	-7.26239	4.919945
C	-3.31712	-7.48076	6.151386
C	-3.34166	-6.48949	7.140618
C	-4.0137	-5.2677	6.930459
C	-5.35327	-5.85035	3.328837
C	-4.50479	-6.23404	2.103812
C	-4.10996	-4.24192	8.048386
C	-5.02424	-4.75021	9.180694
C	-0.47023	-1.16534	6.344705
C	-0.19321	-2.52433	6.101297
C	0.911532	-3.1436	6.708133
C	1.75039	-2.41083	7.562558
C	1.492625	-1.04612	7.790773
C	0.389848	-0.42551	7.183331
C	-2.85072	0.071906	7.274225
C	-2.46271	1.238008	7.969802
C	-3.08001	1.582973	9.1831
C	-4.09979	0.774016	9.71704
C	-4.49839	-0.38273	9.026406
C	-3.87618	-0.72347	7.815467
C	-6.69257	-6.61619	3.336041

C	-2.72738	-3.82169	8.573344
C	-7.68762	-3.32379	1.75423
C	-5.52703	1.719615	6.159984
C	-0.5608	2.919766	2.58784
C	-0.83926	3.48837	1.185813
C	-0.46833	4.036713	3.642668
C	1.943722	1.134965	4.319063
C	-1.49815	-1.49837	-1.36517
C	-3.94973	1.829352	1.222523
H	-3.72779	-0.8817	3.57394
H	-2.26025	1.94508	5.419832
H	-3.05919	-2.6184	5.946215
H	0.192074	0.637814	7.386696
H	-0.55903	1.441653	5.278554
H	0.392652	-0.35834	4.013517
H	-4.17707	-1.61317	7.245647
H	-4.16012	1.149722	3.258776
H	-7.29681	-4.73505	6.172762
H	-2.1205	0.12168	-0.079
H	1.192905	0.042975	-1.80867
H	-2.84039	-6.66701	8.104612
H	3.426933	0.093176	1.884477
H	0.437121	2.4354	2.521261
H	-8.3342	-2.39592	4.988202
H	2.152308	-0.46129	8.450991
H	0.340443	-2.46579	1.827155
H	-5.58371	-4.76964	3.238312
H	-4.59383	-3.34599	7.613541
H	-0.8502	-3.09955	5.435575
H	-4.97612	2.224126	1.068757
H	-3.94788	0.783363	0.85284
H	-3.26697	2.413831	0.574741
H	-1.68214	1.89705	7.560953
H	2.962134	1.380572	3.949096
H	2.01961	0.97109	5.414238
H	1.312456	2.025243	4.149234
H	1.956642	-2.26971	3.631139
H	2.375193	-1.39329	5.128374
H	3.360632	-1.15265	3.656438
H	3.390042	0.153156	-0.61334
H	0.302179	4.787625	3.357812
H	-0.19093	3.652563	4.646822
H	-1.42691	4.583691	3.756449
H	1.739725	-4.36824	2.599109
H	-6.52091	-7.70821	3.441875
H	-7.24615	-6.44744	2.389528
H	-7.344	-6.2937	4.172836
H	-3.08958	-4.19362	3.852907
H	-5.30425	-1.01988	9.424128
H	-5.80464	-0.40299	6.180042
H	-3.40678	4.10288	2.714649
H	-3.59517	3.406314	4.349596
H	-4.99399	3.47437	3.240908
H	-2.79274	-8.43109	6.337363
H	-3.5334	-5.70204	2.098713

H	-5.04751	-5.97175	1.172095
H	-4.29976	-7.3246	2.067258
H	-0.71457	-1.73723	-2.11597
H	-2.49084	-1.60056	-1.85328
H	-1.4325	-2.27041	-0.56924
H	2.614581	-2.8958	8.04319
H	-1.26027	2.000502	-1.51325
H	-2.40798	0.933588	-2.38767
H	-0.65288	0.815591	-2.71042
H	-5.59134	-2.95575	2.157239
H	-3.92138	-8.04233	4.14418
H	-1.81008	4.024527	1.133978
H	-0.84951	2.687344	0.420086
H	-0.04955	4.214695	0.891221
H	-1.66185	-6.01579	4.740474
H	1.111292	-4.20535	6.497802
H	0.771121	-6.1341	4.099175
H	-2.76552	2.495663	9.713409
H	-4.58595	1.051413	10.6655
H	-2.82714	-3.02239	9.335395
H	-2.09463	-3.42825	7.75344
H	-2.19154	-4.67264	9.043914
H	-4.60639	-5.66043	9.660458
H	-6.03603	-5.00511	8.802815
H	-5.13545	-3.97509	9.967689
H	-7.12093	-0.04346	0.47067
H	-7.17081	2.399105	4.037952
H	-4.57157	1.688893	5.603288
H	-5.29091	1.759005	7.241483
H	-6.04698	2.663052	5.892989
H	-7.53795	2.14481	1.582482
H	-8.33112	1.372471	6.331207
H	-7.50497	0.58469	7.718093
H	-8.29992	-0.41501	6.458538
H	-7.94816	-3.53594	2.808533
H	-7.53322	-4.2974	1.244666
H	-8.55748	-2.82478	1.277424
H	-6.85633	-1.96552	-0.4861
H	-5.74365	-3.34208	-0.24169
H	-5.14337	-1.67584	0.011579

Table S22. Cartesian geometry of TS3 (-2.8 kcal/mol) in Figure S104 in Angstrom [Å].

Atomtype	X Coordinates	Y Coordinates	Z Coordinates
C	4.11465	-1.31526	-1.59897
C	3.78825	-0.04689	-1.0248
C	4.61383	1.08486	-1.33734
C	5.75552	0.91028	-2.14097
C	6.1011	-0.34754	-2.65677
C	5.27175	-1.44471	-2.39193
N	2.66318	0.11913	-0.17447
Si	2.69239	-0.40219	1.49463
C	1.85618	0.87985	2.65033
C	2.02862	0.51826	4.13614
C	4.2314	2.47469	-0.84107
C	5.42463	3.32478	-0.3748

C	3.19754	-2.51631	-1.41504
C	2.6266	-2.98637	-2.76437
Sn	0.79533	0.75446	-1.1943
Ni	-0.97586	-0.09506	0.41374
C	-2.3026	1.08831	-0.15379
N	-3.25752	0.97747	-1.14577
C	-4.27181	1.92537	-0.99437
C	-3.94107	2.68201	0.099
N	-2.73502	2.17315	0.58283
C	-3.13486	0.10018	-2.28158
C	-3.70427	-1.19333	-2.21634
C	-3.60251	-2.0079	-3.36109
C	-2.94225	-1.55415	-4.51058
C	-2.36577	-0.27622	-4.53914
C	-2.4534	0.58576	-3.42888
C	-1.96365	2.8065	1.62157
C	-1.37803	4.06081	1.32539
C	-0.71157	4.73257	2.36984
C	-0.60093	4.15519	3.63948
C	-1.12272	2.87484	3.88126
C	-1.81073	2.16677	2.87892
C	-4.43028	-1.6756	-0.96632
C	-5.93344	-1.33573	-1.04169
C	-1.8614	1.99342	-3.4758
C	-0.63207	2.10585	-4.39151
C	-1.41875	4.66866	-0.07441
C	-0.00274	4.99645	-0.58466
C	-2.32916	0.75197	3.1073
C	-3.86611	0.67714	3.11428
P	-0.0279	-1.89881	1.04996
C	1.65856	-2.00414	1.78385
C	-1.13402	-2.69634	2.30819
C	-2.51992	-2.49679	2.13836
C	-3.44219	-3.07809	3.01971
C	-2.98682	-3.85923	4.09744
C	-1.60714	-4.05714	4.27908
C	-0.68435	-3.48276	3.38695
C	-0.08579	-3.20654	-0.26025
C	-0.6303	-2.88379	-1.51874
C	-0.7938	-3.88142	-2.49431
C	-0.419	-5.20613	-2.21838
C	0.13048	-5.53253	-0.96378
C	0.29558	-4.53733	0.01182
C	-2.33957	5.90248	-0.13191
C	-1.7398	0.06985	4.34442
C	-4.21442	-3.17216	-0.6932
C	-2.92049	3.03695	-3.8927
C	3.42685	3.23101	-1.91761
C	3.87521	-3.67448	-0.66302
C	4.49238	-0.84827	1.9855
C	5.36196	0.3954	2.23319
C	4.60803	-1.85853	3.14079
C	2.27174	2.3319	2.37797
H	0.49052	2.05303	0.07988
H	0.70751	-4.80201	0.99896

H	2.18707	-2.90134	1.40542
H	1.55248	-2.11881	2.88221
H	2.34223	-2.16542	-0.80507
H	-2.85146	-1.85088	1.3101
H	0.76877	0.79612	2.39781
H	-5.12895	1.97239	-1.67151
H	3.55695	2.31561	0.02359
H	6.39231	1.78054	-2.3672
H	-4.03332	-3.01944	-3.34418
H	5.52163	-2.42993	-2.82002
H	4.88268	-1.34066	1.06472
H	-4.44813	3.52538	0.57595
H	0.42785	-6.57005	-0.74387
H	-1.53193	2.2368	-2.44078
H	-4.00362	-1.10659	-0.11174
H	-0.93068	-1.84207	-1.71674
H	1.77275	3.02401	3.08923
H	1.96639	2.63629	1.35665
H	3.36723	2.47921	2.47565
H	0.39305	-3.6499	3.53958
H	4.72768	-4.09058	-1.24154
H	3.15471	-4.50116	-0.487
H	4.2741	-3.34565	0.31851
H	2.0523	-2.17281	-3.25334
H	1.94612	-3.84935	-2.62371
H	3.43474	-3.29681	-3.46063
H	7.00541	-0.46735	-3.27434
H	5.67254	-2.1205	3.33335
H	4.07241	-2.8069	2.92788
H	4.19857	-1.45398	4.09067
H	-3.29532	2.82401	-4.91606
H	-2.47709	4.05455	-3.89513
H	-3.7885	3.05234	-3.20669
H	-4.52145	-2.91287	2.87337
H	-1.95555	0.16556	2.21672
H	3.08841	0.6056	4.45705
H	1.69859	-0.5159	4.37223
H	1.44007	1.20622	4.78297
H	-2.86666	-2.20776	-5.39395
H	0.12122	1.32746	-4.15595
H	-0.14831	3.09413	-4.25208
H	-0.90833	2.02418	-5.46414
H	4.0378	3.39	-2.83211
H	3.08929	4.22182	-1.54557
H	2.5243	2.65857	-2.2267
H	-0.54775	-5.98849	-2.98285
H	6.04528	2.78714	0.3707
H	5.06799	4.26641	0.09319
H	6.08876	3.61159	-1.21813
H	-1.83515	3.90241	-0.76023
H	-1.83463	0.05886	-5.44142
H	5.04818	0.94128	3.14854
H	5.30882	1.10399	1.38414
H	6.43128	0.11843	2.36744
H	-1.21391	-3.61139	-3.47417

H	-1.24387	-4.66554	5.12239
H	-3.70693	-4.31077	4.79757
H	-4.66457	-3.45651	0.2788
H	-3.13695	-3.42729	-0.65966
H	-4.69371	-3.80453	-1.46953
H	-6.41092	-1.8541	-1.89995
H	-6.10239	-0.24764	-1.16803
H	-6.45468	-1.65481	-0.11493
H	-0.2526	5.71385	2.17397
H	-0.98629	2.41524	4.86974
H	-0.63342	0.07094	4.31851
H	-2.0764	-0.98354	4.39166
H	-2.06688	0.56539	5.28341
H	-0.07597	4.69229	4.44472
H	-4.27835	1.28409	3.94832
H	-4.19623	-0.3708	3.26089
H	-4.31234	1.04674	2.17092
H	-3.37419	5.66033	0.18673
H	-2.38629	6.31038	-1.16326
H	-1.96703	6.70934	0.5341
H	0.47431	5.80514	0.00775
H	-0.0424	5.33562	-1.64092
H	0.64022	4.09495	-0.52917

Table S23. Cartesian geometry of TS4 (3.6 kcal/mol) in Figure S104 in Angstrom [Å].

Atomtype	X Coordinates	Y Coordinates	Z Coordinates
C	-1.13441	-3.53782	3.32972
C	-1.53249	-2.64937	2.30931
C	-2.88857	-2.28565	2.21052
C	-3.84012	-2.81247	3.09692
C	-3.43788	-3.69758	4.11258
C	-2.08232	-4.05461	4.22902
P	-0.36885	-1.90982	1.06753
C	-0.2698	-3.30123	-0.15575
C	0.30048	-4.55442	0.14256
C	0.32338	-5.57089	-0.82725
C	-0.23449	-5.34526	-2.09936
C	-0.81362	-4.10029	-2.39912
C	-0.8253	-3.08111	-1.43344
Ni	-0.98681	-0.06481	0.08941
C	-2.16742	1.28137	-0.32425
N	-2.53604	2.45368	0.31095
C	-3.64939	3.04611	-0.29829
C	-3.98548	2.25447	-1.36198
N	-3.0666	1.20332	-1.38149
C	-1.87292	3.0621	1.43315
C	-1.20635	4.29526	1.20479
C	-0.70364	4.98264	2.32605
C	-0.83383	4.44472	3.61425
C	-1.43772	3.19346	3.80164
C	-1.97541	2.46928	2.7181
C	-2.98421	0.20375	-2.41346
C	-3.68825	-1.01516	-2.2501
C	-3.62631	-1.95419	-3.29846
C	-2.88411	-1.69223	-4.45749

C	-2.18129	-0.48688	-4.58841
C	-2.21606	0.4904	-3.57435
C	-0.96421	4.79834	-0.21847
C	-0.73197	6.31279	-0.31442
C	-2.62009	1.10014	2.90297
C	-2.28853	0.44393	4.24741
C	-4.5032	-1.29423	-0.99268
C	-4.52758	-2.78483	-0.62241
C	-1.46418	1.80987	-3.73325
C	-2.38653	2.93518	-4.25116
Sn	0.94589	0.1874	-1.40251
N	2.63921	0.0155	-0.05085
Si	2.39231	-0.42851	1.63731
C	1.42908	0.96527	2.526
C	1.864	2.37558	2.11431
C	3.91371	-0.09989	-0.6746
C	4.70252	1.05551	-0.97667
C	5.98512	0.88154	-1.53254
C	6.49672	-0.39208	-1.81706
C	5.69687	-1.52003	-1.58325
C	4.40727	-1.39732	-1.03332
C	4.16698	2.46716	-0.75774
C	4.04518	3.22829	-2.09224
C	3.50907	-2.62583	-0.91203
C	4.19984	-3.85513	-0.30339
C	4.078	-0.90824	2.4156
C	3.96068	-1.7375	3.70822
C	1.26842	-1.95955	1.92562
C	5.02406	0.28926	2.59829
C	2.87515	-2.95779	-2.27674
C	4.99893	3.2818	0.2496
C	0.20579	4.01802	-0.85532
C	-4.14409	1.13117	2.67505
C	-5.93625	-0.73803	-1.12477
C	-0.23502	1.69639	-4.64944
C	1.43188	0.80758	4.05483
H	1.79817	1.41927	-2.41517
H	0.72827	-4.74613	1.13952
H	1.7991	-2.90734	1.70363
H	1.06401	-1.94811	3.01673
H	2.67218	-2.35041	-0.24229
H	-3.17237	-1.55365	1.43868
H	0.38033	0.81681	2.15096
H	-4.79541	2.33504	-2.09212
H	3.14853	2.34705	-0.34052
H	6.59805	1.77202	-1.74976
H	-4.15802	-2.91107	-3.19903
H	6.07917	-2.52021	-1.84461
H	4.5314	-1.56433	1.63684
H	-4.10627	3.95731	0.09889
H	0.77741	-6.54557	-0.58837
H	-1.11363	2.10399	-2.71949
H	-4.00561	-0.74125	-0.16542
H	-1.26042	-2.0935	-1.66009
H	1.24702	3.14563	2.6196

H	1.75578	2.52162	1.02396
H	2.92647	2.57178	2.36952
H	-0.07662	-3.82761	3.4315
H	5.01034	-4.24661	-0.95438
H	3.46606	-4.67679	-0.16388
H	4.64798	-3.61975	0.68438
H	2.29296	-2.09138	-2.66371
H	2.17641	-3.81563	-2.19518
H	3.65156	-3.19774	-3.03405
H	7.50762	-0.50442	-2.23989
H	4.9678	-2.04373	4.06914
H	3.36919	-2.6652	3.56575
H	3.4847	-1.16352	4.53068
H	-2.79637	2.67346	-5.24951
H	-1.81654	3.88228	-4.35274
H	-3.2365	3.12831	-3.5702
H	-4.89775	-2.51681	3.00785
H	-2.16608	0.46483	2.09513
H	2.45174	0.95203	4.47126
H	1.07769	-0.19077	4.38818
H	0.77791	1.56832	4.53313
H	-2.84703	-2.43873	-5.26658
H	0.40627	0.8334	-4.38225
H	0.38796	2.60921	-4.56577
H	-0.52999	1.59163	-5.71499
H	5.03981	3.39596	-2.5585
H	3.57605	4.22321	-1.9338
H	3.41505	2.66291	-2.80868
H	-0.21331	-6.14295	-2.8586
H	5.05518	2.78273	1.23636
H	4.55052	4.28635	0.40354
H	6.03949	3.43253	-0.1101
H	-1.86898	4.5674	-0.81969
H	-1.5906	-0.30175	-5.49671
H	4.61348	1.03777	3.30967
H	5.21409	0.80016	1.63491
H	6.00948	-0.03746	2.99866
H	-1.25116	-3.90946	-3.39085
H	-1.76054	-4.74293	5.0267
H	-4.17842	-4.10285	4.81979
H	-5.02712	-2.93267	0.35527
H	-3.50408	-3.20365	-0.54789
H	-5.09278	-3.38307	-1.36747
H	-6.47452	-1.2327	-1.96079
H	-5.93828	0.35295	-1.31693
H	-6.51111	-0.91694	-0.19214
H	-0.18685	5.9432	2.1887
H	-1.50489	2.77331	4.81471
H	-1.19578	0.38329	4.41176
H	-2.68784	-0.58832	4.27068
H	-2.73796	0.99309	5.10286
H	-0.4368	4.9957	4.48113
H	-4.64086	1.7958	3.41394
H	-4.56692	0.11322	2.80026
H	-4.41572	1.4852	1.66151

H	-1.55204	6.88776	0.16316
H	-0.67014	6.62171	-1.37798
H	0.22255	6.61534	0.16534
H	1.16246	4.25205	-0.34529
H	0.31854	4.26842	-1.93006
H	0.0463	2.92368	-0.77248

Table S24. Cartesian geometry of complex **4** in Figure S101 in Angstrom [Å].

Atomtype	X Coordinates	Y Coordinates	Z Coordinates
C	-2.46031	3.38561	-0.73992
C	-2.34876	3.60093	0.65659
C	-1.8936	4.86219	1.10405
C	-1.55933	5.87234	0.18829
C	-1.67573	5.64436	-1.19513
C	-2.12706	4.39673	-1.65619
C	-2.71479	2.525	1.61525
C	-2.28943	1.18599	1.39782
C	-2.69967	0.18274	2.29813
C	-3.50867	0.4863	3.40571
C	-3.91322	1.81441	3.62894
C	-3.51842	2.82613	2.74014
Sn	-0.73733	0.77628	-0.1019
N	-1.24994	-1.23721	-0.60952
Si	-0.04985	-1.99324	-1.67637
C	1.58914	-2.2755	-0.72945
P	2.19454	-0.78531	0.22365
Ni	1.6104	1.25424	-0.25952
C	3.14002	1.83131	-1.59336
C	4.22427	2.56416	-0.81295
C	4.05927	2.49386	0.73115
C	2.62939	2.31537	1.20984
C	1.54832	3.10289	0.77519
C	1.70229	4.24863	-0.21667
C	1.46664	3.81784	-1.68373
C	1.93012	2.40932	-1.99554
C	4.03065	-1.0033	0.1413
C	4.64817	-1.19821	-1.11495
C	6.04638	-1.20886	-1.23559
C	6.8553	-1.02206	-0.10052
C	6.25195	-0.82859	1.15395
C	4.85242	-0.81393	1.27436
C	1.85608	-1.29638	1.97792
C	2.40171	-2.48293	2.51635
C	2.19866	-2.81037	3.86591
C	1.43305	-1.96516	4.69145
C	0.86097	-0.79987	4.15674
C	1.08179	-0.46581	2.80994
C	-2.4336	-1.9245	-0.23737
C	-2.37454	-3.02983	0.67636
C	-3.53201	-3.79853	0.90497
C	-4.75559	-3.47775	0.30426
C	-4.83589	-2.33459	-0.50084
C	-3.7059	-1.53973	-0.77333
C	-1.11386	-3.34476	1.48268
C	-0.46497	-4.6977	1.13389

C	-3.89968	-0.27289	-1.60179
C	-4.30947	-0.56318	-3.05694
C	-1.40249	-3.30686	2.99746
C	-4.91034	0.67731	-0.93018
C	-0.71388	-3.66864	-2.32112
C	0.40136	-4.64558	-2.73676
C	0.47546	-0.79284	-3.07894
C	-0.65209	0.01436	-3.73309
C	-1.77101	-3.5	-3.42509
C	1.37315	-1.47009	-4.12889
H	1.27214	-2.22581	5.74927
H	2.63858	-3.73261	4.27734
H	0.24226	-0.14183	4.78625
H	-2.13856	-4.08544	3.28842
H	-0.47168	-3.48894	3.56878
H	-1.80285	-2.32609	3.32034
H	-1.15938	-5.53937	1.34282
H	0.44685	-4.85495	1.7479
H	-3.47364	-4.66335	1.58593
H	-3.82577	-0.3159	4.09125
H	3.01175	-3.14359	1.87956
H	0.65466	0.4589	2.38949
H	-2.39096	-0.85815	2.12995
H	4.3931	-0.654	2.26149
H	-5.64806	-4.09649	0.4866
H	0.37914	3.86611	-1.89118
H	6.87567	-0.68423	2.05043
H	-0.38195	-2.54275	1.25995
H	0.97967	5.05152	0.03503
H	-0.17274	-4.76806	0.06971
H	-4.55294	2.06231	4.4908
H	0.65394	3.14224	1.4221
H	1.94368	4.54964	-2.38101
H	1.52523	-3.13935	-0.03833
H	1.39733	1.9186	-2.82824
H	-5.80741	-2.04752	-0.93524
H	2.7118	4.69869	-0.10413
H	-4.61075	0.91525	0.10919
H	7.95243	-1.03059	-0.19379
H	-1.24156	-4.1072	-1.44625
H	-5.923	0.22388	-0.89513
H	-3.86527	3.86059	2.89516
H	-2.62579	-2.89049	-3.07146
H	2.36784	-2.5296	-1.47816
H	4.25543	3.62337	-1.14698
H	-2.9139	0.24038	-1.64267
H	2.52699	1.79562	2.17906
H	-2.17904	-4.48663	-3.73744
H	-4.99763	1.6289	-1.49616
H	3.46533	0.93097	-2.14032
H	1.12445	-4.83504	-1.91604
H	4.03043	-1.34108	-2.01629
H	-1.78105	5.03909	2.1851
H	-5.28371	-1.09483	-3.09909
H	6.50692	-1.36395	-2.22408

H	-0.02574	-5.62927	-3.03281
H	4.50853	3.4037	1.19799
H	-2.88324	2.43718	-1.10305
H	-3.56024	-1.1939	-3.57298
H	1.10586	-0.07046	-2.50847
H	-1.35544	-3.01552	-4.33409
H	2.21166	-2.03687	-3.6712
H	5.20966	2.13122	-1.08153
H	4.6529	1.6405	1.11037
H	0.982	-4.27016	-3.60485
H	-4.41512	0.38037	-3.63284
H	-1.2058	0.62071	-2.98486
H	0.80442	-2.18394	-4.76199
H	-1.39478	-0.63238	-4.24342
H	-1.19885	6.8455	0.55706
H	1.82368	-0.71679	-4.81241
H	-0.25018	0.72131	-4.49285
H	-2.23471	4.20967	-2.7359
H	-1.41159	6.43767	-1.91131

Table S25. Cartesian geometry of complex **7'** (-6.5 kcal/mol) in Figure S105 in Angstrom [Å].

Atomtype	X Coordinates	Y Coordinates	Z Coordinates
C	-1.60174	2.49486	2.5611
C	-2.32556	2.41799	1.34278
C	-2.40956	3.49328	0.42588
C	-1.75734	4.69372	0.77184
C	-1.03627	4.80092	1.96879
C	-0.95095	3.71026	2.84808
N	-3.02408	1.20238	1.02664
C	-2.46438	0.1946	0.26065
N	-3.51412	-0.69541	0.10844
C	-4.68491	-0.22657	0.71581
C	-4.37457	0.97564	1.29092
Ni	-0.87635	0.38299	-0.67016
Sn	0.94858	0.70961	0.89768
N	2.22154	-0.87985	0.21526
C	3.42125	-1.0484	0.96099
C	3.48804	-2.03934	1.99141
C	4.68284	-2.202	2.7156
C	5.80649	-1.40295	2.45294
C	5.72832	-0.40816	1.46995
C	4.55078	-0.20602	0.72279
C	2.25106	-2.86807	2.31129
C	2.55103	-4.28775	2.81335
C	4.47819	0.94517	-0.2727
C	4.5535	2.30099	0.45418
C	-3.46544	-1.93606	-0.61389
C	-3.27164	-1.91367	-2.02232
C	-3.34553	-3.14348	-2.70212
C	-3.60016	-4.34198	-2.01579
C	-3.75062	-4.3378	-0.62443
C	-3.6732	-3.13542	0.10917
C	-3.00903	-0.60731	-2.76094
C	-4.28173	0.24937	-2.88861
C	-3.77919	-3.16077	1.63248

C	-2.65399	-4.00514	2.26268
C	-3.19227	3.37715	-0.87759
C	-4.47788	4.22557	-0.80985
C	-1.55493	1.30678	3.52045
C	-0.3125	1.29524	4.42434
P	0.53531	1.05	-2.17041
C	1.85842	-0.16728	-2.69411
Si	1.98796	-1.56531	-1.40014
C	3.50766	-2.66579	-1.79188
C	3.4722	-4.0245	-1.07134
C	1.4781	2.59355	-1.85553
C	1.02406	3.50018	-0.86133
C	1.6626	4.73113	-0.65107
C	2.7715	5.09791	-1.43249
C	3.22975	4.21417	-2.42895
C	2.59393	2.98155	-2.63918
C	0.29992	-2.45892	-1.55557
C	0.226	-3.27316	-2.85692
C	-0.13751	-3.28834	-0.34585
C	1.33031	-2.12551	3.29905
C	5.52636	0.83943	-1.39235
C	-2.30092	-0.77075	-4.10773
C	-5.16835	-3.6426	2.09332
C	-2.34172	3.72579	-2.11173
C	-2.8334	1.23738	4.38416
C	3.78758	-2.82475	-3.29724
H	2.96417	2.31331	-3.43256
H	2.83634	0.3446	-2.79919
H	1.5987	-0.58583	-3.6893
H	3.48107	0.89429	-0.75215
H	-0.42355	-1.5955	-1.62884
H	-4.98869	1.69367	1.84156
H	1.68956	-2.95017	1.36095
H	4.73804	-2.97055	3.50228
H	-1.79845	5.54885	0.07983
H	6.60127	0.23809	1.28035
H	4.35051	-2.08263	-1.35275
H	-5.62162	-0.78951	0.67455
H	4.09698	4.48888	-3.05086
H	-1.52683	0.39078	2.88721
H	-3.49844	2.31786	-0.98909
H	0.13922	3.23825	-0.26024
H	-1.12898	-3.75275	-0.52322
H	-0.22261	-2.64987	0.55665
H	0.57707	-4.10742	-0.11906
H	6.56223	0.90326	-0.99568
H	5.39618	1.66494	-2.12345
H	5.43675	-0.12244	-1.9387
H	3.75386	2.38039	1.22081
H	4.42055	3.13823	-0.26016
H	5.52796	2.4315	0.97155
H	6.73715	-1.54926	3.02372
H	4.72825	-3.3946	-3.46809
H	3.89741	-1.84574	-3.80786
H	2.97614	-3.3783	-3.81507

H	-2.93088	2.15091	5.00779
H	-2.79393	0.36105	5.0643
H	-3.74909	1.14378	3.7694
H	-2.28716	-0.0334	-2.11948
H	0.89611	-4.1582	-2.81354
H	0.52228	-2.68236	-3.74891
H	-0.8028	-3.65312	-3.03189
H	-0.5249	5.74365	2.21877
H	0.62211	1.39755	3.8353
H	-0.25864	0.33944	4.9835
H	-0.34244	2.11352	5.17446
H	1.8381	-1.95802	4.27241
H	0.39488	-2.69598	3.48469
H	1.04208	-1.12518	2.90752
H	3.27885	6.06084	-1.26657
H	3.23643	-4.82656	2.12718
H	1.6122	-4.87541	2.89028
H	3.01714	-4.28613	3.82176
H	-3.64358	-2.12292	1.99861
H	-0.3641	3.80542	3.77264
H	2.64061	-4.66368	-1.43801
H	3.34861	-3.90449	0.02252
H	4.41587	-4.59079	-1.23569
H	1.287	5.40292	0.13679
H	-2.95795	3.64828	-3.0318
H	-1.48677	3.0279	-2.22186
H	-1.93934	4.75893	-2.06577
H	-4.24239	5.30675	-0.7145
H	-5.10549	3.94144	0.06004
H	-5.08426	4.09061	-1.72974
H	-3.91485	-5.28661	-0.09017
H	-3.20972	-3.16185	-3.79291
H	-1.40421	-1.41311	-4.01337
H	-1.95749	0.22177	-4.46383
H	-2.96349	-1.20423	-4.88708
H	-3.6622	-5.28941	-2.57346
H	-5.03362	-0.25842	-3.52952
H	-4.03979	1.22829	-3.35098
H	-4.75314	0.44583	-1.90509
H	-5.98091	-3.01798	1.66816
H	-5.25052	-3.60534	3.19954
H	-5.35707	-4.6903	1.77754
H	-2.70867	-5.06703	1.94351
H	-2.72899	-3.98345	3.36988
H	-1.65731	-3.6175	1.97583

Table S26. Cartesian geometry of complex **7** (-13.3 kcal/mol) in Figure S105 in Angstrom [Å].

Atomtype	X Coordinates	Y Coordinates	Z Coordinates
C	3.57692	-4.81597	-1.08663
C	3.57019	-3.76738	-0.11695
C	4.69373	-3.62641	0.75789
C	5.77415	-4.52072	0.65428
C	5.77494	-5.55332	-0.29476
C	4.68107	-5.68845	-1.15815
N	2.46688	-2.87073	0.0044

Sn	2.40309	-1.10638	-1.24323
Ni	0.46263	0.01055	0.01064
Ni	-1.99151	-0.05713	0.0765
C	-3.84951	-0.11752	0.00532
N	-4.68705	-1.20153	0.23457
C	-6.0311	-0.88279	0.03608
C	-6.07064	0.43138	-0.32992
N	-4.74885	0.88372	-0.34279
C	-4.27851	-2.49631	0.70874
C	-3.96216	-3.50653	-0.23511
C	-3.6607	-4.78695	0.27019
C	-3.67736	-5.04473	1.6491
C	-3.99048	-4.02498	2.55792
C	-4.30238	-2.72624	2.10784
C	-4.42767	2.25748	-0.62503
C	-4.52179	2.70839	-1.96671
C	-4.27678	4.07389	-2.21748
C	-3.97495	4.95331	-1.17159
C	-3.9109	4.48489	0.14936
C	-4.13744	3.1296	0.4595
C	-3.94049	-3.20563	-1.73119
C	-5.35734	-3.16367	-2.34265
C	-4.69833	-1.6201	3.08483
C	-3.98689	-1.72074	4.44256
C	-4.95891	1.78261	-3.10077
C	-6.46714	1.9583	-3.38794
C	-4.06527	2.61792	1.8974
C	-3.35419	3.58488	2.85353
C	4.72188	-2.48074	1.75569
C	5.3278	-1.2275	1.10451
C	2.42868	-4.98747	-2.07062
C	1.798	-6.39007	-2.03444
P	-0.82268	1.53735	0.92322
C	-0.64075	3.25768	0.25867
Si	0.81658	3.37045	-0.97098
C	0.91228	5.16061	-1.64793
C	-0.46347	5.84104	-1.76486
C	-0.6872	1.66717	2.74658
C	0.15803	2.5865	3.40694
C	0.30253	2.54666	4.80364
C	-0.40322	1.59513	5.55936
C	-1.25504	0.68205	4.90918
C	-1.39295	0.7152	3.51375
C	0.4061	2.07066	-2.30697
C	1.45507	1.74837	-3.37386
N	2.29326	3.01875	-0.04953
C	3.37689	3.94373	-0.0444
C	4.52254	3.71272	-0.86989
C	5.55144	4.67393	-0.91218
C	5.48793	5.84787	-0.15116
C	4.38951	6.05367	0.6945
C	3.33797	5.12126	0.76876
C	4.68143	2.42222	-1.66114
C	5.77667	1.53952	-1.03635
C	2.22073	5.33481	1.78249

C	2.72514	4.9654	3.1924
C	-0.96081	2.36355	-2.93623
Sn	2.37701	1.26996	1.21773
P	-0.78121	-1.57773	-0.84397
C	-0.85209	-1.69702	-2.67523
C	0.0365	-2.47952	-3.44298
C	-0.03576	-2.47707	-4.84504
C	-1.00866	-1.70117	-5.49946
C	-1.89991	-0.91909	-4.74236
C	-1.81559	-0.90933	-3.34137
C	4.94143	2.6544	-3.15835
C	1.6118	6.7461	1.76314
C	-0.45457	-3.29198	-0.21844
Si	1.04194	-3.31394	0.96765
C	1.28653	-5.11033	1.59576
C	-0.02136	-5.91621	1.69489
C	0.55584	-2.08891	2.35787
C	1.62104	-1.60297	3.34484
C	-0.66778	-2.64968	3.09843
C	-3.06759	-4.17405	-2.54233
C	-6.22565	-1.59566	3.31368
C	-4.1703	1.99269	-4.4039
C	-5.45223	2.25358	2.46803
C	5.41601	-2.8039	3.08561
C	2.87112	-4.60482	-3.49599
C	1.72072	5.26472	-2.95242
C	2.1097	-5.18372	2.89342
H	-2.4952	-0.29293	-2.72926
H	-0.25284	-3.98303	-1.06235
H	-1.37351	-3.65	0.2874
H	1.65019	-4.26604	-1.75143
H	0.20668	-1.18754	1.77433
H	3.66269	-2.24497	1.97142
H	6.63377	-4.40703	1.33303
H	4.6794	-6.49449	-1.91027
H	1.90465	-5.57608	0.79584
H	-2.66323	-0.30922	-5.24823
H	0.78939	-3.10655	-2.94395
H	1.17775	-0.87267	4.0576
H	2.45685	-1.08285	2.83634
H	2.05967	-2.43132	3.93881
H	2.51132	-7.17125	-2.37268
H	0.91214	-6.43725	-2.70286
H	1.46849	-6.65856	-1.01018
H	3.22214	-3.55233	-3.53391
H	2.03672	-4.72284	-4.21963
H	3.70713	-5.24936	-3.83982
H	6.62701	-6.24793	-0.36154
H	0.18182	-6.9648	2.00825
H	-0.55885	-5.95762	0.72528
H	-0.72613	-5.48376	2.43537
H	-0.45052	-3.6189	3.59517
H	-1.53641	-2.80276	2.42791
H	-0.98034	-1.94745	3.89801
H	6.39187	-1.38679	0.82876

H	5.26582	-0.34708	1.7795
H	4.79438	-0.97143	0.16244
H	-1.07175	-1.70418	-6.59879
H	5.00542	-3.72605	3.54622
H	5.27343	-1.96986	3.80396
H	6.51106	-2.94576	2.96435
H	1.56603	-4.73856	3.75362
H	3.07722	-4.65192	2.79408
H	2.3406	-6.23836	3.16225
H	0.67285	-3.08622	-5.42772
H	-3.78628	6.01658	-1.3848
H	-4.3285	4.45256	-3.24918
H	-3.671	5.18938	0.95754
H	-6.67306	2.98781	-3.7495
H	-4.36069	2.9941	-4.84302
H	-3.96159	4.49566	3.04418
H	-7.09361	1.79188	-2.49096
H	-2.36679	3.90121	2.46671
H	-6.11304	3.14581	2.50241
H	-3.08157	1.89271	-4.24701
H	-6.80151	1.2472	-4.1719
H	-4.48653	1.24568	-5.16097
H	-3.17809	3.08812	3.82817
H	-4.78778	0.738	-2.76313
H	-5.96522	1.46969	1.87911
H	-5.34277	1.87276	3.50485
H	-6.91092	1.09073	-0.55916
H	-3.45937	1.68612	1.84505
H	-4.40669	-0.65434	2.61816
H	-4.18889	-0.80993	5.04211
H	-6.49743	-0.77965	4.01513
H	-6.8283	-1.61735	0.17543
H	-2.89261	-1.82567	4.32604
H	-6.78937	-1.4308	2.37683
H	-3.49699	-2.19182	-1.82556
H	-4.35039	-2.58708	5.03408
H	-6.56643	-2.55604	3.75492
H	-5.99563	-2.38587	-1.88267
H	-5.29237	-2.94204	-3.42828
H	-3.53391	-5.17938	-2.62042
H	-2.93517	-3.78531	-3.57178
H	-3.98654	-4.23942	3.63655
H	-5.86594	-4.14394	-2.22428
H	-2.06019	-4.29335	-2.10318
H	-3.39725	-5.59455	-0.42748
H	-3.43318	-6.05241	2.01892
H	-0.29153	1.56392	6.65427
H	0.97474	3.2631	5.30098
H	1.92516	5.09459	3.95246
H	3.57883	5.6122	3.48463
H	3.0769	3.91298	3.2271
H	-1.81474	-0.06075	5.49774
H	0.70468	3.34563	2.82858
H	4.3494	6.96013	1.32002
H	2.3424	7.51729	2.08671

H	0.74564	6.80481	2.45552
H	1.41853	4.61797	1.51348
H	6.29836	6.59175	-0.20367
H	-2.05007	0.00475	2.98489
H	1.25818	7.02313	0.74906
H	5.58222	1.36983	0.042
H	-0.49434	3.9835	1.08478
H	6.77552	2.01857	-1.11993
H	6.4262	4.49303	-1.55784
H	5.82598	0.55081	-1.53974
H	-1.59468	3.52162	-0.23841
H	1.48977	5.70551	-0.86935
H	5.91857	3.15335	-3.33165
H	3.71973	1.8773	-1.56381
H	-0.99561	5.87237	-0.79209
H	0.28478	1.13697	-1.67813
H	2.44132	1.51073	-2.93266
H	4.15414	3.28827	-3.61332
H	2.72703	4.80999	-2.8471
H	4.95919	1.68796	-3.70457
H	-0.35868	6.89016	-2.12193
H	-1.13198	5.31736	-2.4804
H	-1.78093	2.36236	-2.18959
H	1.13785	0.86381	-3.97013
H	1.86888	6.32734	-3.24679
H	1.60408	2.58979	-4.08162
H	1.20551	4.7596	-3.79675
H	-1.197	1.58604	-3.69087
H	-0.982	3.34396	-3.4589

Table S27. Cartesian geometry of tri-hydride complex (10.1 kcal/mol) in Figure S105 in Angstrom [Å].

Atomtype	X Coordinates	Y Coordinates	Z Coordinates
C	-0.42959	-3.01338	-0.66067
C	-0.18399	-2.75436	0.70241
C	-0.00023	-3.83705	1.5885
C	-0.06777	-5.15719	1.11785
C	-0.31325	-5.40836	-0.24521
C	-0.48589	-4.33477	-1.13275
P	-0.24113	-0.99571	1.28573
Ni	-0.66291	0.35698	-0.36355
C	-2.4868	0.76154	-0.4908
N	-3.49532	0.04528	-1.11981
C	-4.70976	0.74592	-1.09245
C	-4.4689	1.93188	-0.45223
N	-3.11496	1.9279	-0.10223
C	-3.29579	-1.17791	-1.86474
C	-3.83563	-2.39331	-1.37813
C	-3.67527	-3.55159	-2.16637
C	-2.99025	-3.51094	-3.38374
C	-2.45069	-2.3003	-3.84143
C	-2.60538	-1.10877	-3.10881
C	-2.37847	3.07808	0.36294
C	-1.79178	3.92817	-0.61057
C	-1.09035	5.05843	-0.15087
C	-0.98151	5.33572	1.21937

C	-1.56562	4.47654	2.1585
C	-2.26821	3.32523	1.75023
C	-4.57903	-2.50339	-0.05436
C	-3.93284	-3.56057	0.86099
C	-2.10089	0.21458	-3.6683
C	-3.24149	0.99238	-4.353
C	-1.95752	3.67373	-2.1031
C	-3.07791	4.564	-2.67726
C	-2.89456	2.39503	2.77481
C	-1.87974	1.94679	3.83663
C	-1.49996	-1.19245	2.6515
C	-2.84507	-0.90181	2.36899
C	-3.84532	-1.06442	3.34039
C	-3.50635	-1.51931	4.62519
C	-2.16251	-1.80862	4.92509
C	-1.16739	-1.64471	3.94774
C	1.30673	-0.74265	2.27283
Si	2.58557	0.47268	1.5101
C	1.65033	2.15106	1.54802
C	2.17882	3.19759	0.55568
C	4.13286	0.3809	2.63412
C	3.84063	0.15874	4.12818
N	3.08914	0.10961	-0.12359
C	4.32607	-0.50057	-0.45897
C	5.36514	0.26722	-1.079
C	6.61903	-0.32935	-1.3102
C	6.86972	-1.66507	-0.96596
C	5.83372	-2.4355	-0.42196
C	4.56038	-1.88451	-0.18025
C	5.10911	1.68951	-1.56503
C	4.9727	1.70089	-3.10247
C	3.43921	-2.79178	0.31104
C	3.729	-3.40546	1.69167
C	5.07871	1.57033	2.40377
Sn	1.50542	0.18861	-1.55874
C	-6.07769	-2.78609	-0.27674
C	-0.88676	0.07099	-4.59546
C	-0.64385	3.83787	-2.88397
C	-4.15144	3.01891	3.40943
C	6.17983	2.70303	-1.12343
C	3.12007	-3.88406	-0.7259
C	1.57679	2.72542	2.97348
H	0.17492	-3.65153	2.65919
H	1.76622	-1.71836	2.52748
H	1.00161	-0.2705	3.23079
H	2.5336	-2.16051	0.40158
H	-3.0937	-0.53119	1.36595
H	0.60661	1.88528	1.22706
H	-5.62175	0.33545	-1.53551
H	4.13778	1.99743	-1.12852
H	7.41966	0.26961	-1.77419
H	-4.08373	-4.50653	-1.80248
H	6.01225	-3.49831	-0.18832
H	4.6547	-0.52748	2.25232
H	-5.12617	2.77416	-0.21776

H	0.07206	-5.99552	1.81833
H	-1.75141	0.79797	-2.79396
H	-4.50285	-1.52282	0.4536
H	-0.59102	-2.16808	-1.34776
H	1.57833	4.1307	0.61093
H	2.13189	2.83787	-0.49197
H	3.23767	3.4605	0.76236
H	-0.12028	-1.86275	4.20726
H	4.61075	-4.0804	1.65837
H	2.8629	-4.00594	2.04202
H	3.94165	-2.62494	2.45083
H	2.84338	-3.43099	-1.69923
H	2.27095	-4.50944	-0.38497
H	3.99241	-4.55098	-0.89484
H	7.86242	-2.10914	-1.14153
H	4.7863	0.04727	4.70415
H	3.23917	-0.75645	4.30829
H	3.28852	1.01154	4.57641
H	-3.64703	0.42496	-5.21756
H	-2.87196	1.9692	-4.72882
H	-4.07814	1.19646	-3.65489
H	-4.8916	-0.82402	3.09293
H	-3.21439	1.48687	2.23089
H	2.57693	3.05805	3.32338
H	1.1977	1.99143	3.71467
H	0.90203	3.60666	3.01285
H	-2.86979	-4.42852	-3.98094
H	-0.08753	-0.54021	-4.12757
H	-0.45973	1.07073	-4.81354
H	-1.14666	-0.39495	-5.56974
H	5.93739	1.43284	-3.5852
H	4.67386	2.70676	-3.46754
H	4.20937	0.97542	-3.44575
H	-0.36238	-6.44494	-0.61404
H	6.30784	2.71926	-0.02263
H	5.9005	3.72754	-1.44829
H	7.17109	2.47664	-1.57117
H	-2.27169	2.61977	-2.22381
H	-1.91115	-2.27417	-4.79887
H	4.63214	2.5271	2.74925
H	5.32524	1.68213	1.33074
H	6.03719	1.43779	2.95293
H	-0.66974	-4.51793	-2.20106
H	-1.88539	-2.1602	5.93155
H	-4.28424	-1.64277	5.3949
H	-4.40679	-3.55104	1.86337
H	-2.85046	-3.37032	0.99591
H	-4.04577	-4.58188	0.44121
H	-6.23286	-3.75994	-0.78718
H	-6.55344	-2.00338	-0.90321
H	-6.61622	-2.82442	0.69326
H	-0.62043	5.7339	-0.88157
H	-1.47261	4.69641	3.23314
H	-0.9909	1.49642	3.35543
H	-2.32791	1.18348	4.50392

H	-1.53552	2.79332	4.46687
H	-0.42882	6.22619	1.55732
H	-3.90221	3.9405	3.97726
H	-4.62603	2.30332	4.11325
H	-4.90159	3.29306	2.639
H	-4.04012	4.39233	-2.15289
H	-3.23488	4.35107	-3.75531
H	-2.82447	5.64037	-2.57387
H	-0.27836	4.88604	-2.87566
H	-0.79226	3.55237	-3.94605
H	0.1517	3.18892	-2.46589
H	-0.43736	1.25721	-1.57396
H	1.72471	-1.29031	-2.47614
H	2.06487	1.41789	-2.66947

Table S28. Cartesian geometry of INT1 (8.8 kcal/mol) in Figure S105 in Angstrom [Å].

Atomtype	X Coordinates	Y Coordinates	Z Coordinates
C	-1.77248	3.92796	1.23792
C	-1.56697	3.41219	-0.06525
C	-0.55845	3.89126	-0.9365
C	0.22812	4.96906	-0.48688
C	0.02646	5.52459	0.78368
C	-0.94715	4.99567	1.64389
N	-2.49479	2.44242	-0.58515
C	-2.35536	1.0738	-0.5419
N	-3.44412	0.61877	-1.25977
C	-4.20332	1.67845	-1.76896
C	-3.60983	2.82917	-1.3358
Ni	-0.98574	0.00381	0.1413
Sn	1.52885	-0.62348	-1.52537
N	3.07027	-0.09595	-0.05405
Si	2.64077	-0.00287	1.65274
C	1.67024	1.60554	1.99287
C	2.28114	2.83913	1.31662
C	-3.76057	-0.75308	-1.57572
C	-4.83486	-1.37566	-0.88941
C	-5.21666	-2.66386	-1.31836
C	-4.56032	-3.29824	-2.37932
C	-3.47878	-2.67357	-3.01593
C	-3.04752	-1.39044	-2.62651
C	-5.55175	-0.70348	0.28202
C	-6.10165	-1.7205	1.2979
C	-1.83767	-0.72646	-3.28259
C	-2.22167	0.42566	-4.23298
C	-0.35645	3.29202	-2.32411
C	-1.03719	4.15282	-3.40715
C	-2.87685	3.38376	2.13936
C	-2.54011	3.49732	3.63402
P	0.34604	-1.66939	0.57508
C	1.41926	-1.44101	2.04471
C	-0.44939	-3.29707	0.65621
C	-1.27585	-3.69151	-0.42151
C	-1.97376	-4.90478	-0.37633
C	-1.85026	-5.75023	0.74271
C	-1.02735	-5.36607	1.8154

C	-0.33379	-4.14494	1.77853
C	-1.91485	-0.53516	1.71093
C	-1.64353	0.28113	2.83653
C	-2.15728	-0.02274	4.10983
C	-2.96479	-1.15922	4.29284
C	-3.25674	-1.97043	3.18369
C	-2.74705	-1.6576	1.90939
C	4.37793	-0.47412	-0.47855
C	5.25127	0.50798	-1.04578
C	6.56541	0.14994	-1.40061
C	7.03592	-1.15939	-1.22834
C	6.16669	-2.13818	-0.72826
C	4.84236	-1.82629	-0.36389
C	4.74043	1.9122	-1.33529
C	5.66429	3.02669	-0.8193
C	3.90724	-2.94945	0.07383
C	4.44547	-3.78449	1.24753
C	4.21007	-0.22381	2.72153
C	3.9196	-0.67275	4.165
C	5.14357	0.99799	2.67551
C	-6.71048	0.20972	-0.17658
C	-0.92703	-1.72124	-4.0171
C	1.12773	3.0684	-2.64862
C	-4.22836	4.0707	1.84704
C	4.46687	2.06619	-2.84454
C	3.53251	-3.84883	-1.1207
C	1.44303	1.8442	3.49538
H	0.05687	0.73399	-0.88236
H	-1.4204	-2.14576	-4.91723
H	4.42124	-4.37938	-1.52403
H	-2.82866	0.04761	-5.08297
H	-2.95378	-3.1986	-3.82592
H	-2.39898	-6.70433	0.77985
H	-0.00632	-1.20724	-4.35855
H	2.7779	-4.60685	-0.82207
H	-1.30553	0.89322	-4.6496
H	-0.93383	-6.01687	2.69935
H	-4.88438	-4.29921	-2.70511
H	3.10089	-3.24981	-1.95047
H	-0.61482	-2.56386	-3.36648
H	-2.62479	-5.18574	-1.21834
H	5.3647	-4.34338	0.97092
H	6.52123	-3.17703	-0.62554
H	3.68999	-4.53138	1.57143
H	-2.79988	1.22163	-3.72703
H	1.23643	2.51433	-3.6032
H	8.07076	-1.42019	-1.50103
H	0.28025	-3.8376	2.63811
H	-1.25081	-0.28857	-2.44085
H	-1.39702	-3.02181	-1.2863
H	2.96885	-2.46873	0.40684
H	1.62582	2.47644	-1.85534
H	4.69314	-3.14715	2.12077
H	-6.04114	-3.17999	-0.80635
H	-0.90819	3.69337	-4.40933

H	1.68165	4.02411	-2.75822
H	-0.84184	2.29516	-2.32366
H	7.23502	0.91575	-1.8252
H	3.73943	1.30545	-3.19785
H	-5.06938	1.50864	-2.41316
H	1.9793	-2.37263	2.26965
H	5.39877	1.93234	-3.43467
H	-0.59703	5.172	-3.43872
H	-7.4495	-0.36689	-0.77238
H	-2.12472	4.25951	-3.22194
H	4.74152	-1.05731	2.20812
H	-6.366	1.06377	-0.78758
H	-7.01645	-2.222	0.91603
H	1.01811	5.37172	-1.13854
H	4.05519	3.07029	-3.07907
H	-3.86124	3.88095	-1.49582
H	0.77409	-1.19318	2.91544
H	5.39894	1.27484	1.63288
H	3.77265	1.99408	-0.80315
H	-5.35931	-2.4996	1.54918
H	-4.79141	-0.0792	0.79955
H	6.64456	3.03313	-1.34257
H	-7.23779	0.62686	0.70652
H	6.09904	0.79097	3.20656
H	3.29984	-1.59264	4.20262
H	0.67551	1.41831	1.51432
H	-6.37718	-1.20288	2.23853
H	4.86523	-0.88859	4.71058
H	5.86445	2.91557	0.26591
H	1.02294	0.958	4.01428
H	4.68483	1.88513	3.16178
H	5.19978	4.02274	-0.97866
H	-2.98576	2.30363	1.90375
H	-4.55492	3.91815	0.79998
H	2.2367	2.75539	0.21412
H	0.65129	6.36753	1.11793
H	3.38325	0.10864	4.74251
H	2.39173	2.1134	4.00683
H	3.34469	2.9878	1.59644
H	0.73797	2.68946	3.66077
H	-1.07425	5.42598	2.64785
H	-5.02019	3.65967	2.50738
H	-4.16179	5.16422	2.02994
H	1.72654	3.75699	1.59819
H	-1.53314	3.09308	3.8597
H	-3.27591	2.92299	4.23158
H	-2.57386	4.54989	3.98679
H	-2.97687	-2.32488	1.06485
H	-3.87886	-2.87129	3.31212
H	-3.35716	-1.41271	5.29039
H	-1.91514	0.63067	4.96421
H	-1.00281	1.16768	2.72317

Table S29. Cartesian geometry of TS1 (18.3 kcal/mol) in Figure S105 in Angstrom [Å].

Atomtype	X Coordinates	Y Coordinates	Z Coordinates

C	-4.51742	1.87777	-0.51956
C	-4.2472	0.47435	-0.60477
C	-5.18546	-0.37046	-1.28367
C	-6.37173	0.18062	-1.80039
C	-6.64865	1.55132	-1.68453
C	-5.71668	2.38701	-1.05776
N	-3.0576	-0.09744	-0.07602
Si	-2.81791	-0.4602	1.61706
C	-1.66058	0.90347	2.34795
P	-0.2905	1.21409	1.16292
Sn	-1.30651	0.30433	-1.32559
Ni	0.98009	-0.25809	0.29514
C	2.56884	-0.81166	-0.54568
N	3.55038	-0.13352	-1.24925
C	4.48565	-1.01139	-1.8128
C	4.10653	-2.27463	-1.45345
N	2.94772	-2.13534	-0.68755
C	3.55727	1.27849	-1.53522
C	4.4185	2.12615	-0.79264
C	4.48719	3.48168	-1.17535
C	3.73308	3.96953	-2.24888
C	2.86391	3.11866	-2.94453
C	2.75116	1.7557	-2.6044
C	2.12087	-3.2398	-0.27653
C	1.21715	-3.77411	-1.22971
C	0.46102	-4.89954	-0.85117
C	0.5862	-5.44974	0.43057
C	1.45375	-4.8717	1.36863
C	2.24369	-3.75182	1.03908
C	5.25082	1.60438	0.3775
C	6.57808	0.96624	-0.08824
C	1.78874	0.83856	-3.35768
C	0.66418	1.60496	-4.06971
C	1.06808	-3.16296	-2.6194
C	-0.39325	-3.13534	-3.09504
C	3.18996	-3.11032	2.05243
C	4.67184	-3.40736	1.73726
C	-4.85267	-1.84119	-1.49568
C	-4.03846	-2.01547	-2.79301
C	-3.52914	2.8431	0.1242
C	-2.99677	3.8905	-0.86999
C	0.0216	3.00566	1.07767
C	0.73208	3.50472	-0.03596
C	0.98888	4.87803	-0.15176
C	0.52829	5.77015	0.83434
C	-0.16838	5.27553	1.95116
C	-0.41076	3.89817	2.08155
C	1.33161	0.60469	2.14778
C	2.38267	1.54141	2.37131
C	3.18706	1.46401	3.51242
C	2.97294	0.46225	4.48186
C	1.93574	-0.46416	4.28762
C	1.12409	-0.39522	3.14614
C	5.56182	2.69836	1.41282
C	2.51231	-0.07618	-4.36768

C	1.95981	-3.88681	-3.64891
C	2.88219	-3.5223	3.49817
C	-1.86322	-2.10942	1.81217
C	-1.81118	-2.60326	3.26809
C	-4.48764	-0.36788	2.543
C	-5.38518	-1.58584	2.26469
C	-2.31989	-3.21539	0.85222
C	-6.07159	-2.77505	-1.4858
C	-4.10297	3.50703	1.3879
C	-4.34682	-0.09379	4.05116
H	-0.28007	-1.20535	-0.69142
H	1.04737	2.18259	-4.93786
H	-3.8057	4.55381	-1.24374
H	3.03574	0.5288	-5.13815
H	2.25679	3.52237	-3.76647
H	0.71467	6.85084	0.73383
H	-0.0922	0.89218	-4.45535
H	-2.22414	4.52582	-0.38817
H	1.77642	-0.725	-4.88634
H	-0.52116	5.96693	2.7328
H	3.81276	5.02919	-2.53867
H	-2.53175	3.39709	-1.74874
H	0.1394	2.30402	-3.38788
H	1.54378	5.25026	-1.02589
H	-4.97709	4.14923	1.14815
H	-5.92093	3.46803	-0.98292
H	-3.33857	4.14873	1.87487
H	3.25554	-0.73749	-3.88404
H	-0.48725	-2.52227	-4.01501
H	-7.58414	1.96631	-2.09204
H	-0.93637	3.51547	2.96952
H	1.31929	0.18841	-2.58292
H	1.08086	2.80564	-0.8128
H	-2.65592	2.23753	0.43176
H	-1.0512	-2.69241	-2.32261
H	-4.44394	2.74996	2.12427
H	5.14234	4.16831	-0.62117
H	1.85891	-3.41903	-4.65047
H	-0.77409	-4.14988	-3.33713
H	1.4099	-2.11	-2.5471
H	-7.09411	-0.4753	-2.31113
H	-3.12796	-1.37885	-2.78179
H	5.31331	-0.65663	-2.43243
H	-2.25249	1.83129	2.47453
H	-4.63764	-1.71815	-3.67992
H	1.66772	-4.95452	-3.73904
H	7.18525	1.70006	-0.65954
H	3.0303	-3.85139	-3.36671
H	-4.9776	0.51954	2.07712
H	6.41818	0.07862	-0.72733
H	6.30934	3.42314	1.02653
H	-0.24969	-5.33952	-1.56622
H	-3.71368	-3.06918	-2.92826
H	4.55021	-3.25047	-1.66906
H	-1.23668	0.63235	3.33717

H	-5.53219	-1.7467	1.17891
H	-4.18471	-2.12681	-0.65987
H	4.65775	3.26867	1.70393
H	4.64218	0.81827	0.87577
H	-6.72977	-2.61524	-2.36653
H	7.17434	0.63937	0.78908
H	-6.39185	-1.45552	2.72029
H	-3.76689	0.8294	4.2589
H	-0.82695	-1.83039	1.49784
H	5.99495	2.24297	2.3257
H	-5.34464	0.0291	4.52839
H	-6.68975	-2.63173	-0.57547
H	-1.51259	-1.80864	3.98442
H	-4.95347	-2.51802	2.68798
H	-5.74499	-3.83579	-1.51392
H	3.03416	-2.01082	1.97523
H	4.97856	-3.02225	0.74648
H	-2.21534	-2.89241	-0.20267
H	-0.01368	-6.3297	0.71106
H	-3.83752	-0.92876	4.57688
H	-2.80022	-2.98394	3.60126
H	-3.38088	-3.49833	1.01723
H	-1.08502	-3.43921	3.37952
H	1.51713	-5.30261	2.37732
H	5.32626	-2.92961	2.49578
H	4.86569	-4.50091	1.75837
H	-1.70091	-4.12799	0.98153
H	1.8171	-3.35879	3.75619
H	3.49667	-2.92327	4.19863
H	3.12083	-4.59239	3.67811
H	2.54537	2.34595	1.63837
H	3.98475	2.20632	3.66369
H	3.5952	0.42339	5.38932
H	1.73833	-1.24012	5.04289
H	0.30438	-1.11713	3.03081

Table S30. Cartesian geometry of TS2 (22.4 kcal/mol) in Figure S105 in Angstrom [Å].

Atomtype	X Coordinates	Y Coordinates	Z Coordinates
C	2.9516	-3.59082	-0.51305
C	2.35405	-2.65856	-1.39435
C	1.04798	-2.81857	-1.91955
C	0.33154	-3.96846	-1.54068
C	0.89716	-4.91049	-0.66669
C	2.19249	-4.72598	-0.16354
N	3.05676	-1.44496	-1.70479
C	2.83168	-0.31368	-0.93675
N	3.70172	0.60493	-1.48713
C	4.46216	0.04807	-2.52673
C	4.05124	-1.2509	-2.66165
Ni	1.88205	-0.47177	0.70007
C	1.68372	-1.11063	2.5166
C	1.82556	-0.27375	3.65541
C	1.56996	-0.74402	4.95529
C	1.18167	-2.07957	5.16753
C	1.05654	-2.93437	4.05859

C	1.3046	-2.45712	2.75854
C	3.90225	1.95777	-1.04396
C	3.00947	2.96861	-1.48297
C	3.33234	4.29939	-1.1573
C	4.47628	4.60556	-0.40597
C	5.29588	3.58047	0.08203
C	5.02309	2.23101	-0.2233
C	1.71615	2.61214	-2.2127
C	0.68182	3.7453	-2.16183
C	5.83934	1.10667	0.40462
C	7.35043	1.38768	0.42463
C	0.47397	-1.77256	-2.86866
C	-1.05591	-1.75899	-2.893
C	4.3686	-3.38948	0.01027
C	5.37061	-4.19341	-0.84413
P	-0.04961	0.56852	0.7312
C	-1.33446	0.08093	2.01138
Si	-2.78782	-0.8684	1.25084
C	-4.28497	-0.91657	2.44125
C	-5.37969	-1.90893	2.00843
C	0.31603	2.32054	1.2239
C	1.65159	2.69995	1.47759
C	1.96138	4.01775	1.84984
C	0.94459	4.98197	1.95524
C	-0.39071	4.61688	1.70337
C	-0.70082	3.29418	1.35059
Sn	-1.71001	1.13691	-1.26766
N	-3.23898	0.12928	-0.18777
C	-4.61324	0.42138	-0.46802
C	-5.34286	-0.30106	-1.45971
C	-6.71657	-0.04194	-1.63387
C	-7.3804	0.92209	-0.8657
C	-6.64902	1.68149	0.05744
C	-5.27281	1.46501	0.25808
C	-4.64084	-1.27891	-2.38848
C	-4.3686	-0.60863	-3.75029
C	-4.48749	2.42565	1.14679
C	-4.12179	3.678	0.32248
C	-5.38989	-2.6096	-2.56087
C	-5.1997	2.81863	2.4503
C	1.94287	2.15769	-3.66881
C	5.28477	0.81811	1.81618
C	1.02781	-1.94047	-4.29836
C	4.50999	-3.71948	1.50488
C	-2.1103	-2.61287	0.82864
C	-1.88002	-3.38959	2.14036
C	-2.91375	-3.48127	-0.14866
C	-3.88654	-1.10063	3.91807
H	3.1593	-1.12354	1.28131
H	1.013	4.62973	-2.74607
H	-5.03789	4.18364	-0.04756
H	2.37738	2.98092	-4.27458
H	2.66719	5.11294	-1.47651
H	1.18997	6.01716	2.24022
H	-0.27504	3.40778	-2.6109

H	-3.5424	4.40975	0.92365
H	0.97551	1.86874	-4.13127
H	-1.19568	5.36447	1.7877
H	4.70947	5.65572	-0.16964
H	-3.51601	3.41405	-0.57291
H	0.47632	4.06926	-1.12178
H	3.00831	4.28848	2.05123
H	-6.10944	3.42665	2.26222
H	-7.15749	2.4731	0.62985
H	-4.52583	3.42668	3.08878
H	2.62096	1.2854	-3.73744
H	-1.42742	-0.91967	-3.5157
H	-8.4587	1.0989	-1.00215
H	-1.75158	3.01268	1.18221
H	1.29864	1.74048	-1.65448
H	2.44543	1.94253	1.37767
H	-3.54132	1.91557	1.42237
H	-1.47549	-1.65717	-1.87075
H	-5.50579	1.9249	3.03154
H	6.16225	3.82717	0.71428
H	0.63585	-1.14332	-4.96416
H	-1.4833	-2.68716	-3.32746
H	0.81171	-0.78332	-2.4859
H	-7.27657	-0.61046	-2.39326
H	-3.76041	0.31295	-3.6282
H	5.20833	0.63442	-3.07081
H	-1.72164	1.00309	2.49313
H	-5.31839	-0.31441	-4.24527
H	0.72601	-2.92223	-4.72047
H	7.60707	2.23473	1.09524
H	2.13297	-1.88383	-4.32223
H	-4.71809	0.10209	2.3425
H	7.73662	1.62759	-0.58777
H	5.35061	1.71869	2.46258
H	-0.68722	-4.12829	-1.91928
H	-3.82405	-1.29458	-4.43318
H	4.37163	-2.04199	-3.34564
H	-0.80621	-0.50316	2.794
H	-5.69929	-1.74314	0.95877
H	-3.66051	-1.49437	-1.92309
H	4.22489	0.49457	1.75304
H	5.67797	0.19051	-0.20136
H	-6.34622	-2.47866	-3.10997
H	7.89949	0.50029	0.80133
H	-6.28353	-1.80561	2.64863
H	-3.13708	-0.35138	4.24637
H	-1.10622	-2.40926	0.38114
H	5.85008	-0.00324	2.30412
H	-4.77487	-0.99702	4.58026
H	-5.62369	-3.07163	-1.58086
H	-1.34873	-2.79667	2.90907
H	-5.03613	-2.96095	2.09927
H	-4.77639	-3.33047	-3.14122
H	4.60353	-2.31126	-0.10704
H	5.30885	-3.9173	-1.9168

H	-2.97108	-3.04365	-1.16133
H	0.31545	-5.79777	-0.37147
H	-3.45177	-2.10251	4.11055
H	-2.84443	-3.73207	2.5717
H	-3.95472	-3.6379	0.20006
H	-1.26397	-4.29605	1.95204
H	2.61925	-5.46751	0.52837
H	6.41165	-4.01258	-0.50331
H	5.17072	-5.28342	-0.76848
H	-2.44991	-4.48772	-0.24739
H	3.78311	-3.14137	2.10979
H	5.53173	-3.46466	1.85482
H	4.35429	-4.79975	1.71014
H	2.12546	0.77932	3.52449
H	1.67622	-0.05857	5.81296
H	0.98587	-2.451	6.18603
H	0.75835	-3.98629	4.20442
H	1.19796	-3.14869	1.90619

6. References

[1] A. J. Sicard, R. T. Baker, "Safe and Expeditious Preparation of Ni(cod)2 for Same-Day High-Throughput Screening" *Org. Process Res. Dev.* **2020**, *24*, 2950–2952.

[2] B.-C. Liu, N. Ge, Y.-Q. Zhai, T. Zhang, Y.-S. Ding, Y.-Z. Zheng, "An imido ligand significantly enhances the effective energy barrier of dysprosium(III) single-molecule magnets" *Chem. Commun.* **2019**, *55*, 9355–9358.

[3] Y. Hoshimoto, Y. Hayashi, H. Suzuki, M. Ohashi, S. Ogoshi, "One-Pot, Single-Step, and Gram-Scale Synthesis of Mononuclear [(η6-arene)Ni(N-heterocyclic carbene)] Complexes: Useful Precursors of the Ni0–NHC Unit" *Organometallics* **2014**, *33*, 1276–1282.

[4] P. M. Keil, T. Szilvási, T. J. Hadlington, "Reversible metathesis of ammonia in an acyclic germylene–Ni0 complex" *Chem. Sci.* **2021**, *12*, 5582–5590.

[5] P. M. Keil, T. J. Hadlington, "Accessing cationic tetrylene-nickel(0) systems featuring donor–acceptor E–Ni triple bonds (E = Ge, Sn)" *Chem. Commun.* **2022**, *58*, 3011–3014.

[6] P. M. Keil, T. J. Hadlington, "Protonation of Hydrido-Tetrylenes: H2 Elimination vs. Tetrylium Cation Formation" *Z. Für Anorg. Allg. Chem.* **2022**, *648*, e202200141.

[7] T. L. Kalkuhl, I. Fernández, T. J. Hadlington, "Cooperative hydrogenation catalysis at a constrained gallylene-nickel(0) interface" *Chem* **2024**, *0*, DOI 10.1016/j.chempr.2024.10.016.

[8] M. Muhr, P. Heiß, M. Schütz, R. Bühler, C. Gemel, M. H. Linden, H. B. Linden, R. A. Fischer, "Enabling LIFDI-MS measurements of highly air sensitive organometallic compounds: a combined MS/glovebox technique" *Dalton Trans.* **2021**, *50*, 9031–9036.

[9] M. R. Elsby, S. A. Johnson, "Nickel-Catalyzed C–H Silylation of Arenes with Vinylsilanes: Rapid and Reversible β-Si Elimination" *J. Am. Chem. Soc.* **2017**, *139*, 9401–9407.

[10] M. J. Frisch, G. W. Trucks, H. B. Schlegel, G. E. Scuseria, M. A. Robb, J. R. Cheeseman, G. Scalmani, V. Barone, G. A. Petersson, H. Nakatsuji, X. Li, M. Caricato, A. V. Marenich, J. Bloino, B. G. Janesko, R. Gomperts, B. Mennucci, H. P. Hratchian, J. V. Ortiz, A. F. Izmaylov, J. L. Sonnenberg, Williams, F. Ding, F. Lipparini, F. Egidi, J. Goings, B. Peng, A. Petrone, T. Henderson, D. Ranasinghe, V. G. Zakrzewski, J. Gao, N. Rega, G. Zheng, W. Liang, M. Hada, M. Ehara, K. Toyota, R. Fukuda, J. Hasegawa, M. Ishida, T. Nakajima, Y. Honda, O. Kitao, H. Nakai, T. Vreven, K. Throssell, J. A. Montgomery Jr., J. E. Peralta, F. Ogliaro, M. J. Bearpark, J. J. Heyd, E. N. Brothers, K. N. Kudin, V. N. Staroverov, T. A. Keith, R. Kobayashi, J. Normand, K. Raghavachari, A. P. Rendell, J. C. Burant, S. S. Iyengar, J. Tomasi, M. Cossi, J. M. Millam, M. Klene, C. Adamo, R. Cammi, J. W. Ochterski, R. L. Martin, K. Morokuma, O. Farkas, J. B. Foresman, D. J. Fox **2016**.

[11] A. V. Marenich, C. J. Cramer, D. G. Truhlar, "Universal solvation model based on solute electron density and on a continuum model of the solvent defined by the bulk dielectric constant and atomic surface tensions" *J. Phys. Chem. B* **2009**, *113*, 6378–6396.

[12] F. Weigend, R. Ahlrichs, "Balanced basis sets of split valence, triple zeta valence and quadruple zeta valence quality for H to Rn: Design and assessment of accuracy" *Phys. Chem. Chem. Phys.* **2005**, *7*, 3297–3305.

[13] S. Grimme, "Semiempirical GGA-type density functional constructed with a long-range dispersion correction" *J. Comput. Chem.* **2006**, *27*, 1787–1799.

[14] J. N. Harvey, F. Himo, F. Maseras, L. Perrin, "Scope and Challenge of Computational Methods for Studying Mechanism and Reactivity in Homogeneous Catalysis" *ACS Catal.* **2019**, *9*, 6803–6813.

[15] E. D. Glendening, A. E. Reed, J. E. Carpenter, F. Weinhold, "NBO Version 3.1" **1998**.

[16] M. D. Hanwell, D. E. Curtis, D. C. Lonie, T. Vandermeersch, E. Zurek, G. R. Hutchison, "Avogadro: an advanced semantic chemical editor, visualization, and analysis platform" *J. Cheminformatics* **2012**, *4*, 17.

[17] *Avogadro: An Open-Source Molecular Builder and Visualization Tool*, Version 1.2.0, https://avogadro.cc/
